# Sex- and tissue-specific transcriptome analyses and expression profiling of olfactory-related genes in *Ceracris nigricornis* Walker (Orthoptera: Acrididae)

**DOI:** 10.1186/s12864-019-6208-x

**Published:** 2019-11-06

**Authors:** Hao Yuan, Huihui Chang, Lina Zhao, Chao Yang, Yuan Huang

**Affiliations:** 1 College of Life Sciences, Shaanxi Normal University, Xi’an, 710062 China; 2 Shaanxi Institute of Zoology, Xi’an, China

**Keywords:** *Ceracris nigricornis*, Transcriptome, Expression profiles analysis, Odorant-binding protein, Chemosensory-binding protein, Odorant receptor, Ionotropic receptor, Sensory neuron membrane protein

## Abstract

**Background:**

The sophisticated insect olfactory system plays an important role in recognizing external odors and enabling insects to adapt to environment. Foraging, host seeking, mating, ovipositing and other forms of chemical communication are based on olfaction, which requires the participation of multiple olfactory genes. The exclusive evolutionary trend of the olfactory system in Orthoptera insects is an excellent model for studying olfactory evolution, but limited olfaction research is available for these species. The olfactory-related genes of *Ceracris nigricornis* Walker (Orthoptera: Acrididae), a severe pest of bamboos, have not yet been reported.

**Results:**

We sequenced and analyzed the transcriptomes from different tissues of *C. nigricornis* and obtained 223.76 Gb clean data that were assembled into 43,603 unigenes with an N50 length of 2235 bp. Among the transcripts, 66.79% of unigenes were annotated. Based on annotation and tBLASTn results, 112 candidate olfactory-related genes were identified for the first time, including 20 odorant-binding proteins (OBPs), 10 chemosensory-binding proteins (CSPs), 71 odorant receptors (ORs), eight ionotropic receptors (IRs) and three sensory neuron membrane proteins (SNMPs). The fragments per kilobase per million mapped fragments (FPKM) values showed that most olfactory-related differentially expressed genes (DEGs) were enriched in the antennae, and these results were confirmed by detecting the expression of olfactory-related genes with quantitative real-time PCR (qRT-PCR). Among these antennae-enriched genes, some were sex-biased, indicating their different roles in the olfactory system of *C. nigricornis*.

**Conclusions:**

This study provides the first comprehensive list and expression profiles of olfactory-related genes in *C. nigricornis* and a foundation for functional studies of these olfactory-related genes at the molecular level.

## Background

*Ceracris nigricornis* Walker (Orthoptera: Acrididae) is a severe grasshopper pest of bamboos such as *Phyllostachys heterocycla*, *Phyllostachys viridis* and *Phyllostachys glauca*. *C. nigricornis* can also harm rice, corn, sorghum and other crops and can cause serious economic losses. Typically, the application of a substantial quantity of chemical insecticides, especially wide-spectrum insecticides, is the main method for controlling this pest. However, long-term application of pesticides may lead to pesticide resistance, pesticide residues, environmental pollution, and a decrease in the natural enemies of *C. nigricornis* [1–4]. In recent years, the use of eco-friendly nonhost plant volatiles to control phytophagous insects has increased; for example, plant volatiles from *Trifolium repens* L., *Castanea mollissima* Blume, *Citrus reticulata* Blanco, *Kigelia africana* (Lam.) and *Myrica rubra* (Lour.) have been used to interfere with the orientation and selection of plant volatiles of tea leaves in the olfactory system of *Empoasca vitis*, which reduces the level of *E. vitis* [5–7]. The ability of these nonhost plant volatiles to control the level of insects depends largely on the highly sensitive insect olfactory system [8]. Therefore, the elucidation of the molecular basis of the insect olfactory system is of great importance for new bio-pesticide development and pest control.

Olfaction is the primary sensory modality in insects and plays an important role in finding mating partners, food, oviposition sites and suitable habitats [9–11]. The insect olfactory system involves several different proteins, including binding proteins (odorant-binding proteins, OBPs; and chemosensory-binding proteins, CSPs), chemosensory membrane proteins (odorant receptors, ORs; ionotropic receptors, IRs; gustatory receptors, GRs; and sensory neuron membrane proteins, SNMPs), and odorant-degrading esterases (ODEs) [12, 13]. OBPs and CSPs are highly concentrated in the lymph of chemosensilla and are regarded as carriers of pheromones and odorants in insect chemoreception [13–15]. OBPs are small globular, water-soluble proteins that generally contain six highly conversed cysteine residues paired into three interlocking disulfide bridges [16, 17]. OBPs can bind and transport external odorant molecules to the olfactory receptors in the olfactory neuronal membrane, which is often considered the first step in olfactory recognition [18, 19]. CSPs are also small soluble proteins, also known as olfactory system of *Drosophila melanogaster* (OS-D)-like proteins or sensory appendage proteins, which contain only four conserved cysteine residues but have more conserved nucleotide sequences than OBPs across insect species [20–22]. CSPs are expressed in various chemosensory organs and have many functions. CSPs are also present in nonchemosensory organs and play a role in the transmission of pheromones, the solubility of nutrients, and the development of insecticide resistance [23–25].

The recognition and transmission of olfaction begins with the interaction between odorant molecules and ORs on the dendrites of olfactory receptor neurons (ORNs). Insect ORs were first identified in the *D. melanogaster* genome; ORs contain seven transmembrane domains (TMDs) and a membrane topology with an intracellular N-terminus and an extracellular C-terminus, and the membrane topology of insect ORs are reversed compared to that of vertebrate ORs [26]. ORs are nonselective cationic channels with high selectivity and specificity for odorant molecules. ORs can convert chemical signals of odorant molecules into electrical signals and play a role as a transit station in insect olfactory reactions. IR is a newly discovered gene family that was first studied in the olfactory system of *D. melanogaster* [27]. IRs evolved from the ionotropic glutamate receptor superfamily (iGluRs) and contains iGluRs conserved structural regions: three TMDs, a bipartite ligand-binding domain with two lobes and one ion channel pore [28]. IRs are expressed in coeloconic olfactory sensory neurons (OSNs) without ORs or coreceptors (ORcos) and mainly function in detecting acids, amines and other chemicals that cannot be recognized by ORs [29]. SNMPs are double transmembrane proteins that have a transmembrane domain at the C- and N-termini of the chain. SNMPs belong to the CD36 receptor family and are divided into two subfamilies, SNMP1 and SNMP2 [30]. The SNMP1 subfamily is coexpressed with pheromone receptors, and in situ hybridization indicated that it is associated with pheromone-sensitive neurons [31]. SNMP1 has been confirmed to participate in pheromone signal transduction. The SNMP2 subfamily was first identified from *Manduca sexta* and associates with pheromone-sensitive neurons, but it was expressed only in sensilla support cells [32].

Locusts and grasshoppers are major economic pests, but their genetic information is lacking, partly because their genomes are often very large. Currently, there are data for more than 100 genomes of Orthoptera species in the Genome Size Database (www.genomesize.com). The known variation in Orthoptera genome size ranges from 1.52 Gb for the cave cricket (*Hadenoecus subterraneus*) to 16.56 Gb for the mountain grasshopper (*Podisma pedestris*), with an 11-fold difference in size [33, 34]. Sequencing large genomes has higher requirements for sequencing technology and for human and material resources than sequencing small genomes, which explains why only the migratory locust *Locusta migratoria* (genome size is ~ 6.3 Gb) of Orthoptera has a complete genome sequence thus far [35]. Transcriptomic approaches offer an alternative to genomic approaches; transcriptomic approaches can generate almost all transcripts of a specific organ or tissue of a certain species in a comprehensive and rapid manner, and most molecular mechanisms of different biological processes are also elucidated in the transcriptome [36]. Transcriptomic sequencing results have less data and are more convenient for analysis than genomic sequencing results.

In recent years, there have been increasing reports on the transcriptome of the order Orthoptera, and such reports potentially provide resources for advancing the postgenomic research of Orthoptera insects; however, limited olfaction research is available for these species. Thus far, olfaction studies have been published regarding only *L. migratoria* [37–41], *Oedaleus asiaticus* [42], *Oedaleus infernalis* [43], *Ceracris kiangsu* [44] and *Schistocerca gregaria* [16, 45–47], and most investigations have focused on OBP genes. Here, we present a de novo transcriptome assembly for the bamboo grasshopper *C. nigricornis* and identified 112 putative olfactory-related genes comprising 20 OBPs, 10 CSPs, 71 ORs, 8 IRs and 3 SNMPs. Then, we evaluated the distribution of the expression patterns of these genes in different tissues of female and male adults by transcriptome analyses and quantitative real-time PCR (qRT-PCR). Our study provides the foundation for further studies of the molecular mechanism regulating the olfactory system in *C. nigricornis*.

## Results

### Sequencing and de novo assembly

The transcriptomes of the antennae (A), head (antennae were cut off; H), abdomen-thorax (T), legs (L) and wings (W) of female and male *C. nigricornis* were separately sequenced using the Illumina HiSeq X Ten platform. After the low-quality reads were filtered, a total of 223.76 Gb clean data were obtained from all 30 tissue samples, and the clean data of each tissue sample reached 6.30 Gb with a Q30 percentage greater than 94% (Additional file 1: Table S1). After all of the samples were assembled, 43,603 unigenes were generated with an N50 length of 2235 bp, and among them, 20,914 unigenes (47.96%) had a length of over 1 kb (Additional file 1: Table S2). To assess the transcriptome assembly completeness, the benchmarking sets of universal single-copy orthologs (BUSCO) v3.0.2 completeness assessment tool was used together with the Insecta odb9 database with 1658 reference genes [48]. The result had a completeness score of 89.1%, a fragmented score of 2.5% and a missing BUSCO score of only 8.4% (Additional file 1: Table S3).

### Functional annotation

A total of 24,832 (56.95%), 15,750 (36.12%), 13,150 (30.16%), 11,503 (26.38%), 18,100 (41.51%), 26,933 (61.77%), 22,295 (51.13%) and 12,102 (27.75%) unigenes were successfully annotated to National Center for Biotechnology Information (NCBI) nonredundant protein sequences (NR), Swiss-Prot (a manually annotated and reviewed protein sequence database), Kyoto Encyclopedia of Genes and Genomes (KEGG), euKaryotic Orthologous Groups of proteins (KOG), Clusters of Orthologous Groups of proteins (COG), EggNOG (A database of orthologous groups and functional annotation), Protein family (Pfam) and Gene Ontology (GO) databases, respectively, which covered a total of 29,122 (66.79%) unigenes (Additional file 1: Table S4).

A query of the NR database indicated that a high percentage of *C. nigricornis* sequences closely matched insect sequences (11,695, 74.94%). Among these sequences, the highest match sequence and percentage was identified with sequences of *Cryptotermes secundus* (2548, 21.79%), followed by sequences of *Zootermopsis nevadensis* (1765, 15.09%), *Nilaparvata lugens* (883, 7.55%), *L. migratoria* (729, 6.23%), *Rhagoletis zephyria* (223, 1.91%), *Lasius niger* (198, 1.69%), *Bemisia tabaci* (195, 1.67%), *S. gregaria* (144, 1.23%), and *Tribolium castaneum* (141, 1.21%) (Additional file 2: Figure S1).

Blast2GO was applied to classify the functional groups of all unigenes of *C. nigricornis*. As one unigene could align to multiple GO categories, the assigned GO term was apparently larger than the annotated loci. In total, 12,102 unigenes were classified into at least one of the three main GO categories: 8661 (71.57%) were assigned to biological process, 6828 (56.42%) were assigned to cellular component and 9766 (80.70%) were assigned to molecular function. For the biological process (including 22 subcategories) category, metabolic process (5787 unigenes), cellular process (5539 unigenes) and single-organism process (3361 unigenes) were the most highly enriched GO terms, whereas cell (4705 unigenes), cell part (4674 unigenes), and organelle (3263 unigenes) were the most predominant GO terms in the cellular component (including 17 subcategories) category. For molecular function (including 16 subcategories), the most represented GO terms were catalytic activity (5846 unigenes), binding (5140 unigenes) and structural molecule activity (1190 unigenes) (Additional file 2: Figure S2).

### Olfactory-related gene identification

Based on functional annotation and tBLASTn results, a total of 20 candidate OBP genes (CnigOBP1–20) were identified in the transcriptome of *C. nigricornis* (Table 1). All of these candidate OBP genes had six conserved cysteine residues (Additional file 2: Figure S3). Among the 20 OBP genes, 17 had intact open reading frames (ORFs) with lengths ranging from 408 bp to 816 bp. Except for CnigOBP11 and CnigOBP16, all full-length OBPs had a predicted signal peptide (a signature of secretory proteins) at the N-terminal region (Table 1). The conserved domain prediction of these candidate OBP genes showed that all of them had the domain of a pheromone/general odorant-binding protein (PhBP or PBP_GOBP) (InterPro: IPR006170) (Additional file 1: Table S5).

**Table 1:** Summary of odorant binding proteins (OBPs) identified in *C. nigricornis*

Gene name	Accession number	Full length	ORF (bp)	Amino acid length (AA)	Signal peptide (AA)	Homology match			Score	E-value	Identity (%)
						Name	Species	Accession number			
CnigOBP1	MK982654	Y	468	155	1–18	odorant-binding protein 7	*Ceracris kiangsu*	KP255957.1	750	0	95.53
CnigOBP2	MK982655	Y	537	178	1–17	odorant-binding protein 7	*Oedaleus infernalis*	MG507284.1	590	2.67E-168	92.86
CnigOBP3	MK982656	Y	450	149	1–27	odorant binding protein 11	*Schistocerca gregaria*	MF716568.1	577	2.22E-164	89.27
CnigOBP4	MK982657	Y	456	151	1–19	odorant-binding protein 5	*Ceracris kiangsu*	KP255955.1	521	7.04E-148	97.39
CnigOBP5	MK982658	3′	360	119	1–52	odorant-binding protein 2	*Ceracris kiangsu*	KP255952.1	560	2.19E-159	95.95
CnigOBP6	MK982659	Y	459	152	1–21	odorant-binding protein 1	*Ceracris kiangsu*	KP255951.1	798	0	98.04
CnigOBP7	MK982660	Y	465	154	1–18	odorant-binding protein 4	*Ceracris kiangsu*	KP255954.1	704	0	93.98
CnigOBP8	MK982661	M	318	105	NO	odorant binding protein 1	*Schistocerca gregaria*	MF716558.1	405	1.11E-112	89.51
CnigOBP9	MK982662	Y	474	157	1–21	odorant-binding protein 16	*Oedaleus infernalis*	MG507293.1	401	1.43E-111	83.76
CnigOBP10	MK982663	Y	468	155	1–24	odorant-binding protein 8	*Ceracris kiangsu*	KP255958.1	693	0	93.38
CnigOBP11	MK982664	Y	495	164	NO	odorant-binding protein 15	*Oedaleus infernalis*	MG507292.1	274	3.58E-73	78.18
CnigOBP12	MK982665	Y	471	156	1–18	odorant-binding protein 3	*Oedaleus infernalis*	MG507280.1	483	5.22E-136	85.2
CnigOBP13	MK982666	Y	456	151	1–19	odorant-binding protein 3	*Ceracris kiangsu*	KP255953.1	684	0	93.55
CnigOBP14	MK982667	Y	816	271	1–22	odorant binding protein 12	*Schistocerca gregaria*	MF716569.1	1105	0	91.09
CnigOBP15	MK982668	3′	642	213	1–20	odorant-binding protein 7	*Schistocerca gregaria*	MF716564.1	372	1.00E-98	80.2
CnigOBP16	MK982669	Y	468	155	NO	odorant-binding protein 6	*Ceracris kiangsu*	KP255956.1	693	0	93.59
CnigOBP17	MK982670	Y	417	138	1–30	odorant binding protein 17	*Locusta migratoria*	MH176616.1	429	1.03E-119	85.57
CnigOBP18	MK982671	Y	480	159	1–25	odorant-binding protein 18	*Oedaleus infernalis*	MG507295.1	647	0	92.31
CnigOBP19	MK982672	Y	408	135	1–26	odorant-binding protein 4	*Oedaleus infernalis*	MG507281.1	549	4.36E-156	90.93
CnigOBP20	MK982673	Y	438	145	1–43	odorant binding protein 5	*Schistocerca gregaria*	MF716562.1	556	2.79E-158	89.73

The mark of Y, 5′, 3′, and M means that the fragment of the unigene consists of complete open reading frame, 5′-end containing start codon, 3′-end containing stop codon, and the middle part without start and stop codon, respectively

A total of 10 candidate CSP genes (CnigCSP1–10) were identified from the transcriptomes of different tissues of *C. nigricornis* (Table 2). All of these candidate CSP genes had four conserved cysteine residues and a conserved OS-D domain (InterPro: IPR005055); however, for CnigCSP9, two OS-D domains were identified by the conserved domain prediction (Additional file 2: Figure S4 and Additional file 1: Table S6). Among the 10 CSP genes, six CSP genes had full-length ORFs, the remaining CSP genes were incomplete due to a lack of a 5′ or 3′ terminus. The SignalP tests showed that all full-length CSP genes had a predicted signal peptide (Table 2).

**Table 2:** Summary of chemosensory proteins (CSPs) identified in *C. nigricornis*

Gene name	Accession number	Full length	ORF (bp)	Amino acid length (AA)	Signal peptide (AA)	Homology match			Score	E-value	Identity (%)
						Name	Species	Accession number			
CnigCSP1	MK989603	Y	447	148	1–17	chemosensory protein 7	*Oedaleus infernalis*	MH568703.1	490	2.57E-137	88.107
CnigCSP2	MK989604	Y	381	126	1–21	chemosensory protein 8	*Oedaleus infernalis*	MH568704.1	324	6.65E-88	82.105
CnigCSP3	MK989605	Y	462	153	1–35	chemosensory protein 12	*Oedaleus asiaticus*	KX905068.1	287	3.66E-77	82.769
CnigCSP4	MK989606	Y	453	150	1–19	chemosensory protein 2	*Oedaleus asiaticus*	KX905058.1	473	2.57E-133	88.718
CnigCSP5	MK989607	Y	396	131	1–21	chemosensory protein 9	*Oedaleus asiaticus*	KX905065.1	366	4.57E-101	83.459
CnigCSP6	MK989608	Y	384	127	1–16	chemosensory protein 9	*Oedaleus infernalis*	MH568705.1	438	1.75E-122	86.99
CnigCSP7	MK989609	3′	333	110	1–29	chemosensory protein 23	*Oedaleus infernalis*	MH568719.1	243	1.74E-63	90.374
CnigCSP8	MK989610	3′	393	130	NO	chemosensory protein 10	*Oedaleus infernalis*	MH568706.1	355	2.38E-97	87.5
CnigCSP9	MK989611	M	693	230	1–18	chemosensory protein 17	*Oedaleus infernalis*	MH568713.1	300	3.01E-81	87.405
CnigCSP10	MK989612	3′	375	124	1–19	chemosensory protein 1	*Oedaleus asiaticus*	KX905057.1	457	2.76E-128	88.714

The mark of Y, 5′, 3′, and M means that the fragment of the unigene consists of complete open reading frame, 5′-end containing start codon, 3′-end containing stop codon, and the middle part without start and stop codon, respectively

In the transcriptomes of *C. nigricornis*, 71 candidate OR genes were identified, including 70 conventional ORs (CnigOR1–70) and one ORco (CnigORco). Of these, only 15 candidate OR genes had complete ORFs with lengths longer than 394 amino acids and had 4–7 TMDs (Table 3). Eight candidate IR genes were identified (CnigIR1–5, CnigIR8a, CnigIR25a and CnigIR76b), and six contained a full-length ORF with lengths longer than 319 amino acids (Table 4). Three candidate SNMPs were identified and named CnigSNMP1, CnigSNMP2 and CnigSNMP2a. Only CnigSNMP1 had complete ORFs encoding 532 amino acids (Table 5).

**Table 3:** Summary of odorant receptors (ORs) identified in *C. nigricornis*

Gene name	Accession number	Full length	ORF (bp)	Amino acid length (AA)	Tm domain	Homology match			Score	E-value	Identity (%)
						Name	Species	Accession number			
CnigOR1	MN004970	3′	522	173	1	odorant receptor 115	Locusta migratoria	KP843300.1	641	6.00E-180	89.13%
CnigOR2	MN004971	M	315	104	2	odorant receptor 44	Locusta migratoria	KP843275.1	250	2.00E-62	82.41%
CnigOR3	MN004972	M	558	185	3	odorant receptor 17	Locusta migratoria	KP843324.1	494	2.00E-135	86.37%
CnigOR4	MN004973	3′	420	139	0	odorant receptor 62	Schistocerca gregaria	KY964979.1	523	2.00E-144	90.07%
CnigOR5	MN004974	3′	1122	373	5	odorant receptor 116	Schistocerca gregaria	KY965033.1	566	8.00E-157	90.47%
CnigOR6	MN004975	M	747	248	2	odorant receptor 125	Locusta migratoria	KP843195.1	346	8.00E-91	77.36%
CnigOR7	MN004976	5′	351	116	0	odorant receptor 20	Locusta migratoria	KP843332.1	342	5.00E-90	85.63%
CnigOR8	MN004977	3′	591	193	2	odorant receptor 74	Schistocerca gregaria	KY964991.1	566	4.00E-157	83.95%
CnigOR9	MN004978	5′	1257	418	4	odorant receptor 129	Locusta migratoria	KP843262.1	1027	0	81.59%
CnigOR10	MN004979	3′	1464	487	6	odorant receptor 84	Schistocerca gregaria	KY965001.1	496	1.00E-135	78.20%
CnigOR11	MN004980	3′	873	290	2	odorant receptor 22	Locusta migratoria	KP843343.1	640	4.00E-179	80.23%
CnigOR12	MN004981	3′	1077	358	0	odorant receptor 94	Locusta migratoria	KP843364.1	977	0	83.46%
CnigOR13	MN004982	Y	1185	394	6	odorant receptor 114	Locusta migratoria	KP843317.1	555	2.00E-153	81.41%
CnigOR14	MN004983	5′	1185	394	4	odorant receptor 46	Locusta migratoria	KP843249.1	1288	0	86.30%
CnigOR15	MN004984	3′	567	188	2	odorant receptor 8	Schistocerca gregaria	KY964925.1	582	4.00E-162	86.22%
CnigOR16	MN004985	5′	1110	369	4	odorant receptor 57	Locusta migratoria	KP843340.1	1408	0	89.56%
CnigOR17	MN004986	3′	717	238	1	odorant receptor 68	Schistocerca gregaria	KY964985.1	758	0.00E+ 00	86.61%
CnigOR18	MN004987	5′	450	149	2	odorant receptor 70	Locusta migratoria	KP843266.1	379	5.00E-101	82.04%
CnigOR19	MN004988	3′	183	60	0	olfactory receptor OR10	Oedaleus asiaticus	MH196282.1	272	3.00E-69	95.83%
CnigOR20	MN004989	M	1086	361	5	odorant receptor 11	Oedaleus asiaticus	MH196283.1	388	2.00E-103	89.10%
CnigOR21	MN004990	3′	1050	349	6	odorant receptor 92	Locusta migratoria	KP843261.1	1282	0	88.72%
CnigOR22	MN004991	3′	1104	367	4	odorant receptor 112	Locusta migratoria	KP843264.1	1109	0	85.13%
CnigOR23	MN004992	5′	1182	394	3	odorant receptor 59	Locusta migratoria	KP843311.1	1208	0	88.62%
CnigOR24	MN004993	3′	807	268	4	odorant receptor 140	Locusta migratoria	KP843287.1	872	0.00E+ 00	89.97%
CnigOR25	MN004994	Y	1272	423	7	odorant receptor 39	Locusta migratoria	KP843237.1	1399	0	87.00%
CnigOR26	MN004995	M	1287	428	6	odorant receptor 98	Locusta migratoria	KP843339.1	1301	0	87.59%
CnigOR27	MN004996	3′	1296	431	4	odorant receptor 63	Locusta migratoria	KP843243.1	593	4.00E-165	84.40%
CnigOR28	MN004997	M	1413	470	5	odorant receptor 86	Schistocerca gregaria	KY965003.1	372	2.00E-98	73.31%
CnigOR29	MN004998	3′	1401	466	4	odorant receptor 15	Locusta migratoria	KP843322.1	595	1.00E-165	87.67%
CnigOR30	MN004999	Y	1347	448	6	odorant receptor 1	Locusta migratoria	JQ766965.1	1742	0	89.35%
CnigOR31	MN005000	3′	1287	428	4	odorant receptor 3	Locusta migratoria	KP843242.1	1568	0	89.17%
CnigOR32	MN005001	3′	1296	431	6	odorant receptor 35	Schistocerca gregaria	KY964952.1	1020	0	85.28%
CnigOR33	MN005002	3′	1341	446	2	odorant receptor 105	Locusta migratoria	KY965022.1	198	4.00E-46	75.73%
CnigOR34	MN005003	3′	909	302	3	odorant receptor 85	Locusta migratoria	KP843252.1	1044	0	87.31%
CnigOR35	MN005004	3′	990	329	4	odorant receptor 84	Schistocerca gregaria	KY965001.1	773	0	87.50%
CnigOR36	MN005005	5′	375	124	2	odorant receptor 123	Locusta migratoria	KP843260.1	294	2.00E-75	80.91%
CnigOR37	MN005006	3′	1128	375	4	odorant receptor 49	Locusta migratoria	KP843251.1	1214	0	86.12%
CnigOR38	MN005007	3′	711	236	3	odorant receptor 33	Locusta migratoria	KY964950.1	448	2.00E-121	78.06%
CnigOR39	MN005008	M	1071	356	4	odorant receptor 11	Locusta migratoria	KP843352.1	787	0.00E+ 00	83.67%
CnigOR40	MN005009	M	1185	394	5	olfactory receptor OR41	Oedaleus asiaticus	MH196313.1	518	3.00E-142	86.62%
CnigOR41	MN005010	3′	192	63	0	odorant receptor 21	Schistocerca gregaria	KY964938.1	237	1.00E-58	90.11%
CnigOR42	MN005011	Y	1293	430	6	odorant receptor 31	Locusta migratoria	KP843247.1	1205	0	83.77%
CnigOR43	MN005012	3′	1047	348	5	odorant receptor 89	Locusta migratoria	KP843305.1	1098	0	89.66%
CnigOR44	MN005013	Y	1251	416	5	odorant receptor 77	Locusta migratoria	KP843362.1	1273	0	86.34%
CnigOR45	MN005014	3′	1275	424	4	odorant receptor 31	Schistocerca gregaria	KY964948.1	887	0	88.90%
CnigOR46	MN005015	Y	1275	424	4	odorant receptor 88	Locusta migratoria	KP843346.1	479	1.00E-130	79.40%
CnigOR47	MN005016	5′	1140	379	5	odorant receptor 102	Locusta migratoria	KP843271.1	1003	0	82.94%
CnigOR48	MN005017	3′	1203	400	4	olfactory receptor OR34	Oedaleus asiaticus	MH196306.1	870	0.00E+ 00	89.97%
CnigOR49	MN005018	3′	1389	462	1	odorant receptor 96	Locusta migratoria	KP843235.1	1155	0	83.61%
CnigOR50	MN005019	3′	1125	374	5	odorant receptor 103	Schistocerca gregaria	KY965020.1	320	7.00E-83	80.14%
CnigOR51	MN005020	Y	1248	415	7	odorant receptor 36	Locusta migratoria	KP843255.1	1086	0	85.62%
CnigOR52	MN005021	3′	333	110	0	odorant receptor 112	Schistocerca gregaria	KY965029.1	300	3.00E-77	83.86%
CnigOR53	MN005022	Y	1245	414	6	odorant receptor 28	Locusta migratoria	KP843306.1	1197	0	90.04%
CnigOR54	MN005023	M	1296	431	5	odorant receptor 23	Locusta migratoria	KP843323.1	1120	0	85.07%
CnigOR55	MN005024	3′	246	81	0	odorant receptor 77	Locusta migratoria	KP843362.1	134	2.00E-27	90.91%
CnigOR56	MN005025	M	906	301	4	odorant receptor 120	Locusta migratoria	KP843236.1	754	0.00E+ 00	83.27%
CnigOR57	MN005026	5′	1200	400	6	odorant receptor 5	Locusta migratoria	KF601291.1	257	6.00E-64	75.13%
CnigOR58	MN005027	3′	1410	469	7	odorant receptor 29	Schistocerca gregaria	KY964946.1	1589	0	88.53%
CnigOR59	MN005028	Y	1374	457	7	odorant receptor 1	Locusta migratoria	KP843273.1	1515	0	86.70%
CnigOR60	MN005029	Y	1329	442	4	odorant receptor 105	Locusta migratoria	KP843270.1	1022	0	80.62%
CnigOR61	MN005030	3′	1389	462	5	olfactory receptor OR35	Oedaleus asiaticus	MH196307.1	599	1.00E-166	84.76%
CnigOR62	MN005031	Y	1230	409	5	odorant receptor 46	Schistocerca gregaria	KY964963.1	846	0	80.84%
CnigOR63	MN005032	Y	1242	413	6	odorant receptor 34	Locusta migratoria	KP843363.1	1003	0	81.56%
CnigOR64	MN005033	Y	1230	409	6	odorant receptor 51	Locusta migratoria	KP843350.1	1308	0	85.99%
CnigOR65	MN005034	3′	1059	352	2	odorant receptor 44	Schistocerca gregaria	KY964961.1	1120	0	85.82%
CnigOR66	MN005035	3′	1413	470	0	odorant receptor 107	Locusta migratoria	KP843267.1	1319	0	84.51%
CnigOR67	MN005036	3′	1239	412	3	odorant receptor 58	Locusta migratoria	KP843325.1	1223	0	84.60%
CnigOR68	MN005037	3′	405	134	0	odorant receptor 44	Locusta migratoria	KP843275.1	510	1.00E-140	89.38%
CnigOR69	MN005038	M	732	243	4	odorant receptor 17	Locusta migratoria	KP843324.1	320	5.00E-83	76.85%
CnigOR70	MN005039	Y	1347	448	4	odorant receptor 105	Locusta migratoria	KP843270.1	737	0	76.83%
CnigORco	MN005040	Y	1458	485	7	olfactory receptor coreceptor	Ceracris kiangsu	KU043292.1	2560	0	98.35%

The mark of Y, 5′, 3′, and M means that the fragment of the unigene consists of complete open reading frame, 5′-end containing start codon, 3′-end containing stop codon, and the middle part without start and stop codon, respectively

**Table 4:** Summary of ionotropic receptors (IRs) identified in *C. nigricornis*

Gene name	Accession number	Full length	ORF (bp)	Amino acid length (AA)	Tm domain	Homology match			Score	E-value	Identity (%)
						Name	Species	Accession number			
CnigIR1	MK990725	3′	879	292	2	ionotropic receptor 6	Locusta migratoria	KT279128.1	542	1.00E-149	79.56%
CnigIR2	MK990726	Y	1533	510	3	ionotropic receptor 26	Locusta migratoria	KP843223.1	1435	0	92.99%
CnigIR3	MK990727	Y	2322	773	4	ionotropic receptor 21	Locusta migratoria	KP843211.1	1256	0	91.18%
CnigIR4	MK990729	3′	2034	677	1	ionotropic receptor 29	Locusta migratoria	KT279132.1	1690	0	91.89%
CnigIR5	MK990730	3′	960	319	0	ionotropic receptor 14	Locusta migratoria	KT279126.1	324	5.00E-84	85.71%
CnigIR8a	MK990728	Y	2691	896	3	ionotropic receptor 8a	Locusta migratoria	KR349063.1	3777	0	91.85%
CnigIR25a	MK990731	Y	2715	904	4	ionotropic receptor IR25a	Oedaleus asiaticus	MH196264.1	4052	0	93.86%
CnigIR76b	MK990732	Y	1608	535	4	ionotropic receptor 76b	Locusta migratoria	KP843210.1	2233	0	91.73%

The mark of Y, 5′, 3′, and M means that the fragment of the unigene consists of complete open reading frame, 5′-end containing start codon, 3′-end containing stop codon, and the middle part without start and stop codon, respectively

**Table 5:** Summary of sensory neuron membrane proteins (SNMPs) identified in *C. nigricornis*

Gene name	Accession number	Full length	ORF (bp)	Amino acid length (AA)	Tm domain	Homology match			Score	E-value	Identity (%)
						Name	Species	Accession number			
CnigSNMP1	MK976705	Y	1599	532	2	sensory neuron membrane protein 1	Schistocerca gregaria	AMS24657.1	631	0	70.93%
CnigSNMP2	MK976706	5′	876	291	1	sensory neuron membrane protein 2	Schistocerca gregaria	AMS24658.1	433	2.00E-146	78.77%
CnigSNMP2a	MK976707	3′	663	220	1	sensory neurone membrane protein SNMP2a	Oedaleus asiaticus	QAB43878.1	398	5.00E-135	90.00%

The mark of Y, 5′, 3′, and M means that the fragment of the unigene consists of complete open reading frame, 5′-end containing start codon, 3′-end containing stop codon, and the middle part without start and stop codon, respectively

### Homology relationship of olfactory-related genes

To reveal the homology relationships of all olfactory related genes of *C. nigricornis* with other insect gene sets, we conducted phylogenetic analyses based on the amino acid sequences of 121 OBPs from nine species (Additional file 2: Figure S5), 87 CSPs from seven species (Additional file 2: Figure S6), 293 ORs from three species (Additional file 2: Figure S7), 115 IRs from five species (Additional file 2: Figure S8), and 24 SNMPs from nine species (Additional file 2: Figure S9), respectively. All members of CSPs (Additional file 2: Figure S6), IRs (Additional file 2: Figure S8), and SNMPs (Additional file 2: Figure S9) show orthologous relationships with the counterparts from other orthopteran species. For OBPs, CnigOBP6 and CnigOBP8 are paralogous and may arouse by a recent gene duplication event; the remaining 18 CnigOBPs have orthologous relationships with the other orthopteran species (Additional file 2: Figure S5). For 71 CnigORs, 61 show orthologous relationships with orthopteran species, the other 10 CnigORs (CnigOR5/48, CnigOR17/18, CnigOR19/20, CnigOR35/36, CnigOR40/41) show 2:1 orthologous relationships with the orthopteran species, indicating in-paralogous or out-paralogous relationships among these CnigORs pairs.

### Tissue-specific expression analyses by RNA-Seq

To fully understand the differential expression patterns of olfactory-related genes in different tissues, the Illumina reads of each RNA sample were mapped to the reference transcripts to determine the expression quantity. The average number of mapped reads was 75.26% (Additional file 1: Table S1). Fragments per kilobase per million mapped fragments (FPKM) values [49] were determined to measure the gene expression levels.

**Fig. 1:**
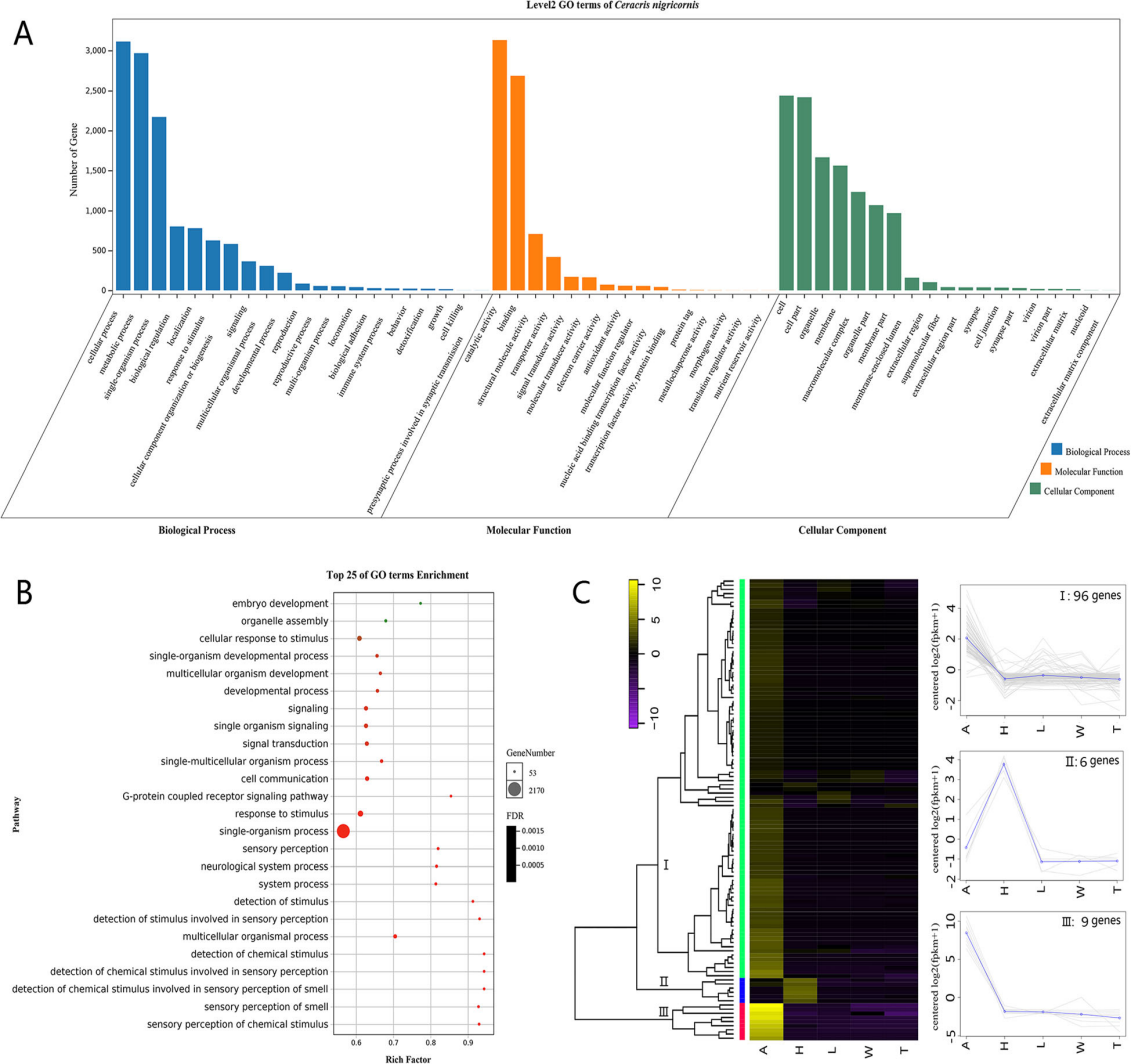
Differentially expressed genes (DEGs) analysis of *C. nigricornis*. **a** Gene ontology (GO) classifications of DEGs in *C. nigricornis*. **b** GO enrichment analysis in the top 25 DEGs of biological process category. **c** K-means clustering of 111 olfactory-related DEGs. A: antennae; H: head (antennae were cut off); L: leg; W: wing; T: abdomen-thorax. Log10 (FPKM+ 1) values were used, and FPKM values were the average values of each tissues, including female three biological repeats and male three biological repeats

To detect olfactory-related differentially expressed genes (DEGs) of different tissues, we considered four combinations for comparison: antennae vs. head (A vs H), antennae vs. abdomen-thorax (A vs T), antennae vs. leg (A vs L) and antennae vs. wing (A vs W). A total of 21,484 transcripts were DEGs, and 6357 of them were assigned to GO terms. Among the 6357 DEGs, we found that cellular process, metabolic process and single-organism process represented a high percentage of the biological process category, catalytic activity and binding represented the majority of the molecular function category, cell and cell part represented the greatest proportion of the cellular component category (Fig. 1a). In the biological process category, significantly enriched GO terms were mainly associated with chemosensory perception, such as sensory perception of chemical stimulus, sensory perception of smell, detection of chemical stimulus involved in sensory perception of smell, detection of stimulus involved in sensory perception and sensory perception (Fig. 1b). This expression pattern suggests that chemosensory perception is differentially expressed in different tissues. To better understand these differences, we manually inspected the transcription of genes encoding binding proteins (OBPs and CSPs) and chemosensory membrane proteins (ORs, IRs and SNMPs) to find DEGs in different tissues. The hierarchical cluster analysis showed that all differentially expressed olfactory-related genes were clustered into three clusters (Fig. 1c). In Cluster I and Cluster III, a total of 105 genes had the highest expression in antennae, but the log2FPKM values of Cluster III were higher than those of Cluster I. In Cluster II, the expression detected in the head was higher than that detected in the antennae, legs, wings and abdomen-thorax.

**Fig. 2:**
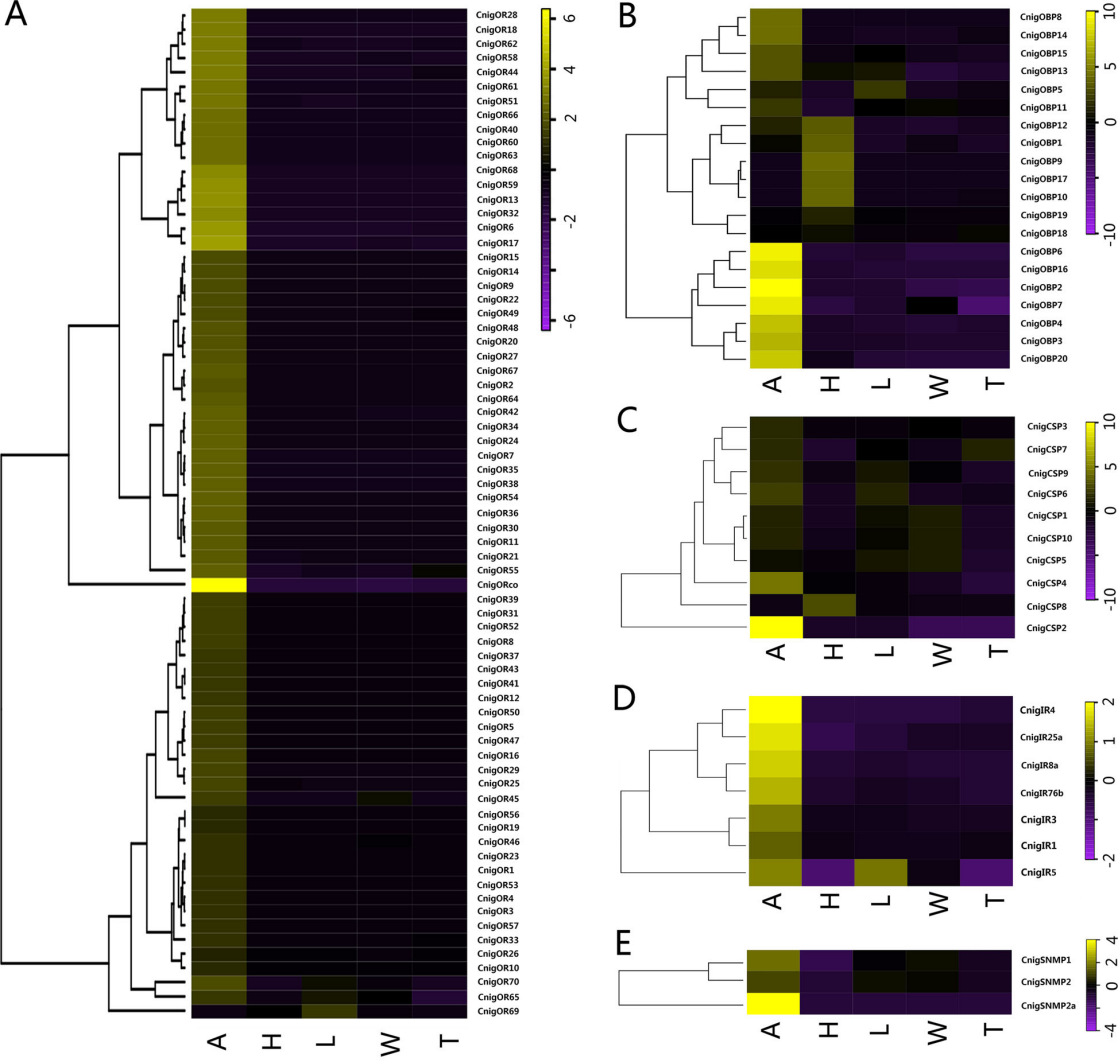
Hierarchical clustering of differentially expressed olfactory-related genes with difference tissues in *C. nigricornis*. **a** odorant receptors (ORs); **b** odorant binding proteins (OBPs); **c** chemosensory proteins (CSPs); **d** ionotropic receptors (IRs); **e** sensory neuron membrane proteins (SNMPs). A: antennae; H: head (antennae were cut off); L: leg; W: wing; T: abdomen-thorax. Log10 (FPKM+ 1) values were used, and FPKM values were the average values of each tissues including female three biological repeats and male three biological repeats

To clearly describe the DEGs of each olfactory-related gene, we performed cluster analysis on their DEGs and represented in heatmap (Fig. 2). Based on the expression levels in different tissues, most of the differentially expressed OBP genes were highly expressed in the antennae and head tissues. Among them, 12 candidate OBPs (CnigOBP2/3/4/6/7/8/11/13/14/15/16/20) showed antennae-specific expression, and seven candidate OBPs (CnigOBP1/9/10/12/17/18/19) showed head-specific expression (Fig. 2b). The expression analysis showed that except for CnigCSP5 and CnigCSP8, all of the differentially expressed CSP genes were highly expressed in the antennae. Among those genes highly expressed in the antennae, CnigCSP6 and CnigCSP9 were also highly expressed in the legs, CnigCSP1 and CnigCSP10 were also highly expressed in the wings, and CnigCSP7 was also highly expressed in the abdomen-thorax. CnigCSP5 was relatively highly expressed in the legs and wings, and CnigCSP8 was highly expressed in the head (Fig. 2c). Except for CnigOR69 that showed relatively high expression levels in the legs, all differentially expressed ORs showed antennal-specific or antennal-biased expression (Fig. 2a). The IRs showed a similar expression profile to the ORs, but more IRs than ORs were detected in the legs and wings (Fig. 2d). All SNMPs were highly expressed in the antennae (Fig. 2e).

### Sex- and tissue-specific expression analyses by qRT-PCR

**Fig. 3:**
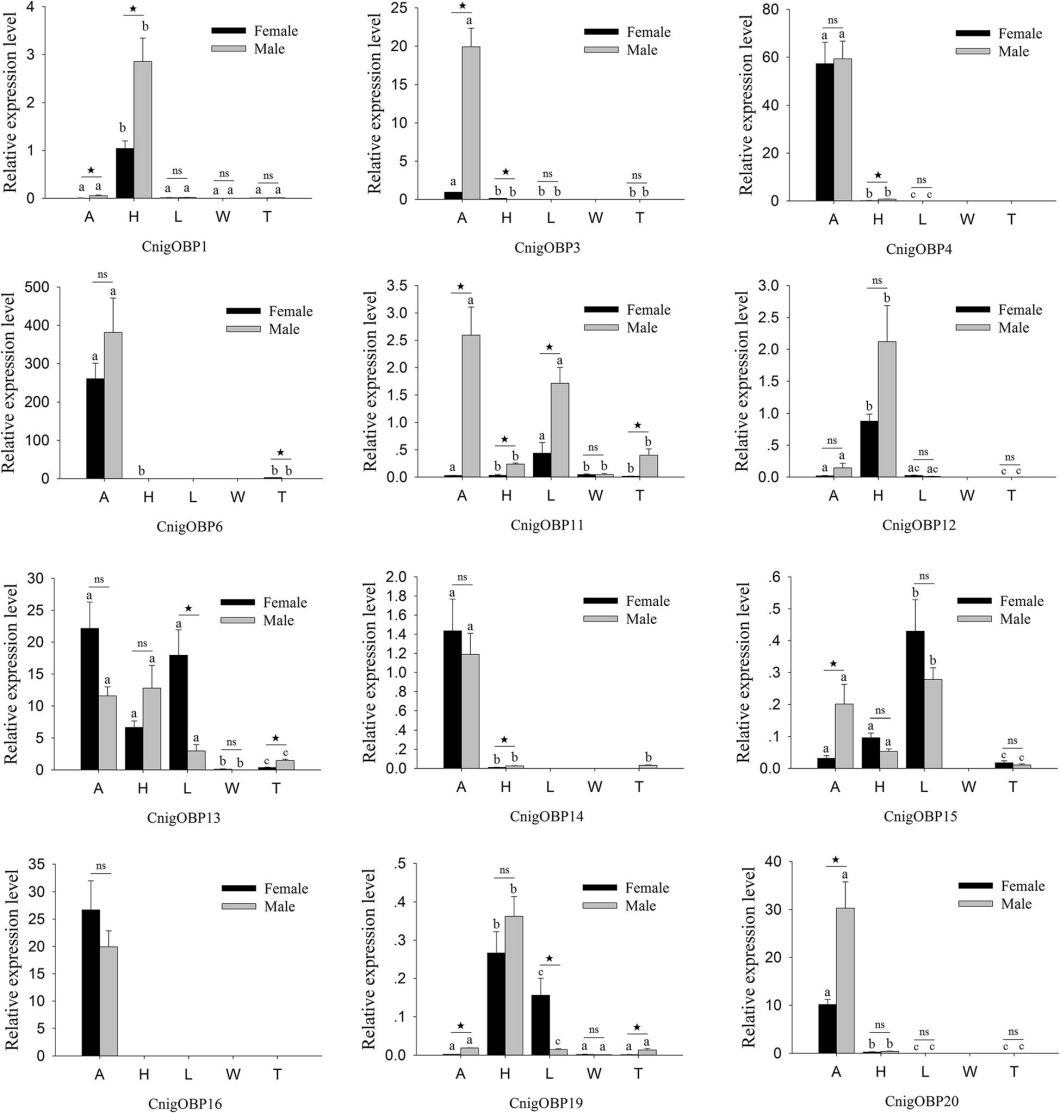
Quantitative real-time PCR analysis of relative expression levels of odorant binding proteins (OBPs) from *C. nigricornis*. A: antennae; H: head (antennae were cut off); L: leg; W: wing; T: abdomen-thorax. The β-actin was used as the reference gene and CnigOBP3 in female antennae as a positive control. The error bars represent the standard error of three independent experiments. Different small letters above bars indicate significant differences among different tissues (*P* < 0.05). * indicates significant difference between both sexes in the same tissue (*P* < 0.05), and ns indicates no significant difference

To explore the tissue distribution expression pattern of olfactory-related genes in female and male adults of *C. nigricornis* and to test the RNA-Seq results, we investigated the expression patterns of 12 OBPs, all CSPs and 12 ORs with qRT-PCR analyses. The results showed that the expression levels of the tested genes in different tissues were mostly consistent with the results of RNA-Seq. Based on the results of qRT-PCR assays, six OBP genes (CnigOBP3/4/6/14/16/20) had significantly increased expressed in the antennae, and CnigOBP3 and CnigOBP20 were predominantly expressed in the male antennae (*P* < 0.05) (Fig. 3). CnigOBP1, CnigOBP12 and CnigOBP19 were predominantly expressed in the head, and CnigOBP1 was significantly more highly expressed in the male head than in the female head among the three head-biased OBPs (Fig. 3). In addition, CnigOBP15 was highly expressed in the legs, CnigOBP11 was highly expressed in the antennae and legs, CnigOBP13 was highly expressed in three tissues (antennae, head and leg), and all 12 OBPs had no or little expression in the wings and abdomen-thorax (Fig. 3).

**Fig. 4:**
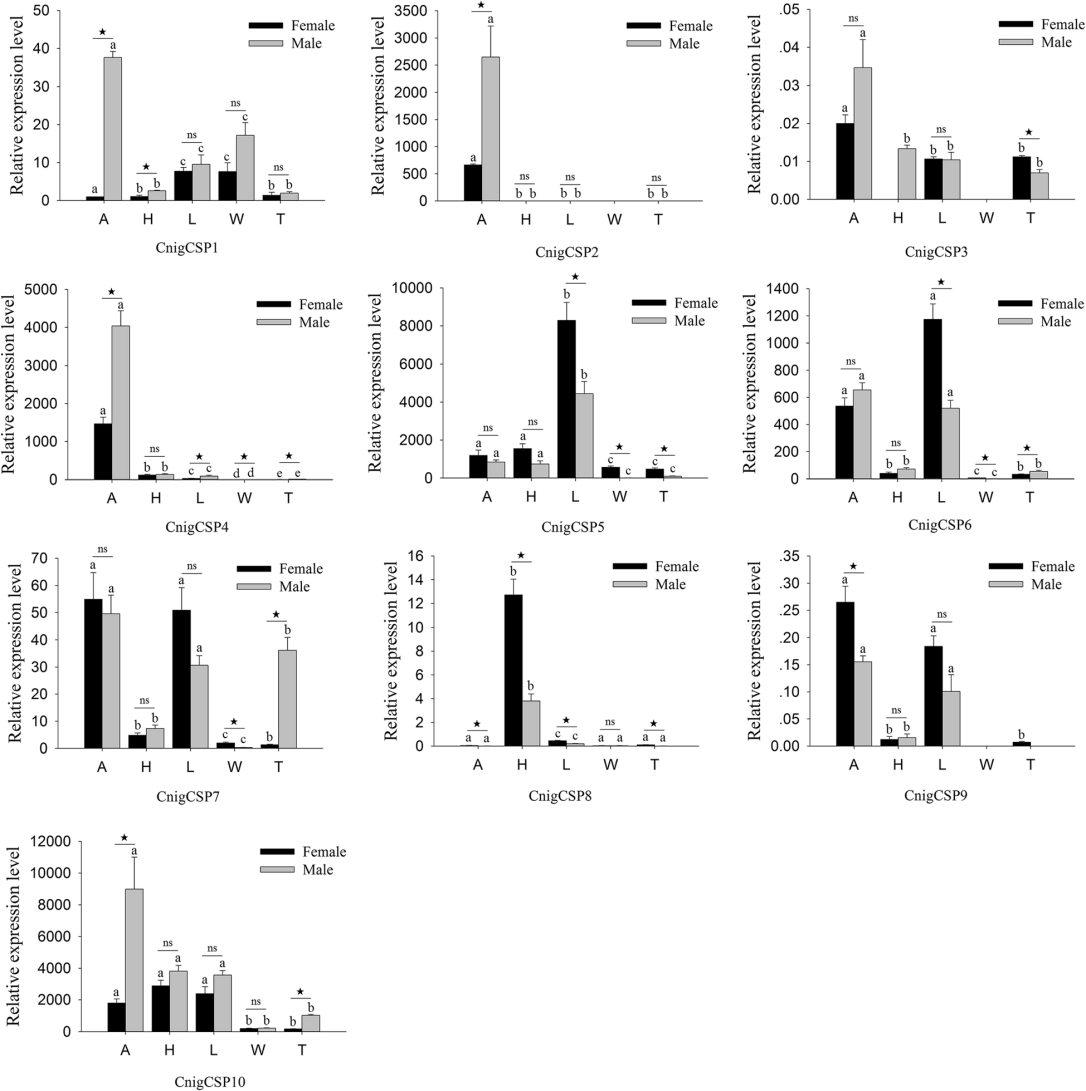
Quantitative real-time PCR analysis of relative expression levels of chemosensory proteins (CSPs) from *C. nigricornis*. A: antennae; H: head (antennae were cut off); L: leg; W: wing; T: abdomen-thorax. The β-actin was used as the reference gene and CnigCSP1 in female antennae as a positive control. The error bars represent the standard error of three independent experiments. Different small letters above bars indicate significant differences among different tissues (*P* < 0.05). * indicates significant difference between both sexes in the same tissue (*P* < 0.05), and ns indicates no significant difference

The qRT-PCR results revealed that all 10 CSPs had significant differences in the expression levels among different tissues. Four CSP genes (CnigCSP1/2/3/4) were expressed more highly in the antennae than in other tissues (*P* < 0.05), and except for CnigCSP3, all antennae-biased CSPs were significantly more highly expressed in the male antennae than in the female antennae (Fig. 4). CnigCSP8 was predominantly expressed in the head, while CnigCSP5 was highly expressed in the legs. Meanwhile, there were significant differences in the expression levels of CnigCSP8 in the head and CnigCSP5 in the legs between males and females, and CnigCSP8 and CnigCSP5 were highly expressed in the female head and leg tissues, respectively (Fig. 4). The remaining four CSPs (CnigCSP6/7/9/10) were highly expressed in more than two tissues; among them, CnigCSP6, CnigCSP7 and CnigCSP9 were more highly expressed in the antennae and legs than in other tissues, and CnigCSP10 was more highly expressed in the antennae, head and legs than in the wings and abdomen-thorax (Fig. 4).

**Fig. 5:**
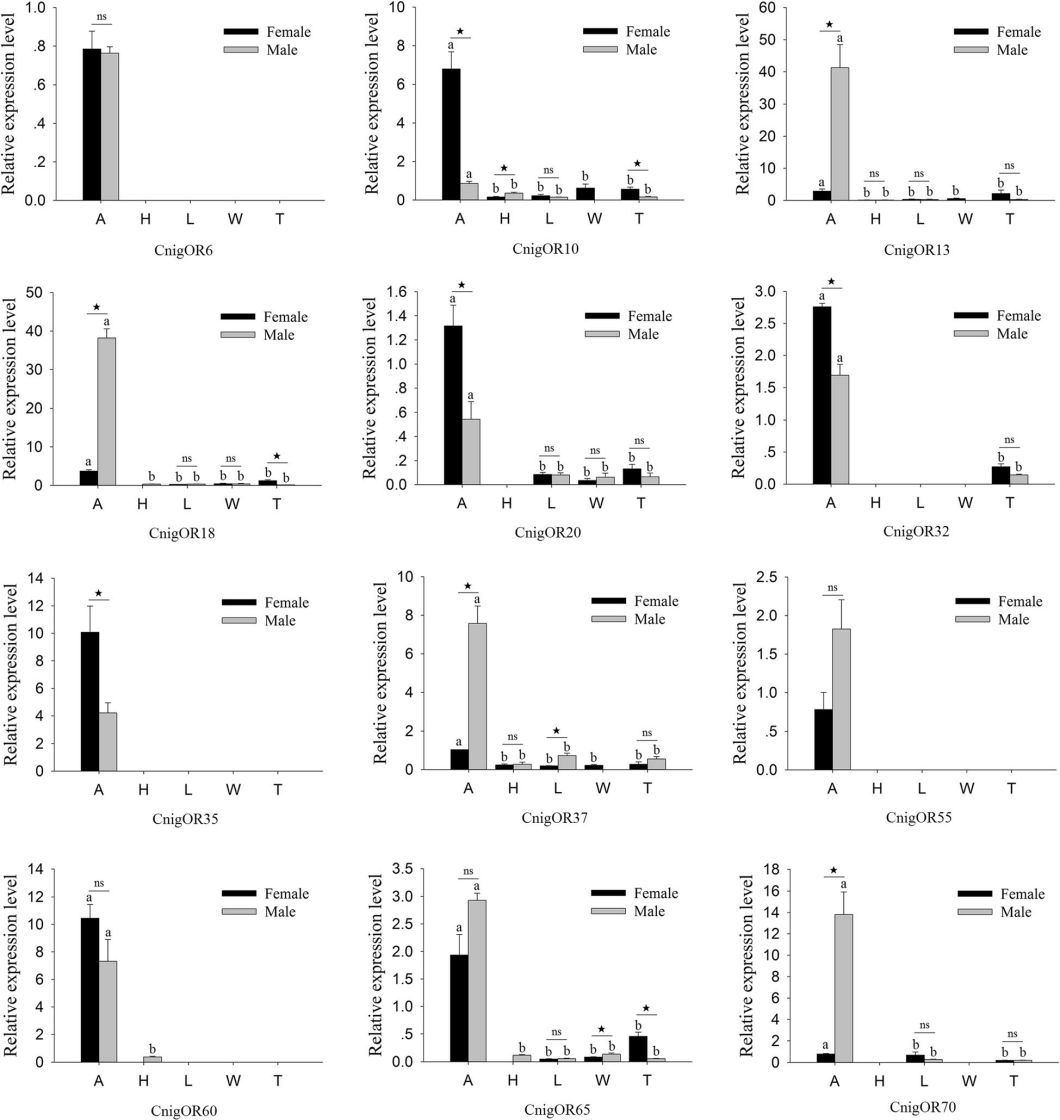
Quantitative real-time PCR analysis of relative expression levels of odorant receptors (ORs) from *C. nigricornis*. A: antennae; H: head (antennae were cut off); L: leg; W: wing; T: abdomen-thorax. The β-actin was used as the reference gene and CnigOR37 in female antennae as a positive control. The error bars represent the standard error of three independent experiments. Different small letters above bars indicate significant differences among different tissues (*P* < 0.05). * indicates significant difference between both sexes in the same tissue (*P* < 0.05), and ns indicates no significant difference

All randomly selected OR genes were strongly expressed in the antennae, whereas they were not or were more faintly expressed in other tissues (Fig. 5). Among the 12 ORs, four CnigOR genes (CnigOR13/18/R37/70) were more highly expressed in the male antennae than in the female antennae, four CnigOR genes (CnigOR10/20/32/35) were more highly expressed in the female antennae than in the male antennae (*P* < 0.05), and the remaining four CnigOR genes (CnigOR6/55/60/65) were not significantly different between the male and female antennae (Fig. 5).

## Discussion

Orthoptera insects have an exclusive evolutionary trend in their olfactory system, which is an excellent model for studying the olfactory evolution of insects [50]. However, few investigations have focused on the molecular basis of olfaction in these species. In this study, we sequenced and analyzed the transcriptomes of *C. nigricornis* from the antennae, head (antennae were cut off), legs, wings and abdomen-thorax, and the results of this study further enriched the molecular biological foundation of *C. nigricornis*. Furthermore, we also identified 112 candidate olfactory-related genes in the transcriptomes of *C. nigricornis* for the first time, including 20 OBPs, 10 CSPs, 71 ORs, eight IRs and three SNMPs. The numbers of candidate olfactory-related genes identified in the transcriptomes of *C. nigricornis* were similar to the numbers of candidate olfactory-related genes identified in the transcriptomes of *O. asiaticus* (15 OBPs, 60 ORs, 6 IRs and 3 SNMPs) [42, 51], but less than that identified in the genome of *L. migratoria* (22 OBPs, 142 ORs and 32 IRs) [35, 52]. One possible reason for this finding is that the olfactory-related genes in *C. nigricornis* and *O. asiaticus* were identified from transcriptome data, and these genes are usually expressed at low levels in transcriptome studies. One limiting factor in transcriptome sequencing is the inaccurate detection of genes with low transcriptional abundance, which may lead to the deletion of genes with relatively low expression levels [53]. However, the sequencing method or depth used in the genomic data of *L. migratoria* may have allowed the detection of genes with lower expression levels.

It has been demonstrated that OBPs increase the sensitivity of odor for insects and mediate the recognition and discrimination of odor compounds [54]. In this study, we identified 20 OBPs in *C. nigricornis*, differing from 22, 18, 15 and 14 OBPs identified in *L. migratoria* [52], *O. infernalis* [43], *O. asiaticus* [42] and *S. gregaria* [55], respectively. This may reflect that different insects evolved different physiological behaviors in the process of adapting to various environments, which might lead to divergent evolutionary trajectories of olfactory genes of the same ancestry, resulting in different functional genes among species [18]. Orthopteran insects possess a significantly smaller expansion of the OBP family than Dipteran insects, such as *D. melanogaster* [56] containing 51 OBPs and *Aedes aegypti* [57] containing 66 OBPs. However, this reduced number of OBPs seems to be balanced by the expansion of the CSP family, of which a large number of CSPs (70) were reported in the oriental locust *L. migratoria* [58]. This finding indicates the specific physiological and evolutionary level of the Orthopteran olfactory system.

Based on the RNA-Seq and qRT-PCR results, most OBPs identified in *C. nigricornis* were higher expressed in antennae than in other tissues, indicating that these OBPs might play roles in the recognition of sex pheromones and host volatile compounds, as in other insect species (Figs. 2b and 3) [59, 60]. Moreover, the expression profiles of most OBPs in the antennae showed male-biased expression, suggesting their possible crucial roles in detecting female pheromone and mating behavior (Fig. 3) [19]. However, we also found that several OBPs were more highly expressed in the head and legs, suggesting that these OBPs might participate in other physiological functions [43]. Among them, CnigOBP1, CnigOBP12 and CnigOBP19 were much more highly expressed in head tissues than in other tissues. The head tissues used in this study still contained other chemosensory organs, such as labium, maxillary palp, labial palp and proboscis, even though their antennae were cut off. Previous studies have suggested that the OBPs expressed in mouthparts and proboscis are associated with gustatory responses [61, 62]. This indicated that CnigOBP1, CnigOBP12 and CnigOBP19 might play important roles in taste functions.

CSPs have similar functions to OBPs in chemical communication in insects, as they bind small molecules, such as pheromones and odorants, and transport them to chemoreceptors [21]. However, CSPs usually exhibit much broader expression profiles than OBPs both in olfactory tissues and nonolfactory tissues and play multiple important roles in biological processes such as ecdysis, leg regeneration and embryo maturation [15, 25, 63]. An interesting example of the diverse roles of CSPs was observed in the locust *L. migratoria*, in which several CSPs showed increased expression in gregarious locusts, but these CSPs displayed contrasting expression trends when the locusts underwent the physiological transformation from the ‘gregarious’ phase to the ‘solitary’ phase, which indicated that CSPs are one factor triggering locust phase shift [23]. Our results show that CnigCSP1, CnigCSP2, CnigCSP3 and CnigCSP4 were antennae-enriched, and these four CSPs might be involved in the chemosensory process (Fig. 4). In addition, in nonolfactory tissues, the expression profiles of *C. nigricornis* CSPs were much broader than those of *C. nigricornis* OBPs (Figs. 3 and 4). CnigCSP6/7/9 showed higher expression in both antennae and legs, CnigCSP5 was mainly expressed in the legs, CnigCSP8 was predominantly expressed in the head and CnigCSP10 was highly expressed in the antennae, head and legs. We speculate that the broad and diverse expression patterns of *C. nigricornis* CSPs might have other crucial physiological functions, which need further functional verification.

ORs are usually expressed in the dendrites of antennal sensilla and act as biotransducers to convert chemical signals of odorant molecules into electrical signals [64]. To transmit odor-evoked signals, two types of ORs are required: one is a highly conserved ORco, and the other is a specific OR, which varies according to ORN type [65, 66]. In this study, we identified 70 specific ORs and a conserved ORco, and the RNA-Seq results showed that all ORs were highly expressed in the antennae, except CnigOR69, which were relatively highly expressed in nonolfactory leg tissues (Fig. 2a). The antennae-biased ORs may play an important role in odorant reception in antennae or be involved in the olfactory sensing process [67], and the nonolfactory tissues highly expressed ORs, which suggests that they may participate in other physiological processes in addition to olfaction [53]. Moreover, we also investigated the distribution of OR expression patterns in female and male tissues with qRT-PCR analyses. The expression profiles showed that all 12 randomly selected ORs were strongly expressed in the antennae, which was consistent with the results of RNA-Seq. Among the 12 ORs, four CnigOR genes (CnigOR13/18/R37/70) showed male-biased expression, suggesting that they may play a role in female pheromone detection and mating behavior, whereas four CnigOR genes (CnigOR10/20/32/35) displayed female-biased expression predicted to function in oviposition-related odorant detection [68, 69] (Fig. 5).

IRs, a new subfamily of ORs, are involved in not only olfaction but also gustation, hygrosensation and thermosensation, which are widely distributed throughout the body, including the labellum, leg, pharynx, and wing sensilla [70–74]. We identified eight IRs in *C. nigricornis*, of which CnigIR2 were not differentially expressed in different tissues, suggesting that this IR may have multiple functions. Except for CnigIR5, all differentially expressed IRs were strongly expressed in antennae (Fig. 2d). Recent studies have shown that antennae-enriched IRs play important roles in odor and thermosensation [75], the antennae-enriched IRs of *C. nigricornis* that were identified in our study may have similar roles.

We also identified three SNMPs, and the FPKM-value showed that all SNMPs were strongly expressed in antennae, indicating that they may be involved in the process of olfaction. However, CnigSNMP1 and CnigSNMP2 were also broadly expressed in nonolfactory tissues, including head, leg, wing and abdomen-thorax tissues (Fig. 2e). Similar patterns were also observed in other studies of *D. melanogaster*, *A. aegypti*, *Spodoptera litura* and *Cnaphalocrocis medinalis* [76–80]. The broad expression patterns of SNMP1 and SNMP2 imply that they may not only function in odorant perception, but also have different functions specific to the various tissues [78]. The exact role for CnigSNMP1 and CnigSNMP2 in olfactory and nonolfactory tissues remains inconclusive and requires further investigation.

## Conclusions

The 112 candidate olfactory-related genes that we identified from *C. nigricornis* compose the comprehensive list of olfactory-related genes in this bamboo pest of *C. nigricornis*. Sex- and tissue-specific expression profiling revealed that most of the candidate olfactory-related genes were antennae-enriched, but some were nonantennae-enriched, and some were sex-biased, indicating their different roles in the olfactory system of *C. nigricornis*. Our results provide a foundation to facilitate functional studies of these olfactory-related genes in *C. nigricornis* at the molecular level.

## Methods

### Species and tissues collection

All adult male and adult female specimens of *C. nigricornis* were collected in Xi’an, China, in August 2018. Different tissues, including antennae, head (antennae were cut off), abdomen-thorax, legs and wings, were dissected from female and male specimens, respectively. Each type of tissue sample was collected from nine individuals as an independent biological replicate for transcriptome sequencing. To minimize biological variance, three independent biological replicates were performed for each tissue sample. All tissue samples were immediately placed into enzyme-free centrifuge tubes, which were immersed in liquid nitrogen and stored at − 80 °C until further use.

### RNA extraction, library construction and Illumina sequencing

Total RNA was extracted from each frozen tissue using TRIzol reagent (Invitrogen, Carlsbad, CA, USA) following the protocol recommended by the manufacturer. RNA integrity and purity were determined with an Agilent Bioanalyzer 2100 (Agilent Technologies, CA, USA) and a Nano Drop 2000 (Thermo Scientific, Wilmington, DE, USA). The cDNA library construction and Illumina sequencing were carried out by the Biomarker Technology Company, Beijing, China. First, qualified RNA from each tissue was mixed in equal amounts. Then, the cDNA library was prepared using the NEBNext Ultra™ RNA Library Prep Kit for Illumina (NEB, USA), and the Agilent Bioanalyzer 2100 system was used to assess the library. Finally, the amplified mRNA library was sequenced on the Illumina HiSeq X Ten platform to generate 150 bp paired-end reads.

### De novo assembly and functional annotation

Prior to assembly, low-quality reads and adaptor reads were removed from raw data through in-house Perl scripts to obtain clean reads. Then, the clean reads were de novo assembled using Trinity v2.4.0 [81] with default parameters except that ‘min_kmer_cov’ set to 2. Next, cd-hit-est v4.6.1 software [82] was used to remove duplicate and highly similar sequences to obtain nonredundant unigenes. Furthermore, the completeness of the nonredundant unigenes was assessed using BUSCO v3.0.2 software [48], and the insect lineage database (insecta_orthoDB9, created 13/02/2016) was used as a proxy for the minimum completeness assessment library.

BLAST v2.2.23 software [83] was used to search and annotate all assembled unigenes with the publicly available protein databases, including NR, Swiss-Prot, KEGG, KOG, COG and EggNOG, and an E-value cut-off of 1.0E-05. KOBAS2.0 was used to obtain KEGG Orthology results of unigenes in the KEGG annotation. Blast2GO [84] was used for GO annotation based on the protein annotation results of the NR database. For the GO classifications, the default parameters were used (E-value <1E-5, an annotation cut-off > 5 and a GO weight > 5). After the amino acid sequences of the unigenes were obtained, HMMER [85] software (E-value ≤1E-10) was used to BLAST search against the Pfam database to obtain the annotation information of unigenes.

### Identification of olfactory-related genes

Olfactory-related genes from the transcriptomes of *C. nigricornis* were identified by the functional annotation results. To obtain more information, a tBLASTn program was also performed using available olfactory proteins from Orthoptera species as queries to identify candidate unigenes. All candidate olfactory genes were manually confirmed using the BLASTx program against the NR database at NCBI (https://blast.ncbi.nlm.nih.gov/Blast.cgi). The ORFs of candidate ORs, IRs, SNMPs, OBPs and CSPs were predicted using ORF Finder (https://www.ncbi.nlm.nih.gov/orffinder/). The N-terminal signal peptides and conserved domains of candidate OBPs and CSPs were predicted by the SignalP V 4.1 program (http://www.cbs.dtu.dk/services/SignalP/) [86] and SMART (simple modular architecture research tool, http://smart.embl.de/) [87], respectively. TMHMM Server Version 2.0 (http://www.cbs.dtu.dk/services/TMHMM/) was used to predict the TMDs of candidate ORs, IRs and SNMPs.

### Phylogenetic analysis

Phylogenetic analysis was performed based on the amino acid sequences of *C. nigricornis* olfactory-related genes using MEGA7.0 software [88]. The maximum likelihood method with Jones-Taylor-Thornton (JTT) model was used to create the phylogenetic trees. Branch support was assessed by bootstrapping with 1000 replicates. All the amino acid sequences used to construct the phylogenetic tree are shown in Additional file 3.

### Differential expression analysis

Using the assembled transcriptome as reference sequences, the clean data from various samples of *C. nigricornis* were mapped back onto the reference sequences using Bowtie2 v2.1.0 software [89]. The unigene expression levels among various samples were estimated by RSEM [90] according to the readcount values of the unigenes for each sample, which were obtained from the mapping results. FPKM values can eliminate the effects of the sequencing depth and transcript length on readcounts. To make the unigene expression levels of various samples comparable, all the readcounts were transformed into FPKM values.

To detect the differential expression of candidate olfactory-related genes among different tissues from transcriptomes of *C. nigricornis*, we used the antennae as the experimental tissue, and four other tissues (head, abdomen-thorax, leg, and wing) were used as control samples. Therefore, a total of four combinations were compared, including antennae vs. head, antennae vs. abdomen-thorax, antennae vs. leg and antennae vs. wing. The differential expression analysis of each comparison combination was analyzed by the DESeq R package (1.10.1) [91]. The resulting *P* values were adjusted using Benjamini and Hochberg’s approach for controlling the false discovery rate (FDR). In general, the fold change (log2 ratio) and adjusted *P* value (FDR) were used as the key indexes for screening DEGs. In this study, |log2 (Fold Change)| > 2 and adjusted *P* value < 0.05 were used as the thresholds for identifying significant DEGs. The log2-transformed FPKM values of the DEGs were used for hierarchical clustering using the pheatmap R package. GO enrichment analysis of DEGs was implemented by the topGO R package-based Kolmogorov-Smirnov test.

### Expression profile analysis

To explore the tissue distribution expression pattern of olfactory-related genes in female and male adults and to verify the accuracy of transcriptome analysis, qRT-PCR analysis was performed to evaluate the expression profiles of putative olfactory-related genes in different tissues of male and female *C. nigricornis* adults. Total RNA was extracted using TRIzol reagent, and cDNA was synthesized by Goldenstar™ RT6 cDNA Synthesis Mix (TsingKe, Beijing, China) following the protocol recommended by the manufacturer. A total of 12 OBPs, all CSPs and 12 ORs were randomly selected for qRT-PCR analysis. To correct sample-to-sample variation, ß-actin was selected as the reference gene. The specific primers used in the qRT-PCR analysis were designed online (https://www.ncbi.nlm.nih.gov/tools/primer-blast/) (Additional file 1: Table S7). qRT-PCR was performed on the CFX96 Touch Real-Time PCR Detection System (Bio-Rad, USA) using 2 x T5 Fast qPCR Mix (TsingKe, Beijing, China) with the following conditions: initial denaturation at 95 °C for 1 min, followed by 40 cycles of 95 °C for 10 s and 60 °C for 15 s. Finally a melting curve of the PCR products was analyzed to confirm the specificity of the primers. Nontemplate negative control reactions (replacing cDNA with H_2_O) were included in each experiment. Three biological replicates with three technical replicates were conducted for all experiments.

The results were analyzed using the BIO-RAD CFX Manager software. The relative quantification among all tissues was calculated by the comparative 2^-△△CT^ method [92]. A one-way analysis of variance (ANOVA) followed by Duncan’s test were performed for the comparative analyses of each gene among different tissues, and Student’s *t-*test was used to analyze each gene in the same tissue from females and males. Both analyses were performed by SPSS Statistics 18.0 software (SPSS Inc., Chicago, IL, USA).

## Supplementary information

**Additional file 1: Table S1.** The summary of the Illumina sequencing data. Note: FA: female antennae; MA: male antennae; FH: female head (antennae were cut off); MH: male head (antennae were cut off); FL: female leg; ML: male leg; FW: female wing; MW: male wing; FT: female abdomen-thorax; MT: male abdomen-thorax. Representation of three biological repeats with Arabic numbers 1, 2 and 3. **Table S2.** Length distribution and quality metrics of *C. nigricornis* transcripts and unigenes. **Table S3.** BUSCO analyzed the assembly completeness of *C. nigricornis*. **Table S4.** The summary of functional annotation of *C. nigricornis* transcriptomes. **Table S5.** Conserved domains of odorant binding proteins (OBPs) in *C. nigricornis*. **Table S6.** Conserved domains of chemosensory proteins (CSPs) in *C. nigricornis*. **Table S7.** Primers used for qRT-PCR.

**Additional file 2: Figure S1.** Insect species distribution of *C. nigricornis* unigenes’ best-hit annotation term in NR database. **Figure S2.** Gene ontology (GO) classifications of *C. nigricornis* unigenes. **Figure S3.** Alignments of the *C. nigricornis* odorant binding proteins (OBPs). Boxes show the six conserved cysteine residues. **Figure S4.** Alignments of the *C. nigricornis* chemosensory proteins (CSPs). Boxes show the four conserved cysteine residues. **Figure S5.** Phylogenetic tree of odorant-binding proteins (OBPs) from *C. nigricornis* and other insects. *C. kiangsu* (Ckia), *L. migratoria* (Lmig), *O. asiaticus* (Oasi), *O. infernalis* (Oinf), *S. gregaria* (Sgre), *A. glycines* (Agly), *D. ponderosae* (Dpon) and *H. armigera* (Harm). The OBPs of *C. nigricornis* are represented by red font. **Figure S6.** Phylogenetic tree of chemosensory-binding proteins (CSPs) from *C. nigricornis* and other insects. **Table S2.** *L. migratoria* (Lmig), *O. asiaticus* (Oasi), *O. infernalis* (Oinf), *A. gambiae* (Agam), *D. ponderosae* (Dpon) and *H. armigera* (Harm). The CSPs of *C. nigricornis* are represented by red font. **Figure S7.** Phylogenetic tree of odorant receptors (ORs) from *C. nigricornis* and other insects. **Table S3.** *L. migratoria* (Lmig) and *A. lineolatus* (Alin). The ORs of *C. nigricornis* are represented by red font. **Figure S8.** Phylogenetic tree of ionotropic receptors (IRs) from *C. nigricornis* and other insects. *L. migratoria* (Lmig), *O. asiaticus* (Oasi), *A. lineolatus* (Alin) and *D. melanogaster* (Dmel). The IRs of *C. nigricornis* are represented by red font. **Figure S9.** Phylogenetic tree of sensory neuron membrane proteins (SNMPs) from *C. nigricornis* and other insects. *A. lineolatus* (Alin), *A. aegypti* (Aaeg), *A. mellifera* (Amel), *B. mori* (Bmor), *D. melanogaster* (Dmel), *O. asiaticus* (Oasi), *S. gregaria* (Sgre) *and T. castaneum* (Tcas). The SNMPs of *C. nigricornis* are represented by red font.

**Additional file 3: Table S1.** Amino acid sequences of 121 OBPs of *Ceracris nigricornis* and other insect species used to construct phylogenetic tree. **Table S2.** Amino acid sequences of 87 CSPs of *Ceracris nigricornis* and other insect species used to construct phylogenetic tree. **Table S3.** Amino acid sequences of 293 ORs of *Ceracris nigricornis* and other insect species used to construct phylogenetic tree. **Table S4.** Amino acid sequences of 115 IRs of *Ceracris nigricornis* and other insect species used to construct phylogenetic tree. **Table S5.** Amino acid sequences of 24 SNMPs of *Ceracris nigricornis* and other insect species used to construct phylogenetic tree.

## Data Availability

The authors declare that the data supporting the finding of this study are available in the article and its supplementary information files. The olfactory-related genes we identified from *Ceracris nigricornis* were all submitted to the National Center for Biotechnology Information (NCBI) (https://www.ncbi.nlm.nih.gov/) with the accession numbers were listed in Tables 1, 2, 3, 4 and 5. The Transcriptome Shotgun Assembly project has been deposited at DDBJ/EMBL/GenBank under the accession GHNZ00000000. The version described in this paper is the first version, GHNZ01000000. The raw data submitted to the NCBI with the accession numbers of SRR9089653, SRR9089654, SRR9089655, SRR9089656, SRR9089657, SRR9089658, SRR9089659, SRR9089660, SRR9089661, SRR9089662, SRR9089663, SRR9089664, SRR9089665, SRR9089666, SRR9089667, SRR9089668, SRR9089669, SRR9089670, SRR9089671, SRR9089672, SRR9089673, SRR9089674, SRR9089675, SRR9089676, SRR9089677, SRR9089678, SRR9089679, SRR9089680, SRR9089681, SRR9089682.

## Abbreviations

CSPs: Chemosensory binding proteins
DEGs: Differentially expressed genes
FPKM: Fragments per kilobase per million mapped fragments
GO: Gene Ontology
IR: Ionotropic receptor
OBP: Odorant-binding protein
OR: Odorant receptor
ORF: Open reading frame
qRT-PCR: Quantitative realtime polymerase chain reaction
SNMP: Sensory neuron membrane protein
