# Supplementary material for: Sex- and tissue-specific transcriptome analyses and expression profiling of olfactory-related genes in Ceracris nigricornis Walker (Orthoptera: Acrididae)
Source: BMC Genomics. 2019 Nov 6;20:808. doi: 10.1186/s12864-019-6208-x (PMC6836668; doi:10.1186/s12864-019-6208-x)
Supplement: Supplementary file 1 — Additional file 1: Table S1. The summary of the Illumina sequencing data. Note: FA: female antennae; MA: male antennae; FH: female head (antennae were cut off); MH: male head (antennae were cut off); FL: female leg; ML: male leg; FW: female wing; MW: male wing; FT: female abdomen-thorax; MT: male abdomen-thorax. Representation of three biological repeats with Arabic numbers 1, 2 and 3. Table S2. Length distribution and quality metrics of C. nigricornis transcripts and unigenes. Table S3. BUSCO analyzed the assembly completeness of C. nigricornis. Table S4. The summary of functional annotation of C. nigricornis transcriptomes. Table S5. Conserved domains of odorant binding proteins (OBPs) in C. nigricornis. Table S6. Conserved domains of chemosensory proteins (CSPs) in C. nigricornis. Table S7. Primers used for qRT-PCR. [file 12864_2019_6208_MOESM1_ESM.docx]

**Additional file 1:**

Table S1 The summary of the Illumina sequencing data.

| Samples-ID | Clean Read Number | Bases (G) | Clean Read GC Content | Clean Read %≥Q30 | Mapped Reads | Mapped Ratio |
| --- | --- | --- | --- | --- | --- | --- |
| FA1 | 21,118,175 | 6.30 | 47.53% | 95.05% | 14,294,845 | 67.69% |
| FA2 | 24,753,666 | 7.37 | 46.53% | 94.00% | 17,665,015 | 71.36% |
| FA3 | 23,339,236 | 6.94 | 47.10% | 95.23% | 16,151,326 | 69.20% |
| MA1 | 24,383,396 | 7.27 | 46.51% | 94.56% | 16,652,202 | 68.29% |
| MA2 | 26,325,218 | 7.84 | 45.67% | 95.03% | 17,407,765 | 66.13% |
| MA3 | 25,063,733 | 7.48 | 45.65% | 94.57% | 16,242,076 | 64.80% |
| FH1 | 23,660,498 | 7.07 | 52.43% | 95.99% | 20,640,788 | 87.24% |
| FH2 | 25,439,127 | 7.60 | 52.44% | 95.74% | 22,050,801 | 86.68% |
| FH3 | 25,183,824 | 7.52 | 52.03% | 95.43% | 21,269,888 | 84.46% |
| MH1 | 22,530,117 | 6.73 | 49.05% | 94.98% | 17,997,125 | 79.88% |
| MH2 | 25,046,088 | 7.47 | 48.14% | 95.14% | 19,612,927 | 78.31% |
| MH3 | 24,110,430 | 7.20 | 49.03% | 95.28% | 18,998,316 | 78.80% |
| FL1 | 23,144,252 | 6.89 | 48.84% | 95.31% | 17,327,083 | 74.87% |
| FL2 | 23,570,823 | 7.03 | 49.17% | 95.24% | 16,614,447 | 70.49% |
| FL3 | 26,391,135 | 7.88 | 50.60% | 95.60% | 20,304,159 | 76.94% |
| ML1 | 25,358,914 | 7.57 | 48.90% | 95.11% | 17,514,079 | 69.06% |
| ML2 | 21,867,088 | 6.51 | 48.64% | 94.98% | 15,787,688 | 72.20% |
| ML3 | 24,848,629 | 7.41 | 49.53% | 95.06% | 18,861,968 | 75.91% |
| FW1 | 23,890,086 | 7.12 | 49.50% | 94.88% | 18,048,160 | 75.55% |
| FW2 | 27,123,308 | 8.11 | 50.58% | 95.00% | 23,008,613 | 84.83% |
| FW3 | 25,331,447 | 7.56 | 50.33% | 94.91% | 20,801,568 | 82.12% |
| MW1 | 22,431,447 | 6.69 | 48.14% | 94.00% | 15,294,311 | 68.18% |
| MW2 | 21,673,785 | 6.47 | 48.39% | 94.29% | 15,736,910 | 72.61% |
| MW3 | 24,446,829 | 7.30 | 48.02% | 94.27% | 16,025,604 | 65.55% |
| FT1 | 29,291,631 | 8.76 | 52.26% | 94.62% | 23,947,622 | 81.76% |
| FT2 | 29,117,366 | 8.71 | 52.78% | 94.35% | 21,078,012 | 72.39% |
| FT3 | 29,089,098 | 8.71 | 51.03% | 94.67% | 24,275,870 | 83.45% |
| MT1 | 27,791,182 | 8.30 | 49.73% | 95.02% | 20,761,137 | 74.70% |
| MT2 | 29,045,148 | 8.68 | 49.97% | 95.11% | 22,704,182 | 78.17% |
| MT3 | 24,311,213 | 7.26 | 50.06% | 94.75% | 18,554,764 | 76.32% |
| Average | 24,989,230 | 7.46 | 49.29% | 94.94% | 18,854,308 | 75.26% |

Note: FA: female antennae; MA: male antennae; FH: female head (antennae were cut off); MH: male head (antennae were cut off); FL: female leg; ML: male leg; FW: female wing; MW: male wing; FT: female abdomen-thorax; MT: male abdomen-thorax. Representation of three biological repeats with Arabic numbers 1, 2 and 3.

Table S2 Length distribution and quality metrics of the *C. nigricornis* transcripts and unigenes.

| **Length Range** | **Transcript** | **Unigene** |
| --- | --- | --- |
| 300-500 | 14,244(20.18%) | 10,635(24.39%) |
| 500-1000 | 19,190(27.19%) | 12,054(27.64%) |
| 1000-2000 | 18,813(26.65%) | 11,142(25.55%) |
| 2000+ | 18,334(25.98%) | 9,772(22.41%) |
| Total Number | 70,581 | 43,603 |
| Total Length | 112,816,350 | 63,769,326 |
| N50 Length | 2,434 | 2,235 |
| Mean Length | 1598.4 | 1462.5 |

Table S3 BUSCO analysis of assembly completeness in *C. nigricornis*.

| Complete (C) | 89.1% (1477/1658) |
| --- | --- |
| Complete and single-copy (S) | 85.2% (1412/1658) |
| Complete and duplicated (D) | 3.9% (65/1658) |
| Fragmented (F) | 2.5% (41/1658) |
| Missing (M) | 8.4% (140/1658) |

Table S4 The summary of functional annotation of *C. nigricornis* transcriptomes.

| **Anno Database** | **Number** | **Percentage** |
| --- | --- | --- |
| total | 43603 | 100.00% |
| Nr Annotation | 24832 | 56.95% |
| Swissprot Annotation | 15750 | 36.12% |
| KEGG Annotation | 13150 | 30.16% |
| COG Annotation | 11503 | 26.38% |
| KOG Annotation | 18100 | 41.51% |
| EggNOG Annotation | 26933 | 61.77% |
| Pfam Annotation | 22295 | 51.13% |
| GO Annotation | 12102 | 27.75% |
| All Annotated | 29122 | 66.79% |

Table S5 Conserved domains of odorant binding proteins in *C. nigricornis*.

| Proteins | Domains | Domain definition | Position (AA) | E-value | Interpro family | GO function |
| --- | --- | --- | --- | --- | --- | --- |
| CnigOBP1 | 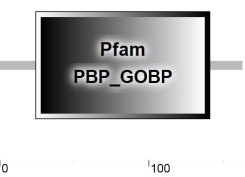 | PBP/GOBP family domain | 24 to 141 | 2.50E-08 | PBP_GOBP (IPR006170) | odorant binding (GO:0005549) |
| CnigOBP2 | 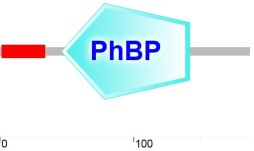 | Insect pheromone/odorant binding protein domains | 46 to 142 | 1.13E-21 | PBP_GOBP (IPR006170) | odorant binding (GO:0005549) |
| CnigOBP3 | 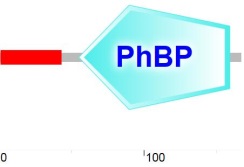 | Insect pheromone/odorant binding protein domains | 58 to 159 | 9.00E-26 | PBP_GOBP (IPR006170) | odorant binding (GO:0005549) |
| CnigOBP4 | 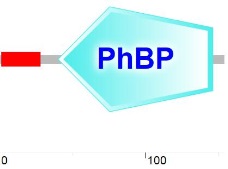 | Insect pheromone/odorant binding protein domains | 43 to 145 | 5.00E-16 | PBP_GOBP (IPR006170) | odorant binding (GO:0005549) |
| CnigOBP5 | 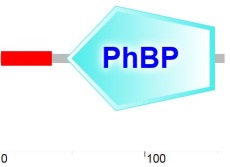 | Insect pheromone/odorant binding protein domains | 46 to 148 | 5.00E-61 | PBP_GOBP (IPR006170) | odorant binding (GO:0005549) |
| CnigOBP6 | 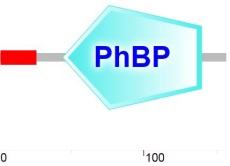 | Insect pheromone/odorant binding protein domains | 44 to 140 | 3.00E-43 | PBP_GOBP (IPR006170) | odorant binding (GO:0005549) |
| CnigOBP7 | 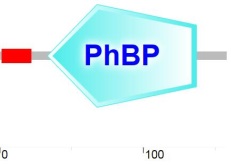 | Insect pheromone/odorant binding protein domains | 33 to 137 | 5.02E-01 | IPR006170 PBP_GOBP (IPR006170) | odorant binding (GO:0005549) |
| CnigOBP8 | 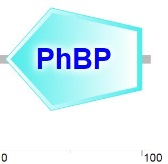 | Insect pheromone/odorant binding protein domains | 4 to 100 | 1.00E-45 | PBP_GOBP (IPR006170) | odorant binding (GO:0005549) |
| CnigOBP9 | 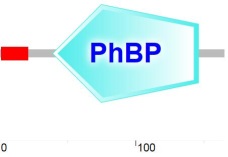 | Insect pheromone/odorant binding protein domains | 52 to 144 | 6.00E-07 | PBP_GOBP (IPR006170) | odorant binding (GO:0005549) |
| CnigOBP10 | 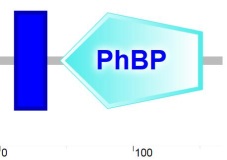 | Insect pheromone/odorant binding protein domains | 49 to 147 | 4.00E-07 | PBP_GOBP (IPR006170) | odorant binding (GO:0005549) |
| CnigOBP11 | 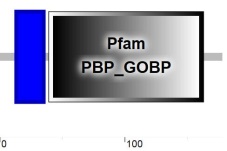 | PBP/GOBP family domain | 39 to 163 | 1.50E-13 | PBP_GOBP (IPR006170) | odorant binding (GO:0005549) |
| CnigOBP12 | 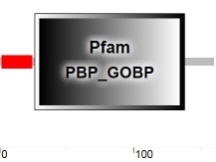 | PBP/GOBP family domain | 26 to 138 | 1.80E-10 | PBP_GOBP (IPR006170) | odorant binding (GO:0005549) |
| CnigOBP13 | 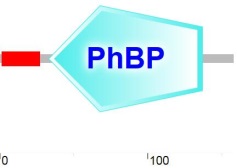 | Insect pheromone/odorant binding protein domains | 33 to 137 | 4.13E-01 | PBP_GOBP (IPR006170) | Odorant binding (GO:0005549) |
| CnigOBP14 | 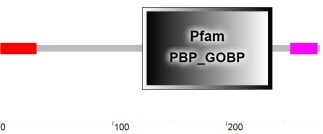 | PBP/GOBP family domain | 126 to 240 | 8.30E-07 | PBP_GOBP (IPR006170) | odorant binding (GO:0005549) |
| CnigOBP15 | 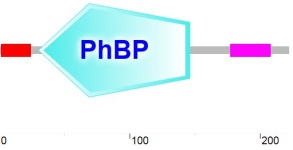 | Insect pheromone/odorant binding protein domains | 30 to 146 | 2.45E-01 | PBP_GOBP (IPR006170) | odorant binding (GO:0005549) |
| CnigOBP16 | 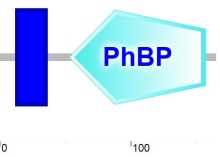 | Insect pheromone/odorant binding protein domains | 53 to 157 | 2.00E-65 | PBP_GOBP (IPR006170) | odorant binding (GO:0005549) |
| CnigOBP17 | 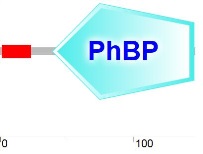 | Insect pheromone/odorant binding protein domains | 42 to 143 | 1.00E-08 | PBP_GOBP (IPR006170) | odorant binding (GO:0005549) |
| CnigOBP18 | 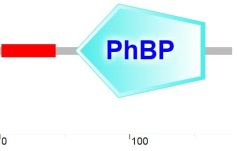 | Insect pheromone/odorant binding protein domains | 58 to 158 | 4.00E-69 | PBP_GOBP (IPR006170) | odorant binding (GO:0005549) |
| CnigOBP19 | 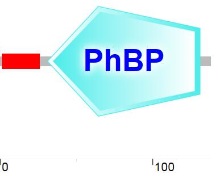 | Insect pheromone/odorant binding protein domains | 34 to 95 | 3.00E-06 | PBP_GOBP (IPR006170) | odorant binding (GO:0005549) |
| CnigOBP20 | 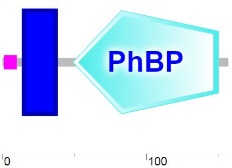 | Insect pheromone/odorant binding protein domains | 49 to 150 | 3.00E-65 | PBP_GOBP (IPR006170) | odorant binding (GO:0005549) |

Table S6 Conserved domains of chemosensory proteins in *C. nigricornis*.

| Proteins | Domains | Domain definition | Position (AA) | E-value | Interpro family |
| --- | --- | --- | --- | --- | --- |
| CnigCSP1 | 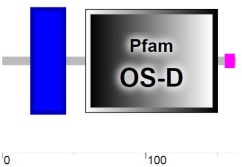 | Insect pheromone-binding family, A10/OS-D | 58 to 150 | 5.90E-34 | IPR005055 OS_D_A10/PebIII |
| CnigCSP2 | 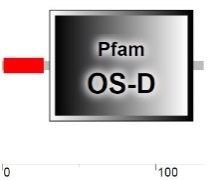 | Insect pheromone-binding family, A10/OS-D | 31 to 124 | 1.60E-24 | IPR005055 OS_D_A10/PebIII |
| CnigCSP3 | 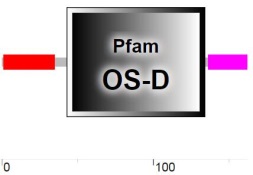 | Insect pheromone-binding family, A10/OS-D | 43 to 134 | 4.50E-32 | IPR005055 OS_D_A10/PebIII |
| CnigCSP4 | 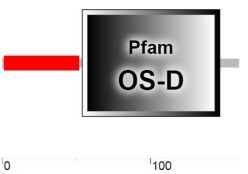 | Insect pheromone-binding family, A10/OS-D | 54 to 147 | 2.10E-25 | IPR005055 OS_D_A10/PebIII |
| CnigCSP5 | 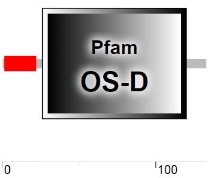 | Insect pheromone-binding family, A10/OS-D | 26 to 120 | 1.40E-22 | IPR005055 OS_D_A10/PebIII |
| CnigCSP6 | 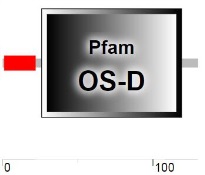 | Insect pheromone-binding family, A10/OS-D | 26 to 119 | 1.10E-32 | IPR005055 OS_D_A10/PebIII |
| CnigCSP7 | 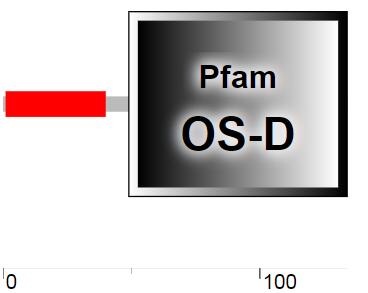 | Insect pheromone-binding family, A10/OS-D | 49 to 134 | 3.90E-16 | IPR005055 OS_D_A10/PebIII |
| CnigCSP8 | 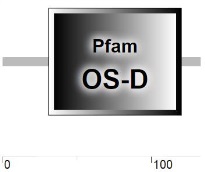 | Insect pheromone-binding family, A10/OS-D | 31 to 120 | 3.70E-21 | IPR005055 OS_D_A10/PebIII |
| CnigCSP9 | 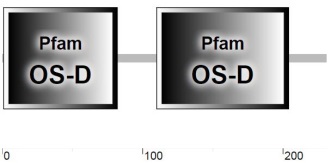 | Insect pheromone-binding family, A10/OS-D | 1 to 82 and 110 to 204 | 2.3E-24 and 4.8E-33 | IPR005055 OS_D_A10/PebIII |
| CnigCSP10 | 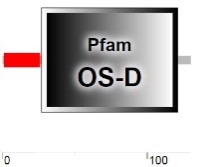 | Insect pheromone-binding family, A10/OS-D | 27 to 121 | 1.60E-34 | IPR005055 OS_D_A10/PebIII |

Table S7 Primers used for qRT-PCR.

| **Genes** | **Forward primers (5’-3’)** | **Reverse primers (5’-3’)** |
| --- | --- | --- |
| CnigOBP1 | GGAAGGCAGACACCCTCCT | TCGAAGCCAGTCGTATATTGTCT |
| CnigOBP3 | TACTGTCTCCCAGCGAACC | TCGTCACCCTTCGTCGGAT |
| CnigOBP4 | GCTACCATGACGTTTGCTTCC | GTACGTGTCGTTGCATTCGT |
| CnigOBP6 | CACGATACGACATGTGGGC | AGATGCACGGCACTTGGTAT |
| CnigOBP11 | TATACTCGACGCAGCACCTC | ACCTTTGCTGGGATGAGCC |
| CnigOBP12 | TTTCCTACCTGTCGCCGTGA | GTTTTGAGCCCACCGAGACTA |
| CnigOBP13 | GCAGCACCCACCAGACG | GGGTGCCTTGCCAGTCTTG |
| CnigOBP14 | AATTCCTGCAGCCATGAACTGG | TTCATCGGTATGACATTTTAGCCCT |
| CnigOBP15 | ACCTCTACCACCACTCCGTC | TTTGTCCATGTCACTCCCCG |
| CnigOBP16 | TTCATACACTGCCTGACGGAC | TCTAGCAGCGACGCAATCG |
| CnigOBP19 | AACGGCGAGGTGTTTCACT | ATCATCACTGGGGTCGTGCT |
| CnigOBP20 | TGGAAGGACTCCAGGAAGGG | CTTCGCAGCATTCACGTTGT |
| CnigCSP1 | TCCCACCGGCCTTCCAG | CGATACTGTCGTAGCGTGTAGT |
| CnigCSP2 | CGACTTATGTGACCCCGGAA | TTGAAGAGGTCGGGGTGTTTC |
| CnigCSP3 | TCCTTCCTGACGCCCTGA | TTGGCCTCCAGGCGTTT |
| CnigCSP4 | CAGTCTGACGCAACAGCCAT | AAGACTTGAGGAGGCGTTCG |
| CnigCSP5 | CGTTTGCACTTTCGCTGCTC | TCAATGCGGTCCTTGTTGGC |
| CnigCSP6 | GGCAGTCGTAGCATTTCTCG | CATCAGGCGCTCGTTGTGTA |
| CnigCSP7 | ACAAGATGCCCTGCGATGAG | CTGTCTCACTGTCTCAGCGTT |
| CnigCSP8 | GCCACCTGACAACCAAAAGAAA | CTGCTCCCAGTGCTTGCG |
| CnigCSP9 | CAGCGACCGGCTACTCA | AGAGCGTCTGGGATGACTTC |
| CnigCSP10 | GACCCTGACCCTCGTTCTCG | CAGGCGCTCATTGTTGAGGAT |
| CnigOR6 | CGTGCCCATCTGTGTGGTTA | CGTCTGCCTCATCGTCTCC |
| CnigOR10 | CCGCTGGTGAAGACAGATCA | GAGATGTAACCAGGGAGGCG |
| CnigOR13 | GCTACAGGACATTAGGGGGC | GGAGGTGTCGAGCAGTATGG |
| CnigOR18 | CGCTTTTCTTCGTGCGTGAC | AAGGCGGAATACACGTCTGC |
| CnigOR20 | GAAGCTCAACATTCGCCACC | TGGTCAGCAAGCACTCTGTT |
| CnigOR32 | TGTGCCTCGACCTGTTCTTC | CGACCTTGGTATCCTCTCGC |
| CnigOR35 | CTACTACGGTCAGATGGTGGC | CGAATGGAAACCAGACGGGA |
| CnigOR37 | GCATCGCCTCTCTAGGACAC | TGCACGAACGTAACTCTGCT |
| CnigOR55 | GGCCTTCGTCTCGCAAATAA | CGTCTTACTGGTTTTATGCGGT |
| CnigOR60 | CCTCACATGGGGACTCAGGA | CGCGCCTGTGGTACTTCTTA |
| CnigOR65 | AATGCACGCCTATCGAGCTT | TACACGTCTCTCGTTCGTCC |
| CnigOR70 | TCGAGTGTTCTCATGCTGGC | TAACATACGCCGGGAAGGTG |
| β-actin | CTAAGCAAAGCCAAGACCGT | ATACCCACCATCACACCCTG |
