# Supplementary material for: Sex- and tissue-specific transcriptome analyses and expression profiling of olfactory-related genes in Ceracris nigricornis Walker (Orthoptera: Acrididae)
Source: BMC Genomics. 2019 Nov 6;20:808. doi: 10.1186/s12864-019-6208-x (PMC6836668; doi:10.1186/s12864-019-6208-x)

**Additional file 2:**

Fig S1 Insect species distribution of *C. nigricornis* unigenes' best-hit annotation term in NR database.


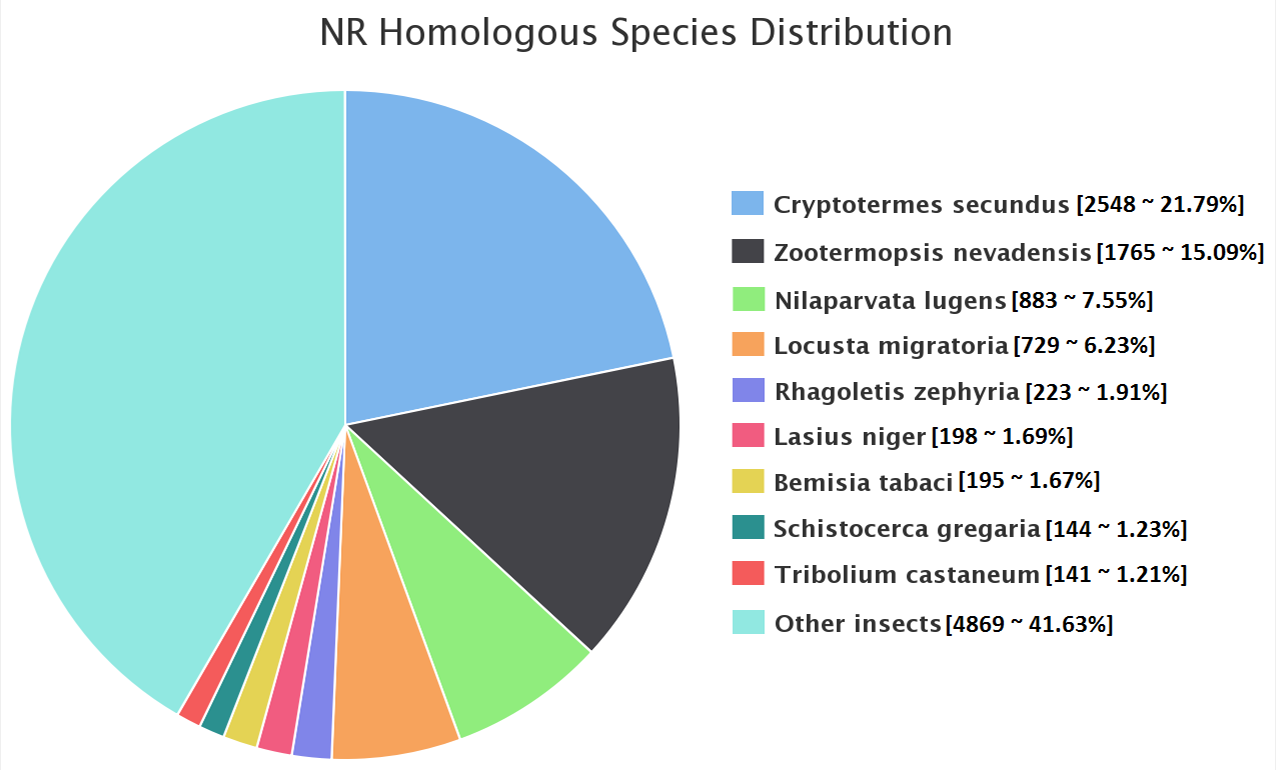


Fig S2 Gene ontology (GO) classifications of *C. nigricornis* unigenes.


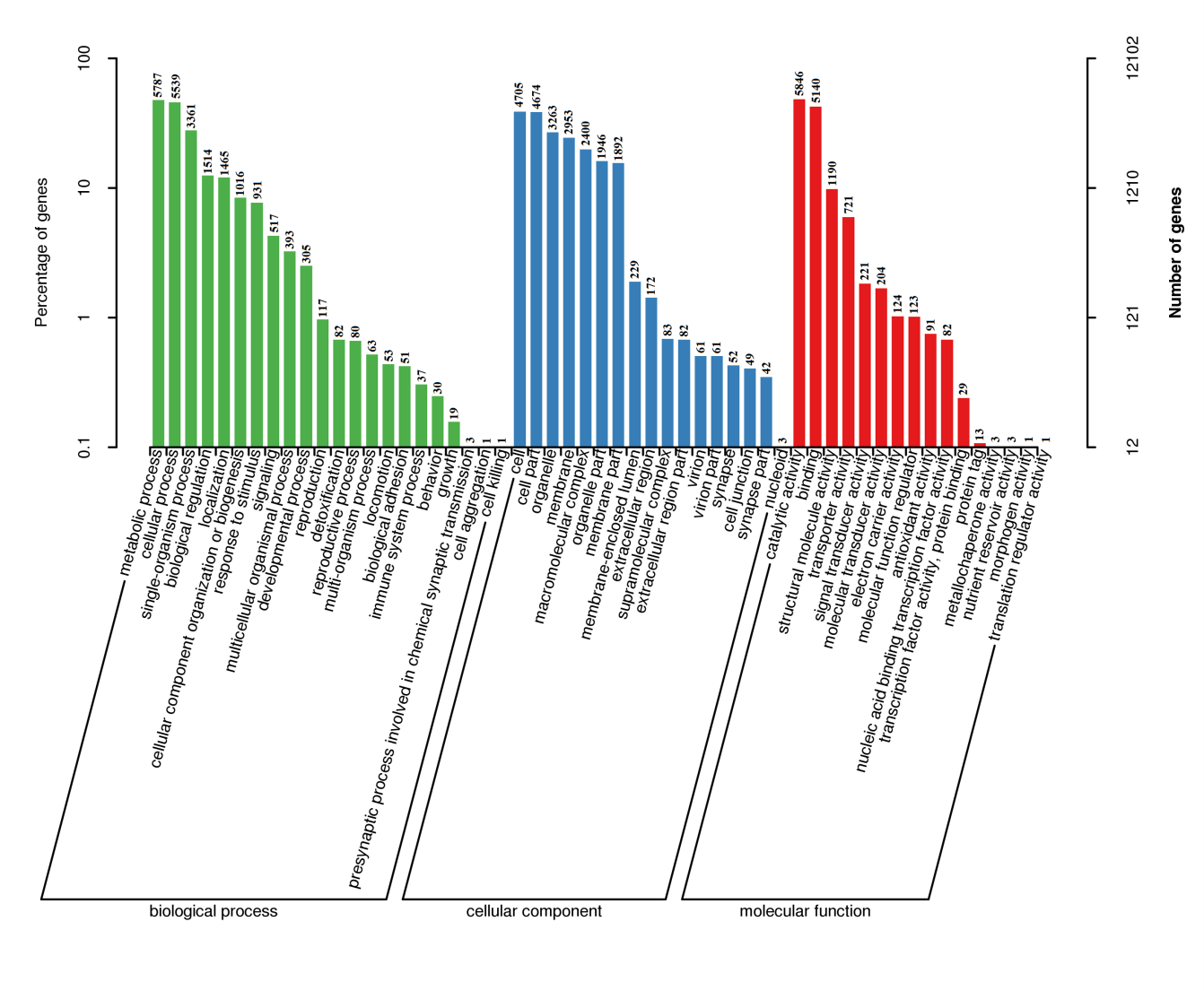


Fig S3 Alignments of the *C. nigricornis* odorant-binding proteins (OBPs). Boxes show the six conserved cysteine residues.


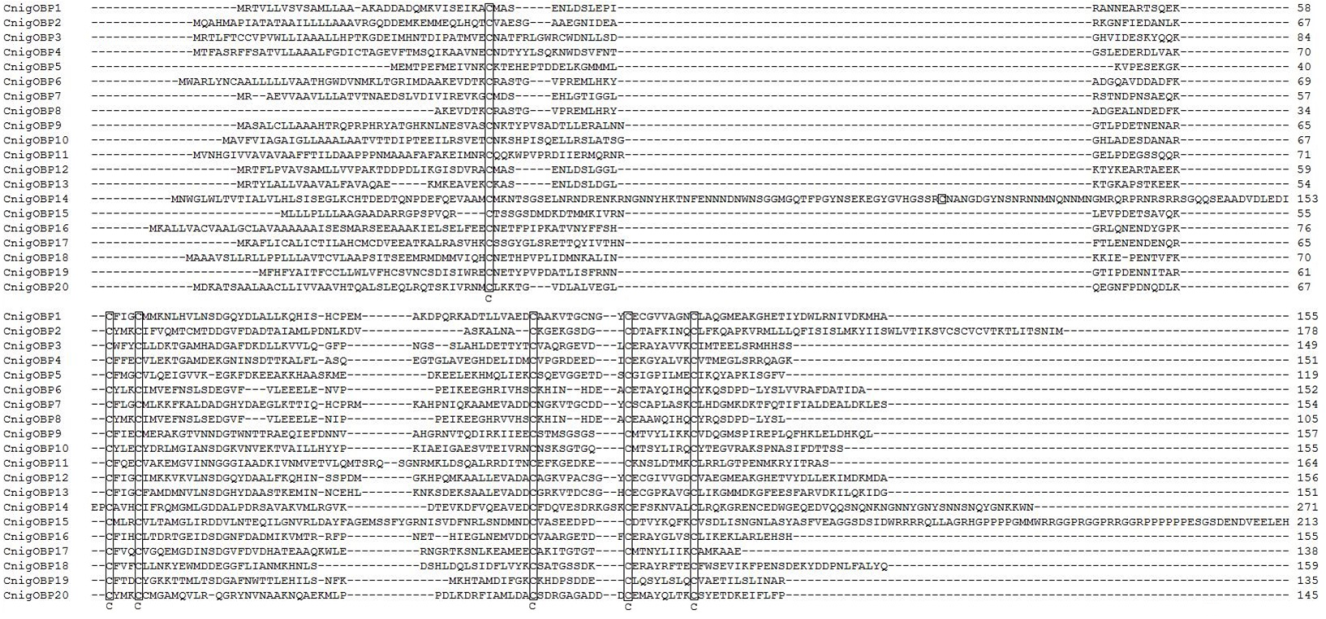


Fig S4 Alignments of the *C. nigricornis* chemosensory-binding proteins (CSPs). Boxes show the four conserved cysteine residues.


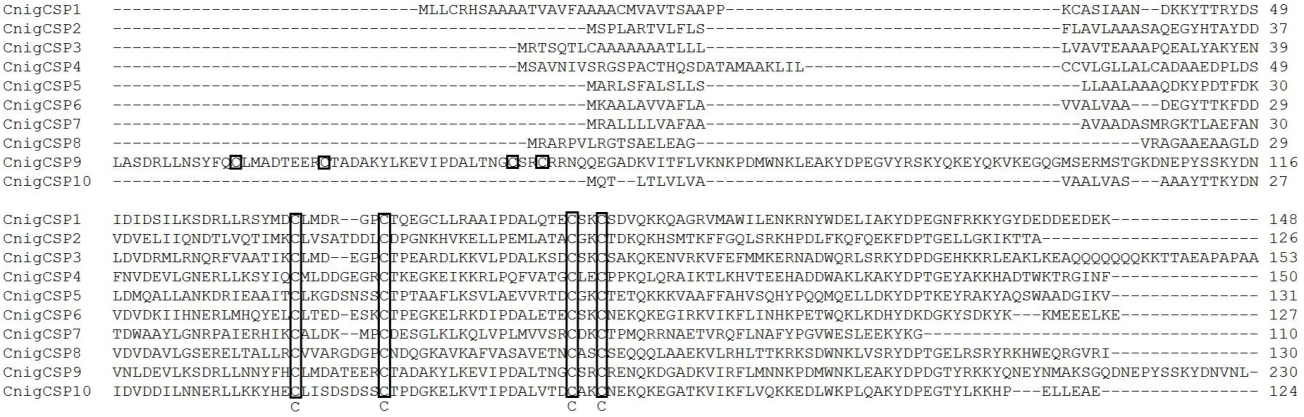


Fig S5 Phylogenetic tree of odorant-binding proteins (OBPs) from *C. nigricornis* and other insects. The amino acid sequences of the 121 OBPs were used in this analysis are listed in Additional file 3 table S1. *C. nigricornis* (Cnig), *C. kiangsu* (Ckia), *L. migratoria* (Lmig), *O. asiaticus* (Oasi), *O. infernalis* (Oinf), *S. gregaria* (Sgre), *A. glycines* (Agly), *D. ponderosae* (Dpon) and *H. armigera* (Harm). The OBPs of *C. nigricornis* are represented by red font.


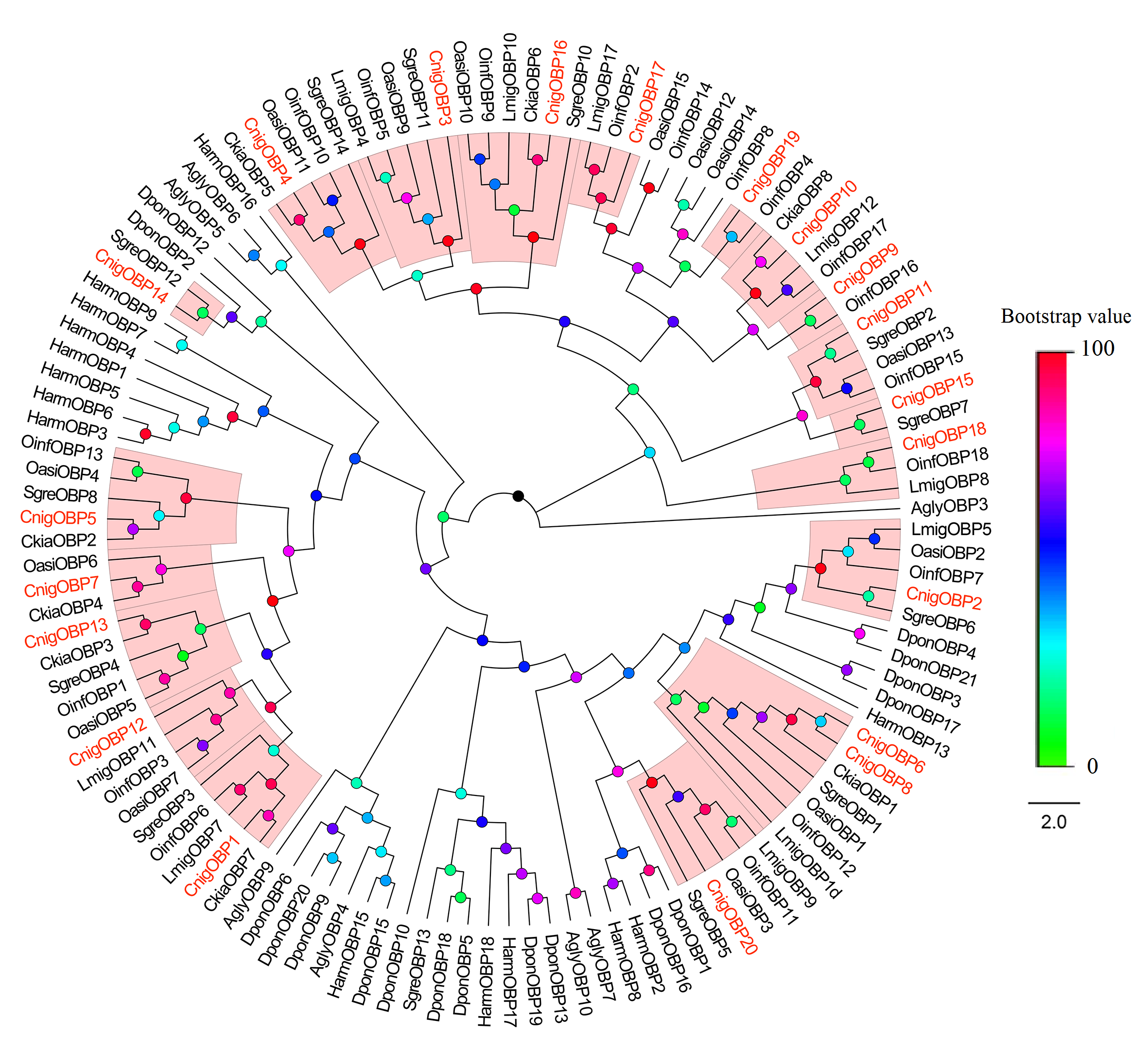


Fig S6 Phylogenetic tree of chemosensory-binding proteins (CSPs) from *C. nigricornis* and other insects. The amino acid sequences of the 87 CSPs were used in this analysis are listed in Additional file 3 table S2. *C. nigricornis* (Cnig), *L. migratoria* (Lmig), *O. asiaticus* (Oasi), *O. infernalis* (Oinf), *A. gambiae* (Agam), *D. ponderosae* (Dpon) and *H. armigera* (Harm). The CSPs of *C. nigricornis* are represented by red font.


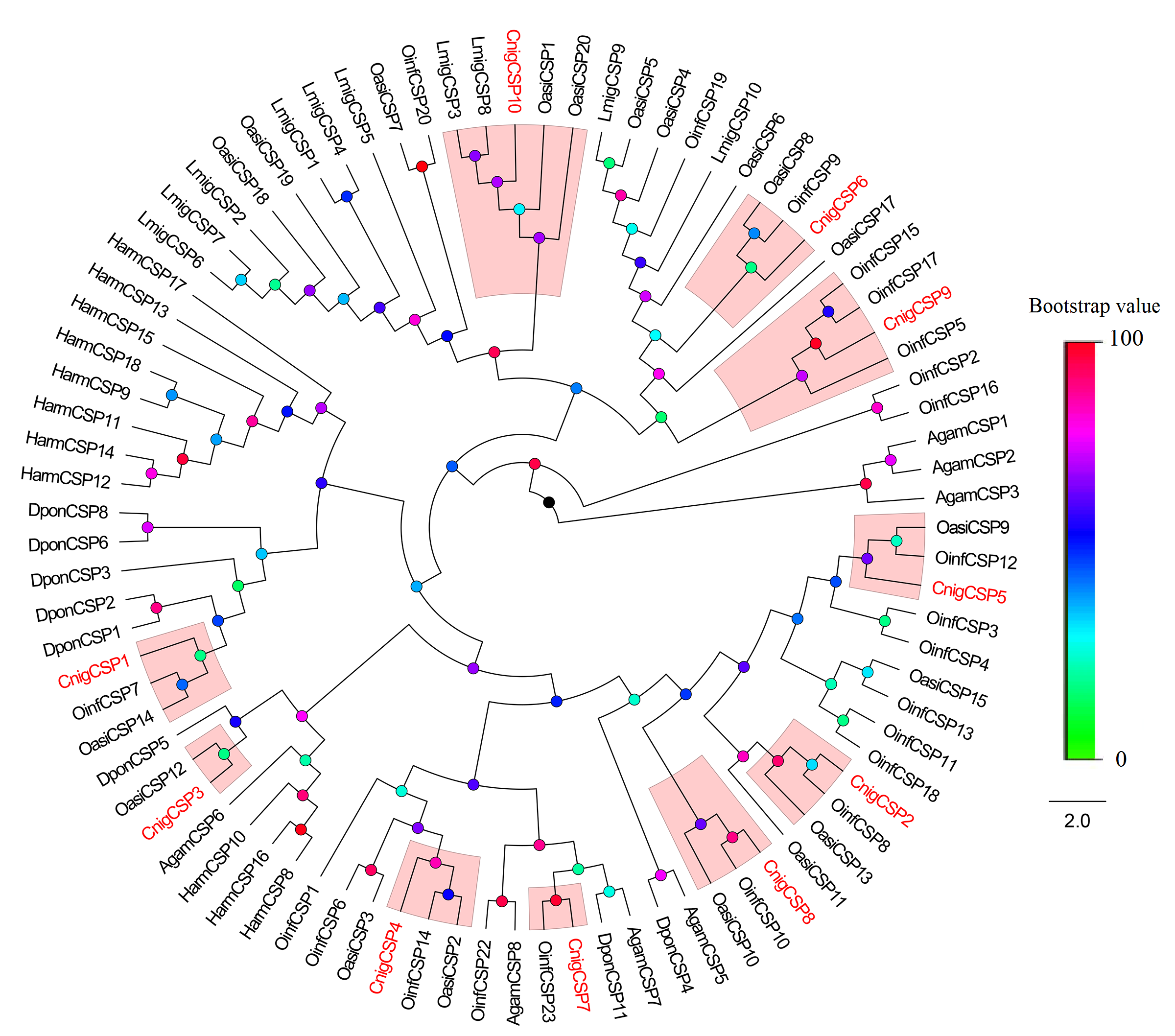


Fig S7 Phylogenetic tree of odorant receptors (ORs) from *C. nigricornis* and other insects. The amino acid sequences of the 293 ORs were used in this analysis are listed in Additional file 3 table S3. *C. nigricornis* (Cnig), *L. migratoria* (Lmig) and *A. lineolatus* (Alin). The ORs of *C. nigricornis* are represented by red font.


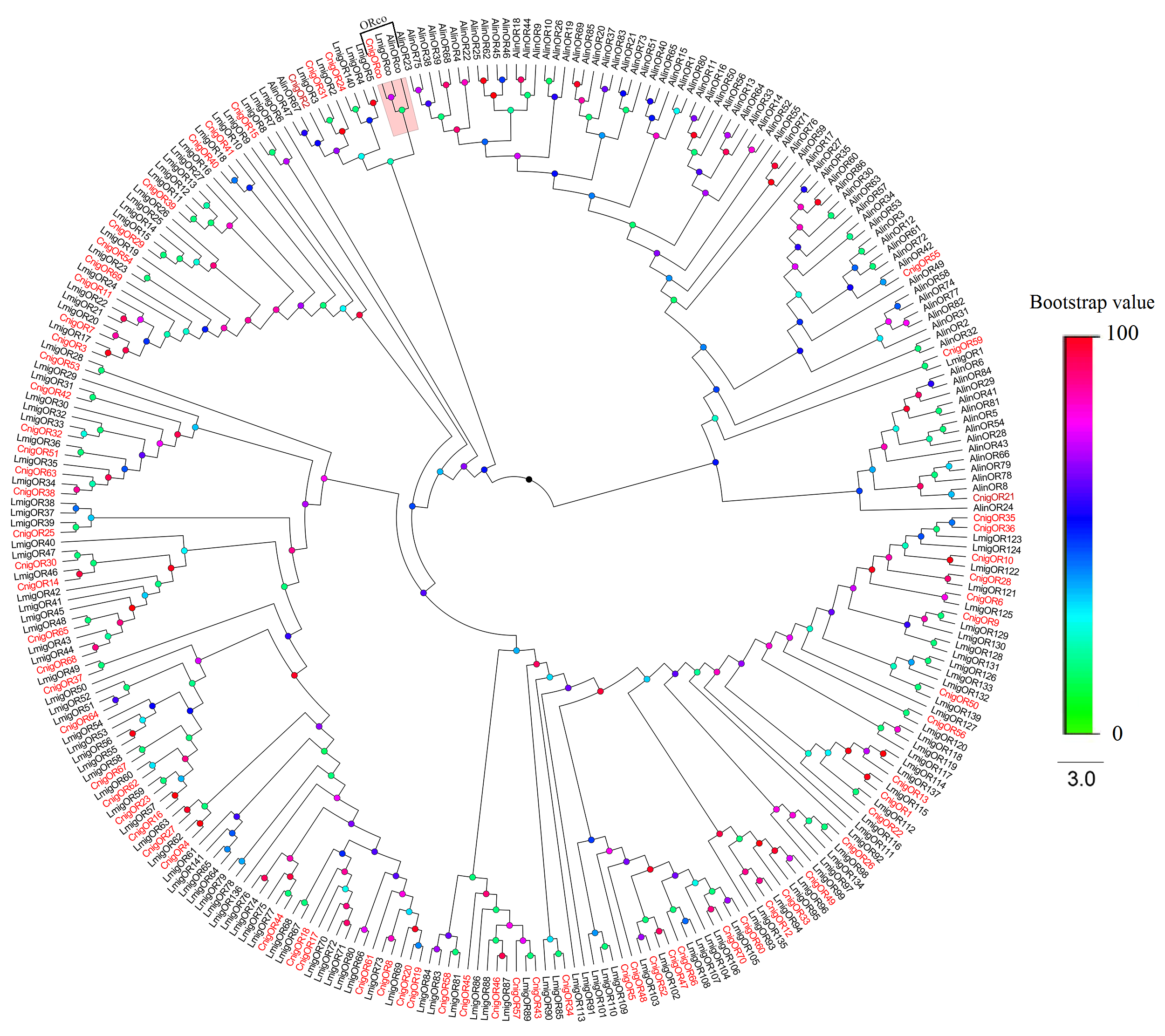


Fig S8 Phylogenetic tree of ionotropic receptors (IRs) from *C. nigricornis* and other insects. The amino acid sequences of the 115 IRs were used in this analysis are listed in Additional file 3 table S4. *C. nigricornis* (Cnig), *L. migratoria* (Lmig), *O. asiaticus* (Oasi), *A. lineolatus* (Alin) and *D. melanogaster* (Dmel). The IRs of *C. nigricornis* are represented by red font.


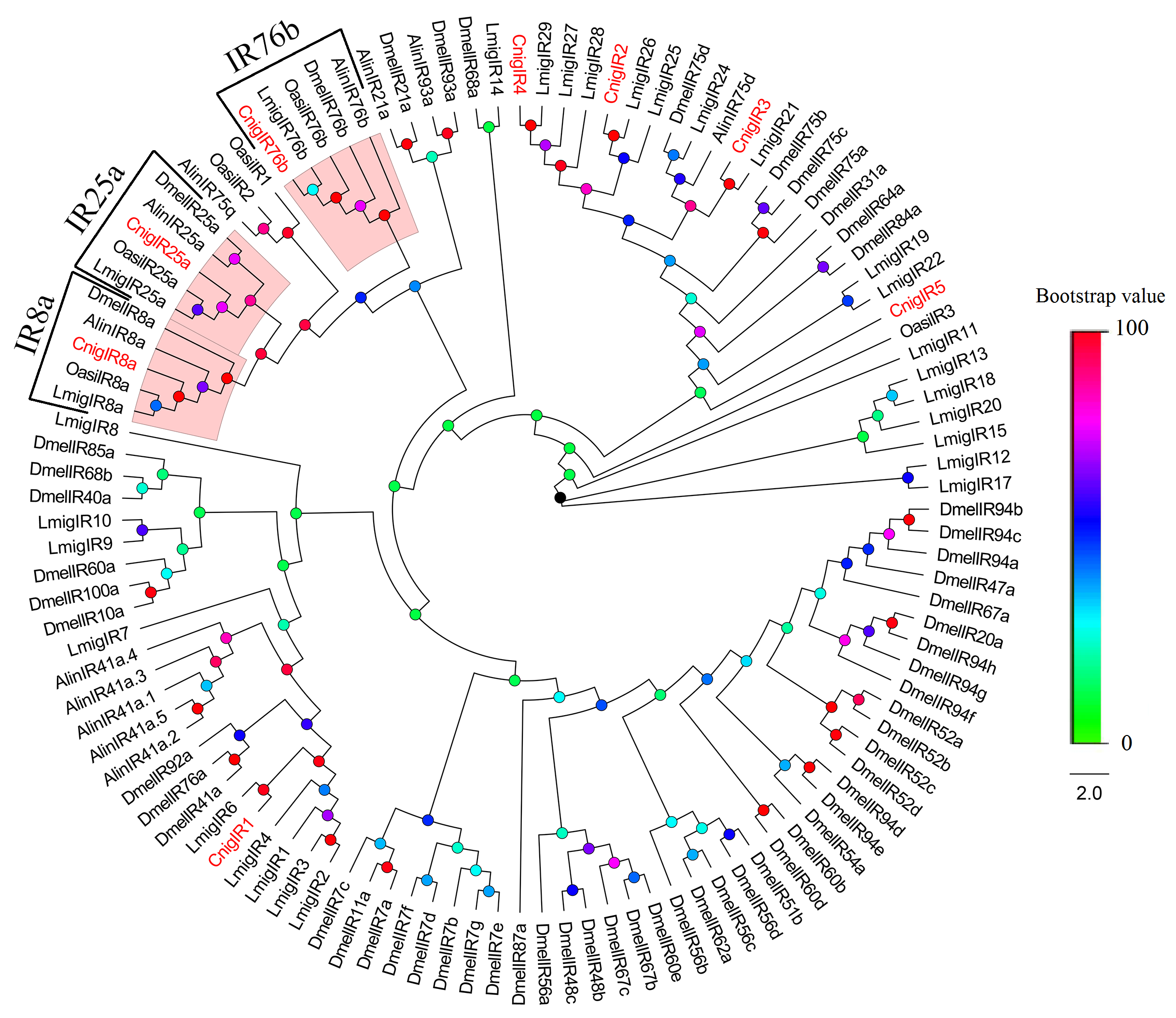


Fig S9 Phylogenetic tree of sensory neuron membrane proteins (SNMPs) from *C. nigricornis* and other insects. The amino acid sequences of the 24 SNMPs were used in this analysis are listed in Additional file 3 table S5. *C. nigricornis* (Cnig), *A. lineolatus* (Alin), *A. aegypti* (Aaeg), *A. mellifera* (Amel), *B. mori* (Bmor), *D. melanogaster* (Dmel), *O. asiaticus* (Oasi), *S. gregaria* (Sgre) *and T. castaneum* (Tcas). The SNMPs of *C. nigricornis* are represented by red font.


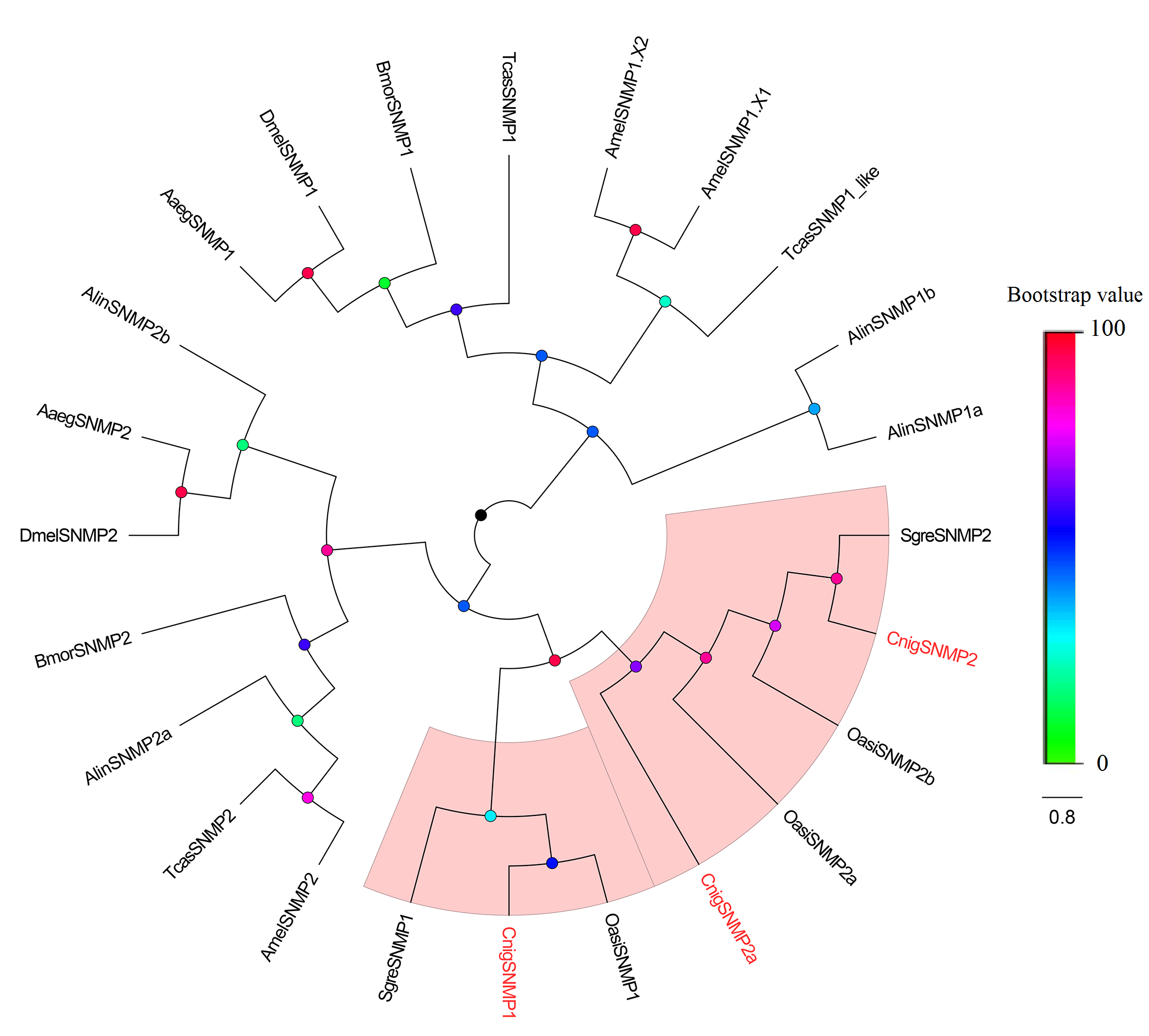

Supplement: Supplementary file 2 — Additional file 2: Figure S1. Insect species distribution of C. nigricornis unigenes’ best-hit annotation term in NR database. Figure S2. Gene ontology (GO) classifications of C. nigricornis unigenes. Figure S3. Alignments of the C. nigricornis odorant binding proteins (OBPs). Boxes show the six conserved cysteine residues. Figure S4. Alignments of the C. nigricornis chemosensory proteins (CSPs). Boxes show the four conserved cysteine residues. Figure S5. Phylogenetic tree of odorant-binding proteins (OBPs) from C. nigricornis and other insects. C. kiangsu (Ckia), L. migratoria (Lmig), O. asiaticus (Oasi), O. infernalis (Oinf), S. gregaria (Sgre), A. glycines (Agly), D. ponderosae (Dpon) and H. armigera (Harm). The OBPs of C. nigricornis are represented by red font. Figure S6. Phylogenetic tree of chemosensory-binding proteins (CSPs) from C. nigricornis and other insects. Table S2. L. migratoria (Lmig), O. asiaticus (Oasi), O. infernalis (Oinf), A. gambiae (Agam), D. ponderosae (Dpon) and H. armigera (Harm). The CSPs of C. nigricornis are represented by red font. Figure S7. Phylogenetic tree of odorant receptors (ORs) from C. nigricornis and other insects. Table S3. L. migratoria (Lmig) and A. lineolatus (Alin). The ORs of C. nigricornis are represented by red font. Figure S8. Phylogenetic tree of ionotropic receptors (IRs) from C. nigricornis and other insects. L. migratoria (Lmig), O. asiaticus (Oasi), A. lineolatus (Alin) and D. melanogaster (Dmel). The IRs of C. nigricornis are represented by red font. Figure S9. Phylogenetic tree of sensory neuron membrane proteins (SNMPs) from C. nigricornis and other insects. A. lineolatus (Alin), A. aegypti (Aaeg), A. mellifera (Amel), B. mori (Bmor), D. melanogaster (Dmel), O. asiaticus (Oasi), S. gregaria (Sgre) and T. castaneum (Tcas). The SNMPs of C. nigricornis are represented by red font. [file 12864_2019_6208_MOESM2_ESM.docx]
