# Supplementary material for: Sex- and tissue-specific transcriptome analyses and expression profiling of olfactory-related genes in Ceracris nigricornis Walker (Orthoptera: Acrididae)
Source: BMC Genomics. 2019 Nov 6;20:808. doi: 10.1186/s12864-019-6208-x (PMC6836668; doi:10.1186/s12864-019-6208-x)
Supplement: Supplementary file 3 — Additional file 3: Table S1. Amino acid sequences of 121 OBPs of Ceracris nigricornis and other insect species used to construct phylogenetic tree. Table S2. Amino acid sequences of 87 CSPs of Ceracris nigricornis and other insect species used to construct phylogenetic tree. Table S3. Amino acid sequences of 293 ORs of Ceracris nigricornis and other insect species used to construct phylogenetic tree. Table S4. Amino acid sequences of 115 IRs of Ceracris nigricornis and other insect species used to construct phylogenetic tree. Table S5. Amino acid sequences of 24 SNMPs of Ceracris nigricornis and other insect species used to construct phylogenetic tree. [file 12864_2019_6208_MOESM3_ESM.docx]

**Additional file 3**:

Table S1 Amino acid sequences of 121 OBPs of *Ceracris nigricornis* and other insect species used to construct phylogenetic tree.

>CnigOBP1

MRTVLLVSVSAMLLAAAKADDADQMKVISEIKACMASENLDSLEPIRANNEARTSQEKCFIGCMMKNLHVLNSDGQYDLALLKQHISHCPEMAKDPQRKADTLLVAEDCAAKVTGCNGYCECGVVAGNCLAQGMEAKGHETIYDWLRNIVDKMHA

>CnigOBP2

MQAHMAPIATATAAILLLLAAAVRGQDDEMKEMMEQLHQTCVAESGAAEGNIDEARKGNFIEDANLKCYMKCIFVQMTCMTDDGVFDADTAIAMLPDNLKDVASKALNACKGEKGSDGCDTAFKINQCLFKQAPKVRMLLLQFISISLMKYIISWLVTIKSVCSCVCVTKTLITSNIM

>CnigOBP3

MRTLFTCCVPVWLLIAAALLHPTKGDEIMHNTDIPATMVECNATFRLGWRCWDNLLSDGHVIDESKYQQKCWFYCLLDKTGAMHADGAFDKDLLKVVLQGFPNGSSLAHLDETTYTCVAQRGEVDLCERAYAVVKCIMTEELSRMHHSS

>CnigOBP4

MTFASRFFSATVLLAAALFGDICTAGEVFTMSQIKAAVNECNDTYYLSQKNWDSVFNTGSLEDERDLVAKCFFECVLEKTGAMDEKGNINSDTTKALFLASQEGTGLAVEGHDELIDMCVPGRDEEDICEKGYALVKCVTMEGLSRRQAGK

>CnigOBP5

MEMTPEFMEIVNKCKTEHEPTDDELKGMMMLKVPESEKGKCFMGCVLQEIGVVKEGKFDKEEAKKHAASKMEDKEELEKHMQLIEKCSQEVGGETDSCGIGPILMECIKQYAPKISGFV

>CnigOBP6

MWARLYNCAALLLLLVAATHGWDVNMKLTGRIMDAAKEVDTKCRASTGVPREMLHKYADGQAVDDADFKCYLKCIMVEFNSLSDEGVFVLEEELENVPPEIKEEGHRIVHSCKHINHDEACETAYQIHQCYKQSDPDLYSLVVRAFDATIDA

>CnigOBP7

MRAEVVAAVLLLATVTNAEDSLVDIVIREVKGCMDSEHLGTIGGLRSTNDPNSAEQKCFLGCMLKKFKALDADGHYDAEGLKTTIQHCPRMKAHPNIQKAAMEVADDCNGKVTGCDDYCSCAPLASKCLHDGMKDKTFQTIFIALDEALDKLES

>CnigOBP8

AKEVDTKCRASTGVPREMLHRYADGEALNDEDFKCYMKCIMVEFNSLSEDGVFVLEEELENIPPEIKEEGHRVVHSCKHINHDEACEAAWQIHQCYRQSDPDLYSL

>CnigOBP9

MASALCLLAAAHTRQPRPHRYATGHKNLNESVASCNKTYPVSADTLLERALNNGTLPDETNENARCFIECMERAKGTVNNDGTWNTTRAEQIEFDNNVAHGRNVTQDIRKIIEECSTMSGSGSCMTVYLIKKCVDQGMSPIREPLQFHKLELDHKQL

>CnigOBP10

MAVFVIAGAIGLLAAALAATVTTDIPTEEILRSVETCNKSHPISQELLRSLATSGGHLADESDANARCYLECYDRLMGIANSDGKVNVEKTVAILLHYYPKIAEIGAESVTEIVRNCNSKSGTGQCMTSYLIRQCYTEGVRAKSPNASIFDTTSS

>CnigOBP11

MVNHGIVVAVAVAAFFTILDAAPPPNMAAAFAFAKEIMNRCQQKWPVPRDIIERMQRNRGELPDEGSSQQRCFQECVAKEMGVINNGGGIAADKIVNMVETVLQMTSRQSGNRMKLDSQALRRDITNCEFKGEDKECKNSLDTMKCLRRLGTPENMKRYITRAS

>CnigOBP12

MRTFLPVAVSAMLLVVPAKTDDPDLIKGISDVRACMASENLDSLGGLKTYKEARTAEEKCFIGCIMKKVKVLNSDGQYDAALFKQHINSSPDMGKHPQMKAALLEVADACAGKVPACSGYCECGIVVGDCVAEGMEAKGHETVYDLLEKIMDKMDA

>CnigOBP13

MRTYLALLVAAVALFAVAQAEKMKEAVEKCKASENLDSLDGLKTGKAPSTKEEKCFIGCFAMDMNVLNSDGHYDAASTKEMINNCEHLKNKSDEKSAALEVADDCGRKVTDCSGHCECGPKAVGCLIKGMMDKGFEESFARVDKILQKIDG

>CnigOBP14

MNWGLWLTVTIALVLHLSISEGLKCHTDEDTQNPDEFQEVAAMCMKNTSGSELNRNDRENKRNGNNYHKTNFENNNDNWNSGGMGQTFPGYNSEKEGYGVHGSSRCNANGDGYNSNRNNMNQNNMNGMRQRPRNRSRRSGQQSEAADVDLEDIEPCAVHCIFRQMGMLGDDALPDRSAVAKVMLRGVKDTEVKDFVQEAVEDCFDQVESDRKGSKCEFSKNVALCLRQKGRENCEDWGEQEDVQQSNQNKNGNNYGNYSNNSNQYGNKKWN

>CnigOBP15

MLLLPLLLAAGAADARRGPSPVQRCTSSGSDMDKDTMMKIVRNLEVPDETSAVQKCMLRCVLTAMGLIRDDVLNTEQILGNVRLDAYFAGEMSSFYGRNISVDFNRLSNDMNDCVASEEDPDCDTVYKQFKCVSDLISNGNLASYASFVEAGGSDSIDWRRRRQLLAGRHGPPPPGMMWRRGGPRGGPRRGGRPPPPPPESGSDENDVEELEH

>CnigOBP16

MKALLVACVAALGCLAVAAAAAAISESMARSEEAAAKIELSELFEECNETFPIPKATVNYFFSHGRLQNENDYGPKCFIHCLTDRTGEIDSDGNFDADMIKVMTRRFPNETHIEGLNEMVDDCVAARGETDFCERAYGLVSCLIKEKLARLEHSH

>CnigOBP17

MKAFLICALICTILAHCMCDVEEATKALRASVHKCSSGYGLSRETTQYIVTHNFTLENENDENQRCFVQCVGQEMGDINSDGVFDVDHATEAAQKWLERNGRTKSNLKEAMEECAKITGTGTCMTNYLIIKCAMKAAE

>CnigOBP18

MAAAVSLLRLLPPLLLAVTCVLAAPSITSEEMRMDMMVIQHCNETHPVPLIDMNKALINKKIEPENTVFKCFVFCLLNKYEWMDDEGGFLIANMKHNLSDSHLDQLSIDFLVYKCSATGSSDKCERAYRFTECFWSEVIKFPENSDEKYDDPNLFALYQ

>CnigOBP19

MFHFYAITFCCLLWLVFHCSVNCSDISIWRECNETYPVPDATLISFRNNGTIPDENNITARCFTDCYGKKTTMLTSDGAFNWTTLEHILSNFKMKHTAMDIFGKCKHDPSDDECLQSYLSLQCVAETILSLINAR

>CnigOBP20

MDKATSAALAACLLIVVAAVHTQALSLEQLRQTSKIVRNMCLKKTGVDLALVEGLQEGNFPDNQDLKCYMKCCMGAMQVLRQGRYNVNAAKNQAEKMLPPDLKDRFIAMLDACSDRGAGADDDCEMAYQLTKCSYETDKEIFLFP

>CkiaOBP1

MWARLYNCAALLLLLVAATHGWDVNMKLTGRIMDAAKEVDTKCRSSTGVPREMLHRYADGQTVDDADFKCYLKCIMIEFNSLSDEGVFVLEEELENVPPEIKEEGHRIVHSCKHINHDEACETAYQIHQCYKQSDPDLYSLVVRAFDATIDA

>CkiaOBP2

MRTSAAAAGAALLVLAAVASAMEMTPEFMEIVNKCKTEHEPTDDELKGMMMLKVPESEKGKCFMGCVLQEIGVVKEGKFDKEEAKKHAESKMTDKDELEKHMQLIEKCSQEVGGETDSCGIGPKLMECIKQFAPEFDIALPQPPSE

>CkiaOBP3

MRTYLALLVAAVALFAVAQAGPEDKLKESVEKCKASENLDNLDGLKTGKAPSTKEEKCFIGCFAMDMNVLNSDGHYDAASTKEMINNCEHLKNKPDEKSAALEVADDCGHKVTDCSGHCECGPKAVGCLIKGMMDKGFEESFASLDKVLQKVDG

>CkiaOBP4

MRVEVVAAVLLLAAVTNAEDSLVDIVIREVKGCMDSEHLGSIGGLRSTNDPNSAEQKCFLGCMLKKFKALDAGGHYDAEGLKTTIQHCPRMKAHPDIQKAAMQVADECNGKVTGCDDYCSCAPLASKCLHEGMKNKAFQTIFIALDEALDKMES

>CkiaOBP5

NWDSVFTTGSLEDERDLVAKCFFECVLEKTGAMDEKGNINSDTTKALFLASQEGTGPAIEGHDELIDMCVPGRDEEDICEKGYALVKCVTMEELSRRQAGK

>CkiaOBP6

MKALLVAFVAALGCLALAVAAISESMARAEEAAAKIELSELFEECNETFPIPKATINYFFSHGRLQNENDYGSKCYIHCLTDRSGEIDSDGNFDADMIKVMTRRFPNETHIEGLNEMVDGCVAARGESDFCERAYGLVSCLIKEKLARLGHSH

>CkiaOBP7

MRTILLVSVSAMLLAATEADDADQMKVISEIKACMASENLDSLDPIRTNNEARTSQEKCFIGCMMKNLHVLNSDGQYDSALLKEHMSHCPEMAKDPQKKADTLLVAEDCAAKVTGCNGYCECGVVAGNCLAQGMEAKGHETKYTTGFETSWRRCMLRSGPSGT

>CkiaOBP8

MAVSVIAGALGLLAAALATTVTTDIPTEEILRWVETCNKSHPISQELLRSLATSGGLLADESDTNARCYLECYDRLVGVANSDGMLNVENVVAILIHYYPKIAEIGAESVAEIVRNCSSKSGTGQCMTSYLIRKCYTEGLGVKSPNVSIFDSSFS

>LmigOBP1d

MWARLNDCAALLLLLAAAARAWDVNMKLTGRIMDAAKEVDHTCRSSTGVPRDMLHRYAEGQTVDDDDFKCYLKCIMVEFNSLSDDGVFVLEEELENVPPEIKEEGHRVVHSCKHINHDEACETAYQIHQCYKQSDPELYSLVVRAFDATIGD

>LmigOBP4

CDPRHDTATMSALFTCCVAAWLLLAAALLQPTKGDDVWHNTDIPATMAECNATFRLGWRCWDNLLSDGHVIDESKYQQKCWFYCLLDETGSMHADGAFDKDLLKTVLQGFPNGSSLAHLDETTYTCVAQRNEVDLCERAYAVVKCIMTEELSRMHQSS

>LmigOBP5

MCLVVLLFGFVQSLSLLFVPDSCLLLFVVVMFYFPCKISQANGSFVMTMLCLEQTCKMMDQLHQTCVGESGVSEGNIDAARKGNFIDDGNLKCYMKCIFVQMTCMSDDGVFDADTAIAMLPDNLKDVASKALNACKGEKGSDACDTAFKINQCLFKQAPKDYILV

>LmigOBP7

AAETKVMEGIKACMASEHLGSLGQLKANNEARTPEEKCFVGCVMKHLHVLNSEGQYDLALVKERANNCPELAKDPQKKADTLRVAEDCAAKVIGCSGYCECGVAAGECLAQGMEAKGHETIYDFLRKIVDKMDV

>LmigOBP8

AVRLLLLLPPLLLLALSCVTAAPSITSTEMRMDMMVIQHCNETHPVALIDMNKALINKKIEPQNTVFKCFVFCLLNKYEWMDDEGGFLIANMKHNLSDSHLDQLSIDFIVYKCSATGSSDKCERAYRFTECFWGEVTKFPENSDEKYEDPDLFALYQ

>LmigOBP9

TDATATAAMDKASSAAVTACLLIAVAALHTQALSLEQLRQTSKIVRNMCLKKTGVDLALVEGIQEGQFPDNQDLKCYMKCCMGAMQVLRQGRYNVNAAKNQADKMLPPDLKGRFIDMLDACSDRGDGVDDDCEMAYQLTKCSYETDKEIFLFP

>LmigOBP10

AISESMSRAEEAASKIDIPELFEECNETFTIPKVTLNYFFSHGRLQNENDYGSKCFVHCLTDRSGEIDSDGNFDVDLIKVMTRRFPNETNIEGLNEMVETCVADRGETDFCERAYGLVSCLVKEKLARLGNSH

>LmigOBP11

VATMRSLLPVAVSAVLLVAPSKTLEPDFTKGISDVKACMASENLDSLDALRTNKEARTAEEKCFIGCIMKFVEVLNSDGQYDVALFKDHINGCPEMAKDQQKKAALLEVAESCAGKASACSGHCECGVIVANCL

>LmigOBP12

AVILTAASTLWFAAAAFAAMVTTEIPTEDILQRVQVCNKTYPVSQEMLRSLASTGGLLSDESDVNTRCYLECYERLGGTVNKDGKFNPEKAVTLLVSYYPKIAELGVDSVTEILKNCNSKSGTGQCMTSYLIRNCFIAGLNAKSPHTSVFDTSSSHI

>LmigOBP17

MKAFQICTLICAVVAHCMCDKEEAMKILRASVDKCSAGYGLSRETTQYIVRHNFTIKDENDENQRCFVQCVGQEMGDFNSEGIFDVDHATETAEKWLEWNGRTKSNLREEMEECAKITGTGTCMTTYLITKCAMKAGE

>OasiOBP1

MWARLSDCAALLLLLASAARAWDVNMKLTGRIMDAAKEVDHTCRTSTGVPREMLHRYADGQTVDDDDFKCYLKCIMIEFNSLSDDGVFVLEEELENVPPEIKEEGHRVVHSCKHINHDEACETAYQIHQCYKQSDPELYSLVVRAFDATIGDD

>OasiOBP2

MKAAMAPLATATAAMLLLLAAAVRGQDDEMREMMDQLHQTCVGESGVSEGNIDAARKGNFIEDANLKCYMKCIFVQMTCMSDDGVFDADTAIAMLPDNLKDVASKALNACKGEKGSDACDTAFKINQCLFKQAPKDYILV

>OasiOBP3

MDKASAAAATAFLLIAVAALHAQALSLEQLRQTSKIVRNMCLKKTGVDLALVEGIQEGQFPDNQDLKCYMKCCMGAMQVLRQGRYNVNAAKNQAEKMLPPDLKDRFLSMLDACSDRGDGADDDCEMAYQLTKCSYETDKEIFLFP

>OasiOBP4

MRTSAAAAAAATGAALLVLAAVASAMEMTPEFMEIVNKCKTEHEPTEDELKGMMALKVPESSNGKCFMGCVLQEIGVVKDGKFDKEEAKKHAAAKMTDKDELEKHMQLIEKCSQEVGGETDSCGIGPKLMECIKQFAPEFDIALPKPSE

>OasiOBP5

MRTYLTLVFAAAALFAVAKADAEKVKEAVEKCKSSENLDSLDGLKSNKAPSTEEEKCFIGCMMMDMKLLSSDGQYDAASTKEMINSCEYLKDKPDEKSAALEVADDCAGKATGCSGHCECGPKAVGCLINGMVDKGYEESFARIDKMLQNLE

>OasiOBP6

MGAAVAAAVLLLVAVTNAEDSLMEIVIREVKGCMESEHLNSIGDLRSYNDASSPEQKCFLGCMLKKFKALDADGQYDAEGLKATIEHCPRMKALPNVQKAALQVADECAGKVTGCSDYCSCAPLAAKCLHEGMKNKSFQTIFIALDEALDKMQS

>OasiOBP7

MRTILPLAVSAMLLVAPSKTHEQDFTKGISDVKVCMASENLGSLDGLRANKEARTAEEKCFIGCLMKFVEVLNSDGQYDVALFKDHINRSPDLAKMQQKKAALLEVADSCAGKASACSGHCECGVIVANCLAEGMEAKGEETIYDLLEKIFAKMDA

>OasiOBP9

MSALFTCCVAAWLLMAAALLQPTKGDEVWHNTDIPATMAECNATFRLGWRCWDNLLSDGHVIDESKYQQKCWFYCLLDKTGAMHADGAFDKDLLKTVLQGFPNGSSLAHLDETTYTCVAQRNEVDLCERAYAVVKCIMTEELSRMHHSS

>OasiOBP10

MKALLVACVAALGCLAVAVAAISESMSRAEEAAAKIDLPELFEECNETFTTPKATLNYFFSHGRLQNENDYGSKCFIHCLTDRSGEIDSDGNFDVDLIKVMTRRFPNETNIEGLNEMVETCVADRGETDFCERAYGLVSCLIKEKLTRLGHSH

>OasiOBP11

MSLAARLFSVTLLLAPVLFSDISTAGEVFTMSQIKAAVNECNDTYFLSQKNWDSVFTTGSLEDEKDLVAKCFFECVLEKTGAMDEKGTINSDITKAVFLASHEGTGTPVQGHDELIDMCVPGRDETDICEKGYALVKCVTLEELSRRHARK

>OasiOBP12

MFYFYAFTLCCLLWVLFHCSVNCVDIDIETIWRECNETFPASEESLISFGKNGTIPDENDSTARCFADCYGKKTTMLTSDGSLNWTTLDFIMRSYNMKPTATETFGKCQKDTSNVECMKSYLSLRCVAETIASLSNIR

>OasiOBP13

MVNHHQGVVAIAAALTAMAAAAPSSIAEATRFSKETVSKCQEKWQVSEEIIEEMQRNKGALPNEDSVEQRCFAECVAKEMGMINNGGGVAADKIVKMLEAVFQMASKETGEKLKLDSRALKRDLEACQFKGEDDECTNSYDTLKCLRTLGTSDNMRRYVTKES

>OasiOBP14

MFYFYAFTLCCLLWVLFHCSVNCVDIDIETIWRECNETFPASEESLISFGKNGTIPDENDSTARCFADCYGKKTTMLTSDGSLNWTTLDFIMRSYNMKPTATETFGKCQKDIPTSHQLKTLCTSLSLKHVNPSHQFHRGRGHGWLLKHVNRGPCTQRCKYT

>OasiOBP15

MRTSHVYTIFCAIIVTCYCDSVEVSDGPEEATMMKCAVELGFGHDEIQRIKSSPIPDETNENERCLMKCIGRKMKYLTSEDIVDVHHLLELSGEMIEKEGYTKSEMRQMLVECTKKTGTEKCMTAFKNLRCLMNAFK

>OinfOBP1

MAAAGPRGHLVSTVPTADMRTCLTLVFAAAALFAVAKADAEKVKEAVEKCKSSENLDSLDGLKSNKAPSTEEEKCFIGCLMMDMKLLSSDGQYDAASTKEMINNCEYLKEKPDEKSAALEVADDCAGKATGCSGHCECGPKAVGCLINGMVDKGYEESFARMDKILEKLD

>OinfOBP2

MKGFQICTLICAVVAHCMCDKEEAIKILRASVDKCSTGYGLSRETTQYIVKHNFIIQDENDENQRCFVQCVGQELGDFNSEGIFDVDHATETAEKWLQWNGRNKSNLREAMEECAKITGTGTCMTTYLITKCAMKAGE

>OinfOBP3

MRTILTVAVSVMLLVAPSKTHEPDFTKGISDVKVCMASENLDSLDGLKANKEARTAEEKCFIGCLMKFVEVLNSDGQYDVALFKEHINRSPDLAMDQQKKAALLEVADSCAGKASACSGHCECGAVVANCLAEGMEAKGEETIYDLLEKIFAKMDA

>OinfOBP4

MFYFYALTFCCLLWLLFHCSVNCSDINIWRECNETYPVPDATLISFRNNGSIPDENNITARCFTDCYGKKTNMLTSDGSFNWTTLEHILSNFKMKHTAMDVLGKCRKDPSDDECMQSYLSLKCVAETILSLINAR

>OinfOBP5

MSALYTCCVAAWLLMAAALLQPAKGDEMWHNTDIPATMAECNATFRLGWRCWDNLLSDGHVIDESKYQQKCWFYCLLDKTGSMHADGAFDKDLLKTVLQGFPNGSSLAHLDETTYTCVAQRNEVDLCERAYAVVKCIMTEELSRMHHSS

>OinfOBP6

MRTLLLFSVSALLMAADAEADEATQTKVMADIKACMASENLGSLGQLRANSEARTAEEKCFVGCMMKNLHVLNSDGQYDLALLKERANDCPELARDPRKKADTLLVAEACAPQVTGCSGYCECGVIAGNCLARGMEAKGHETIYDFLRKTVDKMDV

>OinfOBP7

MKAAMAPLATATAAILLLLAAAVRGQDDEMREMMDQLHQTCVGESGVSEGNIDAARKGNFIEDANLKCYMKCIFVQMTCMSDDGVFDADTAIAMLPDNLKDVASKALNACKGEKGSDACDTAFKINQCLFKQAPKDYILV

>OinfOBP8

MFYFYAFTFCYLLWMLFHCSVDCVDIDIGTIWRECNETFPASEEALISFGKNGTIPDEKDSTARCFADCYGKKTTMLTSDGSLNWTTLDFLMRSYDMKPTATETFGKCQKDTSNVECMKSYLSLRCVAETVESLTDIR

>OinfOBP9

MKALLAGCVAALGCLAVAVAAISESMSRAEEAAAKIDLPELFEECNETFTTPKATLNYFFSHGRLQNENDYGSKCFIHCLADRSGEIDSDGNFDVDLIKVMTRRFPNETNIEGLNEMVESCVADRGETDFCERAYGLVSCLIKEKLARLGHSD

>OinfOBP10

MALAVRLFSATLLLASVLFSDISTAGEVFTMSQLKAAVNECNDTYFLSQKNWDSVFTTGSLEDENDLVAKCFFECVLEKTGAMDEKGTINSDITKAVFLASHESTGTAVQGHDELIDMCVPGRDETEICEKGYALVKCVTVEELLRRQARK

>OinfOBP11

MDKASSAAATACLLIAVAALHAQALSLEQLRQTSKIVRNMCLKKTGVDLALVEGIQEGQFPDNQDLKCYMKCCMGAMQVLRQGRYNVDAAKNQAEKMLPPDLKGRFISMLDACSDRGDGADDDCEMAYQLTKCSYETDKEIFLFP

>OinfOBP12

MKLTGRIMDAAKEVDHTCRSSTGVPREMLHRYADGQTVDDDDFKCYLKCIMVEFNSLSDDGVFVLEEELENVPPEIKEEGHRVVHSCKHINHDEACETAYQIHQCYKQSDPELYSLVVRAFDATIGD

>OinfOBP13

MRTSAAATGAALLALAAVASAMEMTPEFMEIVNKCKTEHEPTEDELKGMMALKVPESSNGKCFMGCVLQEIGVVKDGKFDKEEAKKHAAAKMTDKDELEKHMQLIEKCSQEVGGETDSCGIGPKLMECIKQFAPEFDIALPKPSE

>OinfOBP14

MRTLLIYAIVCAIIVKCYCDSAEVSDGSEEDAMMKCAEELGFGHDEIQRIKNSTVPDERNENERCLMKCIGQKMKYLTSEGIVDVDHLLELSGEMIEKEGYSKSEMRQMLEKCAKKTGTETCMTAFKNMRCLMNRSK

>OinfOBP15

MVNHHQRVVAIAAALAAMVAATPSPIAEAIRFSKETVSKCQEKWQVSEEIIEEMQRNKGALPNEDSVEQRCFAECVAKEMGVINNGGGVAVDKIVKILEAVFEMASKETGEKLKLDSRALKRDLEACEFKGEDDECTNSYDTLKCLRTLGTSENMRLYVTKES

>OinfOBP16

MASALCLMAAAHRRHSHRYGIGIKNLNETMDFCNKTYPVSVDTLLERALNNGTLPDETNENARCFIECVERAKGTVNTDGTWNTTRAKQIEIDMNLALGRNISQDISNIVDECSTNSGSGSCMTVYLIKKCIDSRMSAFREPSQHNTHE

>OinfOBP17

MAVTLTAASALWFVAAAFAAVVTTEIPTEDILRWVQTCNKTYPVSQEMLRSLASTGGLLSDESDVNSRCYLECYERLGGTVNSDGKVNVEKAVTVMVSYYPKVAELGVDTVTEILKNCNSKSGTGQCMTSYLIRNCFIEGLNAKSPHTSVFDTSSYYI

>OinfOBP18

MAAAAAHLWCLLPPLLLAVCCALAAPSITSAEMRMDMMVIQHCNETHPVALIDMNKALINKKIEPQNTVFKCFVFCLLNKYEWMDDEGGFLIANMKHNLSDSHLDQLSIDFIVYKCSATGSSDKCERAYRFTECFWGEVTKFPENSDEKYDDPNLFALYQ

>SgreOBP1

MWARLCNCAALLLLLVAAAQAWDVNMKLTGRIMDAAKEVDHKCRGSTGVPREMLHRYADGETVDDDDFKCYLKCIMIEFNSLSEDGVFVLEEELENIPPEIKEEGHRVVHSCKHINHDEACQTAYQIHQCYKQSDPELYSLVVRAFDATI

>SgreOBP2

MASHCHATVAAVVVAAAFAAVVAESPPHMAAFTFTKEMLSTCQEKSEISQEMVDEMQKNKGLLPDESSVAQRCFQECMAKEMGLLKNGGGVAVDNIVTVLKAALQMASEGSEDTYTIDTDAVTRDLEGCQFEGEDDECNNSHDTMKCLRSLGNPENMKRYITKES

>SgreOBP3

MLLAAPAKADEPDLTKAIKDLKDCMASENLDSLDGLKTNKEATTTEEKCFIGCMMKSVHVLNSDGEYDVDFLKEHINHCPELMKDQQKKAAAIEVAESCAAKVTGCSGYCECGVVAGDCMSEGMEAKGYETIYEWLEKVIVKVDA

>SgreOBP4

MKSYFAFVFAAVALFAVAKADPAKLKQAVEKCKASENLDSLDGIKANRQPFTSEEKCFLGCMTLDMKFLSADGQYDAASTKQMINNCEHLKDKPDEKSAALAVADDCGKTVTGCNGYCECGPMTVGCLIKGMMAKGYEESFARIDKVLQKLDG

>SgreOBP5

MDKGTTAALTASLLIVVAAVHTQALSLEQLRQTSKIVRNMCLKKTSVDLALVEGIQEGKFPDDQNLKCYMKCCMGAMQVLRQGRYNVNAAKNQAEKMLPPDLKDRFIAMLDACSDQAVGEDDCEMAYQLTKCSYEADKEIFLFP

>SgreOBP6

MKAHTASLATATVVILLLVAAVVRGQDDDMKEMMEQLHQSCLGESGASDANIDEARKGNFIEDGNLKCYMKCIFVQMTCMSDDGVFDADTAIAMLPDNLKDVASKALTACKDEKGSDACDTAFKINQCLFKQAPKDYILV

>SgreOBP7

MATTVLSPAALLLALLLAAGSVTARRGPRPFGRCASSVGDIDRDTMMKIVRNFEVPDETSDDQKCMLRCALMTNRLVRDGVLDTRQILMNIRADAHFAGRMASVYGQNITLDMDRLSNDVEACVTSEESPDCDTVYNQFKCVSDLISNGNMASYASFVEVDGSETGDWMRRRQMLASRPHGPPGPMGGHWGPPPPHHRGGPRGGGRPPPPPPESDENDVEELE

>SgreOBP8

MRTSAAATGAALLVFVAVVSAMEMTPEFMEIINKCKAEHEPTEDELKGIMMMKVPESEHGKCFMGCVLQEVGVVKDGKFDKEEAKKHAAAKMSDKDELEKHMQLIDKCSQEVDGETDSCGIGPKLMECIKQFAPEFDIALPHAPSE

>SgreOBP10

MESAMKTLLVVCVAALGFLVAAEISESMSRAEEAAAKINLPELFEECNETFPIPKVTLNYFFSHGRLQNENDYVAKCFIHCLTDRSGEIDSEGDFDVDLIKVMTRRFPNETNIEGLSEMVDKCVAGRGETDFCERAYGLVSCLVKEKLARLGHSH

>SgreOBP11

MNTLFTCCVAVWLLIAAALLQPTKGDEMLPNTDIPATMAECNATFKLGWRCWDNLLSDGHVIDESKYQQKCWFYCLLDRTGAMHADGAFDKDLLKMVLQGFPNGPSLAHLNETTYTCVAQRSEVDLCERAYAIVKCIMTEELSRMHHSS

>SgreOBP12

MKWSLWLTATIALVLQLSISEGLKCHTDEDSQNPDEFQEVAAICMKNTSGSELNRSDRENKRNGNNYHKNNFGNTNDNWSSGGMGQTFPGYNSENEGYGLHGSGRCTANNDGYNNNRNNMNQNNMNGMRQKPRNRNRRSGQQSEAANVDLEDIEPCAVHCIFRQMGMLGDDALPDRSAVAKVMLRGVKDTEVKDFVQEAVEDCFDQVESDRKGSKCDLSKNVALCLRQKGRENCEDWGEQEDDQQSNQNKNGNNNGNNSNNSNNQYGNKKWN

>SgreOBP13

MDKCSFHFCLTNTLIYFSLELSSVLPWITRAEVMKRVNVWTASDELRKKLLDALEECIITENEDLNSSLWSPIKGSPPYGGNTWNIGVATANSNKSINQWRSYNEMMGNRTTSVNRDQKVHIDGVYWKNDSDDQDYKNWKESKCFNRGGNHQMQQRCRRSSELPGGNALSSCVDQCLFVKLQVVDKNGLPVEALFMELLDTSIPEQQMRRKARSELHYCFQKMASVAEEDTCTFGKQFASCLDLNVQDIKKHQSNSSNINKLH

>SgreOBP14

MIFSVRFFTVTLLLGAVLFDGICRAEETFSKNQLKAAVNECNDTYFLSQKSWDSVFTTGSLDDEKDLVAKCFFECVLLQTGAMDDKGTINSDVTKAVFLASHDGTAVDGHGELIDMCVPGRVETDTCEKAYALVKCVTVEELSRRQAR

>AglyOBP3

MISSTFYTSLMFGIVMLISCSFGRFTTEQIDHYGKACNATEDDLVVVKSYKVPTSDTGKCLMKCMISKLGLLNDDGSYNKTGMEAGLKKYWSEWSTDTIESINNKCYEEALLVSKDIIATCNYAYVVMACLNKQLDLDKST

>AglyOBP4

MRGNYSLVVFLLFGFGLLEIYCQKQETSGKCRAPDKAPLNLEIIINICQEEIKSALLQEALDILNDGTLEQNTPSYSRSKRDADEDLSNEERRVAGCLLQCVYKKVKAVDETGFPVVDGLMKLYNEGVQDRNYYMATLSAVRHCISIAQQLKQQQPSKSFDDGQTCDLAYEMFECVSEKIEENCGVENKSNNLSQRQV

>AglyOBP5

MKMSTNGATMKCVAIAVVLFQMSVIFAEAGHQRRGKELLDTEDSDFFRCKQASRKSCCGPENAMKRFGDKDKVAADECYAQVAEKFATVTATTPKQDLFSGEAVKITKKKQFCLHECIGKKNKLLTEDGSLNKTFIADYAMKSVFKEQWQKQIGQKALDKCLEETYIPWPAEETENKCNPVYVQFQHCLWLEYESNCPDNKIKLTKKCEKTRNRYRMQKSPSNQ

>AglyOBP6

MQKVVFLCIFAIICQTVFTVGFERTWILRQKRVTNDDECRTLIPSSEKKLPTCCQMPNILPGLDNAWEVCFEKFKQFKDKHATKEYKEMAHGNEPPCLFQCVFMQSGLTTSDGKVNEDAVIKKMAEGMDNDEKWKSIWRNTFNKCLNDVKQEDKEQIKMTNTPTGRLMKCFLRDLYMNRPKNVWVESSECSNLKDLVEKCPKMPPPVFKSPPKLI

>AglyOBP7

MVARKRMYMLPATVLLAVVAATILKDSDAYLSEEAIKKTQKMLKNVCSKKHSVEEEVFTDIKKGIFPENNNNIKCYFACNFRTMQMVNQKGILDKKMFKDKMTMLAPPNVLAILLPPIEQCIGNDKDTEICRSSYNFIKCAHRVDPKSLEFLPL

>AglyOBP9

MIIKKTLLVSGFVLFGCMFSINKAADDADAKDKELMSKLITVAFKCFKDADWGTCGEMITTKYDITQAKYKQCTCHMACAGEDLGLINSNGQPEPAKFLEYVKRINNSVIKSQLQHIYDKCQNVKGTEKCDLAEQFAICAFKESPEMKERVTKLIEMLVKMKPKSK

>AglyOBP10

MEHLRGTNVVFAIVMALLVVQSSTRPQPDELDDIKKTLYNACSEKFPLTEEIKNNVKNSIVIDDQNFKCFLRCCFDEMSLIDEDGIIDGESLAAMAVDKIKPVAEKIVHDCLPAGKQEKQDGCEASFKFFSCGIKLNPLTIELLPLQ

>DponOBP3

MHCSRACLIVFFSICGLSSSLKITLPPELQEYVDDLHKLCLEKGGLTENDHQTYDINHKNEKMMCYMKCLMLESKWMKSGGEIDYDFIETQAYPEVKDLLLNALNKCRTIEEGADLCEKSYNFNKCLYDADPVNWFFV

>DponOBP21

MALTTWVLSIMLILPAIRALSDEMKELAQMLHNTCVAETGVNEDFIQKVNAEKIFADDENLKCYIKCLMAQMACIDDDGIIDEEATIAVLPEEYQALAAPVIRACGTKHGANPCENAWLSHRCYAEMEPSAYMLI

>DponOBP20

MKVFVVLCIVLFAFTLIVSAKKNKSNDEEKAKSYKKVFKECQKKDETRVDASIIRKLKKHKQVDLPANFGEHKLCVFTGIGLLKADNTVDEDKLKKKIASAKPQKDIVDNIVMDCTSSKSTLQETALNLDKCLTTYSIEF

>DponOBP19

MKAMFVTLTVATVVVFASADLTEEQKQKIVANGKACVAETGADPELIKAARQGKFADDAKLKAFALCMSKKSGFQNEAGEIQSDVVKQKLGLAIGDEAAAKKLVEKCLVSKGSGEETAIETFKCYYENTPTHIAVF

>DponOBP18

MNGFSVFFLLLLAAVVKSDFDFSNYKEFENLAGDQREKAIKLFKECMAETGATHEMMEKSVEGDIPDDIVFKNHLVCIGKKSGFIDENGMHIKEKLKEKLTLLLGNEELVDKILDKCFMEKGSPQDTAFELAKCCHREYHN

>DponOBP17

MQVTMNQGWFLLLVSVVSVFAELDQTSLPPEAKELMAALHKNCIEQVGVSEADVDKLRAANFEEDANLKCYTRCLMAESGVMDENGAIDIEAFGEILPEAIRGNIQAIFRSCSLTKNDIVDQCVKAYEMVKCWHKENPESYFMI

>DponOBP16

MKLMWILVLGAALKSTEGAMTEAQMKAALKLIRNVCQPKNKATDAQIAAMHNGDWNQDKNGMCYMNCVLNYYKLQLPDNSFDWETGLKVVESQAPPSMAGFIMETIKSCKDAVKTGDDKCKAALEITKCLYDQNPEKYFLP

>DponOBP15

MGTTIFLLVGLFMMTNAYVPNVNDKIRDFCIDDSGVSIEMVENLLANPEKELIDVESCYVHCIFTEMGLLSENGNVEIENFKSLKASEAPYIDLNCLEEIKSIDHCNEMMILRACHV

>DponOBP13

MSNLLKLSIAFAVVSVISCQDFTEEQRKKIIENRQQCIEETKVNPDLIEKADLGDFAEDQALKCFTKCFYQKAGFVNDKGEVQKDVVEAKLPPQADKKRALEIVDKCALKGKDACETVYLIHKCYFEHTHPEADEKTAKDGKSEEKKA

>DponOBP12

MHFQWLTNVSVFLCILGVAQLVAAGKPNDLFTRITPGDVEVCGKDTGVDRKDFEEAREKGALNHSMLCFLKCAMEKAGFLKDGHLEIDQAKEASPDKMTEPVVECFKAVGPISTCDDIQKVENCLPGS

>DponOBP10

MQLLFVAVLVIALVQVNSLTDKQKELLTQHYNQCVAISKVDQAVLQKARAGDFANDPNLKTHIKCISEKIGFQGTDGKFRRDVIEKKLKETLPGDNAKNAKLIETCVVANKDPQLQAFNAFKCLYTNAKINLL

>DponOBP9

MKSLAVVFAVLIAASLADQVQDIWDRVHQQCQQSPNTHVPQEIFDQLKRGETPILPANFGLHANCMLKKMNLQDNDGHIISSGVKEAAQRHYQSAEKINQIVKDCSATKKTKEETALNLFTCLGQNRVNIG

>DponOBP6

MKSIVALFVCALTATALADAEINETAFKAGRDRIMAMSRTCDENPATAVDQKALKKYLQSNGPAPANGAAHALCITKNLGWQNEDGSVNKPVITEKVKAIFGSVDAKVQQYIEECTEAKATPEDTAEQLLKCYRKHSPKIE

>DponOBP4

MNTALKVFLVALAIPTIMGLSDEMQELANQLHTTCIGETGAAEDAITNARNGDFSEADSFKCYIKCLLSQMAIIDDNDGTIDVDAMVAVLPEEIQEATEPIIRKCGSIIGANPCDSAWLTHKCYYKEGPEHYFLI

>DponOBP2

MKQLVMVVLTALCVVHCKGLECGLSKISSEHFRKIASECVKDNETLNRIWELTSETSMEEDSVSSDEEVPVTKGREAPNFHDLGSSAHRNMKMSGASRTKRSRKGFNNESPMSNVQKKSSPASTTTEHTTTMQSEENEENAAANNVEESGEVCILQCIFEKLEMTDTNGLPDHKKVASALVKSASGRETQDFLQDSVDECFQETEEGDFENSCEYSTKLVTCLAGKGKSNCADWPVGDLPF

>DponOBP1

MLTKTILIWAAILLTVFIPKGNCRLTEKQLAAAIKLVRNMCMGKSKANPEDIEKMHQGNWDVDYQAQCYMWCGFNMYKMLDKENHFDKKAALQQMDQLPIDLQEYVVKCMDQCENAVTNFDDKCVVAFEYSKCLYFCDPEKYFLP

>DponOBP5

MSEKTHFALVALLLTCLVNIIDADQREKAVEFQRDCMEAHGLLEDELHEIMDGKPIQNEAFYFHFFCVVKKAKLISDNGIVNTDHFEENLKGVIDEENMAHVAALTRRCLIQRDDIFTTIKMAIDCFYSSEHKL

>HarmOBP1

MSKFTFFVLCVVAVSLSKVYASDEDKAKLHEALKPLVEECMKDHEVSLDDLKAAKEAKSADGVKPCFLACVYKKAEVLNDKGEFDADHALEKLKEFVSDEDVLAKVAEVGNTCKAVNDKAVSDGDAGCERAALLTACFLEHKAEILV

>HarmOBP5

MSKFTCLVLCVVAASLSQAYASEEEKAAFREAIKPIVEECSKEHGVSHDELKSAKDNQNADSIKPCFLGCVYKKAEVFNSKGEYDVDKALEKLKKFVSNDEAYAKFAEVGKKCASVNDKAVSDGDAGCERGALLTACFLEHKAEVPL

>HarmOBP3

MSKFTCFVLCVLAVSLGEVRSNALEKAAIRAAVYPLIVDCAKEHGVTLEQLKAAKASHSAEGINPCFQSCVYKKTGIFNDNGEYDVANAKTKLQKFVTDEDEYARIAEVGKTCASVNDKSVSDGAAGCERAALLTACFLEHRAQIII

>HarmOBP4

MSKLTCVVFAAVAVVFSNVNADDETRASFRQVLGPLVMECRNEFGITEDDLKKAQQERSPDALKPCFIACVFKKFGIITSAGKYDSDASISRIKDVVKNDDLLAKLKSVGEKCNSVNDASVSDGDAGCERAALLAKCFIENKSELSI

>HarmOBP2

MMDRKRLCLLIIALFLAQGSDAMSRQQLKNSGKMLKKNCMNKNQVTEDQIGSIDKGKFVEDKKVMCYIACIFEMTNVVKNNKLNYDASIKQIDLMYPPDLKESAKAAVEKCKDVQKKYKDICEASYWTAKCMYDFKPEDFIFA

>HarmOBP6

MSKFTCLLLCVVAVSLSKVHATEEEKEAIRAAVRPIMQECGKEHGVTLDDLKAAKAAHSADGIKPCFQSCVYKKAGIFNDNGEYDIANAKTKLQKFVTNDEEYARIAEVGKMCASVNDKPVTDGAAGCDRAALLTACFLEHRAQIII

>HarmOBP13

MFTGTLPLVVFLATFAYGGKEKPVFSDEIKEIIQTVHDECVAKTGVAEEDITNCENGIFKEDPKLKCYMFCLMEEASLVDDDDAVDYDMLVSLIPEEYVDRTTKMIFSCKHLDTPDKDKCQRAFEVHKCSYEKDPDLYFLF

>HarmOBP8

MLLIEIVKFLTLVAMCEAMTMKQIRNTGKMMRKSCQPKNNVADEQIDPIAEGVFNEDKEVKCYMACIMKMANTIKNGKLNYEAAIKQADLLLPDDIKEPAKEAITACRKVADAYKDICDASFHITKCIYTQNPGIFYFP

>HarmOBP7

MFRFGVLSFVVLLFCMESSYALSSEEELSIKEALHPFVVECAEEYGMTEEMFEEAKKKGSAEDIDPCFMSCFLKKTGFFDDSGKFDAEKSISFAKEHITSESAIKFLEAGAGECVKINDEDVSDGENGCDRAKLLFDCLTELKKKMSE

>HarmOBP9

MCKFSVLFLYSAVMAVNIWSASCISEEDKAAIITAIAPLAQNCGSECGLDNDDFEKYKEDGSDMDPCFKACLMTQMGVLDKEGKYDGKGLHKAMEEADYPGDKDDAQKFLDELDRCFDAKGDNSGSDEEAKMKRADVLFRCMQDMKEK

>HarmOBP15

MGSRHVFFALVVLAVSVKKEKPSKHPMPYITSRFVKVLEECQHELKLNEHILEHLFHFWKLEYSLLGKDPGCAIICMSTKLDLLDLYGRMHRGNAAEFAKKHAAGDEVPSKIVTIIHFCQKKHEQDGDECLQVLEVATCCRTGLHDLNWQHQVEVIVPDVLTEI

>HarmOBP17

MKTFVILAACVMLVQASGLTDEQKEKLKKHRSECLTETKVDEQLVNKLKGGDYKTESEPLKKYALCMMMKSELMTKDGKFKKDVALAKVPNAADKPTVEKLIDACLANKGNTPHQTAWNYVKCYHEKDPKHAIFL

>HarmOBP16

MFKSIVFCALIIVASHADVLKKRDSKGASLKPLSVCCDIPELGDPKNLEKCSNPKMPGPCDDIQCIFEASGFLIDRNTLNADAYKNHLMKWQEEHKPWKVAVDRAIEECANNQTRQYLDFPCKAYDVFTCTGIAMLKKCPEAAWKC

>HarmOBP18

MKSFVVFCVLVAGAFAANVSLPPKQNEKANQIATECMKESGLKPEVLAEAKKGHISDDEHLKKFTFCFFKKAGIVSEDGKLNTEVALAKLPPGVDKAEAEKLLETCKGKTGKDVTDTVFEIFKCYHHGTKTHILLGF

Table S2 Amino acid sequences of 87 CSPs of *Ceracris nigricornis* and other insect species used to construct phylogenetic tree.

>CnigCSP1

MLLCRHSAAAATVAVFAAAACMVAVTSAAPPKCASIAANDKKYTTRYDSIDIDSILKSDRLLRSYMDCLMDRGPCTQEGCLLRAAIPDALQTECSKCSDVQKKQAGRVMAWILENKRNYWDELIAKYDPEGNFRKKYGYDEDDEEDEK

>CnigCSP2

MSPLARTVLFLSFLAVLAAASAQEGYHTAYDDVDVELIIQNDTLVQTIMKCLVSATDDLCDPGNKHVKELLPEMLATACGKCTDKQKHSMTKFFGQLSRKHPDLFKQFQEKFDPTGELLGKIKTTA

>CnigCSP3

MRTSQTLCAAAAAAATLLLLVAVTEAAAPQEALYAKYENLDVDRMLRNQRFVAATIKCLMDEGPCTPEARDLKKVLPDALKSDCSKCSAKQKENVRKVFEFMMKERNADWQRLSRKYDPDGEHKKRLEAKLKEAQQQQQQQKKTTAEAPAPAA

>CnigCSP4

MAAKLILCCVLGLLALCADAAEDPLDSFNVDEVLGNERLLKSYIQCMLDDGEGRCTKEGKEIKKRLPQFVATGCLECPPKQLQRAIKTLKHVTEEHADDWAKLKAKYDPTGEYAKKHADTWKTRGINF

>CnigCSP5

MARLSFALSLLSLLAALAAAQDKYPDTFDKLDMQALLANKDRIEAAITCLKGDSNSSCTPTAAFLKSVLAEVVRTDCGKCTETQKKKVAAFFAHVSQHYPQQMQELLDKYDPTKEYRAKYAQSWAADGIKV

>CnigCSP6

MKAALAVVAFLAVVALVAADEGYTTKFDDVDVDKIIHNERLMHQYELCLTEDESKCTPEGKELRKDIPDALETECSKCNEKQKEGIRKVIKFLINHKPETWQKLKDHYDKDGKYSDKYKKMEEELKE

>CnigCSP7

HTYKTPRSKKLAAGRARRSSAAVRMRALLLLVAFAAAVAADASMRGKTLAEFANTDWAAYLGNRPAIERHIKCALDKMPCDESGLKLKQLVPLMVVSRCDKCTPMQRRNAETVRQFLNAFYPGVWESLEEKYKG

>CnigCSP8

MRARPVLRGTSAELEAGVRAGAAEAAGLDVDVDAVLGSERELTALLRCVVARGDGPCNDQGKAVKAFVASAVETNCASCSEQQQLAAEKVLRHLTTKRKSDWNKLVSRYDPTGELRSRYRKHWEQRGVRI

>CnigCSP9

MASDRLLNSYFQCLMADTEERCTADAKYLKEVIPDALTNGCSRCRRNQQEGADKVITFLVKNKPDMWNKLEAKYDPEGVYRSKYQKEYQKVKEGQGMSERMSTGKDNEPYSSKYDNVNLDEVLKSDRLLNNYFHCLMDATEERCTADAKYLKEVIPDALTNGCSRCRENQKDGADKVIRFLMNNKPDMWNKLEAKYDPDGTYRKKYQNEYNMAKSGQDNEPYSSKYDNVNL

>CnigCSP10

MQTLTLVLVAVAALVASAAAYTTKYDNIDVDDILNNERLLKKYHECLISDSDSSCTPDGKELKVTIPDALVTDCAKCNEKQKEGATKVIKFLVQKKEDLWKPLQAKYDPEGTYLKKHPELLEAE

>LmigCSP1

MQKCTLALLLACLVAAAAAYTTKYDNIDLDDVLHNDRLLKKYHECLLSDSDASCTPDGKELKAAIPDALTNECAQCNEKQKAGAEKVIRFLIKEKPDLWTPLEKKYDPTGSFRQKYDQELKRVSA

>LmigCSP2

MKSCALALLLVGLVAAAAAYTTKYDNIDLDEILHNDRLLNKYHECLLSDTDTPCTADGKELKAAIPDALTNECAKCNEKQKNGAEKVIRFLIKEKPDLWTPLEKKYDPNGTYRQKYGEELKKVSS

>LmigCSP3

MQALTLVLFALVASAAAYTTKYDNIDLDEILNNERLLKKYHECLMSDSDASCTPDGKELKVSIPDALVTDCSKCNEKQKEGSNKVIRFLIQKKEDLWKPLQAKYDPEGTYLKKHPELLSA

>LmigCSP4

KYDNIDLDDILHNDRLLKKYHECLVSSSDASCTPDGKELKAVIPDALTNECAKCNEKQKAGAEKVIKFLVKEKPDLWEPLEKKYDPSGSFRQKYGPELKKVSA

>LmigCSP5

AAAYTTKYDNIDLDDVLHNDRLLKKYHECLLSDSDASCTPDGKELKXAIPDALTNECSKCNEKQKAGAEKVIRFLIKEKPDLWTPLENKYDPSGSYRQKYGQELKRVSA

>LmigCSP6

AAAYTTKYDNIDLDEILHNDRLLKKYHECLLADDDASCTPDGKELKAAIPDALTNECAQCNEKQKNGAEKVIRFLIKEKPDLWTPLENKYDPSGSYRQKYDQELKRVSA

>LmigCSP7

AAAYTTKYDNIDLDEILHNDRLLNKYHECLLADDDASCTPDGKELKAAIPDALTNECSKCNEKQKNGAEKVFRFLIKEKPDLWTPLETKYDPSGSYRQKYADELKRVSA

>LmigCSP8

AAAYTTKYDNIDLDEILNNERLLKKYYECLMSDSDASCTPDGKELKVSIPDALVTDCSKCNEKQKEGSNKVIRFLIQKKEDLWKPLQAKYDPEGTYLKKHPELLSANKNSI

>LmigCSP9

EEKYTTKYDNVNLDEILANDRLFDKYAQCLLEEEDNNCTADGKELKRLIPDALSNECAKCNDKQKEGTKKVLRHLINNKPDVWQQLKAKYDPDGTYTKKYEDREKELHQ

>LmigCSP10

EEKYTTKYDNVNLDEILANDRLFDKYAQCLLEDGESNCTADGKELKKAVPDALSNECAKCNEKQKEGTKKVLKHLINHKPDVWQKLKAKYDPDGTYSKKYEDREKELHQ

>OasiCSP20

MQKCSLALLLACLVAAAAAYTTKYDNIDLDEILNNERLLKKYHECLVSDSDSSCTPDGKELKATIPDALVTDCSKCSEKQKEGANKVIKFLIQKKEDLWKSLQAKYDP

>OasiCSP19

YDNIDLDDVLHNDRLLNKYHECLLSDSDASCTPDGKELKAAIPDALTNECAKCNEKQKAGAEKVIRFLIKEKPDLWTPLENKYDPTGSYRQKYGDELKRVSA

>OasiCSP18

MSEGAEGTAACVGSCRDVTAVYIAAAGGCGRRLSRHSLPTPHRTLANMKSCAFALLLVGLVAAAAAYTTKYDNIDLDEILHNDRLLNKYHECLLADTDTSCTADGKELKAAIPDALTNECAKCNEKQKAGAEKVIRFLIKEKPDLWTPLENKYDPTGSYRQKYGDELKRVSA

>OasiCSP17

MSEGAEGTAACVGSCRDVTAVYIAAAGGCGRRLSRHSLPTPHRTLANMKSCAFALLLVGLVAAAAAYTTKYDNIDLDEILHNDRLLQNYYECLMKDDETGCTPDGVELKRTIPDALKTECSKCNEKQKEGTKKVLKFLINHKPDMWGNLKAKYDPDGTYAKKWEDKEKELHE

>OasiCSP15

MARSSLLLLLALVALAAAENPLISQLENIDVDAVLADPQRVDAAVKCFLSDADDDCNVRSKVIKSLISEMLKTNCAECSEKQKAGVAKFMAHIAKNKPEEMKQLLAKYDPSGEARAKYGDSWRQKGIIP

>OasiCSP14

MHRHSAAAVAAIFVVTACMVTVTSAAPGKCASIVANEKKYTTRYDNIDIESILKSERLLRSYFDCLMDRGACTQEGCLLRAAIPDALQTDCSKCSDVQKKQAGHVMAWILENKRNYWDELIAKYDPEGNFRKRYGYDEDDDEEEK

>OasiCSP13

MSRVARVVVCLCLAAASAQEGYQTAYDSVDVDVIIQDDTLVQSIMKCLVSATDDLCGPENKHVKGLLPEMLATGCAKCSEKQKHSMNKFFGHVARKHPDLYKQFLEKYDPTGELLSKIKTTA

>OasiCSP12

MQTPTLALLIVTAALAAAAAADDRYAKYDHVDVERMLRNQRFVNAAIKCLLEEGPCTPEIRDLKKMLPDALKSDCSKCSAKQKENVRKVVDFMMKQRAADWARLSRKYDPEGLHQKRIEAKLREQQQQQQQ

>OasiCSP11

MKASALLLLLLLTAACVVAAAPEEKYQSPYEGTDVDAILQDDAKVQAILKCLLSDADDVCSKEDKQSKDMLPEALATQCAKCTEKQKHGMARFFAHVSQKFPDLFKQLAAKYDPTGENLAKFSAARRLSA

>OasiCSP10

MAAARQLLVLAAVVAAAAAQFGGTDPGSLLADPQALTQVIRCLLASADDGCSLQGRLLKSVLPKLLQTNCAQCSEAQRQDVAGVLRHLVNDRPEDWQRLADKYDPEGTLRRQHGDEWRARGVNL

>OasiCSP9

MSRLCFALSLLSLLAALAAAQDKYPDTFDKLDLQELLGDKERVQAAIKCLLEAADTECRPAGKLLKSVLAEIVKTDCGKCTETQKQKVAGFFSFVSQNYPEQMQQLLDKYDPTKEYRTKYAQSWAADGIKV

>OasiCSP8

MKAILVLTAVLAVVASVAADDKYTTKFDDIDIDKILANERLLHQYELCLTEDETKCTPEGKELRKDIPDALETECAKCNEKQKEGIKKVIKFLINHKPETWQKLKEHYDKDGKYSEKYKKLEDELKE

>OasiCSP7

MRNSCLAVALLTTVAVVCGGYTTKYDNFDVDQVLHNDRLLKRYHECLVSDSDAACTVEGKELKSVIPDALQTDCSQCNEKQKAQAEKVISFLIHNKPDLWQSLQNKYDPDGSYRKRHDAELKKLSS

>OasiCSP6

MKAALLLVSALAAIAMAAAEEKYTTKYDNVNLDDILANDRLFNSYAQCLLDDGEDRCTADGKELKKIIPDALSTECSKCNEKQKEGAKKVLKHLINHKQDVWQQLKAKYDPDGTYSKKYEARERELHE

>OasiCSP5

MKASLVLLSALAAIALAAADDKYTTKYDNVNLDEILANDRLFDKYAQCLLEEGDSNCTADGKELKRVIPDALSNECAKCNDKQKEGTKKVLKHLVNNKPDVWQQLKAKYDPDGTYTKKYEDREKELHQ

>OasiCSP4

MKAALVLLSALAVVAVVAAEEKYTTKYDNVNLDEILANDRLFDKYAQCLLEEGDSNCTADGKELKRVIPDALSNECAKCNDKQKEGTKKVLKHLVNNKPDVWQQLKAKYDPDGTYTKKYEDREKELHQ

>OasiCSP3

MASNLLVYCCLFAVVAVWVTAEEDKLDAINVDEVLGNDRLVHSYLECVMDDNDSKCTKEGREVKSRLPGLVKTGCNDCTPKQLERAIKTLKHITEKHPEEWKKLKAKYDPTGEYTQKYAETWKQRGVDF

>OasiCSP2

MAGKLTVCCLLGLLALCVEAAPQDPLDSFNVDEVLSNERLLKSYIQCMLDDGEGRCTKEGKEIKKRLPQFVATGCLECTPSQLDRAIKTLKHVTEEHAEDWARLKAKYDPTGEYSRKHADTWKQRGINF

>OasiCSP1

MAIALSARALSYTRRSDMQALTLALFALVASAAAYTTKYDNIDLDEILNNERLLKKYHECLVSDSDSSCTPDGKELKATIPDALVTDCSKCSEKQKEGANKVIKFLIQKKEDLWKPLQAKYDPEGTYLKKHPELLSA

>OinfCSP23

MRACRGLLLLAAYAAAVVTADASMRGKTLVEFANTDWAAYLGDRPAIERHIKCVLDKMPCDESGLKLKSLLPFMMDQNCRKCSNIEKLFTRKLKRMMAKHYPEAYRDMALLARHLQREAAAAAITTTTPITTTVADIDGDTTTVSSSTALSCVCRCRSLYRRRHRHRTATASGRSRQQRDLAFSQPSTAPLR

>OinfCSP22

MAALSLRLALVAAAAVCLASAQQQQQQQRPRVSDQQLDRALADKRYLTRQLKCALGEAPCDPVGRRLKTLAPLVLRGACPTCTPEETRQIQRTLSHIQRNYPKEWSKLVKQYAGF

>OinfCSP20

MRNSCLAVALLATVAVVCGGYTTRYDDFDVDQVLNNDRLLKRYHECLVSDSDAACTVEGKELKSVIPDALQTDCSQCNEKQKSQTEKVLSFLIHNKPDLWRSLQDKYDPDGSYRKRHDAELKKLSS

>OinfCSP19

MKAALVLIAALAVVVVAAAEEKYTTKYDNVNLDEILNNDRLLNKYVQCLMEDGESNCTADGKELKKAVPDALSNECAKCNDKQKEGTKKVLKHLVNHKPDVWQQLKAKYDPDGTYTKKYEDREKELHQ

>OinfCSP18

MAHLQAALCTVATLAALLVAVCAQGGPAQSFARPERLDVDSAIHNETRFQEAMACILGEDDYRLCDHHTVGIKYDGWRMLERGCGGTCTPDEQSVYRFLAYVSHNKPDQWRRILDKFDPDGKLQQSNSEQWRQHGIKV

>OinfCSP17

YDNVNLDEVLRSDRLLNNYFQCLMDETDERCTADAKYLKEVVPDALNNGCSRCRPNQREGAEKVIKFLMNNKPDMWNKLEAKYDPDG

>OinfCSP16

MRKALAPLLILVIAARALPAAADDGKYTTKYDNVDLDEILNNERLFNKYMDCVLDDGNDRCTADAKDLKELLPEMVQTDCAKCSDKQKAKVDKVVKFVRDNKKDAWE

>OinfCSP15

YDNVNLDEVLASERLLNSYYRCLMENTDEHCTADAKYLKEVVPDALSNGCSRCRPNQREGAEKVIKFLMNNKPDMWNKLEAKYDPDG

>OinfCSP14

GKLTVCCLLGLLAVFVQAAPQDPLDSFNVDEVLSNERLLKSYIQCMLDDGEGRCTKEGKEIKKRLPQFVASGCLECTPSQLDRAVKTLKHVTQEHAEDWA

>OinfCSP13

MFRCVFAVLAALVALAATQSQIENIDIDAVLADPAKVDAVVSCFLNDDYQGCNERSKFIKGLIAETVKTNCGSCSDGQKAGVVKFLVHISRNKPESMKQLLAKYDPNGEALVKYGDIWRQNGISV

>OinfCSP12

MVQMEPHFRDATMGRLCFALSLLSLLAALAAAQDKYPDTFDKLDLQELLGDKERVQAAIKCLKEEADTECRPAGKLLKSVLAEIVKTDCAKCTETQKQKVAGFFSFVSQNYPEQMQQLLDKYDPSKEYRTKYAQSWAADGIKV

>OinfCSP11

MSRLLLLVASAALLAAAWVRPAAASFAAPAVLDVDSPTQSEAAFQDTMRCVFGDNDYEVCDSNSVSIKYIMWRLLEKGCNSCTDDELKVFRFMAYLSNNRQNDWQQVLNKYDPQGEFRSRNAAQWKQHGVAV

>OinfCSP10

MAGLRLRLALVLVGCVLLGGAQAQRRGRGRAGGAAAASHDLDVDAVLADERTLAALLRCVVSRGDGPCSEQGKAVKAFVVSAVETNCASCTEQQQAAAEKVVRHLTTRRKSDWNKLVARYDPTGELRSRYRKHWEQRGVRI

>OinfCSP9

MKAILVLTAVLAVVASVAADEKYTTKFDDIDIDKILANDRLLRQYELCLTEDETKCPPEGKELRKDIPDALETECAKCNDKQKEGIKKVIKFLINHKPETWQKLKEHYDKDGKYSEKYKKLEDELKE

>OinfCSP8

MSRVARVVICLGVAAVLATASAQEGYQTAYDSVDVDVIIQDDTLVQSIMKCLVSATDDLCGPENKHVKGLLPEMLATGCAKCTEKQKHSMTKFFGHVARKHTDLYKQFLEKYDPTGELLSKIKTTA

>OinfCSP7

MHRHSAEAVAAIFVVTACMVTVTSAAPGKCASIVANEKKYTTRYDNIDIESILKSERLLRSYFDCLMDRGTCTQEGCLLRAAIPDALQTDCSKCSDVQKKQAGRVMAWILENKRNYWDELIAKYDPEGNFRKKYGYDEDDDEEEK

>OinfCSP6

MASNLLVYCCLFAVVAVWVTAEEDKLDAINVDEVLANDRLVHSYLECVMGDNESKCTKEGRELKSRLPGLVKTGCSDCTPKQLDRAVKTLKHITEKHPKEWKELKAKYDPTGEYTQKFAETWKQRGVNF

>OinfCSP5

MVRPLVALLLLGALVAAQDKYTTKFDSVNLDDILNNDRLLNKYAQCLLDADDRNCTPDAKELKKAIPDALTNECAKCSEKQKEGAQKVVDFLIDNRPAQWKELEAKYDPSGEYRKRYDDRIKKLKS

>OinfCSP4

MKATLAVGVASLALATVLAFGAHFQLLDRIEGDGDEVAIQCMLSDDDEGCTEPLAELKGKLWGVVSTNCGACSDDEREYIIDYFSGLFRRNPHSRKPLQSKYDPTGEYRRKYGKIWRKKGIELD

>OinfCSP3

MRATVTALVAALAAAAVLASGGRFKHLHRLRGDGDQAAIRCLLSDANDGCTEALTELKGNLHIVASSNCGVCTEDERENVIGYFKDLFIRNPKWAQKIQDKYDPTGEFRRKHGAVWRKKGIPVYDLATV

>OinfCSP2

MAKVLAALLVLVTIAAVPLLTAADDGQYSTKYDNLDLDEIFNNERLFNKYVECLLDDGKERCTPEGVELKAMLPELLQTDCAKCHARQKARMDKIITFIRDNKKDVWEKLTAKYDP

>OinfCSP1

MARALALCCLLALFALAADGAPQDRLDNVNVDEVLSNRRLLKTFVQCILDEGDGRCTKEGKDLKQSIPRLVETGCSDCSARQLENGVKVLKHLTENYPQEWAQMKAKYDPT

>AgamCSP1

MKLFVVVALALVAAVAAQDKYTSKYDNINVDEILKSDRLFGNYYKCLLDQGRCTPDGNELKRILPDALQTNCEKCSEKQRDGAIKVINYLIQNRKDQWDVLQKKFDPENKYLEKYRGQAQKEGIKLD

>AgamCSP2

MKLFVAIAFALLALAAAQEQYTTKYDGIDLDEILKSDRLFNNYFKCLMDEGRCTPDGNELKKILPEALQTNCEKCSEKQRSGAIKVINYVIENRKEQWDALQKKYDPENLYVEKYREEAKKEGIKLE

>AgamCSP3

MKFFVVVALALVAAVAAQDKYTTKYDGVDLDEILKSDRLFNNYYKCLMDTGRCTPDGNELKRILPDALKTDCAKCSEKQKSGTEKVINYLIDNRKDQWENLQKKYDPENIYVNKYREDAKKKGINL

>AgamCSP5

MRKVWLLASVVLAFLDFVKSQEVARTLYSTRYDNLDIDTILASNRLVTNYVDCLLSRKPCPPEGKDLKRILPEALRTKCARCSPIQKENALKIITRLYYDYPDQYRALRERWDPSGEYHRRFEEYLRGLQFNQIGGSNGGSGVGNTVLSNL

>AgamCSP6

MKHLTMVAIFAMVVVLASAQKYTDKFDNIDVDRVLSNDRILNNYLKCLLDKGPCTQEGRELKKTLPDALKTNCEKCSEKQRTSSRKVIAHLEERKPQEWKKLLDKYDPEGIYKSKFEKINKRS

>AgamCSP7

MLSAAVIVVMAALVIVGPQPAAANDSQNINRLLNNQVIVSRQIMCVLEKSPCDQLGRQLKAALPEVIQRNCRNCSPQQAQNAQKLTNFLQTRYPEVWAMLIRKYGAV

>AgamCSP8

MLHNLFLSLSLYVSVCGDPSGSTCAAEATTARTQVSDEALDKALSDKRYLMRQLKCALGEVACDPVGKRLKSLAPFVLRGACPQCTPAEMNQIKKTLAHLQRNFPSEWNKLVQTYAG

>DponCSP8

MKIFIVVCCAFIGLVLADTPKYTTKYDNVDLEEIIKSDRLMKNYVNCLLEKGKCTPDGAELKRVLPDALHTECSKCSDSQKKGSRKIMRHLIDNKPEWWTELENKYDKEGAYKKQYREELKKDGIKL

>DponCSP11

MAPFPQSWLQFGALLLLLALVQGQILNGNVYVEKQLLCALDRAPCDNLGRQIKDALPEIIGKNCKACDNKQLSNAKRIARFVQNKYPNVWNDLVRKYGNPTN

>DponCSP6

MKTIIFLVVVASFYGLSSCKPQEKYTTKYDNIDLDAIIRNDRLLRNYIDCVLGKKKCTKDGEELKVHLPDALQSDCSKCSEAQRNGSRKIITHLLKNKRGWFNELQAKYDPAGNYLSKYSEELRKEGIVI

>DponCSP1

MKVVLLLVVVVGVAFGEEYTSKFDNVDLDQILSSDRLLRNYINCLLEKGKCTPDGTELKKNLPDALENECSKCTPKQRDGAKKVIRYLIENKRDYWDEVAAKYDPEGTYYKKYQEQAKKENIKL

>DponCSP3

MWKLVLLGSLLICIGQTLAEVTEKSQYTTKYDNVDINEVVHNERLLKNYVNCLLDRGPCSPDGLELKKNMPDAIETDCSKCSDKQREGLEAMMRFLIDNKPEYWNPLQEKYDPTGSYKKRYLDAKRAEVAIQPAEKTP

>DponCSP4

MLLIISVLIGMALDLTDAKPAAKNYASKYDHIDVGAILNNRRMVNYYSACLLSQGACPPEGVELKRILPEALQTNCARCSEKQATIALMAIKRLKKEYPKIWSELSAKWDPSDSFVKKFETTFESLHGPGRRVESTTSAGNKLDPSEADGNTIDANITQTSPEGSDRVNQPDTTTTPQIITTNPSFSTSTKPAFSSTKRPSPIPGLVPFNTFFTNPPIPIRPIVNLNLGGNIGATVKAIKQVEKMVADIALEKIGIIRSILRPWRKAKKTRYA

>DponCSP2

MKVVLLLVVVVGVAYGEEYTSKFDNVDLDQILSSDRLLGNYMNCLLDKGKCTPDGTELKKNLPDALENDCSKCSAKQRDGAKKVIRYLIDNKRDYWDQVAAKYDPEGKYYKKYQEEAKKENIKL

>DponCSP5

MQCLGLFVVLVLGCSLVAAQSPYTSKYDNVDVDKILKNERVLTNYIKCLMEEGPCTPEGRELRKTLPDALASGCSKCNEKQKDTTEKVIRHLMDKRTKDWDRLSKKYDPQGVYKQRFEKELSARKLA

>HarmCSP14

MNSAIVLCVVALAGMVLARPDGDGDKYTSRWDDVDLDEILENDHLLIPYIKCSLDEGKCAPDAKELKEHIQEALETGCAKCTDKQKEGTRRVIAHLIKKKLQEWEKLKAKYDPEGKYAKKYEKELEEVKNA

>HarmCSP13

MKVLLVLCLFAAAALADDKYTDKYDNINLDEILENKRLLLAYVNCVMERGKCSPEGKELKEHLQDAIETGRSKCTEAQEKGAYKVIEHLIKNELDIWRELAAKYDPKGDWRKKYEDRARANGIQIPE

>HarmCSP12

MNSAIVLCVVALAGMVLARPDGDGDKYTSKWDNIDLDEILGNDRLLVPYIKCALDEGKCAPDAKELKEHILEALETGCDKCTDKQKEGTHRVIAHLIKYKLEEWEKLRAKYDPEGKYAKKYEKELEELKRA

>HarmCSP11

MNSAIVLCVVALAGMVLARPDGGTYTTKYDNVDLDEILANDRLLIPYIKCLLDEGKCAPDAKELKEHIREALENGCAKCTDKQKEGTRRVIAHLIKHKNADWQKLKAKYDPEGKYTHKYEKELEEVQH

>HarmCSP10

MKVLVVLSCLIVAAFAADKYNAKYDNFDVDTLITNDRLLKAYINCFLDKGRCTPEGSDFKKTLPEAIETTCGKCTDKQKNNIRKVIKAIQQKHPKEWDALVKKNDPSGKHRANFDKFIQGSR

>HarmCSP9

MNSLIVFCVLSLAALTIARPDGATYTDKYDNVDLDEILGNRRLMVPYIKCMLDQGKCAPDAKELKEHIKEALENECGKCTEAQKKGTRRVIGHLINHEADFWNELAAKYDPERKYTTKYEKELKEVEA

>HarmCSP8

MKCIYVLSFLLALAAVQAEDKYSTENDNLDIDAVVANVDTLTSFVACFVDQEPCDAVAADFKKDIQEAVTTRCAKCTDAQKHIFYKFILGLKEELPRGYEEFGRKYDPENKHFSALENAVSPA

>HarmCSP18

RPDTYTDKYDNVDLDEVLSNRRLLVPYVHCLLEQGKCAPDAKELKEHIREALENACGKCTDAQQSGTRRVIGHLINKEPEFWKQLNAKYDPNNKYTKKYEKELKEVQEDKQNH

>HarmCSP15

RPESQYTNKYDNVNLDEILVNKRLLVPYIKCALDQGKCSPDGRELKSHIREALENYCAKCTPVQQDGTRRVIAHLINHEPDYWRQLSVKYDRDGKFAVKYEKELRTIA

>HarmCSP16

MKILVLLLAAVVTAQYEEDTYGTDHDDLDIVALVEDKDQFNSFIDCFIDEAPCDDVAETFKSVIPEAVLEVCAKCTPAQKHIVRVFNESFKKKMPEKFQKFKNKYDPEGKYFENFEAAVGAF

>HarmCSP17

ASTYTDKWDNINVDEILESQRLLKAYVDCLLDRGRCTPDGKALKETLPDALENECSKCTDKQKSGSDKVIRHLVNKRPEMWKELSAKYDPNNIYQDRYKDKIEAVKGQ

Table S3 Amino acid sequences of 293 ORs of *Ceracris nigricornis* and other insect species used to construct phylogenetic tree.

>AlinOR1

MSINDYKPGEIFQNNLKPMKKLRPLKKVSYQQPDKRGTYKKVAKRLSEEEALKKGFNDNQGLYLVLGTLYRDSFGSWVHTIVFIIACLFMLFCLGRQTLLITDDLSLLFETIHYITIIGGVLVIVPPMMKNQFRFQKIFKIFAREVYCYDYLDEETAQEILRLRAEGNKEKQLLTKAFTVMLLGTFAGFSVLLPGMYIINGQFFAPQREDGVIMGIPCVIWFPFRVDDKWVVTVRILLLALEEYASFTVVAFIIGQQTTAICIGHTLLYEFKVLALTMNKFEQRAKLMDKKFIGDGTLKPASQTRKYITSCLNESIKHHDVLLDVSEQYSSIFYVPELVILLSSTMVICLSAVSLTSDNIPLEAKAVSVIFTGAEMMNVFVNCYYGQILLDAHNIIGDAMYESNWTSYSSIVHQHVLIILSRVQKPLSLTAGGFAAVNLDTFAQVVKSSFSYFSLLQALKE

>AlinOR2

MTVDELTLHDMVGFPLWIQNVLYMKITGHWVGAVPGPTPLRVNILRAVGGFPVFLVLLYVAGANINGMVHNSDMTDISMNLIVLSTTVSALHKYSVFTNQQQALGRLGRWVKSVAAERKANNVPDTYADRVLKKALKAFYISGNVASPILIVKMMLTGNTFNVNPGVENFPKPFLMFLIAVSFQAIAWEAVVDGCILMNSLFVFRSELVRFALEWEKLNFDPHNPEISRRQLKAMVKKHVMLLGVKKDLKEYNNSMFGYQVFAAVFTTCALIYGCAKDTKFLGQAVIQVLPTSTASLLTFSILCWSGEEVTYLFQQIHRNIYMTNWFEAPREDKKSIIVILEFAKNPIIFTGFTVFTCTLTTFVETMKQSFSLYTILKAVL

>AlinOR3

MTLKSYIKETLKWDEPLGLITTIAVVAGAFNTIAPPKRIRRFIYWLSWYQTISYILFLMSAGTNIFTSTDFFDECLESLHFLVTAFHVFMKYLTLRFRERDFLELFDHIKRVWSSYRIHNEHFLTSKLSSVNITSVLIFTSIFNVVFVNVGAAYLKNILDPTKVHLPIQIWIPSFTKSSFLVGTTIQVVLFTWPLFIVAMSTTFLNSISSHVEALGLALAEDIGREKVWSRDVARDFYKKHQDVISIVLRVNALMAGNWGFEMICASVQLTLPAYRTLRAFRMNDVEVFNHAVILCLNMMVIYMIFSSGNRILSMGEKIHTKVYESNWFELPVKERKNVLFMLFRTTVPVEYRYKIIHFDLPGFTKVVNTVFSYMALLRFLDSGGSEDEGALM

>AlinOR4

MGYVSSKFKSSVQEWHSWEDEYSVEAMRLRYRGFHRIGFLVLDLSPKYALLSVIMCVIAAAVLFIVSFCLTFSCYQMSDDFEDCSGVCNLGFLCVLAFSFLLNHNFYRKKILDLHHMLGKGFHDYQEPQYFPDELEKFKKVVTKQNVALIILASYVALIGFLVVVVCPLIDESLGFGWTEPYDENGVNRQLPVPIWLPYPSHEGFLHWFSFLFLEGFGGAMICLSIGGTALLFTCLSGGLMLEQKLLVLSIKSIEKRAKRRYRELHKGKPGIDEDGNKIALNDDNKYQECIGYCLRQNILHHHKILTYTNHYLDLARSPLLFAFLVETMAIAMSMVKLNEGSNKWGANIAFACIAIAEVANMIMLCVLGELVTSGSIEINDELYYTKWYTFNKSNKKVLLQFLLETRNPVVLAALGLVVCNMDQFSSVMHTAYSFFNMVKLSKLREETVTMGANT

>AlinOR5

MPPNQLLNTSKHEHDVYKRLDKLYYYGMRLLLLGITPHKFFGKCYFKGAILYVTVILLYILYGLGELLWAIIIPGGILERLSHAYVCSYCASYGVIWVYLMVKLETIHQNRIDFKSFNCSRLLGNTEVESILQKNINYFVKTLFAASLLAGANVTSYILGFVVELIVQYAETGTLEEICVLSCFPFPLWGQMIIAAANLVTLFMCFSIIMSMYIITGLLSLEIETQCEILTRTMAYDEVNDDFKTFVIDHIRLIKQTKWVVRLFENINNSLFFSSYVCLAMQMFSLSVIKPEGYYYLGVFSDFCVEFIVMASQCWLSSAVTNSVLSISEGVYNTPWYRKNKSNAIDVILMTQMAQRPYIQRVFLGTMKIEKETMIQVIQQSYSFYALLMILQSKK

>AlinOR6

MTDEHLAGVSRIYRDALGFSQLDVFLDAKPPQNGRFSWHIKRIVAQFFVCFLAPSFISLQICGVLTAESQNLKQLSFDLGFLSHNVQNFVKMTYWLTHLKSVRSLCIDVSTFNVNKYRPILSSWVLKKETDVTRKFMNRCFLISYGNLIFWVALPTIVSICNYFRYVAGVTEGQDSYIPRLSPTRFPVDMSSLRNRLLVGFFEYGLITMGFVYFQPIDMFFSSIVNMVRTQFFILNSSLFEMPADLEEFWGSRIPVQDTQPPMDLRLFVEDHQRLVRYGVQLRKFLNPVLGMVTVDCFNIMCSLLIVITEILEGDMNFTALLELISGLLVILSSLVVFYTYTSTSGMLKEAEESVFEALYAHKWYGKNDEHKKNVIFMQIRTESANKIPMFHIGDVGRDTFIEGLRMCYTYYNFLKQFK

>AlinOR8

MSLRTNAADGKNHLLDLRDVEGLTMGLNTFGIKTFWHILDYFHTTGKRHWLMTTYIVMYHLIGGTYCLLGFAAVFFIDQEDIPRMAAAIMNPLIATQAIFKCWTFSYSTAEYLKLFVLLKKNFMSCVPPEKKLAVDEVTKKNIGLTNQFVKYAMRWNCFTLSMVSFMPYLRSQAFREFFHLGVGPIVPNKVFENEYPFEWNSSPTYEIIWFYEQICAFLAVVTSSAYQAILLYFVMAIVGHLKVLGFVMGNMKATDFTSDSNETMDETAKAKSYKQLVLCIRDHGKINEAGDLLAERYNTFLTFHIGIAIVVGIIAIFNCTVATELADKIKFGIMCVYGLLEVAIYCFCGQLLENACDDVLRQVYSCEWERMDPKFRKAAQLMMVRANSPICLRAGRLYRVNLETLEAIQQLVYTSLTMLTSMVQ

>AlinOR9

MEHADKHAFERMLRREFWILTVIGGTYGFFHEKAWAVVSTINYVVLYSIMALTMSILAYTVYLLQGQLGYLSQALNIFIVGMVVVTATLTLTLSRPKMQAFLAFYDDPWSFCEYSRNEYFENLMLHTVKKKNKLILTWAFLYGLCGAVGVCQPVIDKIFGRSSEITNVNGAWLNLPIIFWWPFDPTESTLIWMVPFMLQSLFLGYSAIVVASAVSLCFATADLVMDQFKLVIYGINNLDNRAKEMYKKRFPGSDMKRMDNREYDDCYYDCLVQNVKHHVDTLKWMDKFNDMASLPVAVPFFGGAVLIGMALITITEEDDPRLGPKCLAAMSAFSELYNMYLLCKLGQEWQQLSDDLLDALYGCRWSGRSERVKKAIRIMRLSCARPMKFTAAKLLVLNMQLFSDLINSAYSIFNLKAVSQEKES

>AlinOR10

MAFSTWIHAQLVTEEQFQMRLRKYGWLHYLFQFSLVNSSYRTVPTLFIYYLYLLFTSSVVVFHLFCYIKTALNAYSIGRADMSVANVHSIILGIFILSVLSSYVVDKTTIVDIEELYLESLFDYDTVIPNSATLFEVAMTFCGKLGIVLGGMGLFTNVYLAAPLMDLRFWKESCVIEGINFCLALPHYYPYDSEDGWKFHATEIFQVLFGVYRISVFCAVQVTLTLWPLHLVRELSKLKASLEGLEERIKKRYYQKTKINLDKVNLITMNKDKVFNECASFCINENIQHHHNILRYHGTIDAIMAIPSFLAYTTGTATMAIAMVKLLSVEGDTTLGGNIAYVTVLGAEIGFMILISVMGEAVTMKAEEIFDEVAHIRIENYDLDFRRKVIIFMEGTIQPIALSSSKFNKCNMEAFGNVLNAAYSFYNVTSASAALDK

>AlinOR11

MKRSNMAGAGKDEYVIKDTKVFATCRRALVLMGFIDDGSLISKIRKATFVFLLYTAPFHHLIPAFVDDTISLDGILISVSLLMLYILLCVSWPFMITRSDDILHLWETVRKGFYHYSDPLTKEERTILSETDDLVIKTTRISIIAYFCAGFGTYLKEMSPASMREYRPPYPGWMPFVINSNFRFILTLFYQLCICLNTTFALEAIFLLFAYHVFHFECQLRLLTQHFKDTFPEGLSATVTYGAKYRKNTLRRLKECVRHHLVIKRFHEQILSYFGICLLVYRVIVTIMLCILCYLVTTGISVNKFMQLLCLAMALLFLCFIFCIKGEQVAQMGDGWRKTVYEVEWWNHPVEVQKTIFMMQLGASKALRVYGVWKPVMYSHEGISVIGQEAFSFFNMLRAMK

>AlinOR12

MTLFSFAKKKLKWDEPLGLTTITATLTGSFVRIAPPPSYRRFCYFLSCYQIIMSALFMSIAATKAVTATDFFAESIEALRFLVTGAHILAKHLTMMVREKEFLELIDQIRTAWVTYQPSNSDLLSKTLSSSNKYTVIIFSALQFTLITSVLGAYAKNTNDLDDIQFPLQVWVPASLRTSFVAGTIFQIIPYWAPLVIYCTTISFLNSITRHVEALGLALARDIRRQKKWKTNRNNTLYKKHQEIVRIVHRVNALMASNWGFEMMCSTLQLTLVTYNFLRCLKRNDVEFVNQAFLVLVNFGVIYLIYGNGNRIIQMSEDLHESLCASDWHEASVKERKNLLIMMFKTINPLEYKYKIIHFDLPGFAKVVNTVFSYITILRSVDEMEDKEGS

>AlinOR13

MVLVPFFIKPRIGRNAAIDRGYKLTSMFYARLAGLYPDLEAGWRYWFFGTYLNSVYVAYAYYVLAYVIANIIAVKYKDFELIGSTFCFGSYTYVYSLITIMFLIKRKKIDRLLEIVGNDVYKYRRPPTKEETLIKETETMKVIVYGRYTFFIPCSVALMQMAVVPAYHGLRGEYTSIVNGSTPINKYSPLPVWTPVEATTGLSFFVLYWCQLCPGFVEFLIFHGSCTFYIGGSCALVSNLKLLHHSLGRLVDRAEYLYDIKNGKYDHQSKIPLNTELFDECMVECLKENVQHHIEIIKFHHLFQDIVGYSNLFIFSGAAVTISTPPFTIIKIAELEDRHQLLCAGIVMIGHAFLSLFLLAQYCKYGQSIEDESEKILESYYFTPWFKARKSFRQLLVVAMSNSLNPLQIKSAVLGISASAATYMSIIKSAYTMLNFLVTAK

>AlinOR14

MAKKSKEVFETDPQRFSTNKELLQALGLLWSKTDWNPLRSIFSLSLITSGMFHYSLRVAFTYTSMELLTIIKILHLTNVYFLAGLCAYVVFLQSRRFHIIHSIIKDGFYNYPEGLTQEQMMIRSKANHKVSKITKYGLRFFVISTLGSCLKEPVPYDFEEWTKEYNGWIPFVVDSWPKYFASQFYHLLAALSAALLGGSVCIVFIAVAEHLLAQLEILSISFRNAIGCIPTRGDKVAEKLAYQRMKYCLQHHNVILRFFDEFQKYYSIPLFCMLAGTTVAMCTIAFVVTDPSSTFGVSAAFLSLMAPEVAFCICYCTYGQKITDMGDVLRETVYNAPWYYQPRPVKMAMLMVLIKTRKPLTLSAAGLKDCSIKSIGEITQTTYTYFNALQIFRGNPTSNK

>AlinOR15

MSGDIEQIKSNSLTRDWYIKKGRDENNGFFLIIGGMYIGYRWISVLHAVLTAWHMPVLLLAVYYGREDFVVVSETIHFIILLLLAFLISMTYLSFRETLDRVFEAMGNGYYDYDGTLDTKTEKMIQQLQFESDRRKTILKYMFIGGCIGAMICVSGIRPILQYYLQKYIKIKKLPDGVNGVKNTFIYYPWDPANLWLNLIGYTLQDVYTIITANVIFGFVLIFVSTAESLSVQMEKLKLSLKRVKIRAAYVISKKPKDSDLSTDRDFSKALHICLRHSIKHHQVVIRIFDDFKSINHTALLWLVGGLTFLLCMSSVLFTADDVSLISKATFVFFISSELLATFLMCWYGEHLGGMSYGLPGDLYDTEWYEFSGDLLIYHRMLAMRSSKPCQLTAGGFSKIDRNTFLEVLKTAFSYANLLQASKQN

>AlinOR16

MGFVLDGSLVCKLRQPVFVFLLYSAPIHHLIPAFTDDTATSDQILMAWSISMLYVLLCIAWPVMIIRSSEIFGLWDTVRKGFFHYSDPFTSAERMILSKTDDIIIKSTRMSIIAYFCAGFGTFLKEMQPQSLHNYKPPYPGWFPWTINSNFRFALALLYQCGICLNTTFALEAIFVLFAYHTIHFEGQIRLLTRHYEDTFQPRLPASVTYAAEFKRRTLRRLKECVRHHLVITGFHKQILSYFGICLLVYRVIVTIMLCILCYLATTGIALNKFVQLLCFALALLYLCFIFCLKGEKVTQMSDEWRHTVYEVDWWNHPVEVQKTILMMQLGSSKALSVYGIWKPAMYSHEGICVIAQETFSFFNMLRAMK

>AlinOR17

MSIANECDRRVGLRGNRIERMYAEVTEIEKTSPQTEGMRLICIIGCIICSWTCVEPFVNAWAGGKELEFPFTGTNENSKLFVAIYLLQCSLMFITANFCIVIFQTLMGTALNLVIKYKVIGMELARLNEKMVNDKDVYKTDLYQSIRRCVQSHHHILRIFERYREVCTYGFRYSYVGLMGATTLSRTLLSGDEPDLGTIPHLIAELSYIGFFCYILNHLEEQNDNLKDAVYAGDWAWMPKKATSALRLIMLRTTKRPHVILVKGGGPANLETFYKLLNGTCGYIIFGLVLDQAAF

>AlinOR18

MNKKIEGDSASAQEAKVERHLQEQFSVLTVIGGVYGFYRSPRWSIISYSMYITMYTCSFLISADLLYSSIVLRNNISLFTVAFHAFLINCVVLTGSVSLTLQRRRTTEFLYNFDFTGYWTEYQESKFFVTLEAQSRRRILQLLILFLSGYCACGMIGVVGPFVDMRLGIDENVTNVTGIYWKGLPFAMWWPFDAHKSTISWISCFMCQGLWASFAPLMCTSAIILCFNSCEKLLNHMKLLIYAIEHLDLRAKNIFKKKFKYVTPDKQGIEYDDCYYECIVQNVKHHQKIVKAIDDFMILANYAIAVPFFGGGLLLGLAGLNILSRDDPRVGPKLFCASLGMTEALNMLLLCVYGEKFQHEGEKLFTSIMYTKWYTRSLKCRKALMILQCGTLRPVKITAAKLIVLNMATFANLTNSAYSIFNLNSVVSSGE

>AlinOR19

MGWLRSSKKYLTKMRDLEDTDVRRVVNENYSIIPRLSNMVSSIDEGYIPLNIIHSIIFNVLVYLYLYLFVVTCYLLRDDFVLVGVQFHYLLLALFGSVFQFHMYTSRLSFLQAHKIIALDFYTYEMDLLVDEKAKFKEYMLKQRRQLIPFMFLIGVIGMFIVGFGPLIDNMVGAGHDGDYLNGVYMKTPIPMYFPFEIDDAVSHYAATGFQIVTVVMLALSISGVVFMYVVTTQNLALQFRVLIASLNKLKERSKARFDKLYPNDKINPKNLSNDDKFQQCIAFCLRENIKHHQVMIKYYLIHARLIGVPILSAFFMGTFIIALSMIILVEKTDRYGLLMTNTLAMIGEVGNLFIACYFGEEILTLVSR

>AlinOR20

MNKENEKKRTSFSCVDYRKYMYCKLIWIDDGLAARGLTRPLLFIMVWMMGMVSFGLISFVMSTQNKARLDNLRSFLLESMVTMSIFNEYMSRKSLAQLHQFMDESMRTSRTELREEEEILETAKSQARKHLAAYIVIFSFNLAAMILSQPLAEWLQGNSWKKLPYPWVVPPSNTEFMFWIVFLYQSIGLYFSHCLGMVIMSFSSITIQVTALFDVLLLSLRHIEARAKVRMEREGTDYITSITDCLKDDVVHHQRLVSELVSATPHLRRTFFALSVTISMIMACEAYPLIMGNFTLGELIKGLLFLVVQFMCWGQMCTRMEIMADQNSEVFHALYNTPWYSSGLKYKKLMVTPLTFSRHSMYIKSPLFTEMSATMSTFYSFVVSSFNILNLIRKMK

>AlinOR21

MAFGYLRIDWLTQDEVDVFDLFQKLMSNVGTMSDSKEKRRLTIIILSLLFIPMVIASLCASIIYRDDFDILAYSLHHTVLMSLAWVLNNVVIPVFRNQYNFLMEGTKKTYYYDSDLVNNYAKDIIHKRIGISRFIGKSIVIGTIGILIEIQIFFAAEVIWFQTYRTLFPVYTFGLDLDNIVVLVSVVLYQEVVMCWTANLPVVLLNIAYSTWSHLDLEMKILVFAITNVEKIVMEKSGKRSLDRTDEKHRSEIYESYCCHLAKHHHCIISYFEAYSQAVSLITTLMFTTGFIMFVLVGLSTMSDNLGVKLKLFWFLVFQVIITFGWCWVGQYIADKSAEISEAVMGTPWWLMPKSCHSTLLLIMVRCKKPLVITSSFGQQANMQTFMDMMKSVFQFVSVLYQVTKGENEE

>AlinOR22

MSFFLDYCDKLEKKRDATVKKFIRDNYSILLQAGTIDMNLSRKYVFWSCLLAIFNYAVLANQDWMLLYSTFLLKDDFETASGDLNYFLLISVSIGFLCNFHYYREDFLDGCKIMSGGLFQYADHMKETEDMIKFRKHMRFQRNLLIALAIYVCNIGGIVVLGPVIDDYTGHGFNGTYDENGVNRRLPVPLFLPFESINGIGYLTALGMTTISGTVTCAVIGGASFLFSTFSHQILTELKILSCSIKAISSRALKMYCRIHNVSKKSVDRKTLYSNPMFQDCITECIKENIKHYTNIAECMEVVERFVKIPVFLSFLIVTLAIGLSMMKLNEDIVRIGSSVSFASVAIGEILNMLSVAVNGEHFLTLSHEVNWEIYFTPWYKFNLKNKKMIRQFLQSTQNELYLSAWIVRFDMEMFASVVNSAYSFFNFLKLSKTINVEEM

>AlinOR23

MYFLAIPAFIGLGTGIIMILPYATKWINGMDEPYIVGMVNENLPFPCWYPFPTHEGVTHWVVVLLQCGAAGSLAFIALTLLLMLLENSQRIKYEYRVLGYSLEIILKRSMKLYLQMNPWKKSASVNVQEPEFQRMIELCLIDSVIHHHKILEYVSLFGQQVSLLGFLTYSVGTGVIALSLFNIIDAINSEDISSIILFSIFIISEVLVQFAFCVLGEAITSESVALRNKLYCSKWHYFDRRNRKIVLNFHTAMTEPVVITAMGLINVSLETFATIMNSSYSFFNIVNST

>AlinOR24

MTDIKNNSRAYYGIDVLYFKFIGFWQLLTEGVWANKITIFSIVYGLLFSAYVIMQFSIMFVQEYDFSIFTEKLSVNLTCLESVIKMLFYCLKRTSLLELLAIFRLDLLLSSKHSPSITDKILTSNISTVNGATKSFVIMIFTTVGIWNCMPLFKRYSSDESTMLQIMPSWYPIDVSYAPVNIIVYIFEFFVMIYCAALLYNVNCTFSALALTASAQFELLSVNMANIESNAENTIDKESIVDMNDEIKKDIMNILLRECLIDHQTLLRLLKQMEDVFNPIFLFQMLTSTFTICLVLFQLNFHLASGNDLPIVVTFKFIMYLVFGSMELLVYSWGGQMIYNKSEEIYWSLQKCGWHKGSHEFQTNVMIAMIRSQYPITLTAGKFYAVNLASFAQVIKASYSYFTFLHGSISEK

>AlinOR25

MMLFLGQTVALQMRILSTAAHKLDERAEKMYMKKSGGQPPPSDPILKRRDKILDACYKQAIRQIVEHHLIIQEFYANYNQLGRWPTFLAVLNGSILIAMSIAVVIMGAKDVPSTFISAGELLVAEVMSLWLLCETGEHVSEWSQKLNEGMYNFNWIDISTSNKRLIMIFKEKAKKPLVLMAGGLTPINRDTFGTIMNSAYTYVNLLRASERN

>AlinOR26

MEDSWLIRYFSAGTGRQEYERIQAVAIKEFTPLVVFVGVFLPTDRTVLLCIFGFTSILVYSFYTTIFTYTCIVATDDFVLWSEIIHHTSLMYLGVFIRTVFMLKAQDMFILTQDYVDGVYNYEEGYVDPIFQELKDKSRALQRKLIMLPLFIVAVTGMAIGLKPYLDYVNEVEPHPDLMKNGVNFNSLVPVVYPFENANTYQVLVMNCVLLYFALLVILTVIAADILFIRVSCRISLEIAILVESLNLIGKRARRLYARKYGLKPPSKKNEDWPLYQDCISTCLKENIVHHQNIIKFYESFSAIAAPAIGGGFFTCTIVLGLGMIVVNMDNVNISDQIAFTGTVFAEMMNAFMISWMSEKIGEQNYELYNAVYGLKWFKWRRDNKKLVITILDGTREPLFLNAFGLAKINMEAFGSVVNTAYSFLNLVNASETLEEKK

>AlinOR27

MEVLHPNIVIFLKLMKSTFYWYDEDPPTSLWDRCRRIYQYSRVVLFVLLFIQNVVGLYNTDGFGIAEGSFLHFPVCIQLVVINGMVFYSRKALIKLTTTLNKHFIESNEPWMIAISNKYTVPLWKMIKIMKVYHFYANIAFFLSPFIADTILHYGFDALEKPFYMPTPTTAWMATNATWDLQYYSVVFLGLWSMQEVMAVVMGFIYNYSILLIFALIQLTIMNEKVKAMKLDGTQKEVDAEFKAIVDSHNDIIRLNADLKSFLGVQCAFQSLFSSFTITLCLFTATQRPEFVVRTSFAWGAVFYASTTFIYCSLGQLLENKSSELFYALYDLPWYRCSPKVRKDLNMIMRQSHNSLLVDYHGHFKMNFESYMQIIQQAYSYFTLLNSMAG

>AlinOR28

MVIEVRNMEQGLKEISGQYYAASGISGIGAYFKKDKPFFLIRLWVQASTWIILPQVTLAHIYTALFTGIPLATRFLSLSLGLDQLQTTIKCHYMLHNLDRFRSIMLQLETFNCVNHLGFEAITTMLKMCKLVKWLRSTYNFACYFTFIAWTLVPVVSSPKALFYGEDGESMMKVLPPEYPFATDYFPMIQFIYGLESLSTFVILTYFASTNLIMVTDILLICQLFRVLNDSVAPNKNRNAMSLRMFAVDHQKLLKICAEVRDLLSPLLALQLGISVMTITLAVFEITMVNQASSDGGINQVVLMSRKTSYTFIIFVELLLYCWLSTELELSCLSVRNGIYNSEWYERLGTKDYRNFIMISMRALRPVKLNAMKIATLSLGTSLEVLRMAYSYYTYLKRLH

>AlinOR29

MDSDQFRYVREVYRESLGLTGMDVFLDEKPPDHRVIRWHVLRKFFLLFVFVYYPIFISTQIWGIAAGDSNTLKQISFDISLLGHNIQNFIKMGIWITRIQTVRSLCLDFPKFHINNYRPNLASWILEKESKDARNFTGRCYWISYVNLLFWVVLPTSTAGVIYLAYLAGYKEERDTYIPRYSPVRFPVDMTLRSSRLLVAGIEMFQFYFGFLLFQPIDMFFTAIIQMAHAQIRVLNSALFSLDGELQEYWGIQVPEGDPKQPMEVRLIIEDHQKIVKYGQRLREFLNPILGFESFNCITVICNMTIVAASEFSAEGEFLDLALPAFSSILVVFTSLTCFYTFTKMTAVLKDAEESIFHALYASNWYEKDVNYRKSIILMQKLTHTPRRIKMCGIGDMGRSTFIDGSRMVYTYYNFMQRFK

>AlinOR30

MTKTDLSDIIQLLQFTGHYFTFEGRRRTSFETLQKLRVIFMVACNPFTLSSLFIGGLKKSMGVELFFGLMGFLTAMQHVYAFNHRKDTEDIIQAILRVRRKYQKGSDAEFKQDTRMIWKVVYIYFSAMTALMVFYITLPKILDVIYGIIWDDPIALRLPQSMDAFLEEHQHRNLKYVIVSLISSVWSIVSTYSHFGLDTFLCLIGFYYSSLVKTFCNSLKLDTSLSSEQLTIQIKTFAAHHHELYKLSLRMRSIFGCPYAMQNTFGAFCIVSLVYAILSDDSGGPLILLANIFNLMILAGMLTSTAYVGQHVTNESSAVFDALYGLPWDELSPTNRKYFVTMICAAREPFTIHFHGRAPLNLTNFMAILNTSYSYFMFMRSTL

>AlinOR31

MDEALKLANSTVLVISGDKKLMLFSIQKAFLNILGFDWFPGGVNLPLPAFVKHTLYRLLYPVLMIVVAILILVYVFTHEVVTENDFIIKINGYIVSMTFFAASFKVLIFIFQKNMFKELFLMVEMVGAIDTDAPSTKSHIYNCYIYITLVLMNPGTWAAWWVIVHNDTPFRAQYPWGNEGFGYMLSYFVGVNGAVFCGLCHILVDTSFMMVVAGITLHVDMLSASLSYLGKNRFKDNKILSAAIDKHAELLRVSQHLSQCYSNLFVAQSVYTVGHSCVLLFGAVHVASQVEVVMNQGTMLATSYSQLLVYCYYGELLTTKFSDLVFDSYNNKWYDCELSVQKALPNLTLMSMRHVSLRGFGNVHPSKSNWLHSLQESVSYFLFLKTISGEK

>AlinOR32

MDVLHYWDLTGFKFFPIYDHMMRSIGVHSERGEKSAVAIKRYFGNFLIILAIIQGWASTVAALDSLKEEDYKAVTNVMSYMSITFSCLSKVQIARSHMSVMHRLGVWIIQVKKNRPKDMKQPLLEYLVLKANPSFFYFGLFAAVFWVWVPIVTLTFQAFIPTKFPYLDKYTSNSISFPVMQLPLYVFFTVAITYTATSLLHFLAVFTTEVKLLSEKWAQVVYDKRRPHAYMESMKTCVQQHIKLLDVMKDLNIIHDSMFAFQVMIFIVHFVSFNFCLVMTSGSNAISSVFPLFSSSMIEFGLLCWMGEEITDALQQFHRSIYMTNWYEASLSDKKNMIVMLEFLKKRHVLTGTKVFVASLDTYVEAAKQAFSAYTLMKALTE

>AlinOR33

MIPFIFKKKDSNDPIVMRGYELTYSYAMRLGGLYPDFRGFRYYIFGFHLNCIHVAYLWFILAYIISAYYGFVYNDMELVSYNICYGLLTIIFFLVGHSVMYKRNHLDRLFETVGKGFFTYEKPLDADEQAIVDECDMKCKKTAKRNILLTMVLTTWTCVVPPLPKALKGEYSSVVAGGVPINKHLPLPVWSPYPIDTPLTYWSMYALEFTAGVTEAFIIATTCTLFCNLCTIVSRELKLLRLALRRTRSRANYTFKMRGYSFLPGSNYAKYKLFQQCMVHCIEECIKHHIALKKFQEEFQGFMGFPIFAIFSGTALTISSPMFMLLTMTEAEDSFLVLMKILQYSSIIFSFTCFLSSYCLFGQMITNESSLVHFAFYETPWIDGDLDFRRKVIMGMIHSRKPFILTAKGLAAASSETLVDISKTIFSCFNLLAATQ

>AlinOR34

MQTEGDKIVQPLIDALKFGGLWFDFSDHKYGEALKWCNIIRNAIAFLVWGIITGYFFIGGLSFLLTESGVFMPISFDEGCMSIIVICNLPAVRNVIQIYNKRFDSFSSIPWARSIIDEEMNKFNKIFQLPKTALVVFYCLYSIAPLMYDGYRACVGNENPYVVSLPLNFLLELPMRRTPTFFLTVYLANIYFLIIVPRFIAFEALVLYMVAFVVIDVKIFIRKMEKLSENDDGTEFIQKAWNLKDVTLHHSSIVCVVRDHFPLLGFAILLQNVSNSISSCLVIYLMKTSYNNGDIILAIFCGNFFVILMVINLMFNGAGVIIENQGELLLAAIYNTGWYKQPPSVRKEVNFMLMQGLKLLKISYKLNSVNLEAAMLVMNRAYSFFTLINTGE

>AlinOR35

MSELHADIAKYVTLMKSTRYWYFPEDSTSSPAVDMLRRYYYHARPFLFFITLIVNAYGIYNTEGMGVLDGNLLFIPLSLLSVVTTSTIYFNRAQHRKLTMLLNQRFLNNNEPWMVEIQTKYTSTMWKFLKIVIVYQRFCAATYIVVPFIVDTILHYVFNYLDAPFFFPLTFSVFLPRHITWDVNYYAIMFLDAWAGFEIIANLQGFIICYTVITLFSLIEIQIFKEKIKSLDFEVSMENRQQQFQMIVQWHNDIIGLNLDLKDFLGPTCAFQSLFTSLVLTLTVFTTTVTTSIPVILAYSCGCFFYFSAGLLYCSLGQLLENESSEVFDALCNLPWYRSGSDVRKNLNMMIRQSHNPLIIDFHGHYKMNLENFMQILKSAYSYFTLLQSMTSSG

>AlinOR37

MNILGINPDRDDGRAVDYRIYPFFKILYSESEDGGKVSMARAVFVIITLAIMASGCLWSVTQSQTAEQMLDNLKGMHLEVMVLMVAINECVSRPRMRRIMAYIDKSRANPRYGFPEEETIMLEASKKARADMTFLVIIFAANFPLMMVTKPVTESVGGHSWKQLPFPWTILPDDDDMIYVAILLFHTLGVGFSHCLGIVGMCFSTITTQITALFDVLLLGIERIEERAARKMKQLGLSYEESMLRCIEESVAHHQELIREVRSEKPHLESQFFAEIVNISMIMACEAFPLIRPNLTVLIAIKGLVFLVVQVICTAVLCDRLEIMADQNTEVFNALYNSPWYKCGVDYGRIVSIGMTFSQHSLTIRGKSFLGLIATRATFYTAMVNTFNLLSMIRKMS

>AlinOR38

MSFLLRFIQNLAAEEDEEFLNLLRKDYWIFLHASMVLPTWKHPIVSFCLFFHHATCLFMHFVIFSYSMYLLLQEGNLEVFSLVLHYNVILSFAVFLVVYCNYMRKELVRLHKIFVTDIGIYRNGRIYSDKWCTDMYKIIQLEKYYFLMIPGLMAAMGGLVCVVPYVFKSLVGIEHPYSSTGLSMTLPVPAWYPFPTHEGIWHLVVMLGQFQACGLTAFLIVNCQYMLLNITSKLKYEIRVIGHSLDTLLTRSQTFHDRITSIHHGQHKPVTVAILQTVEHHGKKRDVLTSIDDEGFQRFIGEEFKDSISHHQTIAAFLSEFQSFGSVPLAAALLLGVVVIAMSLYNILMGMRVDDMGIIVTFSLVIITETLAMYVVTALGASLTDELELLRSTAYFMSWEELDKGNRKIFLNFFTVITEPFALKAAGITDLNMETFSSLLNSAYSFFNIMNSVD

>AlinOR39

MGLMEFFDKLAEPEDEKLMGVFKKLYGPFVQIALIYPSFRRENLPLTFFLFIFTFSVFLYHYYLLSMSIFKSLDNFELASLAFHYWMIFTFTLITLPLLIINRHKMGETHRYLQDNLGEYKSVKIYQEGKPTQYEKNKRLEFIRFLFLPGMVVCLASGLLLIPYIRRFTNPPHYSENGVNLNLPIAAWYPYSTHEGINHGLAVLGQLMTGGNLALTLGTLEVILFRVAQSIIFEYKVLQYGIETVFSRAKKLYWKRNASLAKAHIHFKDPEYQKCVTECFKECVRHHYKIRVVLAGFEYLIKWPGALAYGFGTGVIGLSLVNVLIAKESENYENVVLFLLLSVVESLNMFMLSVIGESITTETKVLRDELYFIEWYKLDITNRRMMLNFQMGVTNPVIVYAGGLVALCMDTFSSIMNTSYSFFNLVNADMGNKDEK

>AlinOR40

MFGAASEKLGFNERTLEEEDIAFMQFCGLYPLNKFYTIYYFTSTTISLFTLGGMVVRSYLDEEMDVAFETAHVLLIATNMFTQNLISHYCHHIVEMLLRAIDKEFYSYGDTMDEETKKIINELNNEKIARKKMSVKLFKFQVACAGIGVIVKRLLLVLFTDSASKKVDGENWGIYQAPLSIYIPYSNYWGPYLLGMFLSCNSVLTIALTAMGAATTFIRFSEELLQQLQIIKLGLRNVLARAHHLHTIKYGRPYPLVNGETKEDFDKCLSICLIKSVEHHTIVIKLFEEFKKMMKVPLFTVIFDGGALICMSMALLITSESASMKLLMPSFIAAELYYTYIYCAYGEKLTNLFTEIGDQLFLADWLIHQKTMKPYMLIMKAYSFYPKKLTAGGFTTPNLESFGNVLRTAYSLLNFLVTQQ

>AlinOR41

MTQSELGMFNGLKLNSSVRIEKWISFGYRLLLLGMTPEKFLDKSPAGRVRVYLTLFMIQSFYCCHEIAAVVYFSRSLLERITHGYIMTYVVAFNMEWYFLLYCVRSFHENELHLERFESTQAHFEFSERAFDRNTKVFLTCLAICVAWWGTNNSIYIFGPLLETVMSFIRSGEFKQVSILPQVFSMPWWLQIIVYIHNALLIFSALVYCVSSFIILGSKILKVKTQCDILSEALKNDTGEESNVRAYVKDHIQIIKAAKLLNEQLATLNAIIFTACYLQIAIQMFTLTLFEPSGAYFFAIAFDSLSIFLILSIQCWFASIITLALESVSDAVYETDWYRRDKTDTLDVLLMLQMAQQEVSQKIWFKSLKVERAASLNMIRSSYAMYTALMIFQD

>AlinOR42

MKDAKPAMEPKRNLFTLSLRLMALQGMARPSNRILRMISFVWKYWMLYTALHFVMICLLAVLIGDNPYYLNLETCSGMFSGMSMVYRHFVLAFNRKEVLRLVARINALVEEIVDVYGVKTILPWENLCYGIMIFSTCIVTFTTIPAYAYSYYVFYTTGEVTAPYEVYMPFERDEHHIHHVVIFQFLSFIDQAMTLVVSNTFIGTIVVIVSEITKIIAARYEMIRKKTVDTLKDTIRWHSEVIMIVEDTNALLGSVIMMDCLLSIVHISVSGYLLVKVGIENGTNLHKYVFLNLLCVTIPSYFCLCGHVIALGRDRLRQAVYQNEWYDLNPHDKKLIILPSWMADKGLALHFKRAVEFNLPTYLAIIKQSYSFIAMLQLMDS

>AlinOR43

MKSGGRSFNKRRAELLKAYNAIHWLTFTKWFYEDNPIEDNVWRDKRLWVHFAFAFICQSNIIIFKVFHLVSEENFFATLTSLTSGLVVLLVVVRTYVLYQIPTFKQLYFKPEIFNCNKHRPTSSLDVLIQTVKHSRKVGIWCMMLFLTFDVSWLVLPIVPPIIEIIKGTNQTYDELIPQYPSINPVRLTWLTKEAKYAFDLFMGAVNTIPWVGFVVVYYAVVQLFLAQHKIMMLSMTRGPQVPGDAKEPLELRLWIQDHALIRKLVYQLRSTVSPALAGTICANVFTVGLNMLALISSPIGPEAPLYTRYLFYFSFGTYSAISIFDIFIHCWLSSEISNSGKELNYAIYAGDWNSDLKRPPQDNVIPLMVCNKEIRFTALGLIPVTMTTFTEVIRISYSYFTILKETGH

>AlinOR44

MSSNTVYISEKSDAAKKATEYVYERLFIFTIFGGFFGFHHTRWWSMFTYTVFTLYYISVISMIVTMSYSSFLNQSNTSVMSGCLHMTITGLVVSCASITLQLARKDLFKLLVNFVLDENLCEYQSNEYFSHLLKKADEKLRSLLILWLLLYGSAASIAVVFPFVDVYLGYELTLNNVTNVYWRGLPFTLWWPMDADNSTFAWMTCFTSQGLYAMFAASLCTGCMVFYAMMCENIFNHIKLLVFSLEHLDERATLMFKKLYPGKSPKKMRDLYDECYYACIVQNVKHHHRIVIFKDAVMKAANLPIAMPFFGGALLLGLAGINLLSHDDNRIAPKVFFSCLGMTEAGQMFLLCKYGERYQDLSEVLFNASFYTSCFRRSMKCRRAMMIFRLGVSKPMRMTAAKIILLNMETFANLVNSAYSIFNLQSVTS

>AlinOR45

MICSVYLFLFGVTAIKQIDDFVTATQTVHFALIISGSILAMVTVAIKRKHFIIIVRTMSVKYFDYGDSFIIPEMEEEYKNTKKQRIIFLTVIPMYFAISTVVLGLGRTIDGYFGSPLNETYVNDVYMLTPEPVWYPMKVDSDILYWFLALSVVSMSYAACITVAGGDWILFVLYLSITQQFKILIYRMRRINAYAYRLHRKAGGVKLRKSMMFSNSSFLKYFNLCLSKHAEHHSIIIKQFEQLSIIMSWPAGFIFIFGSVIIAMSLLGVSVQGGGKPSILVLATFLTFSEIGEMALFCGLSESIQTLGLSLHEELYRLNWADADISAKRTIMIMIEQSKRPNVLKAAGLQSLDWMAFSSIINTAYSYVNILMAVDA

>AlinOR46

MFYTKRMAELFAMDTPKRKTNRRKYYWYLLELGGLITNWDPRYYIWTIAYLIVQLILGFYFLICFLVSVFKAVNLMGMCVYLNLFALLMLSTFMLIVTILLQEKLLIFTSVADDAFYEYGNSLTRTEEIRQMNENAAKMRKMMFIIAPSWISLVALSIMLSDLVDVAFSYPASNETVINGIDQRLPCKMWIILPIDNIIVRLLTILAQGVCFGGAAVVIGTADLIMFFSGQTLVIQLKILNMAVLDTDKRAAKLYETNLGRKPPSEPSEKSKDLQLMGFYEFCLKQTVEHHCAILRYVVIYSELINWPGGLMVINGSIVVAMSMLSLMQGGGQPSVLITSCLLIVAEVASIFMVCEVGETVTDQCRELFDSMYQFKWMDCSAEIGKSINIMKSYMIKRPIVLTAGSLTPIDRNTFGAMMNTAYSYMNLVMASGAVD

>AlinOR47

MVVEKKTTTDFEREVRQTVGEITAEDVFPVQLRVLQIVAMWPAFNGSPQKQMLTKVILNFNSFVLAFCTVGLLLKGIITRDLVDRSEAMDIFTLTGSALYKMVFFYIYHNEMADMMNCGAALIHQVPPKWMQYVTFFTVLHCFMGFICITFWALCPILKLIFGETELNEMTLPINLYDPMEAKGAMFALFYFICHYGLISSAHIYMAADCYLFTAIHLANGGFQTLNGKLEDMKQAIAHKGDTSHDSANEYIKDCIKLHVHILTFIRKTDALFRSLFVADVVHAIISLSFAMLQASESKGIFENLKMTVFVSYCFVHQYLNSYFGQHLIDQQDTLFEKLLLSVPWNDCSRNLRKSYHIMTTGCLKSVRLSAWSVYTLQFATFLEFVKTMISYFMVLRQVQDETEVQIEI

>AlinOR49

MKNLQTVVPSRGWSGKYITRSMFRREYARNQLLFRSEYYYMSRPILWLQRSFGRMPYSVISGWLRHSNWSISFIYAVFVALVNVGSHFYYHEHITDAWIQTMRDSVNFKSVLFSYLLVTQPPTCFVTIYSWLYELPRIVKCYNSTAILEHKISGVFPTSTRSRCTRLMVPFGLASLLIASLVVGSLLLILRFREQPKILLIIIAINLIANYSYNALWCFNFFFISDLAAKLRKHMLLCLQDKKNCSFKLKTCRKIWISIWKQSQSYAQSIAVTVGYSLIINSILFIIGCYGAIASFRSNDILDVVEKLPYVLVTFINAALIFESSYQATDKLGNAFLNTLVFLNKDSVDQESVEEIEQFVDTINHTRNAAITLRGYMTMDRTLLVSFMSNSITYLIVLLQFQGKSQDSGMSTSNSTSPVT

>AlinOR50

MFRREKKERPYVIKDPKDFATDRKVFLWLGFVYDGSIFSKLRLTVFVLLLYSAPIHHMLPVILDKSTTTDEVLIALSINMLYVLLCIAWPFMIYRSSDIIGLWDTVRQGFYHYSDPLSEHERSILSKANDLIVKSTRMSLIAYFCAGFGTYLKEMSPNSMRLYNPPYPGWFPWTINSNFRFAMALLYQLSICLNTTFALEGIFLLFLFHTISFEGQIGLLKQHFEDTFPPGLPAELTHTPAFKQRTLNRIKECVRHHLVITGFHKHILKYFGLCLLVYRAICTIMLCILCYLTTTGIALNKFLQLACLAALILYLLFIFCLKGQQVSKVSEGWRETLYEVDWWNHPVEVQKAILLMLVGAGKTLTIYGVWKPAMYSHEGISAIGQETFSFFNMLRAMK

>AlinOR51

MKDHLILDDMGEVDIEWLTEEERALIISFDKVHSWTGMWRNAKRIQWSYFWLFQMTMFMIIYFYSLYFFLEELEILTHVIHHIIMAGDDFMYIYLLNYNRRNLEIVHDLNLKTYGYGSDLVKNYHRKLMTERLKTYRLVYRFILLSAAATILYLEAFFVLEATILKTYVTMYPIYLPIDLNHPVTYTSVVFLQHLQVYVTLVMGSGLVSILFSAWNHITLELAVLTFAMNNIEEIVKEQLSRFQFKSHGTKEAARNKIYRSCCYHLARHHGSIARYFNTFKSASRLTISCIFLTGIVCFACVGITTVTDNMGIKLKFFLIMVVQTSVIYAWCAVGQYISDQNANIQWVISGIPWWKMPKPCHSTLRLIMVGTSMPWFLTTPLGQDANNESFMDMVTSSYRIFNLVYQMMFSS

>AlinOR52

MMKNKRESSKPTQKGTMRLGEWFSFHFSPQKTSKKKPANTAYHDGVPDFANSVTCFTNVRYSLLYLDGSLVNYLKICIPIFFLATGTISFAMADIVHMNEKNTIWIVENGHWCIIYTAVLFWDAHMGWFSPIILEMNNSVKYGVYRYEKYEANNRMEFGKNNAWISKMNRVFGVIYIVAVIGTLVKSTILEEQYPFKHLFNGWFPFEINSFMRLNIVRIYELGCAWSAASGAQTFFMTTMAYTYHVEAHLRLLMQKVAKVLDSQHPEKQIQECLAHHRAILRLFNNLSAFFDPTVGFSTLTATFMVCTLLYLITNPDFDMNVIVTFSFLVAPELGLLVSVRIRGQKLTDLSSEVNKTIYDLNWLEQDIKLQKDLLMWLRLTSKPLELKAFGYRNVSHAGVKEVLQTSYTFFNMLKAST

>AlinOR53

MAEGEAYFDYMVQILKNYNLWYGYSTASIQGIILTMYSVLQIFLMLGLLSAAISQTYFYGMSYIVHDSAVFLPIGFMGLVIIIYMALNYSAIMKTASKFELFLNSFTVDWEEELIKNHMKDTRTVVTLMVNVISFYVLSTGAMHFNSISLHYFFGIFSKPVLLPLVLEKFMEGNFQLRPMFFVHVLLSLLYIKIAASIITVIALNIHFSGSAVGALQVLIKRLENVSKKTADGCHKLAADSDLRDTIQKHVELLNIVSDMMEWNGFIVSFTLTSCSILFCINAITVKKSIETKEYSSTCVWGSFLLVALAIGGTMCSRGQEIEKMSEELLRAMYNLPWYRESSKSRRNIVLMIAQGNRLISLDYKGLMRVNMVTYSEMVQKAYSYFMILGSVE

>AlinOR54

MNFSKMKMLPLFAKKTRTCGNNRKSVNKKWDAIFKLGLRMLYLGASPDQVWRGERNVLRFGFFPLLILLYSGFGLLIVVFPFIFESDFMESLENLYLGTYFVAAGFQWAYLQIHLDGIYEHRTKIESFNSTQAIPDVANSIARRNIKNFIKLFWVLVILWNVGIGLFTIKITMKIITLLTGESDSSKVDSDSFQNSQSWRLTVDFMNAVMTLLSLPISLSLIIISRFLTLEVVTQCEIIRAAMALDTGKKVMFKGYILDHIRVIKNAKWLIKRLEAVNTANTLLSYLMFALNMFMLTLVEPALYTYGVLALYFFIDFLQLAAQGWFSTSVKIALESLSDGSYETIWYERDRSNALDVLIVSQMAQQECIQKVLFGSLRIERATAISIVRSSYSFYTLLMVLQT

>AlinOR55

MIRNWQDEEEGQSLLARMGCKFLNGHSIYVGSWVLRFPVRLPLFLYVTCAVGISIKMVLNYDNLVLIIDCAHMMIHMVVGIQTTLICTKQKGRIMKLKDQLDKFDDVPGKEASSTRIKEDYELQVMNLYRTFSRCILVTINIYILFPICKLFTEAGRAKLSKVLVWQMWLWVPEETWWGFTIIFLFELVTSLFLLLSVMYAVPYLACLGTITVAHCKVLILRLKSLQKRAEERSGKMSESYEAALNYEIDGCARRLHENLRIANEVADVYKYYLSSFYYGGMFALCMSGLQAVAANENIEESLKFMGVLTGELVAIGLATYVSEGIIEGFADVRASIYDLPWYVYPKLSCKRLHLMSTMSSFRGLRTMFGYELTLLHFGDVLNASYKYYNLLLLSMK

>AlinOR56

MLCCRKKKEKQQPVVQEQNDFVNNYRLFKYIGMIQDGSIFSRIRVPIATFLLFYAWFHHLIPLIMNYDEYSFDELMDLIHLEMVYFLWCIVWPSYIIRSPLLTSLTSKIKSGLYTYSDPLTLEEKTILSTANDAVARITKISVYVYVCGGIGIFLKGMNKEKMRKLSLPNIGWFPFAINSLSRYAIGCLCQAIMGVNAVSIAIGTFMSFAMFMIHYEAQFKLLRTHLRRSFPKNIPLRIAQTDKYKEATLRKLKDCYNHHLAILEYHEELLKYYGILLLVFRVAIVLWLCTLAYVTVIVDINAHNLLKMMSFASTELLFVFLFSFRGQDVTDWNYEWREELYSIEWWEQPKEVQTNIEVMILGTTEPLLLYGVWKIALYSHEGLFAIGNESFSFFNMLRAMN

>AlinOR57

MFARPNMEFVQTWHYYGYWNIFLGGKYDSYKKVALVVRLILFTAGAFTALAKISSDGVESVLNGALIYVPLCVYTFVIPTILFFKRKNLFSLLKVFDECWASLNDEEDRAVFEDYYQKTWKFVRIIHVGLFCQLTYYITFPILYSLVFHYIFDVLSQPYILSYPHMSYLDKNFTWNGEYYAVVFAGVYCIAEIEFALLGWVLMYVVIVGYCYPVLVVTRNAVQAVGSNRSPSSGDRAMKKAVVAHSLLVKVNQDLKAFLGLPCAFQSIFTSLCLTLSVFTTIRSSDYFVEGAYGSAIILYSLSCLLYCSLGQILENQSEKLFEDLYNLPWYQFSPLVRKDLNMMLRQAGKPFTIDFHGGYNMNLENYMEILKSAYSFFTLLETLASNT

>AlinOR58

MWDWIQLRMFNLWGWWPKVIKDPRKRVMMRIYGYCMFGLDSATMIAEFVSLYLAVVNGSFRGAIVNIVTTTLGATAAMKIYTLLVHHEFISHICNTLEDLNNRAIALMGEESQVTMGTRKRNCKLTFVFVGSCLFTVCHYNVRPILVYTLYGERTIAMDMWTPWDEQTSLSGWVIVLIYEWIHIAAAMYGMVVFDSFVLCIFEMVLAEFDVLKIALKKVDFAAEKNAVPIEFCIQFHQDLLVLIAKINDFLIPIQTFQCIMLTLTICFSGFELVSLSDVSMNKAANLLEVLGASTYITFGYCYQCHCITEECVEVITTACDNNWFEGSIKDQKSLSILLERAKNPISFGNIIKFDLGCFIAIIKTAFSYYQVLEAFDIH

>AlinOR59

MDDPGGRALEKSGLNNVMSILAGFQGPREVRFKGTKYQHVFTAYSYFGLCVSHYILICCYLTPLFITDMSFKDMMFFAVPTITSTFSTLRVYYMMWNRSKIIRLLIKNEEASEDDYYQDKQEEEIKKWANTAKVLQPILYFAVCAPIAPWGVSPIINEILGNPWGPRKATIISWYPYDVQKTHFWVLTVCLQTMAGCYATLSNVMFDAVFICIAARQLALLIHLKNSFSKIFQVFHVHLNGNVWYSNYNGEVVERKEIEDVVIQRLKYWIKKHQTVLRLLDESQSMYSFPLFLHFCYVGMVLATGSAAVLKGTLTSMEYYFIGMHLVGLSFTLFIICRIGDYIKIETDEITEGLYGQNYFMLSKEQHMLIKNILTAVKQPFVFTVAQAFPLSTETFKSVMTTTYSFFAMFTQMQHKN

>AlinOR60

MSTVVLHNDIAAYINRMKSFTVWYGSSVSYGDSPYSRYKKFYSRIKWVLFLVLLFVNGFCLYLLDDFGLFDGNFIYWPICLELFVLTSIARFSGQQQEAITMSLNDCFLKNTEPWMRAIKDKYISGMWKFVNFFALYNNVTLFLYLFGPLVADTILHYGFDYLQKPFALPMPLSPIFKYEDSWNTVHYVVTIINFWATAEVVFVFQWFFANFSLLTTFFLTELIIFKHQVKSLNFERDDSLDQQVEDIVNKHNQIIEMSKDLKGYLGLSAAFVCFITSLALTFTAYTMYSSSDLPLRVTYGSGFLLYFLGALLFSTLGQKLENECDEVFKALYGLRWYRFTPNARKSLNMIMRQARYPITIDYHSRYKMNLSNFMQILRSSYSYFTLLQSMASKSSASRL

>AlinOR61

MPSSMSFALIRMMLTGTKTFPFAMSVFGMPTFFGWVLQMLLVANAANILWGFYCVLKIVIYVLGSYSNVLAHMLRERPVDVQPKEDRQMLKLFCDINSLSVKLGNIYGLIAFIETSMASGRCCFLAYHILLAVQNSDYNNLGVPLSTFLTSVAITFALCSCGEDISKQTDTIRKGVMDSKWYAVSPANRKTLLPLLMFTQKPIQFHYKRFMYFNMETFRNVLKTTYTMTTALAQV

>AlinOR62

MSYFSFVEENILPQDLEATMFREYSYLLYIGGLIINYRPFIWIISVIQTVFFISLTIAHALIFSITTVKSSTFVSFSQNLNFASLCCIALGLYFAGHSHRESLVRLLRIMTDDFYDYGDSFDNSEVAEWKKKFRTFKLVIVIGIPVYLSIIAASVVLREYIDNAFGYRITDKPDYIGDIYQKTPVPVWYPFKITNSFTWLATTLLQMLVAAILATTLATGDILMLFLGQTVALQLRILCLATTKIDQRAQKMLKNGIGKLAPGRKADLDECYRICIKQMVEHHLIIKEFYSTYYVIAKWPTAIAFMNGSLMIAMSIIVAMTGTEESPSIYISAYLLLTAEVMSMWLLCETGQNVNTMSEKLYMVTYDCDWMGADWSTSNKKLLLIFKENIKKPLLMMAGGLTPINRDTFATIMNTSYSYVNLLRASEQRTN

>AlinOR63

MGRSVPDLPDLVRLLRFCGLYYTFNGPRAKVYENLQPIRPAFVVATSIMGIIALFVGGLRSSLGIEMCYALLGLVTSLQCVHFYLRREDTESTIELFQEIREKFQKGSEEEFKNNTRGIWMVVKVYGFLLVGTCMAMSLPFCTDLVIWAIWKTPKAFRIPMGMDSFLDKEPIRDAKYFFVMFAAGSWTIIGSIAQLGADAFLFVACFSYSSMVMTFCKSLTIHSNLTPKETTAHLKRVAAHQQQLFKLSVKMRLLFGLPFFVQNLFGAICICSLLFVISTDDSINMLKMFAYLINLLVTVSILGCTAYVGQHVKNKTSEVFEAIYEMNWYELRPSDRRYLVTMLSAAREPMSIDFYGLLPLDMENFMKILNTSYSYFMFLKSMI

>AlinOR64

MFIFIREKELERNPDNMKGRELLEARFAIFSGIYPDFYGWRYYYFVLFLIFIHPGLYCYFLYAYGISFYYGMLYADVELLGQVLCLGTITVIYCIVSIYYIARKTDMDDLITMVGKGFLNYSRGLTEQEKTIIDRMEKVTHRYAFGSTAMLMAISLVHMGLLPMIRGLKGQFTSITNETAPINKFTPLPVWMPFECNSTRSFVLTFIWQIIPGCMEYAIINACCILYVGLAQQLSGNLEILANSIRDIHTRALIMFENDGGILSKITGELYENSHFLKCINACLNENIEHHVKLIEFFNKFQGVAGFSMLAIFSGTGLIISTAAYSLLLIAQTGGDSELLITNAFVWTFYLFVYTFLLTVYCYYGQEVTDKNDALLPALYETPWLEADLPFRRSLLISMSYSQRTMELSAFGLIQSSYATLLDIIKTAFSYLNMLMAVQ

>AlinOR65

MKNLKKGTSSWRDLKGLAREEALRRGYAENGGTYVKMGAQYVATRNDIWMPVVFFSDLMLAVFQLTAAGYFSVIDGDMEAASECFHFITMISNMMIITANLIYYKNIFDDLFVATGSGFFDYGDSLDPQTKNEMEAYISNMKVKKRFRFRTFVLVVWTLGGSMLFKVALAYFRFGDRIDGEGGSVSRKHIVAQWFFGIDKWPNYIFMASVTYIAEVLVMTSVWGFVLPVICFAEESTAQLNVIGLGLKRTSARARYVFTCRFGEYKSVHKLKYEQCVREVLRASVQHHNAVLDVCNEMRTLLNLPLMTVMFNTAVLLCMSGFVMIEDSVPIIAKIISLLFMIGEVIYSYIFCLYGEMMTSTSEEIGNQLYQDNWKEVSVVIKPYLAMIKMRSSKPIRLSAGGFMEVNEAAFSGIISSSYSYFNLMLTSKS

>AlinOR66

MAETGRLKDEDLVEGLSVWYLKASGLWGIFNHYRETRGKSAVFTAWMVGSVIGFGPLFLTSAVGPFFTANDLEGMTLVILNPLSALQMAVKFTVLWFGLDTQMHLLDLMRNNYLTCVPASRQARAKDILKKAAKKANFMANMGIAANAITVSFWNIFPILRSDYVRLTLGITFFGEPKDHNNKILGFWYPVNHKETPWTQILYVYEFIICFWAGFIITLLEALIAQQVVLLAAYLEVIQYLMSELKKTQSAYLDNKTLLSFIIEHQRLMRVGDEMRQLYNFLITMTLSTGLIILIISIFNFFLGTGKGDIILTIKFVIYTMYTLVEVCVYCYAGVELETTSKEVGFAAYNCDWYVMGPDFRKTLQMMMIRTGSPVSLKAGKLYPVNLTTLTNILQVAYSTSTILFRITNKDNTGLDMV

>AlinOR67

MGYREYPKRDLSDPSHLFNFHLSALRVVTMWKLNDMKYHTPFMILFFWNVTVLSICTVGLFIKGCTTDDLVDRSEAMDIFTLTGSAMYKMVYFIYYHDDLVDMITCGLALTEKLPKGWTKHCTLFAKFHCCGGIFCMTFWGLVPMFKTLLGETTLEEMKLPINTYYPFEGRFAFSVTYSIAQYALMVSGQIYMAADIYLFTSIYVAVGALQYISDELEKMNENSNLKIADVGKVDQTHEHLKECMELHVNVLDYIRKTDKLFRSMILADVVHAIISLSFAMLQASEAKGLFEGLKMAVFVVVCFLHQFLNSHFGQTLIDKQDHLIEKIRIAVPWREASRSFKKSYHIMITSNTNAIKLSAWSAYYLQYATFLEFSKSMISYYMVLRELQDQEEVP

>AlinOR68

MSFVSNILSSWAKDEDDELWEVYHKLYGPALELSFTFPSWRRSRLPLTLLIFGYYAIIFPFHIALLSVSIDCVRDDFNMASMEFHFWMIFMFSSISILLMNSNRQSMMKLHRTLTIGVGKYKAGRIYNEDYPVMLERKKPKQTIKFLFLPGLVMVLAGLTLLIPYVKNMDGTVEYNHRGANMKLPIAACYPFPTHEGITYLLAVAGQFMAAGGLALVIATLDLLLFRMTQSVIFEYEVLIYALQTMSSRAKKLYRITYPNSNIKEVRTKDEAFQRCIGECIRDCIVHHHDIIKIVKEYMNLVKWPGLLAYGFGTGVIGLSLVNILSAKEAGNYENIILFMLLMIAEVLNMFMISSFGEAITTESKVLREQLYFIDWPKLDTQNRKMMLNFQVGINNPVILKVGGIVNVTLDTFSSIMNTSYSFFNLVNAQ

>AlinOR69

MKEEKEDNSSGCTDYRKYIFCRLMCIDDRLVDRGLTWPLLLIMAQTVAMETCSFVSIFKSTEIKNGMDCVRSFLLGNLVTMVLFNEFTHRRRLARLHEFLEKSMSTLRTGLPEEEEILKNARDQASSNLKMYMMVFFGNIAPMVFAQPLGEWLSGHSWKRLPIPWSFPPSDTDWAFIFIFLFQFVGVVMANCLGMVFMSFSSITIQVTALFDVLLLSLRHMEMRAKLKGQIEYLDYHQSLYSCLRDDVIFYEQLVRELESITPHLRNTFLAFSATVPMIMACEAYPIMLGNFAIADLIKSFVFLAIQFMCWAQTCMKLETMTDQHVAVFRTLYDTPWYEAGVRFKKLIFMSLTYASPPKYIKARMSNEITATTATFYSFVVSSFNWLNLIRKMS

>AlinOR71

MEADDGTVNEVLLNSGLINWIGIVGGIRFAQRTRFKKNFQSTIYWFYEIFTDLTVFINILSQIFALMASEKMTERCLIGFPLVSCSFCFFISNYPRFKRQEFATLVLRYDDVFPNTQYSDHLEEEIKKAAERVRLTSTILVFLELGPMFTFCLILPLVNEASGFALGPRKVAIPSLWPWDPLASFKNYIILVFIHAWASIFVNLKKIGFEESFFIFASRQVALLRHLRYNLEKLFDPLEVNVDGTVNKNEFTSPQRYWMEEKLTQWVKDHQHCLRLFYELEALYKWPICIYFGATILILCTSTFVTSDNSIDSQTCIICGVFTTGIFCELFFICRMGDHIQIETEELLLGLSGKNAFLSNWKEFKYLRMIMTRCQKESVIRAAGGFPLTISTFKSITTSSYSYYTLLKEVNGKLTE

>AlinOR72

MKEFKSKTHALLLTMLEVAAIHRNPERSMFSPNGFLIYRVGLCLSYITMITSSLLYLNEEGGNQLMKMVLSSVALQFFVCSMVLISKKESITKMLLASTEVFSQLTPNDENRIKMDLLDKKSLKFAKFFAFLISMNVISGVMKGLMNSWLSGETHYPFDLSCLGLSAVPCWFTQLFSTSGTIIYIYCYFAILKLILYQLWGYVDVLSALIRDRPVSASEKDDRDLLKFYCDYGNFAVSFTSIFGITAFIDMIFTSVRCGLISYYIVMSISENNWSDAAGSMVGLASSFILPYIVCSCGEDMDEMNNQIRNGFLESNWYQCSPQSRKRLLPILTLNNVPIKFQYRQCMHFNMERFMQVLRSSYSLTTALANFM

>AlinOR73

MAISDHQIEWLTQEEVLVFDNLTKLLAIVGTTSRSKKWYRMFSLIFWLAAYIPMIVGLTVTAVHYIKDLDFLAYTLHHLVLITVAVFVTHLVVPLFDTQILYVMNASKNTYNYESDFVENYAKKIIRKRIKLSRTITKMIYFGMVWIMLEVQVFFMIETFFLKSYQTVFPLAIGLDLNRWLVFVPVVLWQELIVYYTTFLPTTLAVLCYTAWSHLDLEMRILTYAIANVQKIVNEKLSQDGGPGSVSEIYVSYCNHFAKHHADIISEYRLAVSWITPLVFFIGAILFITVGLSLMSDNLGIKLKLFWFLVVQYIVIYFWCLISQKIGEKSEEISEVVVSTPWWLMPRSCQSTLLLIMTRCKKPLVLSTPLGADVNVESFMEMMKSVYQAISVVYQMKTTS

>AlinOR74

MGKLEISWPQLNYLAIYGLWPVSVGGIRFRKVQWLWGRLIVTLTVMVIVLEIVNLHELLMANEMDLNDFVITMNDILYATQSICILVNILVYKKQFAIMMDELGEMLDHSQLLMKSYAEHVFTKKLRVCRFAFIAFLSGTLLMAHWGISPLLNRILYHRNTFVYHVWIPFDVEPWYIYFSVLTAQITIGVSWIMGQPMFASLFISISEHLLGHFDVLRQGLETLDYSAPSASQEVNRYFEYHQKILKVGYILRSATKLVFLSQFLCVTSIMCLNLYEITYVDVETSRFLNMVEYTLLELLIIALYCSYCNELTLRGSEIMSSAYFSGWESASIQDRKSLWIFMTSTKAPLNYGGMVKMDWTTFVNILKTAFSFYNFLGAVRTSKEGQITNG

>AlinOR75

MGSLLDKFDEWAEVEDEDFWKFIWKSYGPLIQISCMFPSYRRSMLPITLLTFAVHLLLLVPHFLLLVITIYQTIVDWDLELCSLAIHFSLMIFYAIFTLFYVQYIRYAYSSQAKAMSTDIGNYISGRIYEDKWCVRAKEENWQETLRLLIGPAFLMLGSGAVLILPYTLKTIKGLDNPYGAGMVNANLPIPAWYPFPTHEGAVHWIAVLYQLVAALSVGVIMMQVLLLFLSNAQRLRFELSVVGYSMTSILKRSMKLHLRANPGLNRDEVDMWDPKFQSVVESCLRDSLLHHQTILELLSLFTTQTDTLVLLGYSVGTASIGMSLFNILRALNTQSYESIMLFTIMIMGETLVMYILSYIGESITSQTSDLRNQLYYSPWNNFDLRNRKIFLNFHTAITEPIVITAGGLIPISLDTFSSIMNTAYSCFNLLNTQPEI

>AlinOR76

MVNMQRVREVEDVKTRSGLSKLIGPCGGYRGPLYNEYYSKNIFFRAYVHFTDLAVIINYITMIAAAIHSKSVLEFVMVGFPISAESLSLFLSYYSGYKNKEMTNVLLGFDDCFDDDPYPEHLESEINKAAKYYYHFSRTLLWLQVFTMEIYCFIFPVTNELMQDYFRPRALPLPSLYPCDWKESRSCFIMIIFIHFLGATYVNWKIIAFGEVFFAMVSRQVALFRHLNHNLNKILTAIQVSENGVILYRLNKEADHMHIKKALRKWIKHHQSVMAQYDRLQALYSWPLFVHFGLVSGALCCSAYATSDETLDFDANLLCGGFLMGQMLELFYLCRIGDWITIETNELTLALTGSFTSVLDRIESQMLRIILSRVHRPSVMRAVGLYPLNTATLKMLIQSTYSYYTMLKKVNKG

>AlinOR77

MDTLIRKNNIDKYLPWHHPDLTKVWRWPIARYLNTFGWWSEDYTRPSVKFLFRVIKFTFLAVNVVYLISLTIGVHRQLQSTTNFMDNMFAFFAATPSYLGAAKIFALIIQRRELRRIWKNLDDLLKDVLKRGVDDKLDRELRWRMKRCWLMYSIFLTVGSCITLHWLLRPLVYAMYGERTSIVSTWPSYLENWPLWFATYVFQAMNISSVGHALYIYDNVYFCICENILIHFAIVKHHLHEMDISTGKPGGITMNFCISHHIRLTSVCMDLRECSKYVIMQQVFWTIFIICPAVFELISGRQTDTTIVVNLLEITTIMTCILFLYSWYSNEVTLQSSQIYNTCYMSDWVQGTASQRKTLMTMMTRSMKPIIFGGLVNVDLGTFISVLKTTFSYYQFLDTMDKNKRKLDDTS

>AlinOR78

MSRSGRLKEEDILDGLSLRYLKVSRLWGILNHHRETGGRSKVLKAWAIGMVIAFTPLLMVTVVGPIFTEKDLEGMTVIFLNPIATVQMIFKFGIFWFHMEQQTKLLDLMRKDYLSCVPADQKAAVSEIMINAVKDANIYTFFGSRMNIITVSLWNILPVLRSEFFRLTLGISIFGTPVDHNKILGFWYPFDFDGTPGNEIVYVYEFILCIWTGFIITLLECLIAQLVVLLTGHLKAFQYLMENFRSSHDPRMDHRKLLSYVKEHQKLSRAGESICELYNFLITVQLATGLVILILGAFNFILGSGKGDVVVMIKFVVYTMYTLVEVTVYCNAGQNLETTSEDVCFAAYSCDWYEMGVDFRKTLQMMMVRSQSPIVMKAGKLYPVNLTALNNILHMAYSTSMLLYRIKVPEEKQVM

>AlinOR79

MSGIGRIGDDEIVNGLDIWYLKCSGLWDVFNDYRDSGARNKLFRIWMVITVMFFAPLAFLSTFGPFFVEADLEGLTLIILNPMSCSQTVIKFAVLWYGIETQCKLLDLFKNDFLTCVPPDKKLKASRILTASAKKANILAYLGIFMDAATVAVWNILPILRSEFFRVQLGITAFGTPMKHNKILGFWYPVDYDVAPYVQFVYCYEFFTCFWAGFIIALLEGLIVQLILLLTANIRVLQYLLEEIKASNSNLNSETLLLYAKEYQKLLVVGDDMRHLYNSLITMQLSTGLIILIITIFNFFLSSGNGDIVIMFKFVIYLMYTLVEVALYCYVGSDLETTGDEIGFATYCSEWYKVGVKFRKTLQMMMVRSRYSMAIKFGRLYPVNLMALTNILQMAYSTSMLLYRITNKEEENRV

>AlinOR80

MSVEKNLIKSKAFKDNFGQYMVWSGIYCGNNVYPGCVLIFYIVHTFFLLYTLILSISKDNEKLMGETAHFTAFRFSAILLLINGLWKKEDLEALFLKLCQPQVHDYGNTLSDQCRKDIEGARQNCKARKDFYGPNFMRTVTVALVIFWIRSLMEYFNGHLDNPKSDDGINSNLPVPTYLPYESHEWPGYHFALVCEVLLVMMSYFLVLGHDCSFICFAEEILRELDILIITLTEVERRIDHVRKSSNYKISQEESVRLCLKHSVMHHQKVIHIFEHFQHYCFHSLFFMLSGGAFLICLSSLMFTSDTISLRDKSVFLMFLGNELFHIFIFCYYGEHIMGKSDDVGNSLYNSSWVGISKHVKPTFMLLNLRCQVPLTLSAGGFMTASFDTYGNVLRTAYSYLNLLQATN

>AlinOR81

MDRPSDPTRSNGPKFKAFDRLEKWQRSGYKLLLYGMSAKRFMKSSSQRAVVYLVVVMTLGLYSLHELIAALFFSRSLLEGISHAYIMTYILTFLIQWFYMLQHVRSFHENEIYLEKFESTQAHSSFADHILDRNMKDFLKYIMFCAGWWATNNFTHLIGPLIEFSLAFIRTGEFIQISLLPQVFDLPKWGQVAMYIHNAVMTFSFFMYCGANSLILGTRVLKVKTQCDILNEALRNDHGVESNIKAFIKDHIVILRAAKMLNGELADLNLVIFTGSYMEIATQMFTLTLFEPSGVYFFAVAFDFGSIFLITATQCWLSSTLTIALDTVSDGVYDTEWYGRDKNHALNVLIMLQMAQQEKSHRIWFNSFKIDRSAALSLLQSSYAVYTFLMIVQSN

>AlinOR82

MNWFGWWAEEMDRPLVVRLLQIMRAMLIPSHMIFYATLLYQTSHEFREGTIISTVKSAFVSGPSTVACFKLYVIVRHRKSLKEITNSMDVMMKGILSRHIPADLEEEMKSRWGSCRKLYKVCVYFGCSVTTHASVTPLLQTIAGALLTDDPLPFDSWPYFLLGYYCWALNTFCIGHVLYMFDATWFAMADNLQIHFVVLKNYLENLDLTKQGDVDLNLCLKNHIELIRLCRIFRRISRTVIVTTRMCSMLLLCAGTFVLTSAGSEFTPNDRGNLLSTLIYIAAVFFNYCRCADNIAHQLDELTTDCYSAKWVHADKSQKNSILNMMTITRMEPKFCGIASIDLDTFVNVMRGVYSYYNFLTAVDVGDESGEPNADSNTPL

>AlinOR83

MSKQSGPLDNIVDYRKHLSYTMTFTESSQGKLSKPKVIGTAIMMFIIPLGCFVSVVKSEELKESLENFKGVMAEVMMISVLLNETIFGPEREERLFEYLSKCHMTTRYGLAEEHTIIRNAGDSARKETFVYALLFIVNCPLMLLTKPVMAAIAGQSWKQLPLPWTLPEGDDVVLNAVLLLQIVGLLISHVVAIVMMSVLAITTQLNAQFDVVILGLRRIEDLAAQKATEYGLSHSDSMLSCIKESVAHHQELIRELLLVKPHLETEFFCQIMTISIIMACEVYPLIKKDLELSDAVRGIWFLIVQVLCTALICNKMEVMSNKNVEVCDALYNTCWYDCDIRYRRVVLNAITFSQNPIPIRGKGFLGMKACRETFYSAMVSTYNMLNMLRNAQ

>AlinOR84

MTKKIYEKPERIYWDSLGFSQLSVFLDLNPPIDSSKFLWHLKRKTAQFFTFIFSPIFISVHIWSILSASPYNLKQISFDLGFLSHNVQNLIKMFFWLTNLTSVRDLCLDFSQFHVNRYRPLLSSWVLQKESNVTRKYIERCFLISKGGLIIWVFIPTSIALINYFLYVSGLSAGQETYIPRLSVTQFPFNMSYLGNRLFVGTLEYGQMISVFIYYQPIDMFLIAAVNMVRTQYLILNFGLFWMPKDLEAFWGTEVSVKDTEPPMDMRLFVEDHQRLVRYGQKLREVLNPVLGIVTFDCIILMCNCAIFITKLLEDQLELLELVLYMTAYITIILASLVVFYTFSSMSGMLKEAEESVFDALYAQDWYRMSTQKKRSMIFIRKQTITARKIPMLNLGDMDRATFIEGLRAVYTFYHFAKQFK

>AlinOR85

MEEENKARKNKGDNLGCVDYRKYTFARLIMIDDGLAERGLTLPLLTIMVINVCMESCSFISIFLSTQASVSLNNVRSFLLGSSVTLSLFNQYISRKEIARLHDFFEKSFRSFRTDFPEERTILDDARKAATAQLDLYVKIFACNTVAMIFAQPLGGLLSGNSWKKLPVDWTFPPSDNEFSFWLIFLFQVTGVCIAHCVGIMIMSFTSITIQMTALFDVLVFCIENVEKRATTRSQQTGRPYHPSLLACLKDDVAHYQQLIRELSSATPYLRNTVLIISAAVPMVMACEAYPLMQGNIILGDLVKSFLFLSIQFLCWAQTCSRLETMTDRHDAVFNALYDSPWFDTGFEYRKLVFNTMTFSTHPKYIKARLSNEVTATMATFYSFVMSSFNLLNMIRNIG

>AlinOR86

MKTVLVHEDIAKFIHFMKARTMWYGTTVPWDDSLFDRFRKSYTDLKWLFCLLFFFMCGFSLSFADNFGLFDGTFIYWPICFMMTLLTSIAKFTARKQDVLMTSLNDHFLRNTEPWMRSIKDDYIKSLWKFIHFFSSYQLLVSILYMVVPFVADLILHYGFDYLESPISMPTPLSPIIKYNNAWNVKHFAVTVVNLWAFVEVVLIVQWFIANFSLTTVFVLTELIIFKHQVKSLNFEDEDNWEQQVKNIVDKHNQMIRLCKDLKDYLGLSSALVCFFTSLVLTFTTFTMYASSDITLRLSYGCGFSLYFSSALLNSYLGQKLENESDEVFKALYGLRWYRFKPEARKSLNMMMRQARDPLIIDFHGRYKMNLSNFIS

>AlinORco

MQKVKMHGLVGDLWPNIRLMQLTGHWLLEYHEETGGMARLIRIAYCWMTTFVVYLQYAFLVCFLILETYNSDEMAAVTITTLFFLHSVTKFTYFAIRSKYFYRTLSAWNQVNSHPLFAESNARHRAAALSRMRKLLMIIGVVTILAVFGWTTVTFLDDPVWDKTDPDNVNETISVEIPQLMVYAWYPWDAKTGMTYFMTFALQLYWLFITLAHSNLLDVLFCCFVIFSCEQLKHLKEILQPLMELSAALDSVVPNSGDLFKSGSAGSNIALISNGDGGNDFDVRGIYSSQRDFSGFQGGMTNGTTVGPNGLTKRQELLVRSAIKYWVERHRHVVKFVTSIGDTYGTALLLHMLTSTVTLTLLAYQATKIEGVDVYASTTIGYLVYTLGQVFVFCIHGNELIEESSSVMEAAYSCHWYDGSEEAKTFVQIVCQQCQKSLTVSGAKFFTVSLDLFASVFGAVVTYFMVLVQLK

>CnigOR1

ASITEDNDGSRKLLYFQLVENVRHHQAIMKCFQLLELVLNYSISILLLTNILTVCFSIFFASVMLQREGGLRRAMKITSSIPNLLIETGMFCIFGQMVVDQSERLPRSAYSCGWVDADARFKRALLIFLMRTSQPLEFTVGKLIKLSKETFLKILNSSYTLISLLYQFQESND

>CnigOR2

TRQNTFHTASLWSSRPALEMQERKTKRAGGISVSDVLRHNVLLLMVTGAWPPAGSSWWRLLYPVYTATIYFSMLATIFMGFLFAYQSWGDWDSIMLTFVNTFTLI

>CnigOR3

EQPAGEYVAAVMTAAMSSAPQPRSSVGAAAAAASDMRHLLRPLHWTGVLRHPRFAGRSPLLFRLYTASLASFALVFICSETAALIHEGTADMDVVILLLSTINTASIWIFRMVHIAVFERDFHKLALQVGHDFGEFLTWDDIPVIRSSCRVVRRFTLTVVWFGVTACSYFLVTPVSEEGLPFILAL

>CnigOR4

FLDSVMNPIALTQFMLSVMAACVTLFLESYSADGSSVLNSVSYLPTPGIQVYLYCWSAHNVLEEGAAVSEAAYGCAWYAGGGRFKRALRTVMCRAQKPLAVTAGKLYPINRATFVSLVNASYTYYALLSRVHNRDAIKI

>CnigOR5

IVAAFALTAATALLQLCLETPQDLEELSMAVFVCTGATAAGAKVVLFMTHRERTQRLALRLLEARRLFPEDRTGARTQYQRTAERLGLGYQVSVMVPALLWVLTPALVDALSAPAANGTAVPRGTPLPLWAPFDWQRSPNYELVYAVQAVTNVVINLTGLLLDVFFVTLMLLVTAELRVLNLNMAALRPSPAAPDDGLQGLSDHSVVKRGSRSHMYTLLVKNIRHHQLIIMCVEDLEQVMSQSIFIILLTNVINICSQAFASAVLFRRGIAWGLVTKMLFTFVAYMFETGMFCMFGQAITDQSERLVESAGGCDWLEAEVEVRRALLVVLAQASRPLTVTVGKVAVLSRASFLQLLNMSYTVFNVLFELQSAD

>CnigOR6

AEAEVCSQPAEAPLAHSEFAPPTEVAGMDDPAVDGASLLGPGATVRRLLGLWQPRGRVARILTRPFTGFILFAHAFVPICVVIKMYMDPPEELEQITFGGLIICLCVGFFVKATLFVVQGETMRQTAQLLADIRGQFGDRQENERTRRRYEKLSNRVYQRYQMVEVPAVIGWILCPLLSRSVTVSEDGQQVVHRLLPVPAWLPVDVHASPTYELLYVADAFCVVASAESSACIDMFFIHMMLMVAAELE

>CnigOR7

MAASRPVPAAAAADSSDLDYLLSFLHWTAVMRHPRGSATSPFWFRLYTLAMFTLDLSFVCSTFTVLFREGTEDLDVFTLTLSVADTNGTWLFRMAHTVACEGAFHTLSHQVGHDFAE

>CnigOR8

LLAAQLEIVAGRVERLRAGASTPLKEKDGDCKKCTAAKHADRMYGDMRLCIESHQKILSFVAHLQDTMSPIAMTQFAFSVLVICFGLFQATYSEDLSAVLKCASFLPIPCGQVYLYCWAANNVTIQAEAVTMAAYSCSWVEASGRFKRALRILISRAQKPLVLTAGHLYPIDREAFLSLVNASYSYYALLSQMNNR

>CnigOR9

MAPADTEMQTLLGSGASIRSLMGLWRPEERRGRVRSAVSAAVSLGSLAWVPTFTGLKIIIDPPPELEEIAMCYLLFFACIGFFSKAAFFIYKGGTIRQLLLLLSETRRLYRNGESNDAIRRSYEKQSRRLYLSMQGAIWIAFIFWVSTPVLVRAFLTSDDGSPESYRLFPGPIWFPGNIYISPTYEILYGVQSFGMLVSSQSTVCIDSFFFHLMMMIAAELEVLNENIARLEKIKLKTKQYEDQEWRVNVKENTDDLTLLNNGNFTRKALSEEVSDEKMCAELVNNIQHHQAVLRSVLLLQDIMDVSIFILLFVNMVALCSCIFVSAVMLQRDGNVTKALKPLSTIPTVLFETGMYCIFGQILSDQSEKLTVSISCGWVDCNDRFKRDFLLLLISAGKPLEITVGKMSKLSKQMLVQVL

>CnigOR10

FFEKRFARVFELAEVHVTEEYLQQRKRRAISLSSACQGRGQPTQQQKRKSARSLRGATMEHPSADSGSMVVPGATIRRLMGLWRPPDGASGVPSRLLAAMTLTSIMVLATGVTLKLCGDPPKELEQMTLCLLVGSICVAFFCKATMFLAQADTLRQAVRLMEDMRAHFCSGGHHNKLTRRRYVKLSYNVYYYYQTMGVAAVLGWAIVPLVKTDQDHLATRRMLPMPIWSPVDIYTSPTYEFFFIFQNVASLVTSQSCLTIDIFFVHMMLMVAAELDVLNYNLSAMEHYSWQTTISDGDISSLKRNDIRSQLLDGGHSIDEQPLKEDAARKGLHQQLVKNVLHHQAILRSVSLLQSGMNVPIFVLLFLNTANLCSSLFAAAVLLQRDGNAAKAVNALFCVPAIVYQTTMYCIYAHNMTDQSERLMSSAYSCSWTNSDARFKRSLLIFMMATVRPIEITVGKMCTLSKEMLVQVLNGSYALLNLLYHFH

>CnigOR11

VGHDFAEFVTWDDLRLLRSQSRAVRRFSTACIWGSVAACAYFLVSPACPEGLPYVLVLPFDAFHPLGFAIAWLFCTVTCLHTVVMTMTLDVLNISLIAQLRMQLTLLNSKILNLAKGAPGGTLDTSDTDTCREIHSGLEKCIRHHQAIIKNAELLEATLGPMLLGQSISIGASTCFQMFQLATTANGKQVGKSACYLYAMFAELFVYCWFGDALITESENVALAAYDAVTQLQGCPTSIKRSMLLLMHRAQRPLRITARGFFPLCRESFVSVVNVSYSFFAILRNFRKEE

>CnigOR12

QLSFTAYTTLTTFGLVAKLVSFSLDGARLTQLLQLLLECRGRFPDIGGRRGQHHLMATRLHRFLQVSYRVNSVMWMFAPIVSAVIAARSRGDAPVKRTYVLPLWLPFDTQSSPTYEAVYVVQLATGWTLSETTVLLDGALLALILHAAGELTVLNDSLCSNAMNDDPEVPALAADPGTPLRYPKDDTATSRDLKQDHMFRQLVNNIQHHQIIITYTDLLQKVLSRAISVLLACNTVSICFHIIATVALLQEDIELVGMTKMVVGSTLYAYQTAILCLLGQRITTQSERLSASAYSSVWWEGDSRYRKLLLVFCERSSRALSIRVCGLYSLSKETLLQVLKAAYSLFNFMYQAVETSNH

>CnigOR13

MWNGLDPAGGRLKGWVVWAMVAFITVTGAGKLCFDTPAQLADVADYGYFVCHLSAVTMKVAFFILQRGTIQELVMQLAYTRKTYGRTEANHRVRDMYRRRATKVYFILQVLVLAIVSLWVSAPLLQDIRGPHPIWFPAHSPAFEIVFMVQMVSGTTATELAILLDTSYYKLMLMVTAELQVLNDNMAVLGRADESDYLKGISAADVPRKERDVAIPPTKQTPVPATEDNDLTRRLLYLQLVENLRHHQAIIKCFDLLQSMLTYSVTIILASNVFTVCFSIFIASVVFQSDGGLKRIKTLSGIPSVLGETGMLCIFGQMIVNQSERLRVSAYSCGWPDADARLKRALLIFTMRTSQPLQITVGKLIKLTSETFLQILNSSYTLINLLYQFQGPKD

>CnigOR14

MVVLEASDRQTLGESGEPWDVLRRNVKVLRFGGVWRPASRSGWRPCLFPLYFASVCGSLLNIITLDMVRSWLLWGDMTAVTFALVSAMTNLNGVVKMVHCFRHHDTYSRLVSDLNGLVALQRRYCERNGALKAAFQRASRRAARLTVGCLAYMNVLGQMWCVVPLLSAEPPDARESALPLVSLPGLRSDNRACYSLAYLVESHAVFYWNFASLGMDMFFASAMIHVTGQLNILNIRLAQLRREGSTEDQLQPSRSTLNGNNQQQERLAGDSATMYGELCECVKHHQAILRYLEFLESVMSPVALTQFLCSVVAVCVTLYQITFNPEGSGVIKCAMFLPIPALQMFVYCWCGHDIMEAGLSVSLAAYSCAWVGVGQRVTSALRVVMCRAQRPLQLT

>CnigOR15

GAAVGGAGQQKAPSGQCSEPELYRELVACIRHHQNIIAYVDVLQQVLSPVALAQFMCSMVIICLSGFGIAISNDFGTLFRYSVYFTGAAIQLLLFCWYGEVLITKSERVSEAAMGCGWTEARGRRFTSSALLMMIRAQRPLALTGSKFYVVSLKTFVQLLNASYSFFAVLRQLNESGHREDDGVLDSL

>CnigOR16

MGWDSREDQPLTWQYTGDSVLKYDVRILHIIGVWPLRGSKLYRCLVTVIITLCLGHFVEAVINLYTLHGDLEDFTLALSNVSVVIVGILKVSFFLRHERGYCRLVRWLDALVASQRVYTRGRPQLEHVFTGAQSLATRITRGFCMYNASVVLAWVLAPIAAPPEAKRLPFQQLPFAEGSPFSLYALSYAMQGVSMLLIALISVQLDCFFTAAMIHAASQLKILSSRISDLQLGNGDLRLQDDASLDSMYEELRLCIHTHQEITRFVEHLENVMNPIAMMQLAVGVFNGCMLIFPATYSAENDALVKCLAAAPTISAQLLLYCLGAHSVREQGEAVPLAAYSCGWADASAGFRRSLLVVMARAQKPLTLTA

>CnigOR17

YYELSYTLQCVSGLYMSQISFGLDCLFASIMILVAAQLKILSGRLFKLNEEVVPAEGSGSVLRRDQMAMDKDHDKMYERLCFCIDSHQKILRFVTHLQDTMSPVAMTQFASSVVIACMALFQATYGEDMSAALKCASYLPIPGGQVYLYCWAAHSVTESGESVSTAAYSCSWVEGNARFKHALRTLIVRAQKPLVLTAGHLYPIDRGAFLSLVNASYSYYALLGQMNKRSMKDSVQKN

>CnigOR18

MERYQQKEMVKTLSWRESGKSVLHLNIRHLWFFGVWTLGQCPGFKVYTSFAIAMGVWSVAECLLAVYFTWGQLGETTLVLIFTSTCGCGIVKTLFFVRDEQRYRRMVQQVANLLALQNEAICKDPALVAILQDSRRRVFRLTLGMLLFMF

>CnigOR19

EASERFKRTMRIIISRAQKPLVLTAGHLYPIDREAFLTLVNASYSYYALLSQMKNRQKLN

>CnigOR20

PFPTDMDHGQEEDLGKILSWSESGRSILKLNIRHLCLFGLWPLYEFRLYHLYTVYGYILGLWNLTECLLTIYFSWGDMDEITLVMLSSVSNLNGFIQMMFFVYDRRLYNALARRVAALLSVQSDVCSRETALAVILRRSQKRAFRLTLALLLFMLFQCFVWFPMPKFVHPELKLLPFAQHAWDNNTNYYGLSYFAQCAGGLWMTQMSFGIDCLLASIMILLAGQLDILAKRIVALKNGVCKEIVEYSDKGPATRFVDKMYDDLCLCVESHQQILSFVPHLQKTMNPVAMTTFGCSVLIICLGLFQSTFSEDFSVVFKCASFIPIPCSRLFLYCWAAHNVTEQAAAVSAAAYGCSWVEASERF

>CnigOR21

VQRPAGRHARLHSAQRGGHSVGGGSRPDGPRRGGAAAAEGVAAPGAGGAGAAAGAAVPSAGAAGGHAGRAGQHRRPHTPGGRHGGRRPGAPRHRLHLHRRHHHRHDGQDLVVHGAARTHAAAAVAAAGDAPALPARRHGGAAAGPRAGGHALLHPAGERGGGGGGVVCAAAAVWRRAPAAAARVVAVRRDRVALLRGGVRRPVAEPAAGAADLAVPQHLLLRAHAAPRRRARRPLRQRGRRRLAAPLQGDGPPRTGPRVRRPVGEPPARGQRPPSPAHHTGSQRAAADNEHLSLHPPVRQHDQRLLTHLRYFCGAAGDWRHGRGGVHRVQPGRLPQRHRPLLHHRTHHH

>CnigOR22

VPLALWAANFVKAICLVLQQDTLQELVTLLAEAKKKNVIDVQHNEEIRSQYGRTSVRLYRYLQVMIVVSSIAWLFVTVLIRVITAGSTNIEWPTPIPIWLPLDMQLSPTYELIYLAQVWCAVITAGTMLGVDTLFFHLTLMIVAELQVLNDNVSVVGRKTSRDKEEIVFRISGAGERRKDRPDCVTSRLDDGGVSLANSRETVTAEENYLDLIEIIQHHQIIIKMVSLLQTIMDYSVSVLLLTNVLDMCFLIFTMSELLHHEKGLHAVLQTILSLPCLLCESGAFCMFGQMIIDQSENLVQAAFSCEWLAAGIRFRKPLYTFMLMATHPLQIKLGGATKLSRSTFLQALHGSYSLINLLYHSRRPGG

>CnigOR23

MSWGSEEVEQLTWKYSAKSVLKCNIRILHLVGLWPLTESLLFRSSTAIIVMLCVAHIAEAAVNLCTLHGDLQDFTLALSNVSVVCVGVLKLTFFLRHERSYCRLVRSLDSLVDSQREYVQGQPALEARFKATQKRTVRVTIGFLVYAISQLVAWSFAPMIAAPGTRRLPFQQLPLTDATAFLVYELSYAMQVVSIIFIALINCQMDCFFMATMLHTAAQLRILSARIKSLKLQNEDVPAIFSSGGEEGTQTSAMQDSTYNNLCLCIKTHQQLVRFVRHLDRVMSPIAMMQLGLGVFNGCMLIFPAAYSAESDALVKCLAAAPTISTQLLLYCMGAHDIREQGELMSLAAYSCGWPDLNVSFRKALLVVMARAEKPLRLTAGGIYPIQRATFLSL

>CnigOR24

RNARAGAAAMAAQVLLWLAGCLCIGPHALLLDISYPVDGLVAGRARQVLLLLDQAAVAFNHLCSVASFSTMFAHFVYIACQHLQRSMDDLTADNCDIAAVVRHHQQILRYIKGIEDVYCIIMLWVFLPMMAVMCLIMFVVLKMTSVDIEFLEMLAFFLIYFITNGVISICGSMLTSKAERVVLAAYSSGWPERGRGFSGAVRVVMVRFLQPAQLTVAKFVPLSINTFSKLLQESFSYLMVMLSLVNEKDSEAHPAVVPTDVSNHSAHY

>CnigOR25

MYGVPGKGLTSLPGPNSVINVNIGVLKVAGLWPTRPYGLFAAYTVWIYLTQWAVFALDFMSLFYYWGNLNMITAVFCNLTSITAGIIKMTHFFVYKPKYYMLVNKLDTMVASQQQITDPNVNSKSILVQTSKLNKYSTYIIVTYGNLVGVPWIILPFVMDSGDTERTLPVVEWYGITQDKSPVFEIGYVLQCLTIMYWFFASWGLDLFFGSLMIHLAGQFKILNNRIASVGRKVDTGLETSSPGETERKMTKRNEVSKRTDVQQQESALYSDLRRCIKDHQEMISFMTDLEETVNFVVLVQFMAGTLVICVNLFQAALNVQDFSSVLKVCMYMFELILQLFIYCWCAHDVMVESERLSTSAYFSEWTAAPRRFTTALHILMARAHKPLTISAGRIYTINRSTFVSLINASYSYYAILRQMSDR

>CnigOR26

LPQLCCGQPVIVLTSAREAAVCGMETADQLLGPSASALRLLGVWPGTASTSAAVGTCWVGGCYLALVAFAASQLLAEPWRPLEELAIAALIFVTSVSFTFKGAMFLKYRPRLLKLATLLCGGGLRPAVGTETRRRYRERGRKVFIYLQAILSVPTLLWALQPLLVPPGGSAATLSDATAAVRMRSTPLPMWLPPALQRSPAYELVYGLQVASMLVVLQTSVFTGVFFLVLMLSIAAELHILNDSVAGISSQVSSFSRRPLSSFMMEIHCASEADPMNDSVVANFERRLFLQQMYCDLAKNIRHHQLIIKCVKELEAIMNLPIFVLLFLHMVNICAQIFVTSLLLQKDNDSTTMFKVLFTLPIYLYETGLYCVFGQIIIDQSDRLSNSAYSGAWLQGDVTFRRAMLLLTSRATRPLTLTVGKTYTLSRHT

>CnigOR27

GGSGDRYTLIKVSSNNKMKDEEGRRSVSDSSALTWDYTKKSLLWLNVRLLWAFGVWPLSSSRFRYVTKFILFILAVGNALENTLGVLGNLGDMEGVTYALMNGFTIAAGVAKTFHFHLFQDRYFLLVRRLDNLVLDQREYIEGDPEMLAVTRRCRKTALRITVAAFTYLTALCLIWALSPVVVHPGERWLPFIRFPLEPAPVPFYYELSYAIQTASSLLYVQVSFGFDFFFTVVMILITEQLIILNARLAKLHLYAGGVKSTATRVLRMAAREDRDEMYEELCLCVDTHKEIMRLVSFLDSVMNPIVLTQFTLSVMAACLTLYQQTYSPDGNTVMNSASYLPTPAVQVFVYCWGAHSILEQADAVSAAAYSCAWFDGSPRFKRALRMVMCRAQKPLVVTAGKLYPISRATFLSLVNASYSYFTLLSHIHNR

>CnigOR28

VTEKNTLNNGRGTAFSCVGVSGPETDDAATEVSSPASRAGAGMEDPSGVMKILLGPGDSIRRLLGLWQSPGRAGCLLSGAMAGVTLLCILFLVMCASLRLCVDTPTELEQITLCCLVSTVSTGFFVRATWFMAQGGKMREVLRLLADTRAQFCRGHHNEVTRRKYHTLSLNISYKAQLVVVPAVIAWTFCPLLSHLFTKTEQGQQQVQRQLPVPVWLPVDIHETPAFECLYVAQAVAVLVGCESCLSVDIFFVHLMLMVAAEVEVLNDNIAAMKHINMQNLGAEREEFISKYKRNGRQLSLTGGQSLSEQIFTEDVANEWLHQQLVTNVLHHRAILRCVSLLQSAMNVSIFSMLFVNMANICSSLFVASVLFQKERSVGKALNALLTIPGQLYEVFIYCIYGQLMTEQSEKLMYSAFSCGWVNSDANFKRKLIIFMMVTRTPIMITVGKTCTLSKQMLIQVLNGTYGLLNM

>CnigOR29

VVLIGHAAFREIRRWWLASRMEPATSAARDAEEATSLDYLLWPLHWTGAMCHPKAGPWACRVAYLLRFLAVAAMSYADGCDVVAVCQEGISDFDRFTITLSVFETGNTWVYRRCHLTWNERNFQKFAQQVRDDFGEFMTPDDIPVLRTLADPLRRFVTAYLMMGVFSVVVWLSCPTRGEGLPLLIFLPFQTDHSFGWFTGWLICAYITADAVIVNFMVDCLNVCLMEQLRTQQIVLRKHIAELGNEYGSTEYSLLKKDSGFEFGNNFAKYHDSSKPQGADYQKLHTKASFEKEGAQKSQLPTEAANSSDIHSRLRGIIHHHQAILRNGESLQKCLGNMLLVQSLSLGSVLCIILVQISLSAQGARETTKICGYLFIILGELLLYCWFGDKLTSESDNLTLAIYDAVTSLDESPTSIKRSLLLLLLRSQRPLCITAAGFFPLSKESFVSILNISYSCFTVLRNFKEE

>CnigOR30

MVAIGSRAKPILVQQSKSTRKCKDAGDESGRSPSENVLRRNIRILRMAAVWRPTGRWRSRVYPLYFGSVCASMLHIGALAVLRSATVWGNMTEVTFALVSGLTCFNGAVKMIHHYTHSEAYYRLADELNILIDRQRPHCEGDAELDEALQSAYRKAKRLTWGVLLYMFVLGQMWCIVPLFMQFPPDDPSSPLPLVTITRVHKVHNHTLYSLAYLSECHTVLYWNWSSLGMDVFFGSIMIHVTGQLNILNIRLSRLRGGGAGEDLPQYSSFGKGGEQLKGDIRDSGSMYAELCECVKDHQEILRYLDFLENLMNPVPLAQFLLCVGGICLTLYQITFNPDDGGVIECILFLPIPALQIFIYCWAGHGIMEESEYISFAAYSCRWGGADRKVTDALRIMMSRAQRATMLTAGKVHPINRDTFLSLLNASYSFYTLLRQMKNLEEENEASS

>CnigOR31

GAVREADVAGARGRRAPTLLQTLRGDVTTPSHYLGFNVWLCRLVGLLPRPGQPLWATVQPLVWCCLLMHLMCELADIALNIADVQQLGKNLPVSSLVGGSWYRLSYFTLRRNSYWRLVSKVGESFHRGAPGRMRGWLRRSRKYTLVYFVYGTIVCLFWLGHPLLLQQTTHTMFSTNSTNHSRMSETAEFPSGAWYPFDVRERRVYGAVYAFQCLAIYFAGMLIMVTDIMFITLMLLTCGQFEELGDKLRNCWEIAATRALSRAGTTQERELHKVLAHCVRYHDMLLRIVGEIEDLHWTSMLVNFVLQLIILSFSAFEATASADLASPLKGTNLFTYLILAIFQLFLLCRCGDKLMEAEEAVSRAAYEAQWFEAPQSAKRSLAIIVMRARLPQRVTVGKVVALNLVTFSETLSRAFSYFTVLRQIRTSN

>CnigOR32

VSRLRSVLSSTSGASTNYLQKVGSASMKEEEIPWSDTCLWLIARVLALGGSWRPPHVRGFALYRAWVLFTQFTFLYGQMRGVFSFWGDATRVIQDVCLLVTTILGLFKFFVFVVKQEDVFRIVRTIDDRRRAQSKLGNPRVASILEESYRSARTITVYMAGLGGTAPAVWAVMPLVMRRLGVGPPDRELPAMAWYTGRDTTTPVYELLYVLQYFSMQYSYFAAMCLDLFFACLIIHVAAQLEVLNVRLSQIREDYYRNEQTRHPREDTKVENAEEDSAWEELSECVEHHKDAIKLVDDLETLVNPIILSQFMGATIIICVTLFLITTNKQHFVALVRLQAYLAVVVYEIFMYCWFGDDVMYQNSRLVGSVYKCGWPGAPQRLQKALIIVLLRAQRPLGVTAGKFYRVSRETFVSLMKASYSYYALLNQMNN

>CnigOR33

VLQMEKGEDGLAWPLSSVVGELGLLGLWVPPPGRTLLHRLLLGFVMVSHIALLFMVNGSFIVNTPSDLPQISFVAYNCLTDIGLTSKMFSFSLDWDRVTELLRQLEASRRRFPDTGGHRARYHATAVKIHRFMQVTYRVNSAYWFVAPIIRIMTASSSDAGPPVRRDLPIPMWVPFDTQPSPVYELLYCLQLAFGWAVSETTVLVDGSLIALILQVSAELAVLNDNLAAEAGSHPQRLPVSEGDNKDSKANSPKKSPPLIEKPLPDSLQSRTADRDSSNDETYQKLLFNVQHHHAIIGCVDLLQKVLSRATSVLLVCNTISICFQVIATAVLLQEDGDAMQTLKMLMGSTLYAYQVALFCLLGQRIINQSERLPRSAVSGDWPEADIRSWRLVLLLTLGSSRAIKLKICGLYTLSRETLLQILNVSYSLLNFIYQTKSEQSTNQEH

>CnigOR34

AGAAEAALPPGRAGRLLADSARLVRRSAAAFYACGHLMMLAWYASPLIANAALARDPTTNATLPRQLLFDAWFPFDPAPSPNYELALVFQSVTLYIAFITTAVIDVFYVSVMVYLGVELEILNEAVARSCRPPQEKDKDKDEGELGDECSLLAACVRHHQHLNRCVQTLQEVMGISIFVQFVFNMLLICVYAFVLTTTKSDFGTLVKFAMTLESYLFENLLYCWFGNNLIEQSERLPFSAYSSAWPDGGRKFQQSLRLLALRAGRPLQVTVGSLYTLSRHTFLHLLNGSYSLFAVLHHLNSK

>CnigOR35

FFSGDNNELTRRRYQKLSRNIYYYGQMVAVPAAIGWVTCPLLSRILAKTDQDHHEVQRQFPVPVWFPFDVYASPIFEYMYAVQSFCVLVVAECCISTDIFFVHTMLMVAAELEVLNSNLSTLGHNNLQTKKVKGEESISRYKTNDRRLTLLNGVQLLGEYASTEDTAHEWLHEQLVKSVRHHQAILRAVSLLQSAMDVSITILLFVNMANLCASLFVAGVLLHKEGNVGRALNALFSIPALLYETTMYCIYGHVMTDHSERLMYSAFSSGWINSDARFKRSMLIFMMVTMQPMDITVGKTYTLSKQMLLQVLNGTYGLLNMLYHMHGSS

>CnigOR36

MGDPSADSESLEGPGVILRRVMGLWRPRGRVARIFNVLFAGITLVSIAFLVVCVALKLYADPPAELEQIALCGLVASLCVGFFFKASLFMALGGTLRQTVRLLEDTRVEFFSGDNNELTRRRYQK

>CnigOR37

LAYSAFIQLCSVVYIVMCMLSIFSPEGDINDVTLTLMHTFEIVCGILKATIFFLKRDEYYQIVHNLDRLVSSQRQYLTASKDEHLLTMLDAAHKKADFLTLVLTGYIYGLVLVWLPFPLILMPSERLMPLVQIPGNYFKEHFAVYITAYAIQSFVPLALIMVVDGLDCFFVSSVVHAEALLKVLSERIASLGHLHYTDFRLQQFSDMEQESRKRNVENNVDMTAELRSCIIHHQEIIEFLERLEKAMNIMVLIQLSFSMFNLCMALYQQTKIPNFTSALKYVIYLPFPTMKIFFYCWAAHNVKEQGEEVSWAAYNCAWPDTNQEFQKSLAIIMCRAQRPLLLTAGRIYPINKDAFVSLIKGSYSYYTLLRQFESK

>CnigOR38

ETPYYQILCTLQFFCMQYGFFFAVCLDVFFVCIIIHAAGQLEILNARFRHVGEITGNYSANPHKQQEAHEEFSGDVSGSELIWEDLCSCIKQHQDTIEIIQEIERLLSKIVLLQFIGATIIICVTLFQSSKNTENIAALLLLQAYLGVVIYEIFMYCWYTDDILYQNSRIAMSAYSCNWPGAPPKFQRALAFIICRTQRPVGLTAGKFYYVSRESFVSLMSASYSYYALLRQVNDK

>CnigOR39

APPARRPLGQPRLPPAQRRRLRHRRRLQLLAGARHWRGAATNLDRFTHFSSHCNTITMWTLRLGIIAAHERNFHHLAGQVERDFVEFIAAEDIPLLRDSGLRLRRVVRAYTWFGFAGCMWWLLYPVLCFGLTVEGVPYMMAVPYDVSQPLVFIANLLFCTVATLHVAVMTMVSDSYSVSLMVQLRLQLEILSKNLVAMARRDEETGQEKCVGKGPPTGTSQLQDRIRQNIRHHQAIIRNTELLEKCMGTTLLAQCLSIGASVCIQLYQIAMHAQRLVDAGKFACYLFIMLAQLFVYCWFGDDFITESLKVSTAAYDSVTSLEGCSSSSKRSLLLVMLRAQRPLRVTAAGLFPLSRES

>CnigOR40

ELLNICTRGGTKSSHSCNCSGILDTFCDFFRNCKRGNDMVMELSSSEQPQDAAAEEPNDLDLMLKILHWTGTMRHPHAGPWASRAHRLIHALSTVALLLFDCSLVALLWQDGAADLERFTLTLCVLNTAGFFTLRLRHIAAKEPEFHRLALQLRRDFGQFLSMGDLHQLRLGSRSMRRVLSAYQWSLVLTAVAYATVPVNADGLPFVLALPYDASRPLAFAATWLFSVYIVFCVHIGTMAADSFNVTVILQLHNQLDLLGRNLRSLNDSISDMKYFPIQTLPVKYKQHSDASHDIHYRLRKSVLHHQAIIRNVQLLEQCLGGMLLGQSLSIGTSFCLQLFQAATRAKRVQELGKTCSYLVTAFSMLFIYCWFGDDLISESEKLAFSAYDAVTSLQ

>CnigOR41

DAVTSLQACPTSIKRSLLLLMVRAQRPLRLTAGGFFSLSRESFVAVLNASYSFFAILRNFKED

>CnigOR42

MSVRVPWRDSVVWANARLLCAAGVWPPPGHGGSYLLYTCWLFASQCFMIAGQLAGLWYFRADMDKVTLDVCLTVTVVMGVIKGGAIVAHKRRFFSIVNRLDDATAAQLGAGDPEVAAVVESTVRLSRAITVMVPVMGSLSPVMWGLTPLLLRLLGGSQKPELPVVCWYGSWDTASPYYEVLYAVQFVTIQGGYLVVMGSDLFFVTLMIHAAAQLRILNMKLVKILNSEVEVNGKHFEGIKCVPDYHNNDLKSIETSVKGCNSPAKHHHNLSDETSSYDELRLWVEQHKDVIELVQHLEQLLNMIILFQFLGGTIIICVTLYQSSAKTGEVTTLFKLQLYLGTMLSEIFMYCWYADGIVQQSARLATSAYSCGWPDAPQPFRRSVLIIMRRTQRPLSLTAGKFYTISRATFVRLVNASYSYYALLRQMNDH

>CnigOR43

AMLHFWGDILSATNNACVTSSYSMAIFKFFAFRLMRRSAEHLVRDLDRCLQVYGSEYAAEKEAIFGACAQKARLVSRLQVSLGVSVYLSWVVLPAIRAHACHSTDCRVHTGFPALVWYSFSFTEPPVYQMIYAVICMGLFYGCIIFTSQDGFFWSLIIYVGAHLRFLNFMVTSMSSGEGDAKTPRESPEMEENMRRRLKECVCYHNDIDRCVQRLSSLLGPVMLGQFLTDIVTISASAFVATMVKADSGWLLKYGSYLSGTIEHMFLFCWFGDDILTESERLALSAYSSDWTDASPRLRKELRVFLCRSQRPLILTASKFCAISRQTFLRLMNASYSYFALLNQLSSE

>CnigOR44

MAKEVPEDLEQPLSWAASGQSILRLNIRHLWLCGAWPLPGSWMYDTYAILGLIVGAVNAVESAVSLYFYWGDMEETTLLLTSSVSNGCGTVKLAVVMRNQKQYHALARRVQALVALQSDLVQEDPALSAIAQSARRRAYRITLGMLLLMFSQYFVWYPMPFYVDPGARRLPFAQHKWDNNSHYYVLSYFLQCAASARGTQLSWSLDLLFMSVMLLVGAQLEILTRRISSLKTESSEGKLEIVVKSEKAMLGMLANSGDKMYENLCLCIQTHQKILSFIKYLDNTMSSVAMIQFCVSVLLVCVALFQATYSTDSTAVLRCALYLPMPGTQVFLYCWAAHNVTEQAEAVSLSAYSSCWVGTGRRIKQALRIIISRAQKPPVLTAGRLYPINRETFLSLVNASYTYYALLGQMNKRSTN

>CnigOR45

RRHHTANSGTALWPGEMADNVLPKPVVDIGLSKAALVAIGLWAPSRPSRLYWAYTRWSLLQLVLTIAGQLAGLQGHWDNLPVVSTSLCFVLTITCTFIKAVIFVAQRPRVDALMAHLERNLSQFCSYLPRAKAATLRTCRRRVLLMFTSFMGIGGITLVSFYTGPILQNFRDAELLAAGAGNATARPHMGRNLPMLVWWYSGQPEQTPSYQLAYALMCYWLMLIYLACDVQDAFYVTLIIYLSGQLRLLNLMAESVCSGIAGADDASEERARARLAQCVRYHTDVDRCVQQLSALLGPILLGQVLADVVTISATAFVTTTGKTDSGWVFKYGSYLAAIAEQLLLYCWFGNDVLTESERLQLSAYSSQWVGAGAAFQRALSVVMARARQPLAVTAGRVYPINTAAFVALMKASYSYYTLLRQLDE

>CnigOR46

MVKAHNADAGGGGKRLRTDLRLQQWLLRFVGAWAPRRGPSTSAWWRLYGAYSACIVAILLLFVASLLFAMVHYWGHMLGVTMNACLMFTYVMNIIKIVAFLRMRPAIDEFIDELDRCMQEFGQELWSQRAAVFRWIALKSRIVSVARLSVTAMGCVYWSVMPAVRARACGDTVQCRARVGLPAHVWYPFSYTQSPVYEVIYAGVAAGLMYGALLSSIMDGFLVSLFIYMAGHLQMLNLMLSNMCGGQAEDEDHHGKRTRHVAAVHQEQVIRWRLAQCVTYHCHIDRSVQRLSMLFGPILLGQFLMDIIAIAATAFVAIAKNADSTWLVKYTSYLSAVIQQLLFYCWFGTDVLTESERLQTSAYSSDWVDASPRFRMELRIFLCRTHRPMRLTASKFYTISRETFLMLMNASLSYFAVLREINAK

>CnigOR47

MSATAAPSDALLSPCSVALSWLGIWRPPGGRRWGLGVFGAVLIAGLDITISSLALVQMFIDRPEDPADFREVFFICSCGLSWSVKVVAFLLQWERLQRMVLCLLDAKTRFPDHGAGVRERYTKMAYTVWRLWQAFPAVTVLLWMSDPLLQTLMEQPAENATRPLIFWLPVEVQGSPAYEITYALEAFFIGTVSETSILMDIFLIILLVYAAGEIAVLNENVASMGLTKQREPAKAQPAESSGEKMSTSKTGYALSQSSAAPDVTASSKAELLALDRDDARSELYSALARNIKHHQVIITYINDLEVVLSTSIYILLLTNALNVCLHSFGLVALFQEGATSSTVVKEIISFVSFLAQTGLFCFFGQLIIDQADQLQFSAFS

>CnigOR48

APVGALLLAANDVSLGLSALAFVLMESARDLKSISDAFFVIATSVSVSSRIFLLTGQKGLMQELVTLLMEARRSFPECAPGVRARYHRLAVVMFRIWQVLPMFPLMLWVLEPVLLVKFSTREANVTAESLRNTPVPIWLPVDIRRSPNYEITYAMQATSIILAIGFNGFFDIIYVVFMVHITAELEVLNQNISSMKAGGKDDAGDSRGRYESRVKHQSDYKSAVVPVHFHERSFPDDLEEEDRVYSILVQNIRHHQLILAYIRKLENLMKTSIFIILLSNALSICLHVLGLAVMLQEGVDLPSVLKMLITFPAYSCETGLYCMFGQSIIDQSERLVDSAFNCDWLNMGPRFRRSLTIFMLQASQPLNVKVGKRVILSRHTFLRLLNGSYSLFNMLHGVQK

>CnigOR49

AGVWTREGVHLLQIKPFNDRGDQMEVSESELLWPLSSVVWELRLLGLWLPPPGRRLRHRLVLAFVMVSHVANLLAALACITMDTPSDLPQLSFIAYNCLTDAGLTCKMLSFSLDGERLTELLRLLAASRQRFPDKAGHRARHQKTAVRLHRFLQVTYRINTAYWLLAPIVRNIMAVISKKTYNRDIPIPIWLPFDIHWSPIFEILYSLELAFGWAISETTVLVDGSLIAMILQVAAELAVLNDHLADGTAPTLKQALSGTITTEGTVKDSDVQMPPKSSLVVEAYSDFQHSSIRSDGEMYQLLVGYVQHHQTIIRCVRLLQKILSRATSVLLFCNTISICFQVIATAVLLQEDGEIVQTLKMLMGSTLYAYQVALFCLLGQRIINQSERLIRSAFSGDWPDGDLRCWRLVNMLCINTKRTLSLKICGIYTLSRMTLLQILNVSYSLLNFIYQTKTEQNIAKE

>CnigOR50

PLKLVVDTPKELEEIANCSFVALVLCVLWIRVFHFITHGSTLRQVAQLLIDTRTQHNQGEANECTRRRYQRHVERVFKLFQVAVVAALIEWILWPVMSRALLGGDQTSRQLPMPIWLPAALYSSPTYEILYAAQFAGITAAAICSFCLDAFFIHVMLLIGSEFEVLSDNVSAMQNNYEQAATFKKRGKTSEFQTIKETLASSCADIYTECESDEQMYRQLTKNIQHHQTLLRSISLLQTTMSVPIYILLSINMVNLCANLFITTVLLQRDDNVSKVLKPLAAIPFLLCETGVYCIFGHGLTDQSEKLLQAAFRSDWTDCDVRFRRSLLIFMTATTQPVEIRVGKVTKLSKQTLLQVLNGTYGLLNMFYHFQSKI

>CnigOR51

MPAATPWSESALSLNAFVLALGAMWRPPAWRGPALSTCWALYTCWMLFTQLSFLVAQARALWYFWGDMGKVTHDVCLMITVVLGLIKFCVFSLRKDDFFRIVQKIDSARSEQRKSDDPEIVSILHASYKSARNMTLYMTLLGGSSPAVWAVTPGIMRKLHVGPPERELPATAWYSSHDTESPLYELLCVLQFFSMQYSFFAAICLDLFFVSIIIHIAAQLQVLGVKLRSIGKLSDRRSATSTNSHILCQEVEVGFYDERGLWMELCSCIQQHHSIIELVKEVESLLNIIILLQFLGATVVICVTLFQSSTNTSNVMTLVKLQAYLMVIIYEIFIYCWYADDILYQSSQLAVSAYGCGWLEAPARLRRALPLVLARAQRPLGLTAGKFYHISRATFVRLISASYSYYALLNQMNGK

>CnigOR52

ALFQEGATRSTVIKEMISFPSFLGQTALYCFFGQLLIDQAERLQFSAFSCDWPHADEAFRRSLRIFMLQVAQPLNVRVGKLVILSRNTFLQALNASYTIFNMLFNVEKRN

>CnigOR53

MSSLRLNINILRVAGLWPHPGISSSSSRHSLAYTAVAAAFQVGLLGTQALALVWGGAGLALVSLNVCVMTTIAAGVCKAAAFLSQLAAARQLTACLQGCQHSAAADAAARGVTSYYVGVGGAAVVVWNLMPLLLEADRSLPTIAWYPYDETKTPFFELTYLLQGLSTFYCCITNVGLNVFLVSLMIYVSNELNNLNDSISSINYSSSYNCNCSDDKQKGLLHWSSKMVPDDGDINVDTYKVHSKNLRKRAQFCCVVKAQEYLGMCLQHHQELIRTVKKLETTMSGIVFIEFVAGIIVTCLTLFHAAVNAGNMALFLKFIMYLLYMTVGMFIYCWYGQDLMQKSEDMKWAAYSCNWQGAPRSFTDLLKIVMLWAQQPLTLSAGKYYRISLKTFVTLLNASYSYYAVLRQMNDTQK

>CnigOR54

EAARNVLAFSVLLNKRSGRNVPFFLQLTLYLHLISIIIWRAFLQSGSMARSAAPRSVTSAAEAASDLNHLLRPLHWTATLRHPRSAASSPLIFRLCKIAITCIIFSFFCSEATVLFRVGTSDLDIFTITIGVADTNIIWFSRMLHISVCERDFHKLALQVGQDFAEFLTWEDIPVVRSQSLAVRRFTQMYVWFGVGCVVYYLFSPASDTGLPVILALPYDMNQPLTYAVTWLYVAITTFHVVVMTMVFDSFNVSLMAQLRIQLTLLSNKVVRLAKEMSEKPVHSIETSAYHELHQRLETCVRHHQAIIKNSDLLEKTIGPMLLAQCLAIGACACFQMFQVATNTNGLQETGKYGAHLVVMLAELFEYCWFGEGLITESENVALAAYDAVTSLQDCPISVKRSLLLMLQRAQRPLCITAGGFFPLSRESFVSV

>CnigOR55

PTAARGRFKRALTILISRAQKPLVLTAGRIYPINREAFVSQINEDVGWKCYPESLTPVMILRGPHKTSKTTKTGNFCLAKY

>CnigOR56

SHLVAQRLQVVIMATAGESPVALLGRNASMLRLLGLYQQKDGGSKVKPALLSAFSLIFLLYHPVFAAMKLYMEPPEDLVEFALCSFSFIISDGVFIKTAIFIADRGMLHQMLQVLSDSRRLYGGEETSMKIRNRYEDLAERVLLYMQVSTMVASVGWLAAPLVFRALLLASGEPQVPRKLPLPVWLPVDVQASPTYEILYVIEAYCVTLTGLVTLCIDVLFIRLMLMVTAELQVLNNNVATMAKRREKIVNELHPHIATQNFYRGDKALQMANTSCEEGLDGQLYQQLVTNVRHHLIILRTV

>CnigOR57

MEKMRQEDAASASASAPLRTDAVVPRTLLRAVGAWAPQGQPWSSLYGAYSAAVGASLLAFVASQLSAMLHFWGDLLSVTTNACVTFTYSLATFKLVAFLRMRRSVDQLIADLDRCMEQYGRHLCAEKRAVFAACARRGRRVSGARLALAVVFYVSWVALPALKTRACATAACRNKGGFPALIWLPFAYAEQPAYEMVYTVLSLGLFYGSHVSTGLDGFFCTLIIYLAGHLRFLNLMLLNTCADVQVEAARGHKQKMAEASRDPDSRMRSRLRECVRYHNDIDRSVQLLSGLLGPILLGQFIVNIITISASAFIAITLKADSGWIFKYGSYLFAIAVQLLLYCWFGNDILTESARLQESAYGSDWTGASPRFRGELRLLLCRAHRPLRLTASHFYTISRET

>CnigOR58

RVSAVHLRIHSGHFILNGHGNKQVSDSATGTQAERMAKHKDLPDPVVDLRWMSRALAPVGMWGPSGGSRLYDAYSIWTIVQLVLTAAGQMAGLQGHWDDLTTVFTSLCFDLTVTCTIIKGTIFVLQRESLDALSRHIERNAREFCSHLPDERRALLARARNLSRLIVCSFQSVGGVTLVSFITGPIVQNGRDRELLDSGLSNATVNRQLGHNYPMLMWWFGGLPVSSPGYEAAYLTMCYWLVLMYICTNVPDAYYVGLINYISAQLRLLHIALRNIAHPEPGDLLTEKLSHSYGLTVKGGVPANGGAQDSSRDSVFDRLLECIRFHQEIIKCVDEMESLLSLTVLIQFFTSTLVICLTAITVINTEAAYLPTYAAYLATMFYQLFIYCWYGGEVYLESESLQFSAYSCNWPDTDARFRKALRICMARMQRPVNLTAYKFYKLSRETFLLLLNGSYSYFTLLLQMNQKND

>CnigOR59

MSNRNEGIYFGAFINLMHFFKIWTPDDSKRTISFSVYILVPAYLFFFALSCIEIYHNWGDMLSTTDAVNTFVIYLATGHKYFCLIYHEKDYKMLMKIVENNFSAHIRQNDALHNSIIKNHVQEVKKLNILFTTLCFTNQCGFMIVPLADSVYHYYTANVTTEVEWTIPYRTWTPFNDYGPVVTVPLYIYHMFMVFVVIVVLPAFDTTYLSLISHSCAQLKILQNSLINIVIISTQNEHSDEKNSVTFGTILDEHCTIEENKDRSSVPQAHKMNSAFNNYTSNGNYDSRLNPLHDSELDNKIRKNIGQLVNHHEKILEFIDGVQAIVNAAFLTHYLCCGTLFCITGFELSVILKEQQLGGRFLNMMELLGGAIFEMGMFCYYSNRVMDEVNNVGMAAYDSQWYYVSKDYGMSVSIIMARCTRPPRITFGKFADLTMEKFGSILNISYSYFTLLSGLNE

>CnigOR60

MKNQVDNDDETWDTTKEVEAMLGPSAVLMRAIGLWQPPGGEPAGAKVLATAVVLLMMAVVFVGGVVEIVLDPPPLEDILEATFTCACCLTWGLRITIILLRQSRVQRLVVDVLRMRRRFTENTAVLRKKYHRRGLIVCVAWVVFPLLAVPMWFVEPALTKKEVTTSENTTIVIRKTPFIMWMPMDTQGHPNYEITYATHIALLNIIVSVTVLVDLFFATLMINITADIDILNNNIANMRLHKEDALTKNSAARDSTYESKKILTEKGEPYGENTAVHTSYTTDPSTQLYRTLVINIQHHQVLMTIVSDLESIMSESSVIMLAVNSINICLQALGFVDAFRPGSKRSAVLKKILTFPAYINQTAHFCWYGQEIIDQSERLVESAFGCGWADADKRFCTSLRIFMLQASRPLKLQIGKIFTLSRNLFLQILNTSYTIFNMMINF

>CnigOR61

AALSRSHHTSLNRAEVITGFNNSRVGPSRRLKTGGVDASNTKTNPPKDYREKGGMQGGQREDAPLSWSDSGRSALSLNIRHLWLFGLWPLHDFWPFYLYTACGLVLGFWNAVEGVTAACFSWGDMDDMSLALLTAINQGCGALKTVFFVCYKRHYSFLVRRLDALLSMQSEVFGGDSPQLASLRASQRRARRVTLATLLFMLSQCFVWYPMPLVAHSEERRLPFAQHPWDGNRHQYALSYVAQCAAGLLMTQISFGVDCLFVSAMVLVAAQLETLAMRIEGMAVEDISPYNAKVKYSDKELGRRGTDEMYNLLCVCIEDHQKILRFVTHLQETMSPIAIMQFGASVLVICFTLFQATYSEDFSSAFKCTSFLPVACTQVYLYCWAANEVTVQAEAVSVAAYSCSWVETSRRFKHALRMLISRAQKPLILTAGRLYRIDKEAFLSIVNASYSYYALLSQTTNR

>CnigOR62

MDWASQGSQPLTWKYTASSVLKYDVRFLNVCGMWPLQGSRLFRIFTATILALGLGHIAEGGVNLCTLRGGLEEYTLALSNVSVFIVGVLKVSFFLRNEGSWCRLVRWFDSLVESQRQFLLDQPSCGAIFRKAQQRTNRISKGLDAYNASLIFLWMLAPLIASPGAKRLPLQQLPMANTTDFPLYELSYFVQGTSLMFIGLLNVHIDCFFIVVMIHTAAQQKIVASRINDLHLQQNGGGKSLTESKSERNGMVTQDLYKELCLYIQTHQDITSFIVHLEKVMNPIVMMQLAVGVFNGCMLIFPAAYSAESGSLLKVLVCAPAVSMQLLLYCMGAHSVREQGESVSVAAYSCGWPGAPRCCRLALLLVMRRAQRPLTLSAGGIYPIQRATFLSLLNAGYSYYAVLQNFTGR

>CnigOR63

MVTLGNIPWTDTALWMNARVLALGGMWRPPWCQQKWYLLYRTWVLFTQFSFLFAQVQALWYFWGNIDLITHDTCLLITTILGLVKFFTFVLRQDDFFRMVQKIDDTRAEQNKTGDTEIISILEASYKSARTITLYMTFLGGSSPGIWAIIPTIMRKFGIFPPERELPATAYYSSRDTETPYYQMLCTLQFFSMQYAFFAEVGPDLFFASIIIHTAGQLEALNARLSSIGQITGNIGASGEKHVKAPGKYFDEIPCEELVWKDLCSCIRHHQEVIELIKEIERLMSKVVLVHFTGTTIVICVTLYQSSQNTENIAALFMLQAYLGIVIYEIFIYCWYAGDILYQNSQLATSAYSCGWPGAPLRLQRALAFIICRSQRPLGLTAGKFYYVSRETFVRLMSASYSYYALLNQVNDK

>CnigOR64

MEKQKERSFQVQDLGHGDGEDLSWDEVECSVLRQNICLLYYMGLWPLESSWAYHCYTVFNLTSSAAIILMNVVGVCYSLSDIDQVTGALSTILPMSGGLINGLFLLKQRPTICRMVRAIDRLVASQRQYSQRDAQLDAVVGRARRQTLVVTVGVSGYLFAIASYWIVIAFTLPPSLRVLPFVQLPWMPSSGPGLFWSTFAAQLYTAPFCSYTTLFVEFFFLAVMLHLSAQFKVLGSRFASVGRSSPSKAAVHSDAVYEELRLCVKTHQELLRFVRFLDDVMSPFAMMQFVAGTLAVCVVLFQAANNQDLNTNLKCAGWLPGPSLELFIYCGGAHEVVHGGEALVEAAYDCLWYDVAPRVGRAARLVITRAQVPPVLTAGHLYPITRPTFVSLVNAAYSYYALLSQMQNK

>CnigOR65

NTFTLIGGFIKLIYFSSDVRGYRELVAELRDATREQWPHCEAHAELMAIFTAGYRKASWLTFGPLVYLNILGPTWFFMPLIVRATTGSKERLLPFVNMRDSVTEIFPLYVAIYVVQVYCMFFWNIISVGLDMFFVVCMIYVATHLRILNARLSSLGEDSTDEASRAGLNRNIIRGFGSSQKRPFSRDGRTRDVYEELRSCIKTHQHIRSSLKTLQRMMSPVAMTQFLCSASGVCITLFQATFNPEGNSTLKCLIFLPMPAFQIFIYCWAGHEIVYQEELLSLSGYRSAWVGTGRRVSALLHILMCNAQKPLQLTAGKFYPVNRDTFVTLINASYTFYTLMRQTRDEGSMVQT

>CnigOR66

RTHCSVGLCHRRSTLGGHSSSKMEAEVKSLVGPSGWVLQQVGVWRPLDSGPAGLRLLAALLVVATDALVSTSSLVQLIVDTPTDPETLRDVFFQSTCSGAWAIRLVMFMRQRRRLQRLVMTLLDTRKRYAEEVPGIRKSYDRSAAIVFFAWQMLPLTAISLWALEPATAPAETVVVGNSSVVLRREPLVLWLPIDTQQSPAYEGVFALQVVGIATVSEVSVLLDIFFVTLMIHVTGEIAVLNTNVTRIRLSRLSGKASLPYGSGDATAVVQQSYKGGGQPADGDASTGWSAAGTGSLPDKALCYSQDTDDAQRRLYASLKTNIQHHQAVILCVNELEEGMSDSTYLTLIVNALTICLHAFGFVELFQGGGKGPAVVKRLLACPIYMGQTALFCLYGQSLIDHSERLLDSAFSCGWPAADRRFCSALIIFMQQASQPLTIRVGKIVTLSRNSFLQIMNVSYTIFNMLLNTQ

>CnigOR67

MDWNPWEEAPLTWRQTASSVIRFDVRILYVIGVWAMPVTKLYRTYTALILVLAVAYSVEAVMHIWSVRKNMEEVTLAVSTYAVIVTSSFKLLSFLQHESGFWRLVRWLDALVADQKRFCEERPPLQAIFDEAKKRATRYPNALRIYNTSLIIAWVFTPLLAPHGHRPLPFQQLPLSDTDDFPLYLASYLLQSLCMFSLCLVSGCLDSFFTAIMIHTAAQFKILGLRIAALRQENGERKQDTRSDVHEKLRKDAASDHVYEELRLCIRTHQEITSFVAHLESVMNPIAALQLITGVTNGCLMIFPTAASSESGALLKCIGCMPTICSQVLIYCLGAHAVMEQSEAVSAAAYGCAWPDCSPRCRRALLVVMARAMKPLTLTAGGIYTIERATFLSLLNAGYSYYALLKNFNSRE

>CnigOR68

VLQEVMSPVAMAQFVCSATAACITLFQATFNPEGNSIFKCLMYLPMPAFQIYIYCWGGHEIIDEGSSLSVSAYSCAWIGATRRFTTALHILMCRAQKPLILTAGKLYPVNRDTFVSLINGSYSFYALLRQMRGH

>CnigOR69

GTPGIHPRAAALHSSTSGSCPGWLHPRRRGLPSHSARAAVAAAEAASDLGYVLRPLHWMAIMRHPRSAAGSPLLFRLCTLGFGAVCLSFFCSEVVVMCREGASDLDVFTMTLSVSDTTGIWVVRLVHMALRERDFRRLALQVEQDFAQFLQPDDVPALVSQSRAVRRFTTTYISVGVVFSACWLVSPVSPDGLPFILALPFDGTKPLGYAVSWLFCTITCLHAVVMTMALDSFNVSLIAQLRMQ

>CnigOR70

MTDQMHSGGEVWDTTKEAEAMLGPSAGLMRRMGLWQPPGGEPAGAKVLVAVAMDVMFFLIFAGGLVQILLDPPPMDSMLEVSLTLACSITWAVRNMTILVRQNQLQQLVLDLLNMRRRFTENGEFFRKKYYRRSLIGSTLLLGIPMFAIPMWLVEPALTKTLVTTSENATVVVRRTPLVMWMPMDTQTHPNYEIAYTFQLLLISIVVNANIVIDIFVCCLIITVTADIAVLNHNIANMRMYKERFTENSEEGVRISRTWEITYKSKKPQMEESEQYDDRTDVHASYTTDSSARLYRTLVKNIQHHQLLMSIVNDLESIMSESSVLMLALNSINICSQGIGFVDGFRPGTNKTTVLKRFLTFPAYVNQTAHFCWYGQDIIDQSERLLESAFNCGWPDADQRFCRTLRIFMLQATRPLKLQIGKIFTLSRNLFLQILNTSYTIFNMFINF

>CnigORco

MQKPHGLVADLWPLIRMVQYSGHWMLEYSGGKALRAIYSSVVSVLVVTQFALMAVNLIQRSGDVNELAANTITVLFFLHPVTKFGYFAVRSKAFYRTLATWNQSNSHPLFAESQARFHQLSVVRMRRLVMYVVAVTALSVVSWTSITFMGDSTREVTDPDNANETIIEEVPRLMISTWYPFDASSGMGYMLGFVYQLYWLTATLMHSNLMDVMFCCWLIYACEQLVHLKEIMKPLMELSATLDTVVPHTSELFRAASTLPTSEPLYGMGPDMSNGVTDGMTIRGIYSSQRDFSGFNRRSAALSTVREADAGGAVTSAGGIGPNGLSKRQEMLVRSAIKYWVERHKHVVRFVGNIGDAYGAALLLHMLTTTVTLTLLAYQATKIDSVNVYAASVLGYLFYTLGQVFLFCVFGNRLIEESSSVMEAAYSCHWYDGSEEAKTFVQIVCQQCQKSLMISGAKFFTVSLDLFASVLGAVVTYFMVLVQLK

>LmigOR1

MSNGNEGIYFGTIINLMHCFKIWSPDDSKRTISFSVYILVPAYVFFFALSCIEIYHNWGDMLSTTDAVNTFVIYLATSHKYFRLIYHEKDLKKLMKMVENNFSVPVWQNDALRNSIVKSYVQEVKKLTILWTTLCFTTLCGFMILPLVDGLFHYYTTNVTTEIEWKLPYRTWTPFNDYGAIVTVPLYVYHMFMGFVLIAEIPAFDTIYFSLINHSCAQLKIIQNSLINIVSISAQNVLSNEKNGVTFDTMLDEHYTAEENKDQSSLENKMNNGLNSYTPSGSYDSMLNLLHDSELDKKIRKNVGELVNHHEKILEFIDGVEAIVNAVFLTQFLCSATLFCLTGFQLTVILKEQQLARFLNMMELLGAAIFEMGMFCYYANRVMDEGINVGKAAYDSQWYYASKDYGNSVSIIMARCTRPPKITFGKFADLTMENFASVLQISYSYFTLLTRINE

>LmigOR2

MLRQVYEALRDADEPSRYLEFNVLMVRFMGVLVSRSLTGALLTWSLFVLLATHCLAGVFDLVNNSGDIADITANLPVTTIIFSSTYRLFFFTLHRDRYQAIVDTVGARFVASSDSIDMAPWLRRSRIISILYFTYGFFVASTWQLHPLISAQLTAAQMAKESNGTFEGLPRELWEFPTRAEYPFDARQPYVYTVVFILQGAAIFVSGCMILVLDMMFITLTSLICGQFEILKDKLRNMRKIATNQRQNEDIGLVGKALQKQALERRKRILDSNIEITKQDENFNEDLITRKINLLLGECVEHHNMLLSLISEIEFMHWSAYLVNFCVLLIIFSFSAFEVTSGTPTSPAKVVNLAEYLLVSVLQMFLLCDCGDKLVDQELSVSQAAYESEWYHCSESVKRTLQIIVLRSRQPEQITVGKIAGLNLDTFSDMLSRSFSYFTVLRQIRDDS

>LmigOR3

MEEAVEAARGRAPRLLQTLRGDVAAPAHYLAFNVWLCRLVGLLPRPGQPLWATVQPLVWSCLLLHLMCELVDIALNIADVQQLGKNLPISSLVGGSWYRLSYFTMRRDAYWRLVSKVGESFHRGAPGRMRRWLRRSRGFTLAYFVYGTIVCLFWLGHPLLLQQTTHTMFTSSNSTNRSRMSETAEFPSGAWYPFDVRERRVYGAVYGFQCLALYFAAMLIMVTDIMFITLMLLACGQFEELGDKLRHCWEIATTRALSRAGTTPERELQKVLAHCVRYHDMLLGIVGDIEDLHWTSMLVNFVLQLIILSFLAFEATASADLTNPLKGTNLLMYLVMAIFQLFLLCSCGDRLMEAEEMVARAAYESQWFDAPQGAKRSLSIIVMRARLPQRVTVGKVVGLNLVTFSETLSRAFSYFTVLRQIRTSN

>LmigOR4

CEMAVLGADWETRMLTHGAPCLDLTVYLQPILKPLAATGMWSSSIYSNSVHKRAARAAGLSLLALFLAELCAELVGTPLVVARGGGVDRLIHHLSVLGIHLDSFWKWLFMLTQRRRLTCLLRLLQRCFQLGVLASPTHHLPLRNTYIQKPQQSLDPQPQAMPAQLSEVLGKTRMRSWLVTVVWTVSCVLGASHWFFVPLLKGDTTLRFDALYPFDSQQPTTKQTVYWLQYVCSIYSLLLLCFFDCLLVWLQQLLCVQLRCLATNLRELSPNDEHRRKLAICVSHHEHILRAMRELNAFVAPLLFLQCFKNMIVLCVVAFLASVAGVNDLLELSSLVLYFMAACQQLFFYCWCCEELTHLGMEVCDAAYDSGWENWDVSSQKSIIIIMWRAQKPFFFRGGWFYTLNVATFVDLIRLSFSYYTVLRSMREG

>LmigOR5

MLGQGQAGPGGRGQHLPRVDLLAHLSPILRCLAVFGMWPPSVYTSVSQKVAALTTGAATLSLFLMQLVAEVMALAASPVSGAAELYRFIYNFSVVDLHLQGMGKWAVMVARRRRYTALVHRLQHCVRLSGLADADYKNQQAAVSRLRQQLDDCRRWGVRANAVWLSVCVYGVTHWCLVPLLIGDNSLPFDALYLFSTDQPPLRQVAHCIQYVAGLQNVLLSVFFDLFVFWLHLLLCAQLRYLAGNLRRLRHLTDAAQYRRMLAACVAHHSHLLSTMQELNSCAGPSFFLQCFENTIRMCMIAFMATTTVADQMQVWSSAQFFLAAVAQLFLYCWCGQQLSHLAESISEAVYDSGWEDHDVSTQKTVAFIMWRAQKILVFKGGWFYTLTTETFVELIRLSFSYYTVLRNINDT

>LmigOR6

MDVSFAAVSVMRAGLPAEESGGNRRARRVTQWSLALWLGTTCSWMLAALLRLDLPFFAWFPFDTTHHYAEAFIYQLVTANLVVVIISGLDCFCLELMMHLSERLQTLNKLFRSFATDNARQQPQLRMQPSTPASHRGKSPYGIITKKSFMKWVVAPLDVHRKPAKAINSLYSRHDSPNVGNANGSFKHCIQYHWELIELKKETEKFCGVVLFFQILASMFIICFVTFQATVNTMDAGSLTKCVMYLSVALLQLGLFCNEGTNIVTQSEELMLAVYSSEWPDCDAALKQSVIVTMMRLQYPLQIRAASYCTLSFETFSKILHTSYTFFTLLRQVSETQ

>LmigOR7

MRELEEEWERQRLRGLRLVVRALQLMGAWPPGADAGAGGGGGGGGVRRRLYRAYVAAATALMASYPACALCVLAGTRADIQVTAVLVGVTSSYVGAIFKMFTLCYKRSQVSGLVRAVQREFPRSPLLAGRAQRAVAASAGQHGARLTALFVSCFSSVFNWSLGPIALHLLGGGGGALALCLPWFPDHQPAGAAFRLVYGYQVATLVLVALWVGALDAFLLVLLVYAAGQLRVLNCTLLQMGARSDDGTEGQREKFASNASAMLRECAKHHLEVCRFVQDVEQVAAPALTLQLLVSTFLLCMSAFTATQIPVGSPLLARVVVYMLTGASELLIFCKYCDDVISESERVQQALYGSGWSAQGGAFSRGVLIMLARAQRPLCLRAAHVHPVSLQTFTKVLNASYTVFTLMRQIKD

>LmigOR8

MDYQLGDHGCGYPLRCITDRSACDGVRSDEPGCTNGKTSFFSDESRFCLRHHDGGIRVWRHSGKRTLQACIRHRHTCVSPGVMVWVAIGYTSRSPLIRIDGTKRQEELCLATDSSYIVDNGIETLAVLWGFEKFRYFLYGCKTREYTAHSLDLSPTENVLSMVVEQFARHNPPVTTLDELWYRVEAAWASVPVHAIKVLFDSMPRRIKAVITARGAVLTRLCPVVPQYPIIALYPWPVQSGPAYALTFSLQVLCGGLFTMTHLACDTFLLSLLIYICSQIDVLCASLRQLGRRLLRIVSLVSYRYVGVLQQVLSPVALAQFMCSMVIICLSGFGIAISNDFGSLCRYSVYFTGAAIQLLLFCWYGEVLITKSEHVSEAAMACGWPAVRRGRFQSSALLLMVRAQRPLALTGSKFYVVSLKTFVQVTNAIITYFKIAPTLGK

>LmigOR9

KLSLSTLRPRRYRRLMDHVTRRLAAHLRHDRAGRLRLRRDSRAAHAFTMGFVVCGHVTVASWSLLPLLLKPADKLRLPLVAWTPFDSSHGTGFLVTYIYQFTCTLFMAWTSGATDLICVNVVMQLCSHLDILCSHLERVGRRCCREGDGGRCAHGHLDDGDQRPAPGRDDLCGQLRGCIRYHQDIIRCRVAREMDSMLWTIVLSQCLCGMGVLCLLLFQTAMYTLTIETIAKYLSYMASILLQIFCYCWFGDNLSSKSTAVARTAYNCDWTRGSSAFGRSLCILMARAHRPLTVTGGSFYVLSREAFIRILNASYSYFAVLYTMSDE

>LmigOR10

MELAHLLLRLLHWSGALRHPSKPCGCYLLYSAAVVTIIGLFVVTQVAAVVDRQAGSDLDQATLALCVASTMCVGMCKILNILRSEHLFLKLADEVSRQPDGLSAWEAGAWLWSRGRVRRLSAVYLSLAASMAVTWPAAPLAVGGGALPFVARFPFDVAAPAGYAAAFAFQVLVVAVVGVVVPCTDLFTVSCVEHLNSLLHILVHRVERLNALETGARPQHATADDKAAAVADPLHRDLADCVALHQRIISEAECLNQAIGGVLLLQVVASSAGICFLLFQVAKKTTFHLVETGKLLGYLTFMLSQLLFYCWFGDDMLSKSESVSLASYRCRWPDAPTRFQRSLLMVSMRAHRPLTLRAGKFFVFSRQAFVQVMNVSYSYFTVLRSLSEA

>LmigOR11

MGNKVRVYLNEGNLPREHGSSLGEGGRGLKLKGGMGGEGDLLEDMGTLEGIDVLENSGHKADYMLDGGRGSEGVAGVQRRVAEMDGDGRSSGSVHLRWCCRQVERDFAEFLSPEDVPLLRASGRRLRRVVRAYLWFGAAGCMWWLLYPVACFGLTVQGVPYQMLLPYDVARPAVFAANWLFCTLPTLHVAVMTMASDSYSVSLMVQLRLQLQVLGKNLVALARGAEANHQKKCVKHGPSAEGLVRRSQLEDAIRQNIRHHQTIIRNTELLEKSMGAILLAQCLSIGATVCIQLYQIAVHAQGLVDAGKFGCYLFIMLAQLFVYCWFGDDFITESLKVSTAAYDAVTSLEGSSCSTKRSLVLMMLRAQRPLRITAAGFFPLSRESFVAVVNMSYSFFAILRNFKDEMNS

>LmigOR12

SSGLDLLQRLLHWSGTLRHPRAGRWSSLAFYPLNAAASAAIALFLCSQGAAIWREGARDLDRLTLVLSTFNTIATWLFRLGHIAVHEHQFHYLSFQMERDFKDFLNPRDVPLLQASNQAHRRFVLSYLWFGVVLCMVWTLFPVATFGAGPDGLPFIMALPYDVSPLHAFIPTWIFGAFITVHVTMMTIISDTFNVSLMAQLRFQLVVLNEKIINLTTDIETPKSPLTKEEYITTKSAYESVKHTNIHHRLRQNLLHHQVIIRNTEMLENCIGGILLAQCLSIGAAVSFQLFQVAVSTQSLVQAGKFSCYLTVVLVELFMYCWFGDDLITESENVALAAYTAVTSLQGFPAADRRSLLIAMTRAHRPLRITAGGLFPFCRESFVSIVNMSYSYFAILRNFKDD

>LmigOR13

MWAPAAGLDYLLVSLHWAGVMRHPLAQPGSWSGRSFQLRKAAVAAALRGLPXXXXXXXXXXXXXXXXXXXXXXXXVSACLFRLGHISVHEHQLHYLAAQVERDFGAFLRPRDALLVQKRDRQLRRTLLGYIWFGVAGCAWWIAFPLLRFGFCAAGLPFILELPYEVTSAAAFVPTWLFCCLVTLHTAVLTIVVDSFNVSLMAQLHLQLRLLSRNLLALSDGDQDHDSATFKRVASGNEVSCGYANDIHIAHQLRKIILHHQAIIRNTELLEKCLGAMLLAQSLSIAAAMCFQLFQIALSAGSLAEAGKFGCYLSVMLAQLFVYCWFGDDLITESEKVSLAAYSAVTSLQGLPAPQKRSLLLMMVRGQRPLHITAGGFFPLCRESFVSIVNMSYSFFAILRNFKDE

>LmigOR14

EGLPLQLVLPFDTSRPAGWAAGWLFCAFLTVHCVVMNMMADAFNVSLMAQLRTQLILLTRHLTDLAGDVQSEGPSLPQTPRKVVEQPVKFYTPVYRHYSLEGTSSHYQKTNPSVVDAEVQRSQHSYEPSNSVDYRLQKIILHHQTIIRNVDRLQQCLGGVLLAQSLSLGSAICMLLFQVALSAQGAQETGKICGYLCAMFTELSVYCWFGDQLMSESEKVAFAAYDAVTSLQECPISMKRSLLLMMHRAQRPLCITAAGFYPLSREAFVSILNVSYSFFAILRNFKEE

>LmigOR15

RLLAVAAVSYANGCDVVAVCREGITDFDRFTITLSVFDTGNTWVYRRCHVAWHERDFQKLAQQVRDDFGEFMAPSDVPVLRGLAASLRRFVTAYVLMGAFDTVVWLTHPTRGEGLPLLVLLPFQTQRGLGWLSGWLFCAYITADCLTVNFMVDSLNVCLMEQLRMQLLILRKHIAELGNRSESTKYSLSEKESESAKEVGKPQQGKLAFVNETHKGSQVSSEAANSSDIHSRLRGIILHHQAILRNAEALQKCLGNMLLVQSLSLGSVICILLVQIALSAQGARETGKICGYLFAIFGELWLYCWFGDKLTSEGENLTLAVYDAVTSLQESPTSIKRSLLLLMLRSQKPLCITAAGFFPLSRESFVSILNISYSCFTVLRNFKEE

>LmigOR16

MAASAAAAAAPEESVGSAAQSACDLGYLLTFLHWTGTMRHPRAGRRASRAYAAANAAVTLAFVYFVCSQVVVLFRAGTADLDNFTLTLSLIDTQGTWLLRIRHIAAMEPHFHRLAYQVGRDFGQFASAEDVRVLREGSRRMRTVMLLYLAFGLAECCVWLTAPASETGLPFVLALPYDVTRPAAYVATAVYCCFITLHTIMANFAADAFNASLIVQLRMQLALLNRNIVNVNRIVEQERSSPYKSESADALRPYKSYSTSDVNERLRKNILHHQAIIRNVQLLQSCLGSVLLGQSLSIGISVCFQLYQVAKSAESLQDAGKYSSYLFTMFAELFVYCWFADDLISESENVAQAAYEAVPSLLECPTPVKRSLLILMQRAQRPLTITAAGLFPLSRESFVSIVNVSYSFFAILRNFKED

>LmigOR17

MGHLLTPLHWTGVLRHPRYSHQSPLLFRLYTAAVSSFALCFICSEAAALVHDDTGDMDVIILLISTINTASIWIIRMVHIAVFERDFHKLAVQVGHDFAEFLTWEDIPVMRAKSRRVRRFTLGVVWFGVSACSYFLVSPVSPEGLPFILALPFDATTPLGFAVSWLFCTITCMHAVVMTMALDSFNVSLIAQLRIQLTLLSNKIVSLAREMSQRPIYSPETSSYHELHSRLEKCVRHHQTIIRNVDLLERRLGSILLAQSISIGAVACFQMFQIATSANGVQQVGKFGCYLTTMLTELFVYCWFGDDLITESEKLALAAYEALASLQGCPMPITRSLLLLMQRAQRPLCITAGGFFPLSRESYVAVLNVSYSFFAILRNFKEEEQQPD

>LmigOR18

CSATSVSSCRRATCRVVNADGLPFVLALPYDATRPLAFAATWLFSVYIVFCVHIGTMAADSFNVTVILQLHNQLDLLGRNLRSLKDSVSHVKSASTETLPKRMRHSDNSRDIHSRLRKSVLHHQAIIRNVQLLEECLGGMLLGQSLSIGTSFCLQLFQAATRAKRVQELGKTCSYLVTAFSMLFIYCWFGDDLISESEKLAFSAYDAVTSLQSSPASIKRSLLLLMVRAQRPLQLTAGGFFSLSRESFVAVLNASYSFFAILRNFKEEYD

>LmigOR19

QAYIWFGVGACTFFLFSPASAEGLPYILALPFDASQLVGFAVAWLFCMVVTFHVVVMTMVLDSFNVSIIAQLRMQLALLNRKIVSLAKGVNEKLQQCSDTSEYSDLHSRLEKCVLHHQAIIKNADLLEKCLGTMLLGQSLSIGAAACFQMFQIATSANGLQQTGKFCCYLFAMLAELYVNCWFGDDLITESENLALAAYDAVTSLHGCPISIKRSLLLLMQRAQRPLCITAGGFFPLSRESFVAVLNVSYSFFAILRNFKNEDQ

>LmigOR20

MPPSLPPEAAAASSDLGYLLTLLHWTAVLRHPRFIGTSPFWFRVYTLTLLTIDASFVFSVFIVLFREGTEDLDVFTLTLSVADTNGTWLFRLAHTVACEAAFHKLSQQVGHDFAEFLTWEDIPVMRAKSRRVRRFTLGVVWFGVSACSYFLVSPVSPEGLPFILALPFDATTPLGFAVSWLFCTITCMHAVVMTMALDSFNVSLISQLRVQLMLLNSKLVTLAKEESENSKLSSKTTDYRELHYRLVECIRHHQAIIKNADLLESSLGAMLLGQSISIGASACFQMFQCVTSGNGLQQTGKYGCYLALMLAELFVYCYFGDDLITESENLALAAYDAATRLQGCPLSIQRLLLLLMQRAQRPLRITAGGFFSLSRESFVSVVNVSYSFSAILRNFKE

>LmigOR21

AAAAEASDMGHLLTPLHWTGVLRHPRYSHQSPLLFRLYTAAVSSFALCFICSEAAALVHDDTGDMDVIILLISTINTASIWIIRMVHIAVFERDFHKLAVQVGHDFAEFLTWDDIPLLTSQSRVVRRFSKLYMWGGVGACAYFLVSPVCPEGLPYILALPFDAMQPLGFAVTWVFCSVVTLHAVVMTMVLDSFNVSVIAQLRIQLKLLSTKIVNLSKEILNTDVDSSEANVYQELYYRLEKCIRHHEAIIKNADLLERSLGTMLLAQSISIGASTCFQMFQLATRANGLQQAGKFGCYLFAMLAELFVYCWFGDDLITESENVALAAYEAVTSLQGCPLSMKKSLLLVMHRAQRPLRVTAGGFFPLSRESFVSVVNVSYSFFAILRNFKMEEQ

>LmigOR22

MPPSLPPEAAAASSDLGYLLTLLHWTAVLRHPRFIGTSPFWFRVYTLTLLTIDASFVFSVFIVLFREGTEDLDVFTLTLSVADTNGTWLFRLAHTVACEAAFHKLSQQVGHDFAEFLTWDDIPLLTSQSRVVRRFSKLYMWGGVGACAYFLVSPVCPEGLPYILALPFDAMQPLGFAVTWVFCSVVTLHAVVMTMVLDSFNVSVIAQLRIQLKLLSTKIVNLSKEILNTDMESSEANVYQELYYRLEKCIRHHEAIIKNADLLERSLGTMLLAQSISIGASTCFQMFQLATRANGLQQAGKFGCYLFAMLAELFVYCWFGDDLITESENVALAAYEAVTSLQGCPLSIKRSLLLLMHRAQRPLRITARGFFPLCRESFVSVVNVSYSFFAILRNFKEEEQQPD

>LmigOR23

DLDHLLRPLHWTATLRHPQSAHSSPLLFRLCKLVIITIIFSFFCSEATVLFRVGAGDLDVFTITIGVADTNIIWFCRMVHISVCERAFHKLALQVGQDFAEFLSWEDLPLLRAQSGAVRRFTRLYVWFGVGCVVYYLFSPASDAGLPVILALPYDMHRPLAYALTWLYVSVTTFHVVVMTMVFDSFNVSLMAQLRTQLSLLSRKVVSLAKEMSEKPVHSPETSAYRELHSRLEKCVRHHQAIINNSDLLERSIGPMLLAQCLAIGACACFQMFQVATNTNGLQETGKYGAHLVVMLAELFEYCWFGEGLITESENMALAAYDAVTSLQDCPISIKRSLLLMLQRAQRPLRITAGGFFPLSRESFVSVVNVSYSFFAILRNFKNEEE

>LmigOR24

MAPAAASSSAAAAAAASASDLGHLLRPLHWAAVLRHPHSAAASPLFFRLCTVAMASFAFTSTCSEVTVLFRDGTADLDAFTLTLSVVDTNTIWLFRMAHTVACERAYHKLAHQVRNDFGEFLTLEDLPLLRGQSRVVRRFALAYIWFGVGACAYYLVSPVSAEGLPFILALPFDATRPLSFAATWLFCTVTCLHVVVMTMVLDSLNVSLIAQLRIQLTLLSGKIVGIAKEMSEKPVRSSETSLYSELHYRLEKCIRHHQTIIKNADLLERSLGAMLLAQSVAIGAAVCFQMFQIATSANGLQQTGKFCCYLFAMLAELYVYCWFGDDLITESENVAQAAYDAVTSLQECPVSIKRSLLLLMHRAQRPLRITAGGFFPLSRESFVSVVNVSYSFFAILRNFKDEEE

>LmigOR25

GPALHPGAALRLVHAFGLRRHVALLHGGHRARRGDDHGVGRLQRQPHRSVAHAAHVAQHRKLVTIANDSSMRPLDSLRTTDYRELHCKLRKCVLHHQTIIRNADLLERCLSGILLGQSISIGSVACFQMFQVALILLIYNWNHRTSLKKLSFVIGKFGFYLVAMLAELFIYCWFGDDLITESENLALAAYDAVTSLQGCPMSMKKSLLLVMHRAQRPLRITAGGFFPLSRESFVAVVNMSYSFFAILRNFKDQKV

>LmigOR26

EGLPLQLVLPFDTSRPAGWAAGWLFCAFLTVHCVVMNMMADAFNVSLIAQLRMQLMLLRRKIVRIAEEGSSRSLDSLHTTDYRDLHYRLHKCVLHHQAIIRNADLLESCLGAMLLGQSISIGTVACFQMFQVAMSANGLQQVGKFGCYLFAMLAELFIYCWFGDDLITESENVALAAYEAVTSLQGCPLSMKKSLLLVMHRAQRPLRVTAGGFFPLSRESFVSVVNVSYSFFAILRNFKMEDQ

>LmigOR27

MAAAAPESSGGDVEYLLRLLHWSGTLRHPRAGRGASLAFYARNAAVATAVLLFVLSQAAVILGEGPADLDRFTRALCFFNTSLTWLLRLAHVALREKQFHAIALQVGSDFGEFLTPRDAETLGRRGRALRRFVLAYLCFGVAAGAGWDVFPVVRHGVCGDGLPFHMALPYRVDRPLPFAATWFYCFCMTMHVAVVTMVFDSFNVSLMAQLRQQLSVLSGNIRSLADEQRRTASSGVDSPEHTERVRYRIRTIVRHHQAIIRNVESLEKCLGDMLLGQSLSIGASICFQLFQSAESLAEAGKFGCYLSVMLAQLFVYCWFGDDLITESEKVSAAVYSVVPSLQGCPTSVKRSLLLVMRRAQRPLRLTAGGFFDLSRESFVQVLNVSYSFFAILRNFKEE

>LmigOR28

AARGVTSYYVGVGGAAVVVWNLMPLLLEAGRSLPTIAWYPYDETKTPFFEVTYLLQGISTFYCCITNVGLNVFLVSLMIYISDELKNLNDSISSINYRSIYNCCCNGDKQIGLLNWSSKIVTDDCDINVDTHIAHSKHIRQRAEFCCVVKAQDYLRMCLQYHQELIRTVKKLETTITAIVFIEFVAGIIVTCLTLFQAAVNAGNMALFVKFIMYLLYMTVGMFIYCWYGQDLMQKSEDIKWAAYSCNWQGAPKSFTDLLKIVILWAQQPLTLSAGKYYKISLKTFVTLLNASYSYYAVLRQMSDTQK

>LmigOR29

PPAAGLLYRAYTAAVVGLLCHITLPEAAGLVHFRGELRTATEVACLLFAFATTCYKLLAVLLRRRRILRFVHDLDARVAAMAEESAEARAAVSRRDRWTRRLAVLMVLQSTSTAFTWSMNSLRLSLSKGCSLRFLPIISWYPYDMTVWSNYAITYIIQFFTLIASAFSNRTCDILIITLMCQVGSLLEMLNLRFLEISRDSQSKADKRRTEWNQKHIMKRATSVLCKTIGANGLLDHVPTTSEEVPQDEMYAKLTRCIKAHQEIIRYAKELESLVNDIFLVDFLCCMIVICSTLYISTSASSNFGDLMAHFGYLVAMTYPLLFYCLFAHDIMEQSGRVAVSAYCLPWFLGNTKYRRAVCVALCRSQRPLTLTAGKFSVVSRATFLAIMNASYSYYQILREINEVKRSE

>LmigOR30

RRLTLAVTLLGCPSPGVWTAVPLLAPLVGDGAAPRNRSLPAAARYTARDTESPRFEALTALQFFSMQFSYFTTVGVDMLVVSIMIHASAQLELLNLSFGRLGQAAGSLPGNRERRRAEVCVREAADGPGRSGISRRADAEETPPREKFCQELRDCIRHHQDVIQLVADVERLLTSMILTQVLGATLIICVALFQFATNIENIGTILRVSVYMSFMVNEVFMYCWFAHNIIDQSSRIAESAYSCAWPGVPPSLQRSLLVVICRAQRPLALTAGKFYQVSRETFIQLINASYTYYALLRQMND

>LmigOR31

MCVRVPWRDSALWANARLLSVAGVWPPPSSGGWYLLYTCWLFGSQLFMVAGQLAGLWRFRGDLDKLTLDVCLTVTVVMGVIKAGAIVARRRRFFGIVRRLDAATAAQLLAGDPEEAAVVASAASLARTITVWAPVLGSLSPVVWGLAPLLLRLLGNAPRPRELPVVCWYGGWDAASPYYELLYLVQFVTIQGGYLVVMGSDLFFVSLMIHAAAQLRILNMRLVKIARNEVENDKTFGGMKCVPGYRNSNLKIIDKEWTSSATKTQDLTDETSSYDELRSWVEQHKDVIRLVQQLEQLLNVIILFQFLGGTIIICVTLYQSSAKTGEVTTLFKLQLYLGTMLSEIFMYCWYADGIVQQSARLATSAYSCGWPDAPQPFRRSLLIIMRRCQRPLSLTAGKFYTISRATFVRLVNASYSYYALLRQMNDH

>LmigOR32

ATARYTDRDTATPVYELLCVLQYFSMQYSHFAAMCIDLFFACLIIHVAAQLAVLNVRIGQIREEYYRGGAEEAVPPEERRAREDEAWQQLRECVEHHKLAIKLVGDLEDLANVIILSQFMGATIIICVTLFLITTSEQHFAALVKLQGYLIVVVYEIFMYCWFGDDVMYQNSRLATSVYTCGWPGAPQKLQRALLIILMRSQRPLGVTAGKFYRVTRETFVSLMKASYSYYALLNQMNK

>LmigOR33

MQVTRHERRFCPQPGTRTVSFLIRYRESIRTIGAIFQKHPFPELFNVVMQVTSHERRFCPQPETASMREEDIPWSDTVLWLNARVLALGGSWRPPGARGFALYRLWVLFTQFSFLIGQLQGLYYFWGDTNRILQDVCLLITTILGLFKFFTFVARQEQVFRIVRSVDDRRREQSKLGDARVTSVLEASRRAARTITVWMAGVGGLAPAVWASMPLVMRGLGLAPPERELPARARYTDRDTATPVYELLYLLQFFSMQYSYFAAMCIDLFFACLILHVAAQLEVLNVRFGQIREDYYRNGQTREARDDAKVEDVEEDAAWKELCECVEHHKGAIKLVDDLETLVNPIILSQFMGSTIIICVTLFLITTNKQHFAALVRLKAYLAVVVYEIFMYCWFGDDVMYQNSRLVESVYACGWPGAPPRLQKALVVVLQRAHRPLGVTAGKFYRVSRETFVSLMKASYSFYALLNQMND

>LmigOR34

MAGNIPWTDTVLWMNARVLALGGMWRPPWFQPKWYLLYRAWVLFTQFSFLFAQVQALWFFWGNIDKITHDTCLLITTILGLVKFFTFVLRQEDFFRMVQKIDDSRAEQSKSGDSEIVSILDASYRSARTITLYMTFLGGSSPGVWAIIPTILRRLGVFPPERELPATAWYSRRDTETPYYQMLCTLQYFSMQYSFFMAMCLDLFFVCIIIHAAGQLEVLNARFRRVGQIAGNHSADSHKQQKALEEFSGDVFSPEEIWEDLCDCIKQHQDIIELIKEIERLLSKIVLLQFLGATVIICVTLFQSSKNTDNIAALLLLQAYLGVVIYEIFMYCWYADDILYQSSRLAMSAYACNWPGAPPQLQRALVFIIRRTQRPLGLTAGKFYYVSRETFVRLMSASYSYYALLNQVNDK

>LmigOR35

MAVDAPWSDTALWLNARLLALGGMWRPPWCPARCYLLYRAWVFFTLFSFFVAQIQALWHFWGDMDKITHDVCLMISIILSITKFFIFNFKEREVFRLVRRIDDTRAEQIETGDSEITSILDASYRSARGVALMMTCLGGSIPGVWAVIPILMRRLGIFPPERELPGTSWYTGRDGETPIYETLYVLQYFSMQNSFFTAVGPDLLFVAFIIHAAGQLEVLNARLRRVGGASDARKLQKAREEEEESGEAGCGELAWRELCGCIRHHQHVIGLIKEIERMVSKIVLLQFLGATVIICVTLYQSSKHTENMAALLMLQGYLGLIMYEVFMYCWYAEDILYQNSRLAVSAYSSGWVGAVPQLQRALVFVICRTQRPLGLTAGKFYYVSRESFVSLMSASYSYYALLRQVNDK

>LmigOR36

MPAAAPWSESALWLNARVLALGAMWRPPGCRGPALSACWALYTGWMLFTQLSFLVAQARALWHFWGDVGKVTHDVCLMVTVVLGLIKFGVFSLRKDDFFRIVRQIDSARSEQSRSDDAEIASILRASYRSARNVTLYMTLLGGSSPAVWAVTPALMRKLRVGPPERELPATAWYSGRDTDSPRYELLCVLQLFSMQYSFFAAIGLDLFFVSIIIHIAAQLQVLGVRLRGIGKLGHKQSSSLSNSPILLRDVEDGFYNEKSLWMEFCSCIQQHHSIIELVKEVESLLNIIILLQFLGATVVICVTLFQSSTNTSNFMTLLKLQAYLMVIIYEIFIYCWYADDILYQVNSFTSGIYFTVRLQDLDASQVLQGIREWDAVVEDKRGGGEPDTSSRLSAAAAGKFYYISRATFVRLISASYSYYALLNQMNDK

>LmigOR37

MDAREGKISSVQSQCKPGGIIPVVELVIPDAMKRTGCGLLQADALCDYFRLVTELDAFVSEQRSKYYNNEKVIEMLDASSKRTASITKAVMAYLICWTFIVVPALFLIESPYSVLPLMAWYPFTANIWPRYEIIIMLHFLTIGYCFFTSWGMDLFFGCLMYHLSLQLRLLNYHLANIRYRCKSECVLQEGFEGKTDFSTPRVELEVIRNGREKQQVAEYTRSAEDAIYVDLLQCIKHHQRIIRYADNVENVANPVILSQFVLSVLVLCVVLFQTSSELGTLTALVRFLVYLLELLLQIFIYCWVAHQIFEECDSLLQLSFVVKTLDGRRAIRTRSVKCLLQESFWSVMIMPRPVTFSAGKIYAIDRTTFVSIVNASYSYYAVLRQINN

>LmigOR38

MKEIRNLTISNANNSMKVRHAYRLGINSEGDRSDDGLGRDRNQSFDNRAFIISVQPSESSVGAFLRKRFIVKYLPKVLSTVHRLARSITLAMLVFGAASSIGWILNSLLLGGNSMLPMVAWYGIDQASSPTYEMLYVSQSLVIFYCFLTSWGLDLLLASVMIHIAALLKTVCIHFSLVTAVSSNRTPGMQMLQRNKTLPTAGNNLETVVLEMTSTDYKYLQCVKCIQDHQRVIAMVKDLEDLANPVILTQFVAGIVVICVNLYHTTTDTHGFLWASKFASYLLMLVFQIFIYCWCAHNIMEQNLLLAEWTWENGWRLWRAAVDLSPGAGGGGRVNGYACTLLFVSVAGIGLFSSSLSSFRKLINTSYSFYAVLRQLNSR

>LmigOR39

MSKELFSLPGPNSVINVNIGVLKVAGLWPTRPYGLFTIYTVWIYLTQWAVFALDFMSLFYYWGNLNMITAVFCNLTSITAGIIKMTHFFVYKPKYYMLVNKLDALVDSQQKITHPNVDSKSILLQTSKLNKYSTYIIVTYGNLVGVPWIVLPFVVDSGDTERTLPVVEWYGITQDKSPVFQIGYVLQCLTIMYWFFASWGLDLFFGALMIHLAGQFRILNNRIANVGREVDPRLDVSSTLKIEMKEINIDAATRTTLVQRKDAELYSELRKCIMDHQEMISFMNDLEETVNFVVLVQFMAGTLVICVNLFQAALNVQDFSSVLKVCMYMFELILQLFIYCWCAHDVMVESERLSTSAYFSEWAGAPRRFTTALHILMARAQKPLTVSAGRIYTINRSTFVSLINASYSYYAILRQMSDR

>LmigOR40

MEGRLSARHRESVLKWNVWVLSSGGLWPAGPPRLFAAFTSFVFIVKWTHVLMAVRTLYLSWGDLNEITLTLLSMITMLGGSVKMTLFLKNKSAYYQLVQRLDEVVRYQEQYYLGNETMVSTFQKARKKALRLTFITLGYLNVLGPLWFVMPLLENSSEKHLPFIPMHGLNVTSLPLYELAYVTQCTATFFWHLVSVGLDMFYASVMIYVTAQLTILNLRFMNLGLETKDFIGRPSLGSITASFDNRMFADAHDKMYKELCDLVRSHQKIIEFTNYLEQVMNSTVLVQFLSSVLVACVTLFQATINSQGNTVVKCWLYLPMPAFQIFVYCWCAHDLMDQGLEVSTSAFLSAWVEGSRGLRRGLLLVMVRSGRPLEH

>LmigOR41

MAHAQRPRGLPPANVLSANIAILRWSGLWPPERRGGWARLFAAYSAVAFLSQAVAAGMTLHLIYHSWGDIYEITLTMMVTMTLVGGVLKMLHFFRHAGAYHLLVRELPAVAAGMTLHLIYHSWGDIYEITLTMMVTMTLVGGVLKMLHFFRHAGAYHLLVRELRRAALFTRGALGYLNILAPTWFLMPVISGAADDPAGRKLPFTQLKGLRADDLVGYSVAYFVQCHAIFYWNFISVGLDVFFATAMLHAAGQLKILSHRLSRLGKGPAARQHWNPQDDTTQIRQIMPQEGTNDLYTELRSCIKNHQEILRLVLLLESVMGPVAFIQFLCSVVAACVALFQATFNAEGNGVLKCTMYLPTPAFQIFIYCWCGHEIMEEFSRYSLATRRACXYSSGWVGAGRRVSRGLRVLMCRAQRPLLLTAGKLYPVNRLTFVSLINASYTFYALLRQMRDR

>LmigOR42

MRGRARLFALYTAALYACFAAVLLMALQLAYLSQRDINELTYALIVVMSHVGVLFKMTHFLTSRGAYLQLVERLNRLVGQSLADDSSGPVLAACHRKAMRLTFCSFAYLALTGAIWYLVPIVDAIRSGSEGRRLPVANPHWIDTSKTALYAFLYVVQFPSIFYFVGISVGLDGFFATTMIHVATQLRLLSLRLSGLNHGGSRISSVTKFSEDNKSDLPSPLRVREIERLDESEGMYQQLVQEIKRHQEIVSFVKFLEAVMSPVAFVQFLFSVGSICVTLFQSTFNPRPDVVLKCAMYLPTPAFQIYIYCWCGHDIMEEGARVSLAAYSCAWTGASKRSKDALRMLACSTQRPLLLSAGKIYPVSKATFLSMINASYSLFAVLQQMRSR

>LmigOR43

ERRPRLYALYTASVLASQAACIAMGLRHALDSWPDTDAVMLTFVNTATLLGGVAKLAHLCAHVRDYRRLVAALRGLVAAQWPACRRDPRLLAAFRRSYRRALRLTFGMIAYLHFIGPIWYAMPLVARATGGEQRQLPFVDLRGAVKEDLSLYVSVYLLQCHAIFFWCFVSPSLDMFFVTCMLHVAAQLGILNARLSELGGVRVPDGGEMVALPAGRRKSRNLEQHSDDSDISEELRDCVKIHQDILSFLQDMQRVMSKVAMAQFVCSSVSICITLFQATCNPEGNSNLKCFMYLPMPAFQIFIYCYGGHELIDQGLAVSLAAYSCAWVGATRRVTSSLHIMMCRAQKPLTLTAGKLYPINRITFVSLLNASYSFYALLRQTRDR

>LmigOR44

MSRAYIAHVGNSLTESFSPKLLRKRTVLFFLSLRSRGRALEMPEQKVQLQRGARICDVLRHNVLLLVATGAWPPTTRRWWRPLYPLYTASIYFSMLATIAMGFQFAYQSWGDWDSIMLTFVNTFTLIGGAVKLAHFSSHVDAYRRLVTALRDVIGTQWAHCERDAALMAAFAGSHRKALWLTWAPVVYLNILGPVWFMMPVVAWASGAPGRQFPFANVRGVLKTNFPLYVAVYFVQCHSVFYWNFLSFGLDIFFVTCMIYVSAQLHILGKRLSNVGRGPNVDQNGIVDEKQTKLQQFGQKPYKSLEVDRESNEMYAELVDCVKAHQHILSFVAVLQGVMSPVAMAQFVCSATAACITPFQATFNPEGNSIFKCLMYLPMPAFQIYIYCWGGHEIIDEGAALSASAYSCAWMGAPRRVTSAMHVLMCRAQKPLTLTAGKLYPVNRDTFVSLINGSYSFYALLRQMRGH

>LmigOR45

MAATERRQGEVGEDEVLRSNVRLLQLAGAWPPTAPRGLGRLFPLHLASVYLSQLANIAMALRLMQTARGDMHEITQALMNAMTLVGGILKLLHLSTHVPAYRRLVLALRDVIRIQRHQCERDPHVASLLARAHRKALRLTFWPIAYLNMLALGWYSIPVVFWALGWEKRLLPFFTLHGVDSYDFVLYAAIYFVQCHAIFYWCFISIGQDMFFVTCMVHVAAQLQILNARLSNLGGGQGASNELGRLYSCRAEEDGPCERKPISDTMYTELRNCIKTHQEILKFVQLLQQVMSPVAMAQFLCSVGAACVTLYQATFNPEGNSSLKCLMYLPIPAFQIFVYCWGGHELMENEENIGSVRACRSSASQWLFAGRRVTSSLRTLMCRAQRPLLLHAGKLYPVSRDTFLSLINASFSFYTVLRHMNNR

>LmigOR46

MAVLEASGRQTLGEATELWGDVLRRNVKVLRFGGAWRPAALVGWRPRLFPLYFGSVCGSLLNIITLDMVRSWLLWGDMTAVTFALVSAMTNLNGVVKMVHCFRHHGTYGRLVSELNGLVALQRPYCEADGALLAAFRRASRRAARLTVGCLAYMNVLGQMWCVVPLLTPEAPDSRESALPLVSLPGLRSRNRLWYSFAYLVECHAVFYWNFASLGMDMFFASAMIHVTGQLDILNIRLAQLRREGSTEDQFRSFASDAGRSDRQRERGDGDSSKMYSELCECVKHHQAILKYLEFLESVMSPVALTQFLCSVVAVCVTLYQITFNPEGSGVIKCAMFLPIPALQMFVYCWCGHDIMEAGLSVSLAAYSGAWVGVGRRVTGALRVLMCRAQRPLQLTAGKVYPVNRDTFLSLINASYTFYTLLRQMRNR

>LmigOR47

MVAIGSRAKPILVQQSKTSSKGQDGGEESVVSPSENVLRRNIRILRLAAVWRPPGRWRQRLYPLYFGTVCTSMLHIGALAILRSYTIWGNMTEVTFALVSGLTCFNGAVKMIHHYTHSETYYRLVDELNLLIDRQRPYCEGDAELTEALQTAYKKAKRLTWGVLLYMFVLGQMWCIVPLFMKFPPDDPSSPLPLVTITRVHKVHNHTLYSMAYLSECHTVLYWNWSSLGMDVFFGSIMIHVTGQLNILNIRLSRLSHEGVGDGLAQYSSFVKGSELHKGGIHDSASMYDELCKCVKDHQEILRYLDFLESLMNPVPLAQFLLCVGGICLTLYQITFNPDDGGVIECILFLPIPALQIFIYCWAGHGIMEESEYVSFAAYSCRWGGAERKVTNVLRIIMSRAQRASLLTAGKVHPINRDTFLSLLNASYSFYTLLRQMKNLEEENEASS

>LmigOR48

MASPDALLRHNVRLLRLGGAWPPEHGRGLRRLFPLYTASVYFCQSATIAMGALLTYELWGDVDAIMLTYVNTFTLLGGFVKLVCFSGDVRGYRALVAQLRAVARHQWPHCQADAHLMAIFGAAQRAALWLTFGPLAYLNVLGPTWFFMPLIVRATTGSHQRLLPFVNMRDSVTEIFPLYVAIYVVQVYCMFYWNIISVGLDMFFVSCMIHVAAQLRILNERLSNLGRARADDDDDSCRAEANHKNIPGFGSSQKRSFSREGRVGNMYEELRNCIKTHQHILSLLKTLQRMMSPVAMTQFMCSASGACITLFQATFNPEGNSTLKCLMFLPMPAFQIFIYCWAGHEIVYQEELLSLSGYRSGWVGCGRRASALLHILMCNAQKPLQLTAGKFYPVNRDTFVTLINASYTFYTLMRQTRDQGSIVQT

>LmigOR49

MESSARHDDSVGAKRGAVRLTLWKGPEQTTSILRLNVRCLLLGGIWPKSRGALYLAYSAFIQLCSISYIVMCMLSIFSPDGDMNDITLTLLHTFEVVCGVVKAAIFYLKRHQYYQIVRDLDQLVSSQRQYLTASKDDHLLAMLDAAHKKANFLTLVLTGYIYGLVFVWLPFPLILSPSDRLLPFVPLPGRYYKDSLLRYITVYAIQSFVPLALIMVVDGLDCLFVASVVHAEALLKVLSERLASLGHSHYTDFRLQRSSGREQEPRKGNVGDNSNITAQLRSCIIHHQQIIEFLQNLEKAMNIMVLIQLSFAMFNLCMALYQQTKIPDFTSALKYVMYLPFPTMKIFFYCWAAHNVKEQGEEVSWAAYSCAWPDADQEFQKSLAIIMCRAQRPLLMTAGRIYPINKDAFVSLLKGSYSYYTLLRQFESK

>LmigOR50

MKRGDTAVEEDEKDGEVLTWQETGGSVLKYNIRQLHVFGVWPLPGSAPFHAYTVLVSAIGLAGLAQDLAGVCACWGDLQEVTMALIHILSVSSGFVKLAFFVRRRRHFNALVRRTDRLVAAQGHFCDADATLRATFRASHRKAVYVTLLAYGYLSVQGVIWLPLPLIAHPGERRLPFMQMPAAATASVYVYALLYSLQCLSSMFVTFVGVTVDCFFAVVMIHTAVQFRILNTRISALRADAAVPVDTGTAVGGQTSEHDSLYKQLCQCIQTHQRLLRFVTYLDSVMNPIAMTQFTFGVIVVGITLFQASYSPSSSTMFKCVTWLPMPSTQIFLYCWGAHDIMDEGQSVSRALYSCGWVDAPPGFKRALRLVMSCAQRPISLTAGRVYAINRATFISLMNAAYSYYTLLRQFNSR

>LmigOR51

MKKEKERRVQLEDLGSGEDLSRAEVESSVLRQNILLLHYMGLWPMGGSRAYRCFTAFNLSSSATIIVMNVVGVCFSLSDIDQVTGALSTILPMSGGLLNGLFLLHQRPTLCRMVRTVDRLVTSQAQFVERDARLVAIVGGARRRTLVVTLGVSGYLFAIASYWVVIAFTLPPSLRVLPFVQLPWMPSSDPGLFWSTFGAQLYTAPFCSYTTLFVEFFFLAVMLHLSAQFRVLGSRFASLGRSSASKVAADSGAVYEELRLCVETHQELLRFVRFVDNVMSPFAMLQFVAGTLAVCVVLFQAANNQDLNTNLKCAGWLPGPSLELYIYCGGAHEVVHAGEALVQAAYDCLWYNVAPRIGRAIRLVITRAQVPPVLTAGHLYPITRPTFVSLVNAAYSYYALLSQMQNK

>LmigOR52

MAKQKEKSSRLQDLDSGDGEDLSWDEVSHSVLKNSVRVLYCMGLWPLRSSRAYHCFTAFSLASSAVVIAMDIVAACYSLGDIDQMTGALSTILPMSGGLANGLLMILHRPDLCRVVRAVDRLVVHQQRYLRQDSHLAAVVARVRRQTLLVTIGVSCYLITIASYWIVIAFGKPTGLRVLPFVQLPWVQSSGLAHYWSTFAVQFYTAPFCSYATLSVEFFFLAVMLQLSAQFEILGSRFASLGRNPPPKAVVTTKSDMGTADSDAVYEELCLCVKTHQELLRFVRFLDDVMSPFAMLQFVAGTLAVCVVLFQAANNQDLNTNLKAAGWLPAPSLELYIYCGGAHEVVYEAEALVQAAYDCLWYNTAPRVSRAIRLVITRAQVPPVLTAGHLYPITRPTFVSLVNAAYSYYALLCQMQNK

>LmigOR53

MSAEPAQTWRSSASSVVSYNVRLLFLCGLWPLRRGRAFSAFTAAVLVAAALHAAGAFVGLCTEPGGLQEVTLALANLFVVCSAIVKSCFFLADRTRFCTLVSTLDRLVQVSGQQSAVGAGLRSRLSASARRAVRLTLAFHLYVLSALVGWCLMPALKRQRRLPFQQLRWLDTSSAAVYGASYALQCFATFFCSFINTHLDVFFMAVMIHVADQFAILAARFADLRLDADADPEGRLPQGERAVLAEDAYQQLRLCVRSHQELVRLVQLLDDVMSPIAMTQFVVGAINACMVLFPATYSTDVGAVLKCWAALPMVGIQIYLYCSGAHDIMEEAGAVSGAAYSCSWLGADRRRRRALLLVTCRAQRPLQLTA

>LmigOR54

PRLAAAFRESCRWSAKLTFLFFSWVLLALVAWSLMPLTLYPRVRLFPFQQLPWPVLTQSPTYWFLYLHQILATFFFCSIDMNTDCFFATVMTHMSTQFKILASRIADLRLRENTQKSKLCAEVDTSTPHDEMYKELCLCIETHKELIRLVGLLESLMNPVAMLQFLVGAVSSCVVLFSATYSPDSSSAMKCWGSLPLLLTQLFLYCSGAQHILDESE

>LmigOR55

MVDRSVETPLTWRESAESVLRPNIRLLCSLGLWQPVDSMLFHAFTAAVLAVGVAHLAVAALGIWQRPADLAEVAIGLSNAFVIFTALSKAVLFLTRRPLFYSLARLVDQMTAEQKAFRAGDPSLQEVFSAARRSAGRLSVFFHWYVLVADVLWSLIPLVQASREKRWPFQQLPLDGWATSPAYQLSYGLQCASTLFFSLISVDVDCFFVAVMTHITAQLKILTFRFAAIGNRMYISDTSLNNQTASTKDASHEKLRGCVQTHQNILRLVSFLNVVMSPVAMMQLAVGVVSSCMVLFPAANSTDSAVVMKCWAALPVLGVQLFLYCSGAQRLIDQAEAVSGAVYSCAWPEAGGRVQRSLLVVVSRAQRPPELTAGRMFPINRPTFLSLVNATYSYYTVLKQVNSH

>LmigOR56

MDQRATSQSEEEEWAGQSVVRTNTHLLRLLGLWRPARSRLYDAYTAAVLAVGVADLALASAGLWLRPGGLAEVTLGLANLFVILTALSKSVLLLGRRPLFYELVRRVDGATAAQRPFCGEDPLLARLSADARARADRLSRAMHWYVVFAALSWSAVPLLAPPGDRVWPFQQLPPRPWARSPLYEASYALQVAGTTYFALINMDSDCFFMAVMTHVSLQFRILASRFAKLNSTEESLADKKASDVRVTSSESTLPVDDTDRELRACIQTHQKLLRLVNFLNDVMSPMAMMQLALGVINSCMVLFPATYSEDSSDVMKCWGALPLLAIQVFLYCSGAQRLADQLVQATYSYYTLLQHFNSH

>LmigOR57

MGWDSKEDQPLTWQYTADSVLKYDVRILHMIGVWPLSGSQLYRCVVTVIIALCLGHFVEAVINLYTLHGDLEDFTLALSNVSVVIVGILKVTFFLRHERGYCHLVRWLDTIVASQREYTRGRPHLEEAFAGAQTLAVRITRGFCMYNATVVLAWVLAPLAAPPEAKRLPFQQLPFGEGSPFSLYALSYAMQGVSMLLIALISVQMDCFFTAAMIHAASQLRILNSRLSDLQLGKAGLQLQGGTTLDSMYDELRLCIHTHQEITRFVEHLENVMNPIAMMQLAVGVFNGCMLIFPATYSAENDALVKCLAAAPTISAQLLLYCLGAHSVREQGEAVPLSAYSCGWADASAAFRRSLLVVMARAQKPLALTAGRIYPIQRATFLSLLNAGYSYYAVLRNFNSR

>LmigOR58

MGWNPREEKPLTWKETANSILRFDVRILYVVGVWAMPVTKLFRAYTAFTLVLAVGYSVEAVIHIWLVRNSMEEVTLAVSTYAVVVTSACKLVSFLQHEPGYWRLVRWMDAVVADQRHFCEERPELRAIFDEARKRAKRYPNALRVYNTSLIISWVFIPLLAPPGLRPLPFQQIPLSETEDFPLFLFSYLLQTFGMLFMCLVSGCLDSFFTAVMIYTAAQFRILGLRIAALRQDNDEIKRQARSDVYEKPRKGAATDHVYEELRLCIRTHQEITSFVTHLESVMNPIAALQLITGVINGCLMIFPTAASSESGALLKCIACVPTISAQVLIYCLGAHAVMEQSEAVSAAAYGCAWPDTSPRCRRSLLVLMTRAMKPLTLTAGGIYTIERSTFLSLLNAGYSYYALLKNFNSR

>LmigOR59

MSWGSEEVEQLTWKYSAKSVLKWNIRILNLVGLWPLTESLLFRSSTAVIVMLCVAHIAEAAVNLGTLRGGLQDFTLALSNVSVVCVGVLKLTFFLRHERSYCRLVRSLDVLVDSQREFVQGQPPLAALFEATQKRTVRVTVGFLVYAITQLVAWSFAPMIAAPGTWRLPFQQLPLTDETAFLIYELSYAMQVVSIIFIALINCQMDCFFMATMLHTAAQLRILSARIMCLKLQNEDIPAVFRSNEGEEVQASATHDSTYRNLCLCIKTHQELVRFVRHLDQVMSPIAMMQLGLGVFNGCMLIFPAAYSAESDALVKCLAAVPTISTQLLLY

>LmigOR60

MDWDPKETEPLTWQYTTHSVLKYDLRILHLLCLWPLPGSLFFRLLTAFLIALCLGHFVEGLVNLCTLSGDMEDYTLALSNISVVTIGTVKTAFFLRNERKYFRLVRWLDALVAAEKKSVSSRPLSEAIFPAAQKRSARVAAFLLLYNCFLLFIWLTAPLAARPEARILPLQQLPLTDSNAYPLYELSYAMQALSIFFIGLINVHLDCFFTVAMIQTAALLKSLASRLADLQVRNAPSRRNVDEGRKNIVTADDMYRELCLCIRTHQEITRFVQHLENVMNPIAMMQLALGVFDGCMLIFPAAYSSETSALVKCFGAAPTVCMQLLLYCLGAHSVREQGESVSVAAYSSGWADASARFRRAVQVVITRAQKPLVLTAGGIYPIQRATFLSLLNAGYSYYALLQNFNGR

>LmigOR61

MQSKDPVSKSVSFRPPDGGSQNKDGDSAQPTESQDSFLTWKGSSESVLKYNVRCLCILGVWSLTRSRLYYLLSGTAFLLGVMHIVVAIFGSYLYRDNMEEMTLIVANMFVVCAGVTKLVIFVVYRNNYRQLVTVTDGLTDRQRSYCQGDPALKSILEDSERLAVRLTLFVPAYIATLSVVWVPMPLIAYNERRLPFVQLPFVNEVSASTYALLYVMQTVPSLLFFNVGFAVDAFFASVMIHAATQLRILFHRIKDLRLDGRGTLKLLSGDRHDIMYGELCTCIQLHQQLVRYLSFIGKVMDPIAMTQVVFSVLIACTTLFQANYSADTNTAFRCLAFLPTPGTQVFLYCWGAHNLMEQSAAVSEAAYSCSWVEASRRFKRALCLLMCRAQRPLVLTAGGLLQINRPTFISLLKASYSYYTLLGRVNNR

>LmigOR62

MKQSASLEDCSPLTWEYTQQSLLKLNIRLLWALGVWPLPGSWVFSLLKVWLAALAVGNAVENVLGVWKNWGDLTEVTYSLLNAFTIGAGVAKTWHLSRYQPRYCLLVRRVDRLTRSQRRYCDGDAAMRAVTLGCRRTARRVTLSAFAYLTALCLIWMFMPLVAHPGERLLPFNHIPWEPRRFPLFYELSYAVQSASSVVYVFISFALDCFFAVVMIFLTEQLMVLNLRIRQLYARRDGDGSVLVAKQHSDKTVLDKHEEMYKELCLCIDTHQDIIRLLSFLDAVMNPIVLTQFMLSVMAACVTLFLESYSPDSSSVLNSISYLPTPGIQVYLYCWSAHNVLEEGFAVSEAAYGCAWYEGGGRFKRALRIVMCRAQKPLVVTAGRLYPVSRATFVSLVNASYTYYALLSRVHNRG

>LmigOR63

LTALCLIWALSPVVVHPGQRWLPFNHFPLEPAPLPLYYELSYAVQSASSLLYIQVSFGVDFFFTVVMILITEQLMILNARLAQLHLYAGGGKSRAAATRVMTTAATEDRDEMYEELCHCIDTHKDIMRLISFLDSVMNPIVLTQFTLSVMAACLTLYQQTYSPDGNSVMKSASYLPTPGIQVFVYCWGAHSIMEQGEAVSAAA

>LmigOR64

MPTPTATETMTWSDSGNSILKVNIRELCLSGVWPLTGRKLFRVYSVIIWILGLENIVEAMVGIYLSNGDLEQITLVLPNTFTTAGGVFKMAFFLRDPYSYNALVRLMDELISDSSRYSTGNQQMLSIVRESRRSARRLSVFIYAFISTQIVIWFPMPLIAYAGEGKLPFIQHPWINSTTFPAYDTMYALQCLSSGFHIFISLGMDCFFAVVMIHTAACLRILSLRISALRSGDAGSSEVPVTSACSWGRESVAHDEMYKNLRACICSHQKIIGFISYLETVMNPIAMTQFAFSVLVACVALYQATYSEDMSAAYRCASFLPTPGAQVFLYCWAAHNIMEQGLAVSAAAYDCSWVSGDARFKRALRILMCRAERPLVLTAGHLYPVNRPAFLSLVNASYSYYALLGRVQSR

>LmigOR65

MEHPYLAAVTPCRVMKESGGGQRAGADDTASLTWRQTAGSVLKVNVRGLALFGSWPLPESWLYHAFFAVVFASNLGNMAEAAVGLWLGRGGLEEITLVLPNTLTTAAGVCKMVFFYRDRGRYYRLVRRTDLLAGSQLAASGRHGADAVRQADRQSLQLTYTVFAFISLQIIVWFPMPLYAYRDQRKLPFVQLPWNEDKDIPVYELSYALQCFSSFTIIFITLGMDCLFAVIMIHVAAQFEILIVRIRNLRLDLQTTGVTQSKASLGQLSRNDVSSINVQTDYKEVSHHQQQINEAHDKLYSELCHCVESHQEIIRFVRHLETMMSPIAMTQFVFSVLVACVALYQATYSDDLSAVFRCAGFLPVPGAQVYLYCWAAHHVMEQSEAVSAAAYACPWIEA

>LmigOR66

SYAVQCVAGLWMAEISFGMDCLFASVMILAAAQLEILSGRILKLGQSPYVAKKGSPTPDEIYKELCRCVETHQKILRFVSRLQETMSPIAMTQFVCSVLVLCVTLFNATYNKDIITSLSSMTFLSNPCGQVYLYCWAAHNVAEKANAVSTAAYSCSWVEGSERFKRAVRILMSRAQKPLVLTAGSLYPIDRAAFLSLVNTSYSYYALLGQINNR

>LmigOR67

MEDKLSWTFSGNSALKLNIRHLWLLGTWKLGESRLFKMQSTVAFGLSIWSTVECILAVYFIWGDLEQTTLVLLITCTCSSGVVKMFIFVYDRRRYDSLTLRLDALLSLQTGPCSEDPALAAISDWSRKKASRLTMGLLLFMLSQSMVWYFVPLIAHPEERSLPFVQHQWDNNSLYELSYGVQCLSAVWISQISFSVDCLFASVMILVAAQLEILGQRLINLKNGRDSAEKEEKKQLDSKTGESMYDDLCLCIETHQEILRFVTQLQDTMSPIAMTQFALSVVIACMALFQATFSEDFSAVLKCASFLPIPGGQVYLYCWAATNVTEQAEAVSAAAYSCSWVDASERFKRSLRIIISRSQKPLVLTAGHLYPINREAFLTLVNASYSYYALLSQMNNR

>LmigOR68

MEDKLSWTFSGNSALKLNIRHLWLLGTWKLGESRLFKMQSTVAFGLSIWSTVECVLAVYFIWGDLEQTTLVLLITFTCGAGVVKMFIFVTTGRRYDSLTLRLDALLSLQTGPCSEDPALPAIADWSRRKASRLTMGLLLFMLSQSMVWYFVPLIAHPEERSLPFVQHPWDDGGLFGLAYGVQCLSAAYVSQISFGVDCLFAAVMILVATQLEILGQRLVNLRNGRRVAGRREKEQLARKTGESMYDDLRLCVETHQEILRFMTQLQDTMSPMAMTQFAVSVVIVCMALFQATFSEDISAVLKCVLFLPIPTGQVYLYCWAANNVTEQAEAVSAAAYSCNWVDASERFKQSMRIILSRSQKPLVLRAGRLYPINREAFLSLVNASYSYYTLLSQMNDR

>LmigOR69

VVQSARRRAYRITLGMLLLMFSQYFVWYPMPFYVDPGARRLPFAQHAWDNNTHLYGLSYFAQCAAGLWMTQMSFGLDCLFASVMVLLAAQLDILARRILALGSGAHDEKAEYPEKKPAPRFGDQMYDDLCLCVQSHQKILSFVIHLQNTMSPVAMTQFAFSVLVICLGLFQATFSEDFSAVFKCASFLPIPCAHLFLYCWAANNVTVQAEAVSAAAYGCSWVGASERFKRALRIIVSRAQKPLVLTAGHLYAIDREAFLTLVNASYSYYALLSQMNNR

>LmigOR70

MEMYQGKGMLKTLSWTESGRSVLKLNIRHLWLLGVWPLGHSQVFKVYTSFTFAMGVWSVVECLLAVYFTWGHLEETTLVLIFTSTCSCAIIKLVFFLRDERSYSLMVREVASVMAAQSEACRDPALAAILRDSRSRTFRLSLGMLLFMFSQCFIWFPIPIVANAGERRLPFSQHGWDNNCHFYELSYTLQCLSGLYMSQISFGLDCLFASIMILVAAQLKILSGRVLKLNQEVIPPERNDSVLLRNQLAGDKYCDKFYEGLCFCIDSHQRILSGGVQTEECLRVAILDVWRVALFCWNWFVTLLQDTMSPVAMAQFASSVVIACMALFQATYTLHSALYHIHEQLLSVDSKNVLSAIFLWSPSPWHSPPDVQLGTIPRSFANGEGESVSVAAYSCSWVEGSAAGRLYPINRAAFLSLLNASYSYYALLGQMNKRSMKDLASHN

>LmigOR71

MEKYRGKGTVESLSWAESGRSVLKLNIRHLWLMGVWPLGHSPVFKVYTGLTFLMGIWSVVECLLAVYYTWGQLEETTMVLIFTSTCSCGIVKLFFFVRNESSYSLMVREVASVMAAQSEACRDPALAAILRDSRSRAFRLSLGMLLFMFAQNFIWFPIPVVAHAGERRLPFSQHGWDNNSHFYGLSYTLQCLSGLYMSQISFGLDCLFASVMILVAAQLEILSGRILKLNQEVILEQRNESVLWKNKMTMDENRDTFYETLCFCIDSHQKILRFVTLLQDTMSPVAMTQFANSVVIACLALFQATYGEDMSAAFKCACYLPIPGGQLYLYCWAAHSVTENGESVSVAAYSCRWVEGSARARHALRTLMARAQRPLALTAGRLYPINRAAFLSMVNASYSYYALLGQMNNR

>LmigOR72

MEKYRGKGMVESLSWAESGRSVLKLNIRHLWLMGVWPLGRSPVFKVYTGFTFAIGIWSVVECLLAVYYIWGQLAETTVVLMFTFTCSGCIIKMFFFVCNERSYSLMVREVASVMAAQSEACRDPALAAILRDSRSRAFRLSLGMLLFMFAQNFIWFPIPVVAHAGERRLPFSQHGWDNNSHFYGLSYTLQCLSGLYMSQISFGLDCLFASVMILVAAQLEILSGRILKLNQEVILEQRNESVLWKNKMTMDENRDTFYETLCFCIDSHQKILRFVTLLQDTMSPVAMTQFANSVVIACLALFQATYGEDMSAAFKCACYLPIPGGQLYLYCWAAHSVTENGESVSVAAYSCRWVEGSARARHALRTLMARAQRPLALTAGRLYPINRAAFLSMVNASYSYYALLGQMNNR

>LmigOR73

MKKSGMEDLGRTVSWSESGRSVLRLNIRHLWLFGVWPLWDSPLFILYTGYGLSLGVWNVVEGALAASCTWGDMEQTTLALMATSTNCNGLVKMAFFLRDRRRYQALVRRVAALVALQGESCAADPLLGRVQRGSRRRAFRLTAAMLLFMFSQCFVWFPMPAVAHADEGPLPFAPDNTTAYGLSYLAQCAAGLWMTQVSFGMDCLFASVMVLLAAQLQIVAGRIARLGEQNYDFAFSFVGHLQDTMSPIAMTQFAFSVLVVCFGLFQSTYAEAVTMAAYSCSWVEASGRFKRALRILISRAQKPLILSAGHLYPIDREAFLSVRS

>LmigOR74

MGDAEQPLSWSETEHSALRLNLRHLCLFGAWPLSRCRAYHAYGAFNLALGGWYVGEATASLCFLWGDMEEATLVLISAFNIASIVLKLALFAARRPRYAXXXXXXXXXXXXXXXXXXXXXXXXXXXXXAARLTAAMLLLMVSQYATWFPMPLLASAGARRLPLAQHPWDNNSHLYALSYAVQCAAGAWMTQISFGIDCLFVSVMILVAAQMKVLASRVASLRTADVGFGRKQHPLSSPGEQVYRELCVCVETHQQLLGFVKELENAMSPIVMTQFACSVLVTCVTLFPATYSTDFSAVIRCAGFLPVPLGQLYLYCWAAHTVSEQAEAVSAAAYTCTWVEASERFKRALRIIVSRAQKPLVLTAGRIYPINRAAFVSLVNASYTYYALLCQINKRSTQRVP

>LmigOR75

MESGAAEDVEGPMGWSESGGSVLRLNVRHLWACGVWPLPGGWLFEAYAALGLALGAWNAAESLLALSFCWGDMEETTLLLTSTFTIGCGSAKMALLLARRGRYRALARRVQALASLQTGHCLADPALDDIRRGSQRRAFRLTLSMLLFMFSQCFVWFPMPVLAHWEERRLPFAQHAWDNNTRLYALSYAVQCVIGAWTSQLSFGVDLLFVAVMILAAAQLRILTIRIASLKTESWKVKPEGGARCEDVRPENGRDVMYENLCLCIDSHQKILRFLKHLENTMSSVVMTQFCFSVLVACVALFQATYSTDFTAVLKCASFLPVPGGQVFLYCWAAHNVTEQAEAVTMAAYSCSWVEASVRFKRALRILISRAQKPLVLTAGHLYPINREAFVSLVNASYSYYALLGQMNRR

>LmigOR76

MNTIDEAVRANRLRWWGHVTRMGEAMLSKRLMSSAAEGPWLETSCQHWCRSRLFGAYTLFAFSLGLWQTAESLLSCYFSRGRMEQATMVLMTTFNIGGSTVKMALLALQRRRYFSLVRRTDLLVTEGAPPADCSKERPRSLPGPVAAAQXAAHGRHAAADRGAHPWDNNRRFYALSYALQCMGAAWTTQASFGVDCLFVTVLMLVVAQLEALASRVRAIRVEPEVGGGAADKAADQMYAELCACVDLHRKILSFVKQLESTMSPIVITQFAISVLVACVSLFPATYSTNFTDVLLCAGFLPVPLGELYLYCWAAHNLTQQAEAVSAAAYSCSWVEASERFKRALRIIISRAQKPLVLTAGRLYPINRAAFVSLVNASYSYYALLGQINSRSTETTH

>LmigOR77

MKRSGMEDLGRTVSWSESGRSVLRLNIRHLWLCGAWPLPGSWLYRWYAILGLVVGAVNAVESAVSLYFYWGDMEETTLLLTSSVSNGCGTVKLAVVMRNQRQYHALARRVQALMALQGDLVAADPALSAVVEAARRRAYRITLGMLLLMFSQYFVWYPMPFYVDPGARRLPFAQHAWDNNTRLYALSYFLQCAASARGTQLSWSLDLLFMSVMLLAAAQLQVLTRRITALQEEKREAKLGNAANSEKAALADGGGNMYDNLCLCIETHQKILSFIKYLDDTMSSVAMIQFCVSVLLVCVALFQATYSTDSTAVLRCALYLPMPGTQVFLYCWAAHNVTEQAEAVSLSAYSSSWVGTGRRIKQALRIIISRAQKPLVLTAGRIYPIDREAFVSLVNASYSYYALLASMSKRE

>LmigOR78

HDWSGDGGSFYWPSYAQQCCSAFWLGKISVVLDCQFAAIMVLVTAQLEILSARLANLRPDGRALRESPNCKVKLSYVDHDSEMYDELRRAIQSHQEILSFVSCLQQVMSPLAMTQFVCSVIVICVVLFQATYSQDFATVLKCVAFLPVPCGQVFIYCWAADNMTEQAKEVSSAAYRCCWVDAGPRFKRCLLLVIRRAQRRLVLNAGHLNIDRAAFLSLVNASYSFYTLLAQMNRS

>LmigOR79

ELSYAVQCVAGLYLSQISFSMDCLFAAVMILVAAQLKIVSCRILKLKAEESGEQGGGSGDVGGGVALENEQKPYENLCQCIESHQQILRFIIRLQNAMSPIAMTQFFFSVLVACMALFQATYSKDFTAVFRCVAFMPIPCGQVLLYCLAAHDVTEQAEAVSQAAYSCSWVEADRRFKRALRLVMCRAQRPLVLTAGRLYPIDRGAFLTLVNASYSYYALLGHINRRSMQDQL

>LmigOR80

MQTGEVEDADGHLSWEETARSVLRLNIRHLHLFGLWPLRASRLFPLYTAYAAALGVWNTAEGFLAVYFSWGDLEQTTLVLMNTFTNASGLAKICFFARDRRRYASLARGADRLARLQAQACARDRALAQVLRRARRAARRLTAAMLLFMFSQCFVWFPMPAVAHADERRLPFAQHPWDNNTAHYELAYAVQCVAGLWLSQISYGVDCLFASVMLLAAAQLEILAGRVAALGERRNGRGGEPLPADAPDSMYADLCACVEAHQKILRFVSDLENTMSPIAMTQFVCSVLVACSSLFQATYSKDLSAAFSSMSFLPIPGGQVYLYCWAAHEVTEKAQMVSAAAYGCSWVGASERFKRALRIIVSRAQKPLVLTAGHLYPIDREAFLSLVNASYSYYALLGQMNKR

>LmigOR81

SRLYDAYSVWTILQLLMTGVGQMAGLQGHWDDLQTVFTSLCFDLTVTCTIIKGSIFVAQRGSLDALSRHLEANARQFCSHLPAERRALLERARNLSRVIVCSFQSVGGVTLVSFITGPLVQNGRDRELIASAPGNATLDRHLGHNYPMLMWWFGGLPVSSPGYEAAYLIMCYWLVLMYICTNVPDAYYVGLINYISAQLRLLHIALRQIAHPGADDALAEKLGHIYGLDVKGGSPHKDGAQDASRDSVYDRLVECIRFHQEIIKCVDEMESLLSLTVLIQFFTSTLVICLTAITVINTEAAYLPTYAAYLATMFYQLFIYCWYGGEVYLESESLQFSAYSCNWPYTDARFRKTLKICLARMQRPISLTACKFYKLSRETFLLLLNGSYSYFTLLLQMNQKDD

>LmigOR83

MDDGALPQPVVDLWRPCLFLRAIGLWAPARLRWLYSAYTVWSLLQLVVSVAGQLAGLQGHWDHLPTVATSVCLIVTSICTLFKASSFALRRGRVDSLVSRISHNLSTFCAHRPRTRAAVVWAARRRATRMFDTFLGIGGVALVFFYLGPIIQNAKDARVAASAAGNETLPPLLGRNLAMLLWWPSGQPVETPAYQLTYVGVCYWLMLLYLSTSTLDAFYVTVIIYLSSQLKVLNVDFLSITEGDDDSPAEETDPLSKRGGKHELEAKLPKGYEEATDQRTQERLLECIIFHQEIIKTVDEMESILSASILVQFLASTLVICFTAFVITTAENKQDLPTYITYLATMFYELFLYCWYGNELLAESERLQTSAYSCGWVGRSAGLQRSLRVVMVRLQRPVCLTAGKFYQISRETFLLLLNGSYSYFALLHQMNDH

>LmigOR84

MAAGELPEPLVDLRLPCGVLRAVGLWRTGEGGLFYSAYTVWCLLMLVCSVLGQLAGLQGHWANLPTVSTSVCLTLTTSCTIFKALVFLRQRRRVDALVSRVERSVSAFWPLPPARRAAMLLEARRSALLMFSIFFGIGAVALSLFYAGPALQNIKDRELIDSAPDNATLDRHLGRNLPMLIWWYGGQPVEAPYYQLAYALICYWFMLIYLSTSTLDAFYVTFIIFLSSQLKMLNAALADAVQSAGLSDELGNRSGGQPTGAAAGRRADYRRLVHCILFHQQIIQSVHEMESLLSPSVLTQFATSTLVICFTAFAVTTSTKKQEMPAYATYLATMFYELFMYCWYGNELLEQSDRLRLSAYSSAWPDAGGHFQHSLCIVLSRAQRPVCLTAAKLYKLSRETFLVLLKGSYTYFALLHQMNDRQYNA

>LmigOR85

VCCRQLSLHASRPRAGAGMEAHLSPAVRALGAVCLWKPSRAGCWYHAATAAGSLAALSLLASVAAGLPAQWARGDITSFSMNAYVCFAIFAAQIKVSTFGYWGGPQRLVAQLGAERRAAGAAEAALPPGRAERLLGESGTLLRRSAAAFYACGHLMMVAWYASPLIANARLAPDPDTNATLPRHLLFDAWFPFDPVPSPNYEAALLYQSVTLYIAFITTAVIDVFYVSVMVYLGVELEILNEAVARSCRPFEEQENKDKDKEGEKKEEGRGQGDDCSLLVACVRHHQHLNRCVGTLQEVMGISIFVQFVFNMLLICVYAFVITTTKSDFGTLAKFAMTLESYLFENLLYCWFGNNLIEQSERLPFSAYSSAWPDAGRRFQRSLRILALRASRPLQVTVGSLYTLSRQTFLHLLNGSYSLFAVLHHLNSK

>LmigOR86

MAKYSVRGGGGGAPALDLRLQLRLLRLAGAWGPSPSASSTSLPYAGYSAAVVLLLLAFVGSQVSAMLHFWGDILSVTTNACVTFTYTMAMFKLLVVLIMRPSAEYLIQELNRCMQEYGRDLSSEKAAVLARCGRLSRHVAAAHVAIGAVAYVCGVALPAARGRLCPSAACRAADGFPVLVWYPFPFTVSPAYELVFAAVSLDFFYGYILSTTLDGFFVTLIIYVSGQLRLLNLMAQNVCAGAGGGGSEASEQRVRDRLAQCVRYHTDVDRCVQRLSALLGPILLGQVLADVVTISATAFVTTTGKTDSGWVFKYGSYLAAIAEQLLLYCWFGNDVLTESERLQLSAYSSQWVSAPARFRKGLLVFLCRAHRPLRLTASKFYTISRETFLLLMNASFSYYAVLRQLNSD

>LmigOR87

RRESLRSHVTATCLMNCGGGRRLRTDLRLQQWLLFFVGAWAPHRGAPAVCSLLYGVYSACVVLVLLLFVASLLFAMVHYWGHMLGVTMNACLMFTYVMNSIKIVAFLKMRPAIDQFIDELDNCMQEYGGEQQSERAALFGWTALKSRIVSVARLSVTAMGCVYWSVMPAVRARACGDTVRCRARVGLPAHVWYPFSYTQSPVYEVIYAGVAAGLMYGALLSSIMDGFLVSLFIYMAAHLQMLNLMLQNLCVDQPQDGSKGLPPGHHQHLCRWRLAQCVNYHCRIDRSVQRLSMLFGPILLGQFMMDIIAISATAFVAIAKNADSTWLVKYTSYLSAVIQQLLFYCWFGTDVLTESERLQTSAYSSQWVDASPLFRLELRVFLCLAHRPMRLTASKFYTISRETFLMLMNASLSYFAVLREINAK

>LmigOR88

MVNVQKAGGGGRRLCTDLRLQQWLLFLVGAWAPHRGAPAICSLLYGVYSACVVLVLLLFVASLLFAMVHYWGHMLGVTMNACLMFVYLMNIVKIVSLLKMQPSAEEFIRELDRCMQEYGQSLEMEKAAVFQWTALKSRIVSVARMLVALSGCLYWAVVPAARAHACGGTVQCRDQVGLPAHVWYPFHFTHTPVYEVVYTVVAGGLLSGALISCIVDAFFVSLIIYQAAHLQLLNLMLAAVGTEKCYQPGPPTSPGSKRRLAEESAGAEQRMHRQLAECVSYHCHIDCCVQHLSSLVGPILLGQFLMDMVTISATAFVAIANNADSAWLLKYTSYLSSVVQQLLLYCWFGTDIITQSERLQLSAYSSQWVSASPRFGRELLVFLCRAHRPLRLTASKFYTISRETFLLLMNASVSYFAVLREISSK

>LmigOR89

AYAGCVVSALLFLVASQLSAMLHFWGDLLSATNNACVTSSYSMAVFKFFAFRAMRPSVEHLVRDLDRCLQVYGREYAAEKEAGFGACERQARLVSRLQVSMGVSVYLSWVVLPAIRAQACRSTDCRVHAGFPALVWYPFAFTEPPVYPVIYAVLSIGLFYGCIIFTSQDGFFWSLIIYVGAHLRFLNFMVANMSSSEADAKIPRGSPEMEENMRSRLKECVRYHNDIDRCVQRLSSLLGPVMLGQFLTDIVTISASAFVATMLKADSGWLLKYGSYLSGTIEHMFLFCWFGNDILTESARLQLSAY

>LmigOR90

MKKASAQYSQESLSARRGLDVSTCERILHWFSCWRGGSAWYVVYVVVARASVAGVVVSQLGLIPHVWGDLYSTSLCVYLTLAVLSAWFKMVQFDLARPKVDALLQELRGELLLHAEDLSEGTEDVFRLFQKRSGAFRRVFLVLDHLTILSWAAKPFLDRLLGSDGPLSLPQDAWYPFDTTRSPYYEIAYLHQVVSLYVAATCVVTVDVFFTTLFMYVTAQLRVLNLHLRAMDCRPQLTQSEKITRCDKGVVAEKSGDANRLPSADPPDAYAQLVHNVQHHQVIIRTVKILEDAMGPCVFVQFLFNVIVICIYAFVLAGVHSMNIGSLILCLTNLISTCVENFGYCWFGNEIMSQSEQLAFSAYSSGWVGGGGRFQKALVVLTMRAQRPLCLTVGSLYTLSRETFLALLNGSYSFLAVLLQMNNSN

>LmigOR91

GGSALAVHAYYCTVLLANTAKTLVFAACRHSLGRAIHVLSRCPRAERSEKSGASARLTFALPQVMVLLAVATHSLVPLLGAGDGACADPEATGQGGGGCFSGRFPLELWYPAAALATPLYQVVYALQLVAIYCTCHTAINVDLFFFAVTNHASSHLQELNDALCRMGVRHTPVNRRRGSDESDHSADGKHGNGPLRHTSEESLVRAEHQRARYQELVRLIRVHQTITRAIKELEPVISYALFGPILTNVLNICLHMLVLTTERDNMGTNSKAFVGILFNLIQNGLYCSFGETLTHQSDRLFISIYSSGWENGCRRFKKAAAILMFQTRKPVQIKVAKLYTLSRRTFLQLLNNSYGLFNLLYQVKNPE

>LmigOR92

VQRPAAGMRVTAVHKQTALASEAGRDLMGPGEAVLRLLRVWPPPGPEARGLLHRLPYPLLALQAGMAGVLASTAAHLHQGGAGGDEESEAQQTTVALFIVGTIIGMLVKIWSFMGQRGRMQVLLSLLLEMRRRYLRDHTGARPRAQDHGVTLFYILQGNAVLAALAWSVQPLLSGSARRLPLPAWLPFDATVSPYYEAVYAGQALSLLLVPQISLCINICYFALMLHLAAELAVLCDNVAAVGWRRTSKEPAPQSQQQVLDREFAASSENRLLEDNVRHHQLIIRAVSELQQIMSTSVYIHLFVNMINVCSHIFVISVVLLETGEMAVVVSQACSLAVFLSGIALYCVIGHTIIDQSERLPEAVYSSGWTGADLSFRRSVSILLVRASHPLSITVGKMRVLSKPTFVQVLNGSYTLFNFLYRTQSDKEQRERLGLQDS

>LmigOR93

MRLRAALLAAAHLVLPVTLSVNFLSCRFDSLDLLATNIFLLSGMAGVSSKALLFVLDRERFELLLLRLQRTRMRFPDNSGARERRRRMATRVYYAQHGSAQLVVLLWVSVPGVTALVSGEGRELPMPLCPPATAADVHLSPCFELIYALQAACLFVAVEGMISLDSSYLTLMLNIATELEVLNDNLVSIRSGRLPEDKKCSQLSDQSPSNPAHEDDMYFQLVENIKHHQHIISRAQELESVMSGPTFVHLFYSLVCISLSIVSVTVLLQTEGYTPKILKITFVIVVFVSQLGFFCILGNNVIEQSERLLVSAYSSYWPGAQPRFQRALLVLMLRARHPLHISVAKLYPLSKETYLQILNASYTLFNLVFQTNGRN

>LmigOR94

MEALWWPVSPVRRGLRLLGLWVAPPGRRALHRLALSWVLASHAFLLLVGAASLVMDTPEDLPQLSFTAYTTLTCFGLIAKLVSFSLDGARLTRLLQLLAECRARFPDPGGRRGQHHLMAVRLHRFLQVSYRVNSVVWMFAPVVSAALAARSGGEEPVKRMYVLPLWLPVDTQASPAYEAVYVAQLATGWMLSETTVLLDVALLALMLHAAAELAVLNDRLRSQPAAAAAGPALASPVAYPKDGDTDPKHDHMYRHMVENIQHHQIIIVYTGLLQSVVRRAISVLLACNTVSICFHIIATVALLQKDIELVGMTKMVVGSTLYAYQTAILCLLGQRITTQSERLSASAYSSAWWEGDGRYQKLVVVFCERASGALSIRVCGLYSLSKETLLQVLKAAYSLFNFMYQAMETSNH

>LmigOR95

MQQADPLWPLCSVVGELGLLGLWLPPPGRRLRHRLVLAFVVASHLGCFFGALASITMDTPSDLPQLSFIAYNCLTDAGLTCKMLSFSLDGCRLTELLRLLSESRRRFPDRAGHRASQHATAVRIHRFLQMMYRINTAYWLLGPVVRNIVAAVSRQPSISVRDIPVPLWLPFDARRSPVYEALYGLVLAFGWAISETSVLVDSSLIALLLQVVAELAVLNDNLASGTPPAGKLTTRVTADAAGSRVQIPLTTAPSVLEGAVCDLVHRGEVYQHILDNIQHHQTIISCVRLLQKVLSRATCVLLFCNTISICFQVIATAVLLQEDGETMQTLKILMGSTLYGYQVALFCLLGQRVINQSERLIRSAFSGDWPEGDVRSWRLVHMLCMSTRQSLSLRICGIYTLSRMTLLQILNVSYSLLNFIYQTKTEQSSGKQEL

>LmigOR96

PGRRLRHRLVLAFVVASHLGCFFGALASITMDTPSDLPQLSFIAYNCLTDAGLTCKMLSFSLDGRRLTELLRLLSESRRRFPDRAGHRARQHATAVRMHRFLQVTYRINTAYWLLAPVVRNIVAVISNKPYSRDIPIPIWLPFDIRSSPAFEILYSLELAFGWAISETTVLVDGSLIAMILQLAAELAVLNDRLAAGTTPAVKQTPSDAATTEEGTLKDSRVRMPTKSSSAFVETTCTDFLQSRSSIGSEDEMYQQMVGYIQHHQTIISCVRLLQKILSRATSVLLFCNTISICFQVIATAVLLQEDGEIVQTLKMLMGSTLYAYQVALFCLLGQRIINQSERLIRSAFCGDWPDGDVRSWRLVYMLCMSTKKSLSLRICGIYTLSRMTLLQILNVSYSLLNFIYQTKTEQSSGKQEL

>LmigOR97

MEEEEASELLGATAVALQLMGLWRGGGGGVAAQAAVAAPTLLVMGSAVLLSGAKLCAEPPAVYEELIAVIFILVASVSWTFKATAFVGQRRRLQALAALLVAGSQNYGDGSGTRAHYRALARRVFIYTQAITAVPIAMWALEPLLSGGQNTPLPAWLPLDLHATPAYELLCTFQAVAVTLSVEASVCLDMFFIVLMIAVAAELHILNDNLESIRLQPVHSLPLKPVDDSAMQSYRNASSLTEKDIPQQYEFYRDSHKSMNAIHGTADAHEVMYCLLVKNIQHHQLILKCIKELETAMTYSIFVLLFLNMVTICTLIISTTVLLQSDSDPTSLYKMVSSLPIVMFQTGLFCIFGQMIIDQSERLPAAAFSSGWLDGDIRLRRALLLLMRRAASPLCIIVGRMYPLSRHTYLQLLNGSYTIFNMMYQVRGRSD

>LmigOR98

ATSRTCLVAGCYLALVAFAGSQLLAEPWRPLEELAIAALIFVTSASFTFKFAMFLRYRPRLLKLATLLCGGGLRPAEGEETRRRYRERGRQVFLYLQAILSVPTLLWAMQPLLVPPGGSAANLRNATAPVRMRSTPLPMWLPPALQQSPAYELVYALQVVSMLIVLQTSVFTGVFFLVLMLSIAAELHVLNDSISGNRSKPSSSSGRRCSSLSMEIHCPSNGRLVNDSSVINFEGRLFRQQMYCDLVKNIRHHQLIIMCVKELEAIMNLPIFVLLFLHMVNICAQIFVTSLLLQKDNDSTTMFKVLFTLPIYLYETGLYCVFGQIIIDQSDRLPTSAYSGAWLQGDARFRRALLLLTSRASRPLTLTVGKTYTLSRHTFLQILNGSYSLFNMLYQVQGNK

>LmigOR99

MNAYVCAMGALLSVKVAAFLWHRERLWQLACQLVSCWRQFEDADGGVRDMYRSQAARVVRYMQVMAAIPAMMWILEPLFSGGDEQSQGRSLPLPTWLPLTLQQSPTYEILYVLQVLIIIIAVAVSVYVNIFFAVLMLSIAAELHVLNNNMMAMGQCRDCEVPVRYEREEGRSRDQVTSSFLRTHRRQRVPKAAANKSLPSPVVVHMHKGSYDRMYHQLVKNIRHHQVILRSVEELQKAMTHSIFVLLFLNIFNICVVIFAGTTLLQKQADQVAMYKMLCSIPIYMYETGFFCVVGQTIIDQGERLSMSAFASTWLDGPRRLHRLLLVFMLRCARPPTITVGKTYTLSKRTFVRILNGSYTMFNMLYQFQRNK

>LmigOR101

PGSTSAAGLCRERVDDLEEMSEGIFICTTVIVSTARMLYFLAYRMRLQRLASLLLEARRCFPVQGAALRSRYQRHAANVCIGFQATAVLPVSLWVLDPLLTAAVTSQNTNNASADAAAEASRLPLSLWLPVDGRQSPSYEAVYAFEGFLVVFTAQVLLFLDMLFIVLIIHITAELNVLNDSVAAIREAVAGGGETGNGTDASGKLDNTYGSVSRSDSDMYGQLVEAIRHHQTVMRYVQELEDFMSQPLYILLFTNMMNMCLHMFTFVVLLQKDIERSSMVKMMLTFPAYLYQTGTYCIFGQTIIDQLLKDTYTMFNMLYTLQGNK

>LmigOR102

MEDAAGQSATLLSPCSVALAWLGIWRPPGGRRSGLGLPGAVFIAALDITISSLALVQMLIDRPEDPADFREVFFICSCGVSWSVKVVAFLLQGDRLERMVLSLLDAKTRFPDNGSRVREKYTAMAYTVWRMWQAMPAVTVLLWMADPLLQTIISPPADNATRPLIFWLPVQVHDSPAYEVTYAVEAFFIGTVSETSILMDIFLIILLVYAAGEIAVLNENVARMGLTMQRETAKAQPVESSSEKASMSKTRYVPSGDSPAADVGAGGKGQLPLLDGDDALWDMYSALVTNIRHHQAIIAYINDLEVVLSTSIYILLLTNALNVCLHSFGLVAFVIIVFLQLLAEGATSSTVFKEVISFASFLAQTALFCFFGQLIIDQADRLQFSAFCCDWPDADESFRRSLRIFMARATCPIKVTVGKLVELSRNTFLQALNASYTIFNMLFNLQTSDE

>LmigOR103

MEALEKGEPTNGELHLINGTFTGPQMAGRSCSSPASYSKYFHDAPVSGHLGFVKTLDWKGFYRSVNHYEASAANGAESAGRQEAVPRQRSRHPGKIRQDCLHRVETVDGESPRTGVTVTVVLWMGDPLLQMLTSPAGNSSRPLIFWVPLEVRHSPAYEITYAVQALGIAAIGQTSILMDIFFVVLLLQAASEIAVLNENIAGMGFKKLRDRENESEEHTSIQRVESSTYVVRTSYVLSECTPHPEISSDNNGNPFAFDRGHKYCKMYSTLVRNIRHHQHIIAYVKDLEVVMSTSLYLLLLANALNVCLHSFGFVALFQEGATRSTVIKEVLSFPSFLGQTALYCFFGQVVIDQADRLHYSAFSCDWPHADEPFRRSLRIFMMQAARPLNVKVGKLVTLSRKTFLQALNTSYTIFNMLFNVERRS

>LmigOR104

MLHVYLGTCFTASPLRQWVSAPACRQVATAAQEVRRLVGPAAPALEWLGLWHPPGSPPTGFKALRGATVLLIDLLLFVFCSLLLLLDPPADAEGQRETFFYIMASFTWTVRGVFFMLERTRHEKLVSILLSLRQRFPDDGCDIRGTHLKNAALLSLAWQVAPVLVLPAWIIEPMLETHYVTYGNITEVYRRTMLYMWTPGDMQQSPNYEISYVSQVVVSLIAVEASVLQDIFFVNIMVQVTSELDVLNANISSMRLPAAAKSQSVANGPEYSDTKLEQYEATYFSHKSKTSAEDELQRSVTIYKNTNDENEELYKKLVKNVRHHQMIMVCIDELEAAMSKSIAVVLVISTLNICVHAFGFVGMFQEDAPRVTVFKRTVAFVIYMTHNALFCFLGQSITDQSERLLHSTFSCGWADADRPFKRSLMIVMRQTSRPLVINVGKFFTLSRNTYMQIVNTSYTIFNMLLSVQ

>LmigOR105

MKNQMHSDEKVWDTTKDVEAMLGPSAVVMSAIGLWQPPGGRPAGAKVLVTSAVLLMMLTIFLGGLVQLVLEPLPLEEILDAVFTCACSFTWGIRVMVIRLRQRRVQQLVVDVLNMRKRFTENAAALRKKYHRRGLMVCLAWVVFPALAVPMWFVEPAMTKKLVTTSENTTMVIRKTPFIMWMPMETQTHPNYEITYVAHLSLLVCSVSPTLIIDLFFACLMTAITADIDILNNNIANMRLYKEDGFTENSEKGVEVTATWETTYQSKKSLAEKSERVDDNTQVLATYSTDPYEQLHRTLAKNIQHHQTLMSIVGDLESIMSESSVLMLIVNSINICLQALGFVDAFRPGAKRSTVLKKVLTFPAYINQTAHFCWLGQAIIDQSERLQDSAFSCGWADADQRFCSSLRIFMLQASRPLKLQIGKIFTLSRNLFLQILNTSYTIFNMMINF

>LmigOR106

AGLGSQLACLTAAKAMKDHVQSDGQVRDTTQEVEELLGFSAVLMRWMGLWQPPGGAPAGPKLLIALSFDAMFFVIVAGGFASVVLDPPPMDSVLEVVLTLASSVTWGVRNVAILVRQNRLQNLILDVLDMKKRFAENGTEFRKAFQRRAKIRTALLVGIPMLGIPMWLVEPAFSKTIVATSENTTVVVRKTPLVMWMPMDTQTHPNYEITYAFQMLLISIVVDANVIIDIFFSCLLITVTADIAVLNNNVANMRLCKDDGFTKESGKEVNLSATWETTYKSKKSLEEKDQLYDGNTDVYASHTTDPSTQLYRTLVKNIQHHQVLMSIVNDLESIMSESSVLMLVVNSINICTQGIGFVDGFRPGSNKTTLMKRFLTFPAYVNQTAHFCWYGQDIIDQSERLLESAFNCDWANADSRFCSTLRIFMLQTSRPLKMQIGKIFTLSRNMFLQILNTSYTIFNMFINF

>LmigOR107

MESEAKSLVGPSGWALRQLGLWRPPGAAPAGPRLLAAFFVVATDALVSTSSAVQLAVDTPTNPETLRDVFFQTTCSGAWAIRTVLFMQQRDRLQRLVMTLLDTRKRYAENVPGTRSSYDRGAAIVFFAWQMLPLTAISLWALEPATVPAEPVLVGNTTVVLRREPLVLWLPIDTQRSPTYEVVFVMQVIGIATVSEVSVLLDIFFVSLMIHVTAEVEVLSGNVLNIHLSTLDGQLTQSREYGDGRLSYKGGGQLAAIDEPTDLSAAGSGYLPNKTLFYSQDMDDAQRRLYACLKTNIQHHQTIIHCVNELEEAMSNSTYLILLVNALTICLHAFGFVELFQGGGKGPAVVKRLLACPIYMGQTALFCVIGQSLIDHSERLLDSAFSCGWPSADRRFCSALLIFMRQASQPLKIRVGKIVTLSRNSFLQIMNVSYTIFNMLLNTQ

>LmigOR108

MACTFSLPTGCTTRGLTAAPCGADGVGGAFAGGPQRVGAAAAGAVAALVSAASVVQLAVDTPTDPETLRDVLFTSTCGLAWASRMVLFMKQGVRLQRLVTTLLVTRKRYAEEFPGIRNSYDRAAAVIFYAWQVLPLTAVSLWALGPVTGAPLAVASGNYTVLERREPLVMWLPVDTQRSPTYELVFAMEMVGVYAVAEISILLDIFLVCLMILVTAEVAVLNRNVSSTRLSRLDQDQPGGVTAIGREGYGGLSADGEWSAAANQTLPNSTLLPYQDTDAAKRRLYDCLKRNIQHHQTIMTRREQRTAFYYLGSLPASHKSVNLFHNLVSSLAGIRFLKYAAIKLVLRPELQMLVFGVLVTIIKTALFCLIGQSLTDNSERLLDSAFSCGWPTADRRFCSALLIFMQQASQPLSIRVGKIVTLSRNSFLQVMNVSYTIFNMLLNTQ

>LmigOR109

VNIAANISLVITSGVELCADKPRQTERASVTAFLFSVSIINFVKAVSLLRHRSRLRRLVRRLVAVRAAFADPAGTRGRYARHAALLASTWLVTAETNVAFWCLDPLISEAAGGAAAERQLPLPLWLPFNQSRPHSYGRLFALEAAVLMSAVQIAILVDALFVTLIINVTAEIHVLNSNIRSMSKAAASGGGLQSGGTIENTTTSDESTLVANLHRSNVRSSNSAISDISASGRNGKSADDEMYGLLVKNIQHHQLIIICVKELEKAVSTGTFALLSINILNLCSHIFSLVVMLEGENSVSAITKMLVAVPVFMCQSGLYCLTGQAIIDESARLSTSAFSCGWPDADQRFKRSLRLFMTRAAQPLHIRVGTLISLSRATFQELLKGSYQLFNVVYQVHTN

>LmigOR110

MLPSADDEKRLLGLMRFLLGRSVASRDGSFRTQLANGATTATLLSTAAANLIIMVCSGLKLYLDPPEETEKASQVAFLMTVSVANAMKGFSMVQQRARLQQVVAGLLAMRRAVCDGSGARHRYACSATLIGNIWMVMSVILGIVWGVDPVFNQPPQLNGTSPDPVLPLPIWLPLDASVPLTYWLMFALEAVVCGWTIFFVMIVDLLYVTLILNFAAELHVLNHNIQITCNAVDVTAHPKRKIGVSRHAGVSLYKGHNDDTAAIPNFSLAHFTAANPLMPAYIPDHFRVELSEDYDTYRLLVKSIQHHQLIVKCVNEFGKATGLPVLMVVSINVVNLCSNIISLAVLVEEDPHASAIAKSLIFTIALGSQTALYCLPGQMIIDQSERLAHSAFCCRWPDAGVRFKRSLLVFMACAGRPLRLRVGKLVTLSRETFQELLKLSYQLFNLVYQLQSS

>LmigOR111

LASRLFSSDDVVEETSSPLKTPLPTWTPAAAQRSPAYEALYLFEALCLTASSQALLCIDVFFIDLMLLVAAELRVLNDNVAAVSAGAARSDSREDTAGHGSGVSTVQQRSREFPDTFSAFDVLVDRRMSEDMYRQLVGNIRHHQMIIECVELLQMTMTYSIFALLFFNMTSICLNIFVTASLLQSDADLVTAMKAVFTTPVFLYESAMYCIFGQMIIDQSEQLPLSAFNCGWPETHTRLQRALLVFMLRSSQPLRIQVGKTYELSKETFVRVLNGSYALFNMLYTFQGNK

>LmigOR112

MGDGCEALGASVSVLRLLGLWVPTESSGAGGKAAYVPGALSCVAIGLLSLSCASKLFIDTPTELTELTVCAYLFVIITANFVKAFCLLLQQGTLHELVTLLVEAKKKNVIDVQHNEDIRSLYGVMSARLYRYLQVMIVVSSVAWLFVTVVFRVITAGSTNIEWPTPLPIWLPLDTQRSPAYELVYVAQVSCAVVTAATMLGADTLFFHLTLMIVAELQVLNDNVSVLGRPAPPSADTRQVVCTMNGAGERQTHPPGGVAERQSDSDGAVSLSDHQGTAAAESKYLVLIEIIQHHQIIIKMVSLLQTIMDYSVSVQLLTNVLDMCFLIFTMSELLHHEKSLHAVLQTILSLPCLLCESGAFCMFGQMIIDQSENLVHAAFSCEWLEADGRFRKPLYTFMLMATHPLQIKLGGTAKLSRSTFLQALNGSYSLINLLYHSRRPVG

>LmigOR113

MDITEEELEAVVPCMARSGLLGYWRSSASREGAGSYLRGFLSCCLITCISLSAAERLLTDTPSDLAELTMTAFELTVPLTVVSKGLFFILQRDTIHELVDLLVDMRRRYAERDDGPNRRRACYLYVLAVQRVLLVMALLIIGGWLAGPMLPHVFSFASQNESSVPWQTPLPLWLPVDLQRSPLYEALYLFQGLCVLTSLTSASALDACFCNMMLMIAAELQVLNDNISSPSGNETVVDKGESESITLEVHSELESVVPQFKSGATGDAGLSKTGRSRPRNQTSLRL

>LmigOR114

ATSAGKLCLDTPQELAAVADYGYAVFHLSAVTVKVACFILQRSTIEELVNLLDETRKTYGKTEANYQVRQLYQRRATNIYRVLQALAVAVLCMWISSPVIQRQGLKEGERPPPNPIWMPDNSPGYEIVYSVQSLCGSAAVQASMLIDTSFYKLTLMVTAELQILNDNLARLGRAAEAADRKGTAAKQDGKEVAVPAIKQTSAPVTEDNDQLLNDQMVDNVRHHQAIIKCFDLLQSVITYSVSIVLLTNILTVCFSIFVVYVLLQSDGGLKSASKTIIGIPSVLGETGMFCIFGQMVINQSERLRSSAYSCGWPDADGRFKRALLILVLRTSQPLQFTVGKLIHLSNETFLQILNCSYTLINVLYQFQGSKE

>LmigOR115

PLLQHKAADEFSGAEKVERQMPIPSWFPFDVQRSPAYEVVYAVQAVCGTAAVQLSMLLDASFYQLALLLTAELRVLNDNLALVGAATSATGGTGVAGRDSHHQHMPAPKLTTSAVTEDNDRSRTLLYFEFVENVRHHQAIMKCFQLLESVLNYSISILLLTNILTMCFSIFFASVMLQADGGLRRAMKITSSIPNLLIETGMFCIFGQMVVDQSERLPQSAYSCSWVDSDARFKRALLIFVLRTSQPLEFTVGKLIKLSRETFLKILNSSYTLISLLYQFQESND

>LmigOR116

EPADDLLDLTWTVFILFGGAIVFVKMVCFVHQRAVMQEAFQLLLACRNCHYGGDSIRTIRGSYQRLGNITYFSIQVMVIVAWMIIVFMPLLNHRISAAGREPTAKEHGPLPIWLPFEVHNSPFYEFTYAFQALWLAFVAETSICVDCIFVNLMLMITAEIHILNSTLSTLQEHSIINKVPVVTTNQSTEDILPFTHSSRIHDSKQLFEKLISIEGTDYSPSDMCDISCAESQKIIKQQIYHQLLQNVQHHQTVIKCTSSAQKAMNFSVFVLLSTNIIEICSSIFGTVELLKNDMPAAAMKTLCVIPIILSQSGMYCFFGQMISDESEKLLQSAYNCDWYEGDIHFQRVLFILMLRATGPLKLKVGKTMSLSRQTFLQVLNGAYALLNMAYHVSK

>LmigOR117

GAGGDSPRLLPLPAWLPLDLQASPTYELVYAAQVLLIPLSTTSVCSDFVFIGLMLRISAELEILNDSISGLHKIRKDVPTAPKNEAKSIGRVSDGEINLHISRNVKHHQAILKSVALLEEAMSTAIFILFLGTMIAISINIFAATAVLQTVGGMTKALKMITAIPPIMFEVGLYCIFGQIVTDQSEKLMHSAYSCGWVDCDPRFRHSLLTFCVGSRRPLEITVGTVYKLSKETFLQVLNASYAMFNMLYGFQSNT

>LmigOR118

MRQTRSVESVLGSSAALLRLLGLWSPRIEDFSRTHRVLRGGLMLALSFGLMVTTLLKLVMDCPRELEELSACIFSATMLCEVFFKMVFFVLKVPTLHKLVQLLSEIRTEDSIGERNDEIRRRYQIVVDKMFLFLMATAVVTETMWAAIPLMHQLLNMDGEVTRLLPLPLWLPLDVYASPTYEVIYGAQVLLMPLTTTSLFFDFVFIDLMMRIAAELEILNYNISSVHENRKTVSTMSKDIHKLCQTVSDNKTNVQLVKNVRHHQAILRAVVLLEEAMNTGVFILFLATTIAVSSNIFTATALLQAHDGRIKALKMLSAMPPVLFEVGLYCVFGQIVINQSEKLMHSAYSCEWVDCDTRFRRSLHMFCVGAIRPLEFTVGRMYKLSRETLLQVLHGSYVMFNMLYTIQNRK

>LmigOR119

MVVFGRGVEAVLGPSAKLLRLLGLWSPQKGDTSHNGSALTGYLTLALIFGLMVTSALKLLMDRPRELDELGACIFIVTMLAEVFFKMLCFVVQRPTLHKLVQLLTEIRADGSTGERNDEIRRGYQILVDRMFLLIMVTATATQTLWAAAPVIYQPLNEDGEVTRLLPLSMWLPLDMNASPNYEVIYLVQVLLMPLASASLLFDFVFIDLMVRIAAELEILDYSFSGLSKNPKSVSATSKNEFKSVHTVSDGEINLQLAKNVKHHQEILRSVDLLEEAMNTGVFIQFLASTIAISCNIFAATSVTLYDSQFKQLIIILLIQSGLYCIFGQVVTDQSEKLMHSAYSCEWVDCDTRFRRSLLTFSVGATRPIEFTVGRMYKLSRETFLQVLQGSYAMFNMLYTFQSNR

>LmigOR120

MLRLLGLYQQKGGGGRVKPKLLSALSLIFLLYHPVFAAMKLYMEPPEDLVEFALCSFSFIISDGVFVKTAIFIADRGMLHQMLQVLSDSRRLYGGEETSKKIRNRYENLAERVLLYMQVSTMLASVGWLAAPLVFRALAMASGDGGEVPRKLPLPVWLPVDVQETPTYEILYVIEAYCVTLTGLVTLCIDVLFIRLMLMVTAELEVLNYNVATMAKRREKISDERRSLEYQQGTESQDFYRGDKALPMASESYEDAMDNELYQQLVTNVRHHLIILRTVDLLEAAMSKSIIILLFINMGALCSNLLVVGVLLQAGEGVTRPLTLTAMIPFLLYQTGMFCVFGQMVTDQSEMLTTSAFSCGWNESDARFRRSLLIFMAMVNRPLEITVGKTCKLSREMFLQVLNGSYTLFNMFYQVHSTS

>LmigOR121

RGRLARLLGGVVRAATMFGLVFMWLGTVLKLCVDPPPQLEQLTLCSLVSSICTGFIIKAAFFLAFGGTLRQTVRLLADTRARFCTGDHNEATRRRYHKQSNNIYYFIQIVAAIAIVGWILCPLVTHILAKTDEDHPEARMQLPVPVWLPGDIHETPVFEMLYAFQSFTITFGAQFCLSIDIFFIHMMLMLAAELEVLNYNLSAMGHVNLPKLGSHGGKSISRYKSSGRQSALLSSGQQLGEQILTEDSGNEWLHQQLVKNVLHHKAILRSVSLLESSMTVSIFGLLFINMANLCSSMFVASKLLQKEGSIGKALNALLTVPSELYETCIYCIYGNVMTDQSERLLESAFHSDWVNGDTRFKRSLIIFMAVTRRPIVITVGKTCKLSKETLLQVLNGTYALLNMLFNIH

>LmigOR122

MPIWSPVDIYSSPTYEFIYLLQSFASLITSQCCLSIDIFFVHMMLMVAAELDVLNYNLSAMKHYDSQTPISDGEEFISNVKTSGRRLELPSSDKSFGEQALKGDIEGNGLHQLLLKNVLHHQAILRSVSLLQSAMNVSIFVLLFINMANLCSSLFVAAVLLQRDGNAAKALHALLCVPALTYETTIYCTYAHIMTDQSERLMYSAFSCGWVNSDARFKRSLVIFMMVTVRPIEITVGKMCTLSKQMLLQVLNGTYALLNMLYHFH

>LmigOR123

MGHSSEERESLEGPGVVLRRLLGLWRPRGRLARGLDMLLAGVTLVAISFLVVCVALKLYADPPEELEQIALCGLVASLCIGFFFKALLFMVLGGTLRQTVRLLEDTRLEFFSGDKNETTRRRYQQLSRNIYNYGQMVAVPAAIGWITCPLLSRLLTHTGDDQHEVKRQFPVPVWFPVDVYTSPTFELLYAVQSFCVLVVAECCIATDIFFVHTMLMVAAELEVLNSNLCAMGDAKLQMKRVKEEEAVSRYKTKGRRWAFLNSDQPVGEHGLPENAVHEWLHEQLVKNVRHHQAILRSVSLLQSTMDVSIFILLFVNMANLCASLFVAGVLVHKEGNVGRALNALVSIPALLYETTMYCIFGHVMTDHSERLMYSAFSSGWINSDARFKRSMLIFMMVTMQPMNITVGKTYTLSKQMLLQVLNGTYGLLNMLYHMHGSE

>LmigOR124

MGLWQPRGRAAQRVNALLASLTLGSLCFMALCVTLKLCADTPQEIEQLTLCTLVASICVGFICKTALFVIQGDTLQQTVRLLEDTSEQFCTGDHNRLTRRRYLRLSNNVYYYCQMVAVPAAILTNTDDEEQQLPWQLPLPAWFPGDIYETPHFQILYVVHSFCVLVAVQSCLSIDIFFVHMMLMVAAELDVLNCNLAAMDHITVQTTRNEEERFIPRYKRNGRRLPLLNSGQSLAEQTLSQDTAHKDLNQQLLNNVLHHQAILRSVSLLQSAMNVSTFTLLFVNMANLCSSLFVAGVLLQKEGNVGKALNALFSIPALLYETIIYCIYGHIMTDQSERLVYSAFSSGWVNSDPGFKRSMLIFMMVTVRPMAITVGKTCRLSKQMLLQVLNGTYALLNMLYHVHRSE

>LmigOR125

WRPRGGRAARLLNGLLTAFILASHAFLPVCVALKLYVDPPEELEQITLCSLVTSICMGFLFKAALFVAQGETLRQTVRLLADIRAQFGDRQQNHSTRRRYRRLSDSVYRRYQMVAVPAVIGWVLCPMLSRSVRGSDQAPQVAQRQLPVPVWFPVDVYASPTYEFLYVAHSFCALVAAESSVCVDIFFIHMMLMVAAELDVLNDNLTVMEDVNLYATPNERRGLTSVNGSTGRQSAIHDSSQSTLGENAAREGIHEQLSKNVQHHQAILRSVSLLQSAMNVSIFSLLFFNMANLCSSLFVAAVLVQRDGNVGKALNALTSIPALLYETRMYCIYGHIMTEQRARC

>LmigOR126

MEFESMMGPGLPLMRLTGLWQMGRQGGGVSRGLRLATIVLSVLLVVAGSTLHLVFDTPDQFEDITLCGFNIDIVSLDLLKGVLFVVQGAPLRELVQLLCDARAGFTFADINHAIRGRYEAVADRMRILLQATVVLPLVGWLSAPLMSRLAAGAGGSRAPRQLPVPAWLPVDIHATPTYELLYALQAFGCTAAGAFSICVDAFFIRLMLLISAEIEVLCENISAIGVPHPAQGSGGCICRCQPNAADLACTCKGCVKAFTSSPEEASDEMYQLLVKAVRHHQTIIRMVALLQQTMDALVFIVLFANMANLCCSLFATAILLQRGGSLTKTLKGLSAVPVVLYQTSLYCLFGHIVTDQSEKLYNAAISCGWVNCDARFKRSLLIFMVEAMKPLEITVGKFCKLSRQMLLQVFHSSYALMNLLYYYHYNTE

>LmigOR127

MGERGEAPAGLLGPEAAVLRLLGLWRPRERQGQGLTPPAVVAAATIAAVAFIPAGVVLRLCGDFPEEIEETAHCSYICIVCFGCIVKAVLFVMEGDTVRELVHLLQATRAEYGSDEGSDRIRSGYQGTVDRMYRYFQVMALLPTLYWICWPLVAAAVSPGEASGVSGARQLPLPFWLPSGASGTPTYHLLYAVQALSLSLTVASAVCLDVFFIRLMMMLAAELQVLNENIAAIDGCRASGSAYGREEEEFDSLVPSDDRALEPTKKSAANFSDDDLFSRLLNNILHHQAILRCIWLLQTAMNVSIFILLFINMANLCFNMFVTAGLLQDGRNVTKAVTAFSPVPGLLLQTAMYCLFGQITTDQSEKLLHSAFGCGWDDCDTRFKHNLLIFMLMVGRPVEITVGKTYKLSKEMLLQVLNGTYVLLNMLFHVHSDDHI

>LmigOR128

PGAVADWQFPVPHWVPVDMQRSPTYHLLYVLQSFCLLVASQSTIAVDLFFIHMMLMLAAEIEVLSENVSAMGKIDSGLMALENEDCGLSTKDLLSYRDGNGLVSEIFQKEYISEDQMRALLVKNVQHHQTILQAVGLLQDAMDISIVILLFTNMADLCSCMFASAILLQRGGNAAKALKPLMTIPPCFYETAIYCFFGNIVTEKSEQLVTAAWSCGWPLCGSGFRRGLLLFLTEAARPVEITVGKTFKLSKQMLLQVLNGTYALLNMLYHVHRSE

>LmigOR129

MSMPDSELRTLLGSGASIRQLMGLWWPRGRRGRGRACSAAAAAVSLASLAWLPTFSGLKLLIDPPPEIEEIAMCYLLIFACTGFFSKAAFLIYKGETVWKLLDLLSETRRLHRNGESNDNIRLSYQQQSRRVYLYMQGAICVAFVFWVSTPLLVRAFLASDEDSPESYRLFPVPLWFPGNMYLSPTYEILYSVQSFSVLVAAQSTVCVDIFFFHLMLMISAEVQVLNENIALMEKVNLKSEKQEDHELRLNIKEKAEDLSFRSVGYSTGKALTEEVSDENMCVQLVKNIQHHQLILRSVVLLQDIMNLSVFILLFVNMVDLCSCIFVGAVLLQRDGNVTKALKPLSTVPPLLYETGMYCIFGQILSDQSEKLTDSAISCGWVDCNDRFKRDFMFLLISAKKPLEITVGKTSKLSKQMLVQVLNGSYGLLNLLYHFQSIQ

>LmigOR130

MKPVGGGCGGLLGPGLTIRRLMGLWWPQGGRGRIAAAAAATLTVASLTMLVAFPALKLIMDTPSELEEITLCCFVIFLCSGFIIKSALFIYQGDILKELLQLFSDNRRIYSNDRNSEGIRQSYIKLSERVYVYMQVSVLPAVAGWVSAPMLARFFLTAESTQQFPVPLWFPGDIYQTPTYEILYAVQSFCVLVTGQCTVVIDVFFIHLMLMVAAELHVLNENISLMQKLNVKTRVSEAEEWQFRIRRNDEELTFPIHDHRARVGYFCSEDVSDENMCLKLVKNIQHHQQILRSVLLLKSVMNVSIFILLLLNMADLCSCMFITAVLLQRGGDVTKALKPLLTIPPLLYETGMYCFFGQILTDQSENLIDSAFSSGWVDCDSRFKRDLLIFLMAANRPLEVTVGKISKLSKQMLVQVLNGTYGLLNLLYHFHGSQ

>LmigOR131

MGRASAEVSNSRPLLGPGAAQLRAMGLWRPGRSLLHSLAAALMLACLAWVSATAALRLLIHPPAELEEVALCSFIATICSGFTIKHSERIIVAKVDRIPTPTTVVQVYMSTSTADDEDIEEMYEEIKEIQKVKGDENLIVMGDWNSVVGKGCVVPALIGWTFFPLVSRALNDSGEESPGAVADWQFPVPHWVPVDMQRSPTYHLLYVLQSFCLLVASQSTIAVDLFFIHMMLMVAAEIEVLSENVSAMGKIDSGLVALDDEDCGPSTKYLLSYRDGNGRISEISRKDDISEDQVRALLVKNVQHHQTILQIGEKIQFCRISSKRRHRLVGHVLRLEGIVNLFLKGSGVGRRYFPCLIDLTYQVEHFCSRLALLMISVLLQFYSRGFVSTQILQCSVKFLSHANSKERNVDHGDEGFVSHVVFHVRRALLLFLTEAVRPVEITVGKTFKLSKQMLLQVLNGSYALLNLLYSIR

>LmigOR132

PAAPRQLPVHVWLPADLNRSPTYEALFAAQSFSLMVLSQATVCMDIFFVHLMLLVAAELEVLNENLSAMERGRLQHGRSEYSETTDIHGEDSDRSTFTNTDRRLDVTVGTSQREHDERMYAELVKNVRHHQAVLRSVSLLQKAMDASIFILLFINMANLCGAVFVAAVLLQRDGNITKALKEVMLIPCVLYETGMYCLCGHMIISQSERLVTSAFRCGWPDCDRRFKSSLLIFMMAAIRPLEITVGKMCKLSKQMLLQVLNGSYALLNMLYHFHHTL

>LmigOR133

MDAVVGPLLPLMRLLGLWPCSGGSGRLPAAARCALTQLPVALMVAGSALKLCVDTPDQFEDVALCAFITNVVAAILVKAVMLVARGQRLRRLARLLADARARFPAHRSCTRGRYQALADRMERLFQVGGLVPLACWLSAPLVPQLTAAPGQGRGRPRQLPVPTWLPADLAASPTYQLVYTLQVLGCIGACASTVCADSLFVRLMLLIAAELQVLKENISSLRKTDSVRGGGYACRCRETVSFLASACKDCHDIVTPLSEKTTDEMHQLLVKIIRHHHMIMRMVSLLQEVMDVSIFILLFANMVNLCSSLFTAAILLQGGGSVVKVLKGLSPLPVVLYQTSLFCVFGHIITDKSGELTDAAVSCQWVDCDTRFKRSLLILMTVALKPLKITVGRVCTLSREMLLQVFHGSYALMNMFYYYHHKTK

>LmigOR134

ELQVLNDNLASVKAAPLLRSSPYSGWNRATNVTNESWFHSGVSSPTGAIACNSYQSSGDRSESTTSITGYTNRTAVEMYRALANSIKHHQAIIRCVEELESAMTYSIFVLLFLNMMNICVHIFVTSVLLQKEVERTTMSKMLCTLPIYMYETGLYCVFGQTIIDQHGCDVLQSEQLTASAFSGDWPEGDARMRKALLLLMLRASRPLQLTVGKMYVLSRHTFLQILNGSYTLFNMLYQVQKNK

>LmigOR135

AFMMPLWLPLDTQASPTYELLLGVQVPCCWICSETSVLLDCAMLALMLQAAAELAVLNDRLSAVGPGQRRAADDKRDLQANDHMFSGLVDNINHHQIIITYMHLLETLLSRGISVLLICNTISICFHIVATVALLQEDIEPVGMTKMVLGSTLYAYQTAILCLLGQRITTQSERLPVSAFSCDWPSADGRFRKLLMVFCLRSSQALIIRVCGLYSLSRETLLQVLKAAYTLFNFVYQTVGEEEPLN

>LmigOR136

RLPFAQHLWDDNGHWYGLSYAVQCVTGLWMAEVSFGVDCLFATVLMLAAAQLRVLALRLVRLKVDAGGAPGDQPGATDGAYRELCLCVESHQEILRFITHLNGTMSPVAMTQFVFSVLVACVALFQATYSTDITAVIKCVSFLPIPGGQVYLYCWAAHHVTEQAEAVSTAAYCSPWVDAGPRFKRALRILISRAQKPLVLTAGRLYPVNRETFLSLVNASYTYYALLGQMNKRSAN

>LmigOR137

SKAAALLDGAEELVCPNATFLRLMGLWRARGWTGAARGWFCWTLVLFVTATSAGKLCLDTPQELAAVADYGYAVFHLSAVTVKVACFILQRSTIEELVNLLDETRKTYGKTEANYQVRQIYQRRATNIYRVLQALAVAVICMWISSLVIQRQGLKEGERPPPNPIWMPDNSPGYEIVYSVQSLCGSAAVQASMLIDTSFYKLTLMVTAELQILNDNLATLGRAAEA

>LmigOR139

AEFEVLSDNFAALHKVHEQSEKGKIRGTVYERQIKEESPPASCAHIYIECVSDEQMYRQLTQNVRHHQALLRSISLLQTAMSAPIYLVLFVNMVNLCTNLFIATLLLQRDGSFSKALAVLLTIPTLLSQTAIYCLFGHALTEKSEKLTQSAFSSGWPECDVRFRRGLLMVMTLAEQPAEVTVGKMTKLSKQTLLQVLNGTYGLLNMFYQLHSQM

>LmigOR140

LLDISSPLDGLVGGRTRQALLLLDQAAVAFNHLCSVAAFTTMFVHFVVIACRHLQRSIDDLTADNCDIAAVVRHHQQILRFIREIEEAYCILMFWLFLPMMVVMCLIMFAFLTMTSLDIEFLEMLAFFLIYCVTNGVISICGSMLTSKAERVMVAAYSSAWPERSRGFSGAVRVVMVRFLQPAQLTVGKFVPLSINTFSKLLQESFSYLMVMLSLVNEKDSEAQPGVVVEATANHSAYH

>LmigOR141

RQSVTVAERLPLAVYAYIALLCLIWFPMPLLVEPHNPKLPFIQLHYWIDHTRFPVYEASYLMQVVSSFFFIFISTGMDCFFAVVMIHVTVQLKLLIYRITEIRLRDAPAVAAESEARWNRDVGDAHAEMYKELCLCIESHQKILGFVKYLESVMNPIALTQFIFSVLAACVTLFQETYNPDISAVFKCASYLPTPGAQVFLYCWGAHS

>LmigORco

MQKPHGLVADLWPLIRMVQYSGHWMLEYSGGKALRAIYSSAVSLLVVTQFALMAVNLIQRSGDVNELAANTITVLFFLHPVTKFGYFAVRSKAFYRTLATWNQSNSHPLFAESQARFHQLSVVRMRRLVMYVVSVTALSVVSWTSITFMGDSTREVTDPDNANETITEEVPRLMISTWYPFDASSGMGYMLAFVYQLYWLTATLMHSNLMDVMFCCWLIYACEQLVHLKEIMKPLMELSATLDTVVPHTSELFRAASTLPTNEPLYDAGNGAADGLTIRGIYSSQRDFSGFNRRSAALSTVREADAGGAVSSAGGIGPNGLSKRQEMLVRSAIKYWVERHKHVVRFVGNIGDAYGAALLLHMLTTTVTLTLLAYQATKIDSVDVYAASVLGYLFYTLGQVFLFCVFGNRLIEESSSVMEAAYSCHWYDGSEEAKTFVQIVCQQCQKSLMISGAKFFTVSLDLFASVLGAVVTYFMVLVQLK

Table S4 Amino acid sequences of 115 IRs of *Ceracris nigricornis* and other insect species used to construct phylogenetic tree.

>AlinIR8a

MRTCLWFHVVLFVAHEVAGQGVKLLVVKDNNAGIWDSVSTSFFEQLPVTVDKEDTNSTINDLCEVLKEGVWGVLDLTWSGLDEIKAVCNTWGLPYVRLEYGITQYLRGADKSLATIRKAPDAALIFQTEEQLDQSLFYLIRESSMRVILFKGLSDKEAETLTTMRPTPNFNIIFADTPSMNVMFLKAVERNLVRYDDRWILVFLDNEHNSFDRKTLVKRVTLATPTIDANAAANFAENVAETLEEVAKTSGIDLSPVPAQCEGSSTAAKDLTVFQEKLSEIVEKKPWLDWRQQESTMALHLDMDWTAESSKGEKLFIGSWNSKKGLTIAGNVTKIPRFFRVATGYMVPFAYPVIDPSTGAPKLDDKGNEVWEGYCIDLINRLAEDMDFDYELTTSYNFGRKLPNGSWDGLIGDLASGRVDIIVAALTMTSEREEVIDFVAPYFEQTGFSIVIRKPLRKTSLFKFMTVLRVEVWFSILAALCLTAFMIWFLDKYSPYSARNNKDKYPYPTREFTLRESFWFAVTSFTPQGGGEAPKSLSARTLVAAYWLFVVLMLATFTANLAAFLTVERMQSPVQSLKQLARQSRINYTVVQDSDAHSYFRNMKFAEETLYRVWKEITLNASANQSQYRVWDYPIKEQYGHILISMEKTGTVNSTEEGFQKVRENEDAEFALIHDALEIKYEVYRDCNLTEIGEPFAEQPYSIAVQQGSHLNEEISRRILDLQKDRYFESLSGKYWNSTMKGKCDSSDEDEGITLESLGGVFIATLFGLVLAMLTLGIEIVYERKAKKNVIKVKSAKPEKSEKSEKKEKMMNNPFFNDDKLFSREFGSFPKKPSKLLAPKPKVSFITVFPRDQLY

>AlinIR21a

MKLEVLFVTTLVHLTHSVKITKLLNSMAFADLETISCMAPDGIPYMVPLINSIAKRYLKDHATVILYDDYFYYHPRLKNMIDILISNYAYPLRHGLVNTTMAKPTVPAGILEARENEQMAFIVFTKESEIGAEAIREFTGHNTMTLLIAQTSVYHVKLFLQTKLAADITNLLVFVDPMIKIDHFVQKTARVLKECDILIFSHKVITDSLGISMPVIVTAWRRNHLTRQVQLFPPKYKRGLGGLHLVASASEIPPFVFRKHGHDSGAGYTITKWDGIEVHLLYMLSQMLNFTVEYKEPEFNEEEDVAQTVIKDLHTKKTTLAIGGVYLTPERIGGLTFSFPHTQDCASFISLASTALPKYRAIMGPFLWDVWLALTAVYLLAMFPIAFSVWHSIKPLLNDIREVENMFWYVFGTFTNCFTFTGKNSWSKADKTATKFFIGTYWIFTIIITACYTGSIVAFITLPTYPETIDSSKQLLEEDYKISLLGSGGWEGLFNDTEDPVASKLYESVERVPNLYSGLRNVTRNVHSWRQSAFLGSRRLLEYTVKTNFTPDEDSKRLMFHLSDECFVPLFVSIVMDKRTNYLEEFNNALERIIQSGFMTKIVREVEWQEYRSASGKLLTMHKGLKGAPEDRELNLDDTQGMFLLLGAGFGIGLLVLIIEISVWSSEQRKNRQFGELTLKQRAINKLKEHWETLYACLLAPANSGIIYFRERRVSSAFGEYVTQPYASWSITSIPSPTVEPPSPSPPNGEISSAPTVGNAASLSMDQLSFPPEKPIRMMSF

>AlinIR25a

MPSFTARLPGTTSVAKVFTVFMFYVSLLQKVHSQSATSINVMFVTEDRNDIARLAFDVVSDYVKRNSKLGIEMEVFRVTESGSDAKFLLENLCETFNASAKAGKPPHIILDTSVVGVTSEAVKTFSRALGIPTLSASYGQEGDLRQWRALEVEIAKYLLQINPPADIIPEVVRSIVILQNISSAGIVFDDSFVMDHKYKSLLLNVPARHIMGRVRNIQEIRNQLTRFKELDIVNFFILGSLSTIRNVLNEANGMKFFDRKYAWHAITQDKGQLKCDCSNATILHIKPEPDPGSKERLDNLRTSYNLVEEPEITSVFYFDFFLRGLLAAKALIEKAPWPKDYNKTSCDNYDENHDFIRKDLDLRSSLRDVKEAYSYAPFLISTNGKSFMEFNMKIEKVVIVNSIAESAEAIGTWKAGLSNQIQTKDIASMRNFSAVTVYRVVTVKQKPFVIETFENGKPKYSGYCIDLLEDIRSFVHFEYDIYVAPDNAYGNMDPSGNWNGMIKELIEKRAEIGLGALSVMAERENVVDFTVPYYDLVGITIMMKKQTTQTSLFKFLTVLENEVWLCILASYFFTSLLMWVFDRWSPYSYQNNREKYKNDEEKREFNLKECLWFCMTSLTPQGGGEAPKNLSGRLVAATWWLFGFIIIASYTANLAAFLTVSRLDTPIESLDDLAKQYKIRYAPINGSEAMTYFQRMADIEERFYEIWKDMSLNDSLSEVERAKLAVWDYPVSDKYTKMWQAMKEAGLPATLEEALDRVRKSQTTSEGFAFLGDATDIKYLVLSSCDFQIVGDEFSRKPYAIAVQQGSPLKDQFNNAILQLLNKRKLEKLKEKWWTENEDRMQCEKQEEQSDGISIHNIGGVFIVIFVGIGLACITLGLEYWWYKYKKPASPKQVGPMAQIISTNATNKQLSVTGLMDYNTREPRARYPIRRTAVNATQDYSRPTFPAQQERLSHW

>AlinIR41a.1

MPDMVKNLQNKTLKIMTFDYDPYTHFEPLDGTEIKLIQEFCKKHNCSLVAVDDGHFWGDIFENGTSDGLAGMVYDGRADFGAAAVYLWLPYFYFVDYSTSYLYSASTLLVPKPHPVSGWKTPFMPFDVFTWIGYGLSVIMAAVFMYVITYFTVKYTRFTEAVRKRRMFLDKLDCLFRALGLAVLQQPSTPLVPNTPIRHLFTSFEFLFLITSSIYAAELASYLTVPRYEKPIDTLIELADSGMIWIGEHESWTYSLRGMTDPEIVTIVNNYRIFSHEKLRQLAPTGEYGLIVERLPGGQYTEQEHVTDEVVAQSHMMAENLFGSPPVIAVRKGSPYRKYLNKVISNVLCGGFYLYWEREMSRKYLHSRRQLALREAEHPHYKDIPKRLTISHIQGGLYLYSLGITISLFVFILELFHFKAKGPKNRPKDK

>AlinIR41a.2

MLSSSSESLPKNCCYILTIIVSLCAADFRTKNEEDVFNAKMVYLARQVAQDYMSDRLRCIVVVSDEGLLEDFTGYNDTTVLRVLFNGSRDECDPTMHKYILQAFYHKCTRYIVQISKPTCFFPAWFLARNGSTYEKHNPRVLFLPVKPHATVYGEEVLAMNQTNISHDILIAETSPQTPIGAIAMPKEIDPNRPVTLYTNNFWQYVGEPGRIGRIYLDEWSWELGFKNGVDLYPDKVRDLRGKVLRLSAFPYLPYGNNEPMDGSEARILLEFCVVYNCTVVDVDDGHLWGEIYPENGTGVGEAGTIYMELSDFGVGANYLWLEFWPYLEFSNCYLYGALTVMVPRPELLSGLLTPFLPFPLSLWLVIVMCVVVSAVGLHWVTEATIKFAPHFLDEIYKNHKFITYTDSMIRSIGMLVLQQPQRLVTGSPVRHLFTAFEFTYLVITSAYAAELYDFLTIPRTTKPINSVFDLAESNLIWMTDHEVWVFGILHAEDPSIRKAASNFRALPTPELIKLGESNHPYGLGIERMAGGHYTELPYITDKFIEKSRVMRDNYYVSPLVVNMQKGSPYANRLNDIIGRMENGGLYYAWEADCVRKYLNYTKQLDMQWSTRPIKYPPKVLNVADLEGAFLLYFIGTALAVGLFFLEIYFKKGLKLKNSFLNPTPKWFDEYLLGQSGSHD

>AlinIR41a.3

MNNPLNLCTDGLIISMWLFVIGSSTTSQANVLFDNKHTILLGSLEKSVITQYFHKDKCIVLIVEDDTWTTNKELEHYYSLLSLILVSSIDICRDPYLVESIVSAIDSGCYSYILRVAEPKCVLKSWGESQLSYNVHTFQRVSPKMFATISNHQKDGSKIVDEFYSLPEAELSSNIVVAVFGNESLEWPVTIYTNNFYEPMSSADREPKVFLDRWNELNGFELQADLYADKILDLQGKELKVAVVDLLPYAELRRFIGQEAQILKHFCAHRNCTIKGITDEWFWGEIFENGSGNGLLGMVFDGRADFGIAGVYGWASVFRHTEFSASYLHSGVTLLVPKPVKVGGWLIPIFPFSSEMWLAYILSVIVAGISMHIITMATIKYTRFAEVVLKRGMFLTAVDTAFRALGLSVLQQPSTPLVPHTPIRHLFTAFEILYLVFCTVYAAELASYLTAPQYSKPIDSLEDLADSGMIWLGEHYGWVYSLLDVDTPSILKIVDHFKVVTFEEMDKLAGTGMYGLIVEQLAGGHFSERQYLSEKIIAQSHIMAEYLYDSPVITIMRKCSPYREHYNELIGRLLENGLLLFWEAEAARVYMSSWLQTALKSAIKVNMEDEARALTLSDCLGMFLLLAFGLFTSSVVFIVELWIIRQKNNTKD

>AlinIR41a.4

MVNVSLVGSTQRFRWILQLSDPAKFMENWEKARVDSLIRFKPRILFLPWNESNYATTLFEAPELNYIDDAVAVELEDADTNRNKQLKLVTNNFFVDIGSKNETTEIYLGVWPLNNSIEIFPNKIGDLQGKELRIATLQYLPYSQVSPELDGVELRILKSFCKQSNCSLVPVTDDFLWGELFENGTSNGIVGNVLQDKADLGVGAVYLWYYDHIEFAYPYMPSRVTVLLPKPSPMPEWRVPLAPFDFALWVALIVSIATVAFVLFYMNHYLQRFSSHHVSPNEFQSWSGVFLRAVGMAVGQSPQNAFSAGSTLRIVFTTFEILFLLYGTVYSSALASVVTVPAYYPPIDNMRQLYASGLPWTADHIAWVKNLMDADEPFIKDLLSKFEVHDQEMLSQLAKKGGYGFTIELTNGGHVSEASFLSADMINNLHVMKEALYYTYSTTIARKGSPYVDELNKLLHKCFDTGLLQLWESDMISKHGSSIIQTAFKLSKSAKASNEHHELVKLKLKHTQGAFILLLLGNTIGTLVFLFEFYCKSMKQVVKPTKMN

>AlinIR41a.5

MDLHSDWCNLMCFLIITQHIGSNWCVDHDEMTTGLAHEIAEHYFSEYNECITVVADLGALQKFSPPNNSFIRVSFDHTNDSCDPSVKESLRNSFREKCVRYVVQIAKPNCFFPSWFESMNLGYERHNPTVVFLPSINADDQNYGDDLLSKNETNISADILVAEISDAEEWAVKIYTNNFYQLTHEPERISKIFLDEWHPSKGFRFKADLLPNKLKDLKLKTLRVFTVQYLPYSSYDPFDGSEVRMVKEFCNVVNCTAVGLTNDGNWGTFDEETNTGTGQMGAIHSGEADIGVGGNVVWLEFFPHLDFSDAILGGASAIIAPRPKVLGGWFTPFLPFPLDLWVVVWAVVIASAILLYLFTTLTIRTIPHLAEKHRRNEKFVTFTDSLLRAIGMLISQQPSNLVTGSPVRHLFTSMEVIFLVITTCYCAQLYDFLTVPRTTKPIDTVKDVVENNLTWLAPSDLWIYALKHSDDPVVAKFVQLFQAYPPEEIIELSEKGEVGVTVEKLAGGHYVEDSYISYKFISQSRVSTGPDIYGMTPITLFMQKGSPYTEALNRFHGHMQNGGLHFAWEAGTARDHLNYTIQEGIAQSSRKQVYPPKVLRLADLEGAFLIHFIGVAVSIIVFFIESRVGKKKKSL

>AlinIR75d

MGALLPYSLITQYFINIHVSSIIVVSCCTTSQTAQLLRHLSQRGITASWAVDNLSPLEVRRSGIVLDLSCNQSKEILHDMSSRKMFGLEMEWLLMSEGSAPEEAELPDLYILPGSSVTLSVTSPSSISFYDTYRITRRLPYKFTLLGAVARDEDVLPQWKRPSRVNYEQNLLTTVSVIHSLDIRKLTDPDVAEEDRWPAIHFPVVVNVAYQLNFKFDLRLESVHGWKFPNGSFEGMIGVMEREEVDFGASGVIMREDRRKHVDYTVDYFEFKTGIIFKQPSLSSVSNIYLLPFSRHVWAACGGLLLFVLIILCIAVSSGDAQTFTPPATFLDMVNIVLGFVCQQGSYLAPVTISGRIVVFVSSLAALFLYTSYSANIVALLQSTSSVLKTLKDLTNSHLGLKVQINEYHLGYFLEAVDEDVITLYNKKVKNQPETFVNGTRGVEFMRTGDFAFCVEFDLAYKQISKTFQEEEKCGLGEMHLFFVPRLSIPVIKRSGHREHFTQTIIWQWESGMLDRISRIWLARRPRCESTGGGYLRVGLKDFNPALKVILVGIIISIWFFLCELITDRGFKAYYRKIKHNQEKIMGDDGHLIADLCFPSWKMLFKNKRF

>AlinIR75q

MQQVGLVGKDYNYIITSLDLHTIDLDIFKWAGTNITGVRLVNTESEHYREIMELMLAIKKEEEEEAYRPFTYDEQRRKRRNISSTLKSEDLGSNKIMSATSGRKTYSKRELQNKEGSRIIPPVEAMLIYDAVVLAAQALHSLSHVNPKQINCLMRSAWESGYSVINYMKMSEFYGLTGEVKFDNEGFRTDVALDIIELTQSGLHVKGNWSTYGGVNIQYPEPETDLTEATDDLRNTTFVVIIALTHPYGMLKESKYTLVGNDRFEGFGIDLIHELSEMTGFNYTFRVQEDKSSGNPTTLPNGTRVWNGMIGEVLAGRADLAIADITITREREHDVDFTMPFMSLGISILYRQPRAAPPSLFSFLSPFSYEVWGYMLSAYLGVSFLLYLMARISPPEWTNPYACIEEPVELENQFSLSNSLWFTIGSLMQQGSEIAPIAVSTRLVAAIWWFFTLIMVSSYTANLAAFLTIEQKVSLFDNVQELADQEVIKYGAKRGGSTANFFRDSHDPTYKKMWEFMSSHPEVMTDSNEVGVDRVDSTTDYAFLSESTSIEYETERRCNLYKVGRELDEKGYGIAMRQNSTYRNVLSRSVVKLQEGGQLDELKKKWWKEKRGGGSCLESPSGGAEDLGLDNVGGVFVVLLGGCIFATFLAFGELSFAIYMMEDKESFKEEFKKELKFIMKCSGTSKPRKIPSISSSTSSNPSVRSKSRSASRSRTSTVRTPNFSLQFP

>AlinIR76b

MSPFVHLMLVAMCANYQTNLLATDNQNFTCILKSEEQIKKEVYKGKVIKILTFDEMPLSGARKDGKGGMIGEGVAFELVETLKEKFGFDYTVERMAPIVGDESHGALGKLVSREIDMVAAFIPVLPDAHEFVKFGKDLSQAAYYVMLKRPADSNSGSGLLAPFDTVVWLLILVSLAVVGPVFYGVMWLRDRLCPGDIDQVYPLSTCVWFVYGALMKQGSTLNPLADSARMVFATWWIFILILTAFYTANLTAFLTLSISTLPIKEIDDVAKDNRHWFALQGGPIEHAIKDKEDEKLRKLRDSAASGRATFLETKQESIILQKITNDWYYLDDSYSLTRMMYDDYNRKSDMNAESSLRCAYVLTEKPFLVRSLAFAYGKDSPLPDLFNPILERFIESGILQHKLNLDLPDAVICPKDLGNKERKLRNADLWTTYLVVFSGVSVAFMIFLIEIIWRFYRKVKGSNQGVFNKQQMFQSRLNTDKLLAMRDQVQTKINGRDYYMVTNKGGNSHFIPLRTPSALLFQYG

>AlinIR93a

MSTGLFLVQFVLLMIMLKDHECNAAIKLHNDKRANDTLLIIIDESFVDSLDRNIEQQVRQIVSEISSRILKKGAVDILYHSNANIYLEPDVTAVFSMTSCLDLWTLFNRGKKFDILFISLTEANCPRLPPDSGITVPLYRRGWEIPQIILDLREDGSLTWETTAIIFDDNLEEDMLKSIIEVLNRPKKSSEMACSVVLYRLHSFPKDIQNKKKKNMDNLLENLPPIDMVNNFLVLIDQRKITPTLELVKKMGLVIPTAQWMFVVRNLNVMKRRTSPEKRYVDLIGEGENVAFLINSTRADSRCDMGLLCNARQLVEKLIIAIEKSIEQEIILADSLSDEEWDVLKPKKLERREHILDFIKKKTRDEASDCDSCTEWIIRSSDSWGMDFVQSKGSYGNKESNSTTGALLEVGHWQPRSGLVLVDHLFPNIVGGFRGRTLPIASVHFPPWQFVKYDEFGQPSEYGGVVFNVLNELADKLNFTYEIVLLPNGTSAANKFTLHKELGEVVIDSSVEFAAWDQVVLDLKSNKVFLGAVAFVETEERKADVNFTHPVATDAYAFLVSRPKELSRALLFIQPFTGETWLCIIATILLAGPLLWFVHRVTPFYDHYSHRGKGGYTRLYNCFWYLYGALLQQGGGVMPEADSGRIVIGTWWLVVLVVVTTYSGSLVAFLTFPKMDKVISNVDQLLERSAVSGDGMITWSFPKISTIHRLLKDTDNKKFNMFYEASEKLEQLTPEIIAKIQNGEHVYIQRKTMLLYIMKQEFLRTQRCDYSIGSEEVLKERLGLVVKSNSPYLKIINQHIHDMHKVGLINKWLEDSLPKKDKCWMSTLGSSSSTHTVNMSDMQGCFFLLFIGVFTSILLIGGECFMKWWKLRKQKSIIQPFIS

>CnigIR1

PDVERRHTRVVGPTRHVLSWLLITYLLVTSSYGGGLSSVLTIPRYEPPIDTVADLHSSGLEWGQIDLVYLNSLRGRSDKVYQDLMDRYRVLTPDNLRSRIYTRDLAIAVERLAGGYFTLEEFIDEEALSKWLRPMREDIYWENVVLVVRKGLPFIEELNQIVDRLLASGILRAWEGQISRKWLIPRLQLAAQAGMRSYAQAPDEPIKLQMTNVQGSFALLILGLCLSLVVFLLEIAIHWKTNGAIQLEFSSTIKVNLGVNRPESRLKKRARKSALPPHDRQRHRGSNTDLIY

>CnigIR2

MDRRLWVTSAAHTSVCCVARVIVAPHYNTTWNGFPEVIQRERWEPELPVANLSFSTQMTWHRGKTNTNLTGVHFRAIVVVTSTQTDNLHERLVSENEKHLDPLARFNYALFLHLKEVYNFTFSVQATRSWGYKTKAGRFDGMMGVIQRNEADIGASSALIKKERLEIVDYAGHTWKFWPRFLFLHPSGQRLHTALLTPLSTKVWFCAILAGLIITLILHLSSCVHADEFCNLDGSWSSTLITIVGTFTLQGAGSSWSQISWRIASLTALLLASLLNIHYGAAVVGSLLIPAPHTIRTLQDLMESPLQVAFENVSYNREYVARTTDKLGRELIHRKKPEFVQLSEGVFKIRKGFFAFHTEGGSVFRLAAMTFTESQKCALSDVSLFTPAVMSMPVKKKSPLRELFARGLLLLSEYGIKVRLEKIWRASRPACSTSGDVQAVEFSSLLPAELLILIGTLTSVAILFGELICHRLSARRFNCAFQDHQSPPTDPPMVVVKKNDSANPWLKLRH

>CnigIR3

MTALCNTSVIMAVLILCLTSTVFCERQKDNSNNINLILDFIGHMSAASSIRGVHAFVCWDTGDLQLMKALSRNGVPASVYRGWQHWEQLPTFHMDGNSLLFILDLKCGKSVALLKKAGENGEFFRPPHGWLILHDVYPAGTLHPIDPTAGTMLPVSARANNGRNLVIQHHSALSTYNNTITPNAIELGDEEYNTADVYDISPRRRAGYTNGNSTHITQTLSRISKNNSEFKSEHINDDAPRGKIVDVCFYCEVFRDLNILVDSNVVVGRREADTKYTLLEAYRRRKQGELVVSELGYWVRLTGIVWQAAREVALRRLDLKRTKLVASIVVTNPETLDHLDDLHNRHIDTVTKLNYLLLLHAADILNASLELVVTDEWGYESNGSWSGLVGSLQRGESDVGGTALFVTADRMQLIDYIALTTPSVAAFVFRQPPLSLVSNLFTLPFSRAVWASAAALVVTCAGLLMAATRWEWRRGDAQLASYLQQFQAAAAAGQLRDKWGDVAMLAVGAVCQQGSPAESRGVPGRIVTLSLLVTVMFLYTSYSASIVVLLQSTTTSIRTLADLLYSPLGLGVHDIVYNRHFFPAADDPVRRALYRQKVAPPGGEERFMTLEEGVRRMRTEPFAFHSELSPTWQLVQETFREEDKCGLQAIPFLQLMHPYIAVQKNTSYKEMFKIAYRRLWERGLQHRQLSRLYTMRKPPCAAGRGTSFVSVGIADCYPALLVPVYGVAIAVLVVLLEILFHRRKGKKTPTLTTTNIRRNEGHKSSSFSVCTLQ

>CnigIR4

LVYFARLAAARSVGGPKGDMSAPTLRLLAAVCLQLCSARLHPQAASMASDYLGHRNAHTVVTYMCSEHDSSRLAHLLLARHRVWLMLSPDPEDIPYNSYIFLDYSCEQNQILLTQCAKAEMMNTSTRWLVVSTQEPDERLQVRVDSDVTWATWGGGGSGSVSGAVLWGLIRLHPGWTLERWPVGRWAPSAGLHYDNTRLQWVAKKNLRRLALGAALPIVDTPLDNLSERLKNVNDRQLDTMARFGWSLSTTLAELLNFSIILYRVRNFGSLINDFEMDGAVALIHNGTVEFGAAGFIMTTRRMDFMDYTGPGRLWAPEIMFRHPKSASVLTTIFKPYTAQLWVSSGALFVLILVVSRIFCWLEHKVTGAVDEIDNSWNSTFLLVSSAIGQQGVSRSSEWLSWRMLLFVSFLCTNLLDTHYAAGIVTSLLMPPPRTINNKKDLAESSLGFGLENVSYTYQFFVKSDDPVDRALCSRKLYPPGSRPNFFPAEVGVRKMASEAFAFHAEDVRVGPLIERFFSDDDKCALVFIPLLTPVATYHTVRKNSPVKELFNFGLRMMWERGHVQYLRKRWYFTRVRCLSETEYASVDLVPMSPAFMLLGCAFLLSGLLLLIEVRQYRRSQTEVVPVVANLSSENLKKKLGNSQTGRQKVHFSSTTQIKLLPSLDKSIYWDQSEPSK

>CnigIR5

LSIVTVSIVRMDRMCACCVRTREEVAAVREQLNALLATVSRIQAADSGCSGGGESGASHGTPQVSRVSCTVSAAEAPSCVPDAVDPPSPQGEWRVETRSRRSRRRAAVVAGRGASPVRPVSGRVAAPSAGSGQAHGGRGLLVIGSSNVRRVMEPLRQIAYRAGKESNVHSVCLPGGLIRDVEAALPAAIERTGCSRLQVVAHVGTNDACRLGSEDILSSYGRLAELVKTAGLERGVQAELSICSVVPRVDRGPLVWSRVEGLNQRLRRLCDNHGCRFLDLRYRVGNCRTPLDRSGVHYTKEAATRVAEYLWNAHGGFLG

>CnigIR8a

MWPLWVSVVAAQLQLASSQAASDPLLIRFLVVTEVNASWVGGELRANLSGLEARYAGLRLQLDLSAIEVDREHEVEEFQQKVCGTLATGVSALLDATWTGWRRLRDEAQHRGLPYLRLDATLANLVDAVDKYLQAREASDAALIFHTEEELDQALYHLIGNSVLRVIALNGLEADTVNRLKDMRPVASYFVIFADTAHMTELYNKAAAGGLVRKAERWALVFTDWEWRSFKSDQLNLSTALLQMKTSSCCALQAEPESCKCTLRKVAPAFLRSALSAVVESLAELHSKGMDVRAAPRQCSAADAAADDDGEGAADAEDATEPPANATGNYDAFLRALAPRGQSNSTLFFRAAAAQLTFNTPLQLRMVNRSEDVSLGDWSPEKGLQLNTQLKPAKRFFRVGTAEGVPWSFPVRDEQTGEPLVGPEGEPIWDGYCIDLLKKLAEPTHMNFDYELVPAKNNDFGSRSPSGSWTGLVGDLAMGETDMIIAPLTMTSEREEVIDFVAPYFDQSGISIVIRKPVRETSLFKFMTVLRLEVWLSIVGALTVTGIMIWLLDKYSPYSAQNNKEMYPYPCREFTLKESFWFALTSFTPQGGGEAPKALSGRTLVAAYWLFVVLMLATFTANLAAFLTVERMKSPVQSLEQLARQSRINYTVVMNSDTHEYFRNMKNAEDVLYNVWKDITLNSSSDQSKYRVWDYPIKEQYGHILQAIDQAGPVPNASIGFQKVIDQEEGKFAFIHDAAQIRYEVSKNCNLTEVGEMFAEQPYAIAVQQGSHLQEEISRQILDLQKDRYFESLTAKFWNNSAKGTCPNSDDSEGITLESLGGVFIATLFGLALAMITLAGEIFYYKRKKLTAVNVTSSSAKIPKKQVTIGKEFRPVMEKTAPRVSYISVFPRNQLY

>CnigIR25a

MILLLLTMGLLSNLFSALGQPTINYLHVNDDRNLIPNKALKQVVSDLSNQGMIFDGVFKATANGSDVEALIESMCLGYNTSIDQNKKIHVVLDTTLQDVSSEAVKYFTRALELPTISASCGQEGDLRYWRNIDKKQEKYLIQVMPPIDTIPEFIRSFCSEQNLTNAGILFDDTFIMDHKYKSLLQNVPTRHIINEIKFQNIAKQLSTFKQREVFNYFILGRIDTVNKVLEAAADMEFYGRQFGWYAVTQDEGNPSCQKCGKGASVLHVKPNDAERTVLGAENPKLTYQFYYELFRNTFLAIGQIMDEGSWPDMQYIPCEEYEENDNIPPVRKLNLLDALQQISMQNPGVYGQMILSSNGHSHMQFNMTAFNVSLSDNTATEVGTWAADLDSPFITKVKPSVPVTQYTVVVALQQPFVIKYQDENGNTKFKGYCIDLINAIRNITNFEIEIYEVADGKFGNMDEEGRWNGMIKDLIDKKAHIALGALSVMAERENVVDFTVPYYDLVGITILMKKPKTPTSLFKFLTVLENDVWLCILAAYFFTSFLMWVFDRWSPYSYQNNREKYKDDEEKREFDLKECLWFCMTSLTPQGGGEAPKNLSGRLVAATWWLFGFIIIASYTANLAAFLTVSRLDTPVESLDDLSKQYKIQYAPIVNSSAHVYFQRMAAIENRFYEIWKDMSLNDSLSEVERAKLAVWDYPVSDKYTKILQAMTEAGFPATMEEALERVRASKSSSEGFAFIGDATDIRYQVLTNCDLQMVGEEFSRKPYAIAVQQGSPLKDQFNNAILQLLNKRKLEKLKEQWWSQNPEKRNDCEKQDDQSDGISIQNIGGVFIVIFVGIGLACITLAFEYWWYKLRPQHNAVVEAAPPRTKSDSLQALNMMRSSFDKRYGRRQGVALAGVTNPW

>CnigIR76b

MQLSPVLKMVVTTVCSNYFLNGTKMEVPEDEPDPGCVLRIPKLMEGKTIRIGTLENPPLTLTNKTDGTLIGQGVIFEIVDILQHKLGFNYEVVKPESNVLGDENHGIIGLVHKKQVDLAVGYLPQFSQHAKLVQHSESLAEAPWVFLMKRPLVSASGTGLLAPFDGTVWYLVLASVVLMGPAIYLIILVRVRLCVGSERLTRIFPLSSCVWFVYGALMKQGSTLMPVTDSSRMLFATWWIFITLLTSFYTANLTAFLTLSRFTLQITNLKDIATKKAHWAAQKGSAMEYLVYNNDEYSFLNQSLQAGFGQFVDISDADMLLRIRKDDLVYLREKQHVEHTMFRDYLEKTRNPKVEEKDRCTFVMTKQPFLHLPISFYYPLNSSLAHIFDPLLKALVETGIVRHLLRKDLPQIEICPLDLGSKERQLRNSDLYMTYMIVVTGFCAATVAFFGEILTRQVKRCIAEAELQTGPSASYPDDWKNVKAQANRMPYTMYLNGNIINVKQPAYSITKDFQSSNRKSLSRQRNTNYVFQYTS

>DmelIR7a

MFHHLWLLMGLRSLAMGALHPPQPEAMTPLVAAALEILAEQVSPSQSTLAVMDLTQDAEHRDERQEQLMTIILRSVGSEMALRTFQKPPAEVPASFVVFLVNSAQAFNTLGFHFTDIHSTREFNFLILLTHRMSSRAERLQVLRDISRTCVRFHTSNVILLTEKRDGVVLVYAYRLLNMDCDLSVNLELIDIYKNGLFRHGHEARSFNRVLSLSGCPLQVSWYPLPPFVSFIGNSSDPEERAQIWRLTGIDGELIKLLASIFDFRILLEEPCNKCLSPDIKDDCSGCFDQVIISNSSILIGAMSGSHQHRSHFSFTSSYHQSSLVFIMHMSSQFGAVAQLAVPFTVIVWLALVVSSLLLVLVLWMRNRLVCGRSDLASHALQVLTTLMGNPLEARSLPRSSRLRILYAGWLLLVLVLRVVYQGKLFDSFRLPYHKPLPTEISELIRSNYTLINQEYLDYYPRELTVLTRNGSKDRFDYIQGLGKEGKFTTTSLIATMEYYNMMHWSTSRLTHIKEHIFLYQMVIYLRRHSLLKFAFDRKIKQLLSAGIIGYFVREFDACQYRKPFEEDYEVTPIPLDSFCGLYYISLIWLSAAVVAFILELLSQRIVWLRRIFE

>DmelIR7b

MKYWLYILSCCSLVASTMESSSDWDLAEALAQVVANSEMGRFKTLYIYTHTNSQSTGGHLEELLDQVLMIVPNNLQARRLLLQQSMEYKPYVHAVLALVDGLPSLSAIYARIRATQDLSHTLIYMSMPTDAYGEEMQATLRFLWRLSVLNVGVVLRPPGDHILMVSYFPFSALHGCQVISANVVNRYQVGTKRWASQDYFPSKLGNFYGCLLTCATWEDMPYLVWRPDGSGSFVGIEGALLQFMAENLNFTVGLYWMNKEEVLATFDESGRIFDEIFGHHADFSLGGFHFKPSAGSEIPYSQSTYYFMSHIMLVTNLQSAYSAYEKLSFPFTPLLWRAIGLVLILACLLLMLLVRWRHHHELPRNPYYELLVLTMGGNLEDRWVPQRFPSRLVLLTWLFATLVLRSGYQSGMYQLLRQDTQRNPPQTISEVLAQHFTIQLAEVNEARILASLPELRPEQLVYLEGSELQSFPALAQQSGSSARVAILTPYEYFGYFRKVHPMSRRLHLVRERIYTQQLAFYVRRHSHLVGVLNKQIQHAHTHGFLEHWTRQYVSAVDEKDESVARIASTSYSTLDGIDGDPSLSESEEDQQVAPVRQNVLSMRELAALFWLILWANLGAVVVFVLELLLPRIKLRKILRKMKSDIKKQISKLVRK

>DmelIR7c

MLHSAVHNVSLVYALVWAIDNYYGMATSTPLAVVQFPTSRESRRLHNDLIDAALGRSSGTGRIQFLLEDDRVEMTETDTDPPPPSGLTGRPIAIWFLDSLRSYFRLEMYLNQLGSPYKRNGFFLVIYTGLEDQPMESLKIMFRRLLNMYVLNVNVFLQRDGTVHLYTYYPYGPHHCQSSLPVYYTAFQDLAAPANGFGLTKPLFPRKLTNMHGCEMVVATFEHRPYVIIEDDPKTPGGRSIHGIEGLIFRSLAERMNFTIKLVEQKDKNRGEILPDGNFTGILKMMVDGEVNLTFVCFMYSKARSDLMLPSTSYTSFPIVLVVPSGGSISPMGRLTRPFRYIIWSCILVSLIFGFVLICLLKITALPGLRNLVLGRRNRLPFMGMWASLLGGLALYNPQRNFARYILVMWLLQTLILRAAYTGQLYLLLQDVEMRSPIKSLSEVLAKDYEFRILPALRTIFKDSMPTTNFHAVLSLEESLYRLRDEDDPGITVALLQPTVNQFDFRSGPNKRHLTVLPDPLMTAPLTFYMRPHSYFKRRIDRLIMAMMSSGIVARYRKMYMDRIKRVSKRRNLEPKPLSIWRLSGIFVCCAGLYLVALIVFILEILTTNHRRLRRAFNVINRYAA

>DmelIR7d

MDIRCVVALLLGLCKVQAVVWPHQHLLEEQLASQISATLQKIFINGLAVYNFGVFISTSYEEMDRDRVILVHQVLNRNLYPPNFPVAVVLASKMNRKITAQVFTQLLFVQNAEQAIAIAEGVNRNGLCVIVLLTSQPERPIMTKIFTYFMQERYNINVVILVPRLHGVQAFNVRPYTPTSCSSLEPVEIDIKDGDLWDVFPRRLKNLHGCPLSVIVWDIPPYMRINWKSSDPMDGLDGLDGLLLRIVARKMNFTLKLIPNEPNGLIGGSSFMNGTFTGAYKMLRERRANITIGCAACTPERSTFLEATSPYSQMSYIIVLQARGGYSIYEVMLFPFEKYTWLLLSTILGLHWIVGSRWRMPSPILAGWMLWIFVIRASYEASVFNFIQNSPVKPSPRTLDQALSGGFRFITDHASYRMTLKIPSFQGKTLISAGQPVDVFDALLKAPWKTGAFTSRAFLADHLVRHRKHRNQLVILAEKIVDNMLCMYFPHGSYFAWEINKLLFNMRSFGIFQHHSQILAWDNLPTTTDTDTPGKRIHSSTESVATGFAESMSFVVAALNCLMGALCISIVVFGLELLSRRRHWTGLEWLFERV

>DmelIR7e

MNISALLNSYYDLSGEQMNHINEFVARAVLHVVHHYILSVTPSLVLTLCCRSNHTCNFYNKMMSTLFREWGLAPLQIVNVLRGVPWHPVPGRRHFNVIFTDSFAAFEEIRMEYYSREYNYNEHYFIFLQARDRLLQGEMRLIFDYCWRYRLIHCSIQVQKSNGDILFYSYYPFGEHGCSDMEPQLINRYNGSMLVEPDLFPRKLRNFFGCPLRCALWDVPPFLTLDEDQEEVLRVNGGYEGRLLLALAEKMNFTIAVRKVHVNMRDEALEMLRRDEVDLTLGGIRQTVARGMVATSSHNYHQTREVFGVLASSYELSSFDILFYPYRLQIWMGILGVVALSALIQLIVGRMLRERMGSRFWLNLELVFVGMPLLECPRSHTARLYCVMLMMYTLIIRTIYQGLLYHLIRTHQLNRWPQTIESLVQKNFTVVLTPIVQEVLDEIPSVQHMRFRLLEANSELDPLYFLEANHQLRQHVTASALDIFIHFNRLSADKVHQRGEQGSGAHFEIVPEDIISMQLTMYLAKHSFLIDQLNEEIMWMRSVGLLSVWSRWELSESYLRNEQSFQVLGTMELYAIFLMVLVGLIVGLLVFILELVSMRSIYLRKLFT

>DmelIR7f

MNTTSDSNAGSSLSSGSGYSIYKSYLENSRIDMQGEDANLYVARALRLVIENVLAQLSTTLVVTISTRHLGTAHWFEYMMNILMDSWRMVAVQLLRIRPDLVVNPVPGRKRVSLLMVDSYQGLLDTNITASNANFDDPDYYFIFLQARDHLIPKELQLILDHCLAHFWLHCNVMIQTAQVEVLVYTYYPYTADACQKAYPIPVNTFDGRKWKASQMFPDKLSQMHGCPLTVLTWHQPPFVELVWDPKHNRSRGSGFEIQLVEHLARRMNFSLELVNIALLRPNAYRLAEGSSEGPIEKLLQRNVNISMGYFRKTARRNQLLTTPMSYYSANLVAVLQLERYRIGSLALLVFPFELSVWMLLLLALLIHLGIHLPSARRGNEEDGGGGLQVVALLLGAALARLPRSWRHRFIAAHWLWASIPLRISYQSLLFHLIRLQLYNTPSFSLDQLLAEGFQGICTANTQRLLLEMPQLARDPDSIQSVDTPFDWDVLNVLTRNRNRKIFAVANQDVTLSFLHSSAHPNAFHVVKQPVNVEYAGMYMPKHSFLYEKMDDDIRRLDASGFIHAWRRASFASVHRKEQVHMTSRRYINHAKLSGIYMVMAGLYLLAGLLFAGEVLLRQRN

>DmelIR7g

MNVTSLLNFESMKYIGAQTQAASINHHVAQALRVFIEDFYQRIAPAFIVVLSCRRPSPMNFYRNIMQLLYESVDTMIVQLVLVELGRPRRIAGPRTHNLLLVDSLDALLDIEIHTYTAQSDTSEYYFIFLQQRDALIPHDMQGVFAYCWRHQLINCNVMTQSSGGQVLLHTYFPYAPGQCNDSQPTRINMFLGESWKHRDYFPSKLHNLNGCPLIVLARKVSPFLDLDEGQRELRGLEGRLLQELSRRMNFSIQFSGLQDQLKNRTTWTEKQLLQKLVQERIAHLAIGYVRKRIQYATNLTPVFPHYSNRVVGCLLLNAHNLTSLEIWSFPFQALTWICLLLFASWLIFGLIVRSMYSALLFFILRYHLHQRLPGNLQDLTHGDYAAVMGRTTLQDLREVPSLQDLLGLKSVIVTSEREEEVLRTLDRCTLREGAGSHPLFFGLISQDALLHLTQRGHRAGAYHIIPQDVLEQQLAIYLQKHSHLASHLDHLVMSIRSVGLVHHWAGQMASERYFRSRFLYREKRIRQPDLWAVYILTAGLYLLSLVVFICELLASRRAGL

>DmelIR8a

MELPLLVLLLALRFAGSEVLKITFWIEPVQRAEFDTDIAMVLKELDALRLDVKVDDTTLTLTRSEDGLDMQRFCEILSTVGASAVIDLTYSHWEEGYNLVRSLGIGYVRLERIMRPFLDMFGDFMRQKRANNVAMVFMNARDAVEAMQQMLVGYPFRTLIMDASQTDPGQHFLERIRSLRPAPTYIALFARAAAMNGIFEKVQKADLFQRPLEWHFVFLDTRDRVFKYRRQAELCTRFTLNPRAICRSMPMPDLYCGSGFTMQRAMLLNVLRSLINAAQVSPGYPLAIYQDCNATASSSEVSDPLEKDDYNWLDMVHWSNFLAYAPPLPHIQDQFQSPVPGLTFAVNISAGYYSSEHEAKTDLAAWSSVGEMRLLNETISPARRFFRIGTAESIPWSYLRREEGTGELIRDRSGLPIWEGYCIDFIIRLSQKLNFEFEIVAPEVGHMGELNELGEWDGVVGDLVRGETDFAIAALKMYSEREEVIDFLPPYYEQTGISIAIRKPVRRTSLFKFMTVLRLEVWLSIVAALVGTAIMIWFMDKYSPYSSRNNRQAYPYACREFTLRESFWFALTSFTPQGGGEAPKAISGRMLVAAYWLFVVLMLATFTANLAAFLTVERMQTPVQSLEQLARQSRINYTVVKDSDTHQYFVNMKFAEDTLYRMWKELALNASKDFKKFRIWDYPIKEQYGHILLAINSSQPVADAKEGFANVDAHENADYAFIHDSAEIKYEITRNCNLTEVGEVFAEQPYAVAVQQGSHLGDELSYAILELQKDRFFEELKAKYWNQSNLPNCPLSEDQEGITLESLGGVFIATLFGLVLAMMTLGMEVLYYKKKQNALEITQVRPVNDSSGSGGNSSTAPPTATSTTKQAWHIPVLEAEEKPAKVSPPPSFETATFRGKKLPARITLGDGKFKPRHGLYARRNLGASDSHSGYME

>DmelIR10a

MAVLGTVFLLFMLDLKTLNLTRLNGLLVEPTRDLPQLELWLRAGSDHQDAENPYVQWFLLRTEIPLSIVTYQENRYWMDDPFGRRNLVLVMSLDQLLTNRGAAAPIQKASTFFYILADQDKDLSADEQLRLEGSCRQLWTQHKVYNRFFLTRDGVWIYDPFKRRDSAFGRLVRYYGSETLDKLLFRDMAGYPLRIQMFRSVYTRPEFDKETGLLTRVTGVDFLVAQMLRERLNFTMLLQQPEKKYFGERSANGSYNGAIGSIIKDGLDICLTGFFVKDYLVQQYMDFTVAVYDDELCIYVPKASRIPQSILPIFAVGYDIWLGFVLTAFACALIWLTLRVINLKLRIVSLGNQHIVGQALGIMVDTWVVWVRLNLSHLPASYAERMFIGTLCLVSVIFGAIFESSLATVYIHPLYYKDINTMQELDESGLKVVYKYSSMADDLFFSETSPLFASLNKKLSWNRDLRADVIDEVARFRNKAGVSRYTSLILESSHFTLLRKIWVVPECPKYYTISYVMPRDSPWEDAVNALLLRFLNAGLIVKWIQDEKSWVDIKMRSNILEADAESELVRVLTIGDLQLAFYVVIGGNLLAFLGFLAEHFRWKLQKKGV

>DmelIR11a

MRFAILWLFSGCLLPGIQVGIWVVVRAQPTGRDVLLSRLGNQQNELNTRRLANASSYLTRNYIANRINTLVVREICVECPYELSERQRQLVDQILASLAPELSVLLHKGTAEETTWEYTLFVVNDHTAFTGQVFIFPDELLEREFFCIVVVSEIQSRQFVRQTVGSIVKSNLQMHFVNVVVVAQLEDGTVGTYSYKLFKANCTPGITVRQINHFDRITGKPQQSMPDLYPVRNGHLGDCPFNVGAAHMPPHLIYKRHKDPPPASNVSIPAEDLAGIDWDLLQLLAKALKFRIQLYMPQEPSQIFGEGNVSGCFRQLADGTVSIAIGGLSGSDKRRSLFSKSTVYHQSNFVMVVRRDRYLGRLGPLILPFRGKLWGVIIVILLLAVLSTCWLRSRLGLSHPIEDLLTVIVGNPIPDHRLPGKGFLRYLLASWMLLTLVLRCAYQARLFDVLRLSRHRPLPKDLSGLIKDNYTMVANGYHDFYPLELTCRQPLDFSARFERVQRAAPDERLTTIALISNLAYWNHKHPNISRLTFVRQPIYMYHLVIYFPRRFFLRPAIDRKIKQLLSAGVMAHIERRYMQYENKRKVASNDPVLLRRITKSIMNGAYRIHGLVIVLATGMFILELLAGRSNGRLRRWMEWVHQ

>DmelIR20a

MLASLNRSTGLSAELLDLYGLVVHFLLSGEHTTLVYFNPAGLDCSWGVLWQRNLTAHPQIVWQRNYSYPDLYYQFNAKLLVLACLPMDSRAAIQLEILANSLSHLRTVVRLLIEVAGPDQVTLARQYLSFCLRRSMLHVELYFRDYHHSLILYSFRAFPSFELVMRWISVGQGVKLFLHKLDDLRGHRLRVIPDLSPPNTFFYRDARGDNQVTGYLWDFLATFAGRLNAGLEVVRPSWRAGSASDSSYMLEYSAKGLIDVGLTTTLITKWNLWAIHQYTYPLLVSSWCTMLPVEKPLATPDLFGRIVCPTLAMTLLLIILVTWLVFRQLRCLTRLKNSRPARIVPHLLTLLLLTTCSAQLLSLLIFPPYHVRIASFEDLLRGDQKILGMRNEFYNFDGAFRARYAGVFYLIDDPNELYDLRNHFNTTWAYTMPYIKWLVIKTQQRHFSKPLFRWSKDLCFFDFMPTSVIVAPDSIYWESIKDFTFRIHQAGLMKHWIRKSFYDMIKAGKMSIKDYSDLETLKPLNIGDLEIVWRVCGAAIAVASAIFIMELLYFYINVFFNSL

>DmelIR21a

MSYYWVALVLFTAQAFSIEGDRSASYQEKCISRRLINHYQLNKEIFGVGMCDGNNENEFRQKRRIVPTFQGNPRPRGELLASKFHVNSYNFEQTNSLVGLVNKIAQEYLNKCPPVIYYDSFVEKSDGLILENLFKTIPITFYHGEINADYEAKNKRFTSHIDCNCKSYILFLSDPLMTRKILGPQTESRVVLVSRSTQWRLRDFLSSELSSNIVNLLVIGESLMADPMRERPYVLYTHKLYADGLGSNTPVVLTSWIKGALSRPHINLFPSKFQFGFAGHRFQISAANQPPFIFRIRTLDSSGMGQLRWDGVEFRLLTMISKRLNFSIDITETPTRSNTRGVVDTIQEQIIERTVDIGMSGIYITQERLMDSAMSVGHSPDCAAFITLASKALPKYRAIMGPFQWPVWVALICVYLGGIFPIVFTDRLTLSHLMGNWGEVENMFWYVFGMFTNAFSFTGKYSWSNTRKNSTRLLIGAYWLFTIIITSCYTGSIIAFVTLPAFPDTVDSVLDLLGLFFRVGTLNNGGWETWFQNSTHIPTSRLYKKMEFVGSVDEGIGNVTQSFFWNYAFLGSKAQLEYLVQSNFSDENISRRSALHLSEECFALFQIGFLFPRESVYKIKIDSMILLAQQSGLIAKINNEVSWVMQRSSSGRLLQASSSNSLREIIQEERQLTTADTEGMFLLMALGYFLGATALVSEIVGGITNKCRQIIKRSRKSAASSWSSASSGSMLRTNAEQLSHDKRKANRREAAEVAQKMSFGMRELNLTRATLREIYGSYGAPETDHGQLDIVHTEFPNSSAKLNNIEDEESREALESLQRLDEFMDQMDNDGNPSSHTFRIDN

>DmelIR25a

MILMNPKTSKILWLLGFLSLLSSFSLEIAAQTTQNINVLFINEVDNEPAAKAVEVVLTYLKKNIRYGLSVQLDSIEANKSDAKVLLEAICNKYATSIEKKQTPHLILDTTKSGIASETVKSFTQALGLPTISASYGQQGDLRQWRDLDEAKQKYLLQVMPPADIIPEAIRSIVIHMNITNAAILYDDSFVMDHKYKSLLQNIQTRHVITAIAKDGKREREEQIEKLRNLDINNFFILGTLQSIRMVLESVKPAYFERNFAWHAITQNEGEISSQRDNATIMFMKPMAYTQYRDRLGLLRTTYNLNEEPQLSSAFYFDLALRSFLTIKEMLQSGAWPKDMEYLNCDDFQGGNTPQRNLDLRDYFTKITEPTSYGTFDLVTQSTQPFNGHSFMKFEMDINVLQIRGGSSVNSKSIGKWISGLNSELIVKDEEQMKNLTADTVYRIFTVVQAPFIMRDETAPKGYKGYCIDLINEIAAIVHFDYTIQEVEDGKFGNMDENGQWNGIVKKLMDKQADIGLGSMSVMAEREIVIDFTVPYYDLVGITIMMQRPSSPSSLFKFLTVLETNVWLCILAAYFFTSFLMWIFDRWSPYSYQNNREKYKDDEEKREFNLKECLWFCMTSLTPQGGGEAPKNLSGRLVAATWWLFGFIIIASYTANLAAFLTVSRLDTPVESLDDLAKQYKILYAPLNGSSAMTYFERMSNIEQMFYEIWKDLSLNDSLTAVERSKLAVWDYPVSDKYTKMWQAMQEAKLPATLDEAVARVRNSTAATGFAFLGDATDIRYLQLTNCDLQVVGEEFSRKPYAIAVQQGSHLKDQFNNAILTLLNKRQLEKLKEKWWKNDEALAKCDKPEDQSDGISIQNIGGVFIVIFVGIGMACITLVFEYWWYRYRKNPRIIDVAEANAERSNAADHPGKLVDGVILGHSGEKFEKSKAALRPRFNQYPATFKPRF

>DmelIR31a

MNLLISMFILILAAGEGEIIPSMEESVVTNFVKSLVKTKQAIVFSCLFKDFKEISLALMRINQFVSVVNLNQSYSLTSILTRENYARTSVMVNARCSGSSELLFEASENRYFNKTYQWFLWGVDLEVQSLFPLNLNYVGPNAQITYVNETADGYAYWDIHSKGRHLKSNLEINLIATLINDTLNIARDIFHLQSIDFRGQFNGLTLRGASVIDKEDIISNEQIESILSRPTKDAGVAAFIKYHYELLGLLRERFNFTVNFRNSRGWAGRLGNTTFRLGLLGIVMRNEADIAASGAFNRINRFAEFDTIHQSWKFETAFLYRYTSDLDTHGKSGNFLSPFSDRVWLFCLLTLGAFSIIWVLFEIIDYKILRIRVNSQKLEHLNQKSSVICIKTTCIERILQTFGACCQQGLDPNPVDRSVRFLVMTLFLFSLVMYNYYTSSVVGGLLSSSDQGPSTVDEITASPLKISFEDIGYYKVLFRESQNRSITRLIEKKLSSSRSLNELPIFSHIEDAVPYLKAGGFAFHCEVVDAYPVISEYFDANEICDLREVSGLMEVEILNWILHKNSQYTEIFKTAMCNAQEKGFVERILRRRQIKKPACQSLYTVYPVSLSGVLPGFVILICGFGASLLLLCLEKVYAHFGPRKFCGF

>DmelIR40a

MHKFLALGLLPYLLGLLNSTRLTFIGNDESDTAIALTQIVRGLQQSSLAILALPSLALSDGVCQKERNVYLDDFLQRLHRSNYKSVVFSQTELFFQHIEENLQGANECISLILDEPNQLLNSLHDRHLGHRLSLFIFYWGARWPPSSRVIRFREPLRVVVVTRPRKKAFRIYYNQARPCSDSQLQLVNWYDGDNLGLQRIPLLPTALSVYANFKGRTFRVPVFHSPPWFWVTYCNNSFEEDEEFNSLDSIEKRKVRVTGGRDHRLLMLLSKHMNFRFKYIEAPGRTQGSMRSEDGKDSNDSFTGGIGLLQSGQADFFLGDVGLSWERRKAIEFSFFTLADSGAFATHAPRRLNEALAIMRPFKQDIWPHLILTIIFSGPIFYGIIALPYIWRRRWANSDVEHLGELYIHMTYLKEITPRLLKLKPRTVLSAHQMPHQLFQKCIWFTLRLFLKQSCNELHNGYRAKFLTIVYWIAATYVLADVYSAQLTSQFARPAREPPINTLQRLQAAMIHDGYRLYVEKESSSLEMLENGTELFRQLYALMRQQVINDPQGFFIDSVEAGIKLIAEGGEDKAVLGGRETLFFNVQQYGSNNFQLSQKLYTRYSAVAVQIGCPFLGSLNNVLMQLFESGILDKMTAAEYAKQYQEVEATRIYKGSVQAKNSEAYSRTESYDSTVISPLNLRMLQGAFIALGVGSLAAGVILLLEIVFIKLDQARLWMLCSRLQWIRYDRKV

>DmelIR41a

MFIDLSWSLVLSAIVGKYLNESTICIFWNDKFEFQLLHKSDYISFVGINIKSFDDNGGHYIIDTGLKKKELQNKHLFLDELVIKIIISIEVTHCETFVVFDKDIDRFVNAFNKASVYSIWRSLHNKFVFAHIANESPESRNHFFEDQPNILFVVRDHSSASSFDIKTNKFVGRKAENPSQMILVDRYLASEQRFQFGKSLFADKLNNLQGREVIIAGFDYPPYTVIKHNMSTNAQDMGVSGESDFKNVYIDGTETRIVLNFCEQFNCTIQIDSSAANDWGKVYPNMSGDGALGMLINRKADICIGAMYSWYEDYTYLDLSMYLVRSGITCLVPAPLRLTSWYLPLEPFKETLWAAILLCLCAEATGLVLAYKSEQALYVLPGYREGWWTCTSFGVCTTFKLFISQSGNSKAYSLTVRVLLFACFLNDLIITSIYGGGLASILTIPSMDEAADTVTRLRFHRLQWAANSEAWVSAIRASDEALVKDILYNFHIYSDDELLRLAQDQHMRIGFTVERLPFGHFAIGNYLGPQAIDQLVIMKDDIYFQYTVAFVPRLWPLLDKLNTLIYSWHSSGFDKYWEYRVVADNLNLKIQQQVQETMTGTKDIGPVPLGMSNFAGFIIVWILGSAIATLTFLLELSLTYILKQSNLK

>DmelIR47a

MRQIKLLVWLLVVGVVSSTEQLQFLKNFLEAVHKERSISTILLIQRKVHKNDFLHGLYPIFWPIICLDETKRVELVNNFNKDFLALVYMESEADTLLLSALAADLNHIRDARIMIWLQMSPSENFLDRIVFQASKQKFLNLVVIENTLKTRRFYPFPQPKVQVIDKPFEEKEIYPALWRNFMGKNAIAVPDLVPPRSFNSFDPKTGHRRESGSIYNVFKAFTQRYNITMLLKWPLIRNTTQEEIIGKSVRGEIDLPITGQLISFRHPNGSRSQPLLGMTALSIAVPCGPELPMFDRFFLFYGLATPITITGYYVLLNTIEIILGTLSDRIKRHPRRKKILNLVLNLRVFSCILSLPTPQGNRLRSVKGQLTMVMSITGLILSCIVAAQTSTILTMKPQYRHIKNFQELSDSNITVVCNHLNYLTIKQQMDPKFMAKFMQNIWIVNSIEQMKMIFDLNTSYAYQTFSYKKDPFTLLQMHTTRKAFCRTPGLDLVSGLAYTAVLEKNSIYALALQDYTLKAFSAGLVYYWAEESIRDLISTVGRTQFEKLPIVIGYQSLKLQDYNVCWKILLIGGALAFCVFIVEVVVGLINRRI

>DmelIR48b

MILQQSSNLLKLLLLLAISSVRTQGLNDIIIELNQRLLISNNFLYCNQSDKLNEYEIKYLQHMPPISLMIFTSIESMNFTQVEYNLGADNKLFLIMGNEEPPYDFLHALNLHFQFAEYIIVIDEPVDLKKSTKWLDFVNHLWQQGYVQLLIYTSYDEKLYHKIIFPETVIEETLVEQYISIRGSFNNLYGYPVRVAAYNNAPRSMLYVNRWGKHIFAGFYMRFLRAFIDARNGSFVPVLTPSNSPGNCTLNLVNETVDVCADALAANPAAFSLTHGFRIASANVLVTHAKPLHSYRYLTAPFQWSVWACLVIYVLLVVNFLSFIGWLRSGKWEFSKYLLEVFSSLLFSGFYLKEIRGRERYILFGVLFIAGFVYSTEYLGLLKSMLISEVFEKQIDTFEALVESNITLMVDPYDKILFAKYNMPEILSPIMELVSFETLLKHRNRFDQDYAYILFSDRMALYDYAQQFLKHPKLLRIPIDFSFLYTGIPMRKRWFLKHHLGRAWYWAFESGLTRKLALDADFEAVRVGYLSFLITEHVEAQPLNVDYFVMPAIALAIGYILALLSFVIEMTAWRIREFLGCRKATMTSTGCSEGGHVDVD

>DmelIR48c

MSLLRIILIIIFLRIVSSIPDTIISHLSAELQIKIQIYFGLGNDLYDFSRLDGNYQKIIISHNISEEFKTYHDEPVLIIIRLERDLNLNLATLDVLRSYLTDRQYNDILLIDNDEENLNSYVDIRKAYWNAGFSQVLIYNSQQRTWSIKPYPYLQIRPTSLKEYIENRNTRNLMGYPLRVLVTNDPPHCFVDKDELPGSPNRYKGSIVTMLKIFADQLNATFQANPFREFRRYSTADCVQMVSDDEIDACGSIFIRTYTYATSQPVRLNRVVIMAPFGNPIEKFYYFFRPFDLYVWIGTGIIVVYIAVMGSLLHRWHFKEWNVGQYLLLAVQTLLNRELSLPQSSSGSKFMLLLLLFAIGFILSNLYVALLSMMLTTKLYQRPIENLADLKAANVNILLQTHNIRPNSVYGSSEELRERFLLVEESQHLEKRNGLDPSYAYVDSEDRMDFYLYQQKFLRRRRMKKLSNPVGYTWAVQVIKQNWVLEKHYNDHVQRFFETGLQNKLVDDVHELAVKAGFLHFFPTQTQTIEPLRLEDIVMAAMVLGGGHALAVICFLVELFA

>DmelIR51b

MCKVLTLLVVILLLALTNAAYNVTLLKSVLSLISTREPWINTPIFVGHNTQGGDLNDLIIWLHQTMGVTSLTMNLFLQPEHIRPLGHFKITRYNGIALFFCHDKHDIMWLTLDRNLRKLRRIRLIIILRNQRSGSQGAIKSIFNALWQYQFLNVLVLQRDQLYSYTPYPAMRFFKLDIHTEPLFPHAARNFHGYVVSTPAENDIPRVFHVHDPLTKSRKVLGYAYRTFVEYLDHYNASLRLTNPDENLDPTTSVNMNHIVQLIIDGQLEISLHPYVFTPPTATKSYPLLIYPNCLIVPMRNEIPRHMYLLRPFQLYSWYILLFAVFYITGILYCISPKLNKSSWPQRLGLNFLDAISKILFISPPITIYRPTWRHLIIFLQLSVLGFMSTSWYNIELDSFFTTIVVGEQVNSMDQLVHQQQRVLVKEYEINTFLRHVEPRLVEKVSRLLVPVNASEQVSALLSFNRSFAYPFTEERWQFFAMQQQYAFKPIFRFSSACLGSPHIGYPMRVDSHLETSLNHFILKIQDTGLLNHWVVSDFNDAMRAGYVRFVDNVLGYQSIDVDTLRLGWCVLGIGWILSALVFSCEYWHLYPWRFIA

>DmelIR52a

MALGWSVIILGFIGQLSAQILNYTQSRDLELLEGSLFRVLSRLNLEEEYNTLLIYGKECVFHSLLRKLEISAVTVPSGSTDYDWSFSTAILILSCGYDAENEENSYTLMKLQRTRRLIYLEDNSEPESVCMRYSLKEQHNIAMVKSDFDQSDTFYSCRLFQTPNYVEGHFFKDQPIYIENFQNMRGATIRTVADSLVPRTILYRDEKSGETKMMGYLGHMINTYAQKLNAKLHFIDTSKLGAKKPSVLDIMNWVNEDIVDIGTALASSLQFKNMDSVWYPYLLTGYCLMVPVPAKMPYNLVYSMIVDPLVLSIIFVMLCLFSVLIIYTQHLSWKNLTLANILLNDKSLRGLLGQSFPFPPNPSKHLKLIIFVLCFASVMITTMYEAYLQSYFTQPPSEPYIRSFRDIGNSSLKMAISRLEVNVLTSLNNSHFREISEDHLLIFDDLSEYLVLRDSFNTSFIFPVSVDRWNGYEEQQKLFAEPAFYLATNLCFNQFMLFSPPLRRYLPHRHLFEDHMMRQHEFGLVTFWKSQSFIEMVRLGLASMEDLSRKRNEEVSLLLDDISWILKLYLGAMFISSFCFILEILRCGERCKRLWRCRW

>DmelIR52b

MTWLVILLCFLGYMAAHIADISVQNQSLMDNELINLLLKLRNEEFYDTLLVYGKDCEFHSVIKNVDVAVVLVSDSMNFEWNFSSLTLILSCGPDIDNGGPNSTSIKLQRNRRLVLLKEDFQPSNICNIYTQKEQYNIALVRENFTKSKSIYTCRYFQDPNVDEVNLSGTKPIFIEQFQNMKGKAIRIVPDLLPPRVMLYQDANDGELKMIGYVANLITNFAQKVNATLQLDFLKPSTSITEISRMAKDDELDMGITLEASLNTSNLETSSYPYLLTSYCLMVQVPAKFPYNLVYALIVDPLVLGIIFVLFLLLSVLLIYSQKMSWQDLSVANILLNDKSLRGLLGQSFPFPLNASKKLRLIFTILCFASIMLTTMYEAYLQSFFTNPPSEPEICSFQDVGSYNRRIAMSALEVNGLIKTNNSHFREIRMDDLEIFDNMPECYELRDAFNLSYNYVVTGDRWRSYAEQQTLFKEPVFYFARDLCFSRLIFLSVPLRRHLPYRHLFDEHMMQQHEFGFVNYWMSHSFFDMVRLGLTSLKDLSRPLAYTPSLLMDDISWIMKIYLAAIVLCVFCFLLEIGVDKWKRWMKFRNLQILNTC

>DmelIR52c

MVWLIIILFCLGNSSSQILDVTNNSHLDFDYRLFGLLQRLQVEKSYDTLLVYGEDCAIPSLFERLQVPAVLVSSGSTNFDWNFSSLTLILSCNFQDEREENYRTLMKLQTSRRLILLKGHIKPESVCDFYSKKEQHNVAMVKENFYQLEVVYSCRLFQDQNYEKLNLFDGKSIYKDQFRNMHGAPIRTLSDKEPPRTIPYIDSKTGEEKFKGYVGMLISQFVKKVNATMQIREDLIKDDEEVSFVDITNFTSNDILDIGICEARTLEMSNYDAISYPYLMSSYCFMAPLPDSLPFSDVYMAIVAPSILIMFLIIFCICSVLIIYIQERSYRSLTIRSVLMNDICLRGFLAQPFPFPRQYNRKLKLIFMLVCFSSLISTTMYTAYLQAFLWGPPIEPRLTSFDDVKKSRYTMAINIYEREFLEALNVSLEDVEIYDYGKFSKLRSTFNTNYLFPVTALQWFTINEEQKLFKYKIFYYCDAFCLNQFDILSIPLRRHLPYRDIFEEHMLLQKEFGLTKYWIDQSYRDMIRANLTTFKDFSPLLENDYIEVHNLYWVFTMYFVGMGMGLCFFILEILRPLRYWRNCKIKCEYCYAFLKNFAK

>DmelIR52d

MVRIIIILLCLGYTKARILDATNTNHTDLEERLLSLLLRLQQEQFFNTLLIYGEDCAFSSLSRRLQVPTILVSSGSTSFEWNYSSLALILTCEFKAEREENYQTLKKLQMNRRLILLNGNIKPDSVCDFYSKKDQYNIAMVNNNFHQVGIIYACRLFQERNYEKVYLSEGNPIYVDQFRNMQGALLKSITFNLIPGSMAYRDPKTGQEKHIGYVANLLNNFVEKVNATLDMQVKLHKAGKKTSFYNITKWASEDLVDIGMSYAAYFEMTNFDTISYPYLMTSTCFMVPLPDMMPNSEIYMGIVDPPVLVVLIAIFCIFSVMLNYIKQRSWRSLSLVNVLLNDICLRGFLAQPFPFPRQSNRKLKLISMLVCFFSVITTTMYTSYLQSFMWGPPIDPKMCSFADLENSRYKLAIRRYDIEMLRPFNVSMDHVVVFDESSQLEYLRDSFDDNYMYPMSALSWSAFKEQQKLFAFPLFYYSEKLCLKPISFFSFPIRRHLPYRDLFEEHMLQQNEFGLSTYWIDRSFSDMVRLKLATMNDFSPPRLEDYIEVSDLSWVFGMYFTGLGISCCCFGLELLGLPSWTRRLRLTNWLRVRN

>DmelIR54a

MWTVITGIVLWAPVLVAGSAVDFIFRAAAEHSLSVIMIRIDYCPYNWAKDIFENQTIPVVVLSDSETFINIRMFSRPLHVACLPGHELQKDLALLENFTSSLMDFPSQKKIVYISNNFSDPTRMDYIFETCYHRRIWNIVGLLASDEHRYFYRYHLYPSFRTEYRSLESSTIFDKDFPNMHGHPLTVMPDQWLPRSVLYVDRRTGKQILAGSVGRFFHVLSWKLNATLQLSKKVTTGRFLNATALKELSESFSVDVPASLTIMERVEQLASTSYPMEVTHVCLMVPVARRIPIKDIYFILSSASNMFLAIVIVSSYGLALNLLRNMTHRDVRLVDFVLNDKALRGILGQSFNLPLSRSFSTRLIFLMLGIVGLNVSSIFGAGLDTLMAHPPRQFQARSFAGLRRTKIPLVTTEEDFPTWMKLRVPMLVVNVSEYNHLRNGRNTSNAYFASRLYWNLFSEQQKRFTRELFIYSTDDCLWSLALLSFQWPQNSLFTEPVSQLILEVNANGLYDFWVGMHYYDMTAAGLSGLEDPSLQLKEREHPTSLRIVDFQWMWQAYGTFMVIAILVFLLEVSWHRITSLFVSLVY

>DmelIR56a

MGSRFFIRNLILFGLLASSNMQIPFGELEKKFELDVDFLLGVTELVGHIQGLYSITVYADCIDIHPSIQQRIMDKFMVPVNTIGSNLSRPNYHKLDNSRIRIVLFTGLNDTILVNLNKTDVPYSDNFYMLAYASAIKNKCIELDFIEEVFTLLWKMSIQNAILLIRGEFMMEMWSYLYMGKIHKIKLTKPNSYLESLRKYNYRFSLEVINDPPAIFWYNSSEQADVTGGGNLSVSGPLGLIIINFLRHLNVTIDIVPIPGKQTSQYELFQQPDNLRAENGVNMVGSALLKYSPMVTQSRMCLLVSNRRMIPFSRFLDRLVSPGVHKLTFVSSIGIFVIKYFSHRPRSFVDAIFCTIRFFFAIPLPSIILNRLPVVDRFIEVFIIIFVQILLSSNISITTSALTTGFWEPPIINVETMRASGLHILTEDPTILQAFKENILPSSLADLVILVDEDTYFHHVTTLNNSYVYVVQAHNWQIFRLYQQQMTNEPFEIASEELCSKWRILGIPLNPKSPLRFMFKDYFYRILESGLREQWVHSGFKKFCEFNNLKKLPVDSVDSWQPLSIEFYSNVIRAYIIGLVIATLAFVAELLHNGYRRKNVKKT

>DmelIR56b

MLLDTDLASGVIRSPYSFDIPHAFIFNETQFVVPKFCGPYMEIVKHFAEVYHYQLFLDSLESLPKKSVVEQDIISGKYNLSLHGVIIRPEETSDFFNATQHSYPLELMTNCVMVPLAPELPKWMYMVWPLGKYIWTCLFLGTFYVALLLRYVHWREPGNATRSYTRNVLHAMALLMFSANMNMSVKLKHASIRVIIFYTLLYIFGFILTNYHLSHMTAFDMKPVFLRPIDTWSDLIHSRLRIVIHDSLLEELRWLPVYQALLASPSRSYAYVVTQDAWLFFNRQQKVLIQPYFHLSKVCFGGLFNALPMASNASFADSLNKFILNVWQAGLWNYWEELAFRYAEQAGYAKVFLDTYPVEPLNLEFFTTAWIVLSAGIPISSLAFCLELFIHRRKQRRPQYERFECYDY

>DmelIR56c

MQHLLNLLAPFGRMNVFQEIVWFVSPHQRLDQLDEFIMRIDEAFGKSATQTVVNNNTEMRMIYSSARRNHMSFVFTTGAEDPIMKVFSKVLLGRHFYVSMVIYVDKVGDMHPIYDLLTFAYNQQFFNSMVHFESMEGVNQLFGVSKFPVMSFENRTDFLKYMGKIWKQVQNARSDVGGFGFTTPLRQDLPHLFQSQGHYDGSTYRIIETFVRFINGSFKELIMPPDSLGGQVINMKDALQLIRERKMEFCAHAYALFMSDEELEKSYPLLVVQWCLMVPLYNSVSTYFYPLQPFDWNVWFFALGALLALVLLELMWLRMFGGWSGYRGAVLNSFCYIINVPIEGQLQQPCLLRFLLLATVFFHGFFLSAYYTSNLGSILTVNLFHAQINTMNDIVSAQLPVMIIDYEMEFLLNLNKELPQEFLELLRPVDSAVFSEHQTSFNSSFAYFVTEDHWEFLDEQQKHLKQRLFKLSSICFGSYHLAFPLQMDSSLWRDIEYFTFRIHSSGLLNFYARSSFGSALHAGLVQRMPDTQEYTSAGLQHLAIAFILLLVMSFLAGIVFVLETLSR

>DmelIR56d

MDNRAAELILRERNIFPTNGSDNITLLNNMFVLEMFYRITQLYHFKNFIFYISERLDLNNKDSQEFFHNFWTYFPMAPNLIITREHHLGIPMMQFISTPSLVMVFTTGKDDPIMELASHNQQGIHWLKTIFVLFPSLQSRDFETNPESLAQFTAEIKDVYDWVWRKQFINTFLITIKDNVFILDPYPTPSIVNKTGVWQAEEFFHKYAKNMKGYLVRTPILYDMPRVFKSDRPTNRYEKNFIHGTSGNLFLGFLEFVNATLMDTSANVTADYLNMTNLLDLVSQGVYETLIHSFTEITTKFVVSYSYPIGINDCCIMVPYRNQSPADQYMHEALQENVWVLISLFTLYITVAIYLCSPLRPRDLSAAFLQSICTLTYSVPTFIIRTPTLRMRYLYILLAIWGIVTSNLYISRMTSYFTTAPPVRQINTVQDVVEANLRIKMLAIEYERMAKSPLQYPESYLNQVDLVDKHMLDLHRDPFNTSFGYTVSSDRWRFLNLQQLHLRKPIFRLTEICEGPFYHVFPLHKDSHMRSVMTEYIMIAQQAGLMNHWERETFWEAVHLHRIHVHLFDDEPMALSLDFFSSLLRTWTLGLILAGLAFAAEMKWHEHVTFKRRPVIRITRKPRSFLRRFMKL

>DmelIR60a

MWCNNPGLIIIIFLGQILNLCQGIVNLSNETANTVIFMLPEKDLGPDVWKAGVGCLDSFAQIFFFRNPKERFTRAYNLMLVHAFHLSSPADQIQEGFSKLINEAVTNPGPPDREELFQMRVASDYNITNGTEDKGELILADNYVIVVDSVDRLKELMKKKIVEMRSWNPGARFLVLFHNATCRNRPLGVASNIFKDLMEMFYVHRVALLYANSTMNYNLLVNDYYSNVNCRILNVQSVGQCHDGKLYPNNAVVKASMQDYVSGFSPRNCTFFACSSISAPFVEADCILGLEMRILGFMKNRLKFDVNQTCSLESRGEMDGPANWTGLLGKVQNNECDFVFGGYYPDNEVADHFWGSDTYLQDAHTWYIKMADRRPAWQALVGIFEAYTWIGFILILIISWLFWFTLVMILPEPKYYQQLSLTAINALAVTISIAVQERPICETTRLFFMALTLYGLNVVATYTSKMIATFQDPGYLHQLDELTEVVAAGIPFGGHEESRDWFENDDDMWIFNGYNISPEFIPQSKNLEAVKWGQRCILSNRMYTMQSPLADVIYAFPNNVFSSPVQMIMKAGFPFLFEMNSIIRLMRDVGIFQKIDADFRYNNTYLNRINKMRPQFPETAIVLTTEHLKGPFFILVVGSCWAALTFIGELIIHRWRTQLVSTSEQQDRRSDKRRRRRRRRKPEKDNRWQRQVQVAPVVRFTPVKRRKVFQGQTSQK

>DmelIR60b

MRRSLYLIIAIGLVDVHCVSLRYILNALENELQYRAILLVESASEIESCWEQKYIQGAVPILNFNANQSLYLKDALNTNILALVCLNENVESTMQALYENLEDMRDTPTILFVLSDSKVQDVFLECLRRKMLNVLAFKGLDRGFVYSFRAFPTFRVIERNVMDILQYFEQQLEDLGGHTLTTLPDNIIPRTVVYKSPDGSRQLAGYLYPFLRNYVSTINATLKVCWHLVPEDGMIQLGEVVRLSEIHDVDFPLGMHGIEHGSTSQNVPLEVSSWFLMLPMEPSLSRAQFFIMLGFEKVTPVLLLLTILLSTAHRIEMGLRPSWRCYVLGDRVLQGTLGQAFFLPRRLSVKLMLVYSLILLNGFTFSNYSITSLETWLVHPPSGHPIHSWEQMRTLNLKVLIVPSELDSMTKALGKQFTESNSDLFELSKSGNFQDKRLAMDQSYAYPVTCTLWPLLEHAQIRLPKPEFRRSREMVLIPLLIMAMPLPKNSMFHKSLNRYRALTHQSGLYEFWFKRSFNELVALRKIHYKVNGDHQIYRDFEWQDFSYVWLGFVGGTIASILVLLAEIGYHRWQLNQN

>DmelIR60d

MRLAIYVAFLSSIGNRSGFLSSLLMSLGKELHYKTILLVGGSSTCWSLEPFETGVPILNLRGENNAYPQDTFNSQMLALACLQTESEDAVKLLYRSLKDMRDTPTLLFASSEEHIHDTLFLGCFRENMLNVLALTASSKEFIYSYQAFPTFRVIKRKLVEIHRYFEPQLKDLGGHIVSALPGNIMPRTMCYRNAEGERQLAGYLNTFIRNYVESINGTLRISWGLVPEDDMRHLTISRLSKIQHVDFPLGIIPLYNKTDKQHVYMEISSWFLMLPMETSVPRAHLFVKLGLERLLPIIVVVGAVLGNAHRIEVGLGPSWRCYYLADKVLRGALAQPIVLPRRLSPKLMLIYSLLLLSGFFLSNYYMASLTTWLVHPPASDRILEWDQLRYLHLKVLTIPEEFKYMSLILGTDFMTAYGSIFQLTNSTDFQRRRISMDPSYAYPVTTSLWPFLELSQVRLRRPLFRRSYDMVLQPFQVMSLPLPRNSIFHKSLLRYAALTRETGLYYYWFRRSYYELVALGKISYKEEEGNPYCDLKWNDFRIVWLAFLGGTIISCLALLLEVAHYRWHLGNSSL

>DmelIR60e

MVIKMISFLLVSVLLCLVGASDSESMQVQVLQDLNLALQTELNVFIDFECCATSEILHKLDSPRILLSSNSREARDLRIRGNFTESTLIIVSVMDSDLNPLVASLLPRLLDELHELHIVFLSNEEPGFPKQDLYTYCFKEGFVNVILMSGKGLYSYLPYPSIQPISLSNVSEYFDRARIIRNFQGFPVRILRSTLAPRDFEYSNEQGGLVRAGYLFTAVKELTYRYNATIESVPIPDLPEYDVYLAVAEMLHTKKIDIVCYFKDFSLEVAYTAPLSIIREYFMAPHARPISSYLYYSKPFGWTLWAVVISTVLYGTVMLHLAARGARVEIGKCLLYSLSHILYNCHQKIRVAGWRDVAIHGILTIGGFILTNVYLATLSSILTSGLYDEEYNTLEDLARAPYPSLHDEYYRSQMKAKTFLPERLRRNSLSLNATLLKAYRDGLNQSYIYILYEDRLELILMQQYLLKTPRFNMIRQAVGFTLESYCVSNSLPYLAMTSEFMRRLQEHGISIKMKADTFRELIHQGIYTLMRDDEPPAKAFDLDYYFFAFVLWTVGLISSLLVFFAELVSGHL

>DmelIR62a

MYLQFLFALFLSRYQIVATENFDRAFELALFLDRIGRVHRLHAITIVNSLGSVDPSYLDDLHRGLMCNSSNHFYMLPQMTATDKDSSHVHFSSLQDEETIYLVFARDSKDAVIYLQAERARGRRYTRTMFLLRKQESQKDIKYFFELLWKLQFRSALVVVAARNFYQMDPYPTVRVIRMRRLSSYDPHHVFPPANRKNFRGYRMRLPVQQDVPNTFWYKNRRTKAWELAGLGGILINQLMMHLNVTMDLFRFEVNGSSLLNMAALTDLIVKGKVELSPHLYDTLQSNTSVDYSYPTQVAPRCFMIPLDNEISRSLYVFLPFSLTMWLCLLFVLLVVHFVYVRRLIPDGHFWAILGVPGAGQVRYGNRKPVRRFSTFLILFGIFILGQTYSTKLTSSLTVTLIRRPDNSLEELFLLPYRILVLPTDVYAIVDSLGHAEQFSTKFSCTDAENFSQKRISMHPEYIYPISTIRWRFFDMQQRFLRKKRFYFSKICHGSFPYQYQLRVDSHLKDALHRFLLHVQQAGLHDLWLDTCYRKAHRMGYLKDFSTLAELEEKLRLRPLALNLLVPAFSLFLCGMLGSGIAFLVEIRHSFGCRQKPPSINRNPGD

>DmelIR64a

MHWWLLVFLPLSCQGLPEHELLELELDYGLAEPQRTSLLQSSLILQFSQDYKHIPRITYFTCQKPHLQTPNQIPNAAEHRDAFAAKNFQLIKSLYESELFVRIVLLDVLAQSPTSGRPNRPGNGPTGGFSQTPSQAQSNSEWLEGVLRMEALRQIAVVDLACGAVSRRFLELASAKMLYSEKFHWLLIEDFAWHGRTQTAEGSGKRDDGEMEEEEPPGQQIQATDDEDLPSIESFLGGMNLYMNTELTLAKRMSEAAHYTLFDVWNPGLNYGGHVNLTEIGSFTPTEGIQLHTWFRTTSTVRRRMDMQHARVRCMVVVTNKNMTGTLMYYLTHTMSGHIDTMNRFNFNLLMAVRDMFNWTFVLSRTTSWGYVKNGRFDGMIGALIRNETDIGGAPIFYWLERHKWIDVAGRSWSSRPCFIFRHPRSTQKDRIVFLQPFTNDVWILIVGCGVLTVFILWFLTTIEWKLVPHDGSALIKPKGGAPPRHHYQQQQQQEQVEAPVRPITAVSVVVSKEKVEEKQEEYEDSTPIDAGTLWQRCYQKLNKYIKDRKAKQKKAPERVGLFLESVLFFVGIICQQGLGFSTSFVSGRCIVITSLLFSFCIYQFYSASIVGTLLMEKPKTIKTLSDLVHSSLKVGMEDILYNRDYFLHTKDPVSMELYAKKITSVPTTKENEADEDEPVDPNPVSTDPAKSYRDIVHSHETGAHAKDNAASNWLDPETGLLRHLGFAFHVDVAAAYKIIAETFSEQDICDLTEVSMFPPQKTVSIMQKNSPMRKVISYGLRRVTETGILTYHFNVWHSRKPPCVKKIETSDLHVDMDTVSSALLILLFSYAITLMILGTEILYSKWHNRIQLKWVGAT

>DmelIR67a

MLPILVPVLLLFNETSWINPILTSIYKDRHHETVLLLQHSQHGNASGLERFPWPVFSFNEQMDFYVRGKYNSEMLVLIWQTGNSDWDLDLWQALDRSLLNMRKVRVLLLRKWEKIPTADVAATAEHLLFLHVAVIGQGNRIYRLQPYAPQSWLQVDPIESPIFIKIRNYFGRYIVTLPDQFPPRSIVYRNPKTDEIQMTGYVYKFLLEFIRIYNFTFRWQRPIVQGERMNLILLRNMTLNGTINLAISLCGFETPSELGVFSDVYDMEEWYIMVPRAQEISIADVYVVMVSGNFLIVLIIFYFIFTILDTCFGPLLLKERVDWSNLMLNERMISGIMGQSFNMSARNTISSKVTNATLFLLGLVLSTLYAAHLKTLLTKRPTSQQISNFKQLRDSPVTVFFEEAERFYLKHAWDRPIRYIKDQLNFRETIEYNALRMGLNRSNAFSALTSEWMIVAKRQELFKQPIFTVQPELRVIQTSVLLSLVMQSNSIYEDHINDLIHRVQSAGIVEYWKHQTLREMITMGMISQKDPFPYVAFREFKVGDLFWIWLLWVSFLFMSFVIFLCELLVDCFISKTLIRNKRPH

>DmelIR67b

MELLYLNTLQSLSLLEGNRLVQTVQELNNIYQTELNVFLEFGNGADILESAQGTFVPTLWIKNPQNQKVMKGNFTSCTLTILYLEDEHLDRGLYYLANWLWEYHHLEVLIFFNGGSYDKLIQIFSRCFNEGFVNVLVMLPGSDELYTFMPYQDLKILNLKSIKEFYSLSRKKMDLNGYNITSGLVIAGAPRWFSFRDRQNRLILTGYMLRMIVDFTNHFNGSVRLMNVLTVNDGLELLANRTIDFFPFLIRPLKSFSMSNILYLENCGLIVPTSRPLPNWVYLLRPYAFDTWIAWLIMLIYCSLALRILSKGQISISAAFLKVLRLVMYLSGSRDMGTRPTTRRLFLFVILTTSGFILTNLYVAQLSSNSAAGLYEKQINTWEDLDKSDSIWPLIDVDIKTMEKLIPDRTKLLKKIVPTLEADVDTYRRNLNTSCIHSGFFDRIDFALYQQKFLRFPIFRKFPHLLYQQPLQISAAFGRPYLQLFNWFVRKIFESGIYLKMKDDAYRHGIQSGLLNLAFRDRHLEVKSNDVEYYYLIAGLWFGGLTLATVCFLLELLIGYAKIKVTISCKMNIM

>DmelIR67c

MFCWLIFLNIILLSDRSESWSAREVIHQFNHDQQLQLNIYLDCNDVELQIGQEVSNLFVNSTADKMKILGRFSSHSLIIACFKDSTRNRTLNGVKELLWGLQYLPILFVVDSNMDFYFQQALRHGFIHVLALNFMNGSLYTYKPYPKVEVHQIKDMQKFYKLTKLRNLQGQAVRTTVETMTPRCFRYRNRHGQLVYAGYMYRMVKEFISTYNGTEEHVFGNVDTVPYKEGLAALKNGEIDMMPRIIHALEWYYFYRSHILYNIKTYIMVPWAEPLPKSLYFIQPFRGTVWITIMVSFVYASIVIWWIRYRQQGNSSLTQSFMDVLQLLFQLPLSKIWHFNMGTHQVVSFIVLFVFGFMLTNLYTAQLSSYLTTGLFKSQINTFDDLFREKRTLLVESFDAEVLHNMTKEKIIQKEFESIILITSIEEVFKHRKSLNTSYAYEAYEDRIAFELSQQRYLRVPIFKILKEVYDQRPVFVALRHGLPYVELFNNYLRRIFESGIWIKLQEDSFLEGIASGEISFRKSKSREIKIFDKDFYFFAYILLGMGWCVSTIALFLELWSFKYSVTNVLHEG

>DmelIR68a

MRCLWILIVAFISLAMATSIPIPIANPAPLSGYEMQLKILLQKILWVANVKRCFAVITDDLHYPIYDRIFFESVGRRVIPFFVMRTNESDDLQRPSRQVELFVKAIKSSDCELNVITILNGWQVQRFLGYIYDNRSLNMQKKFVLLHDLRLFESDMIHLWSVFIDAIFLKRQLDNKYTISTIAFPGILSGVLVMKNIANWELGKGLNGRILFADKTSNLFGTSLPVAISEHVPMVLWANATKSFQGVEVEIMNALGKALNFKPVYYKPNQTENMDWTELDGGASVAYGSGNPDGYAQNGTHIDSMLVDEVAAHSARFAIGDLHLFQVYLKLVELSAPHNFECLTFLTPESSTDNSWQTFILPFSAGMWVGVLLSLFVVGTVFYAISFLNAIINGNVSSEFFRCLRPNRNVPMDPKIYRRISFRIAISRYRSSKGDRMPRDLFDGYTNCILLTYSMLLYVALPRMPRNWPLRVLTGWYWIYCILLVATYRASFTAILANPAARVTIDTLEDLLRSHIPPSTGATENRQFFLEANDEVARKVGEKMEVFGYSDDLTSRIAKGQCAYYDNEFYLRYLRVADESGSALHIMKECVLYMPVVLAMEKNSALKPRVDASIQHLAEGLIAKWLKDAIEHLPAEALAQQEALMNIQKFWSSFVALLIGYVISMLTLLAERWHFKHIVMKHPMYDVYNPSLYYNFKRIYPQH

>DmelIR68b

MKFLVGLLLQWYLPGIYALAEIACRIAVEQNVQVTYLYRCASCPASFDADYSALELDLYRCVGSRLPVITRNMEAHELEPFRRTDSLSIFQIPAAEKGDSLVRRILDMLNPHQRRKHMHKYLFVWPNAGRHQLLRLFRGSWAKKLLYGLAITGRENGTFDFDPFAWGGLQVIQRLDGEVPYARKVKDLRGYPLRFSMFTDPLMAMPRSPVETAGYQAVDGVAARVVGEMLNASVTYVFPEDNESYGRCLPNGNYTGVVSDIVGGHTHFAPNSRFVLDCIWPAVEVLYPYTRRNLHLVVPASAIQPEYLIFVRVFRRTVWYLLLVTLLVVVLVFWVMQRLQRRIPRRGVIQFQATWYEILEMFGKTHVGEPAGRLSSFSSMRTFLMGWILFSYVLSTIYFAKLESGFVRPSYEEQVDRVDDLVHLDVHIYAVTTMYDAVRSALTEHQYGLLENRSRQLPLGIATSYYQPVVRRRDRRAAFIMRDFHARDFLAITYDSQAERPAYHIAREYLRSMICTYILPRGSPFLHRLESLYSGFLEHGFFEHWRQMDLITRVGASPDAEEFLEDLGDQTDTDSGSNELAIRNKKVVLTLDILQGAFYLWSVGIGISCLGFAVEHAHWFWRRQTLRNAVEARTS

>DmelIR75a

MQLVQLANFVLDNLVQSRIGFIVLFHCWQSDESLKFAQQFMKPIHPILVYHQFVQMRGVLNWSHLELSYMGHTQPTLAIYVDIKCDQTQDLLEEASREQIYNQHYHWLLVGNQSKLEFYDLFGLFNISIDADVSYVKEQIQDNNDSVAYAVHDVYNNGKIIGGQLNVTGSHEMSCDPFVCRRTRHLSSLQKRSKYGNREQLTDVVLRVATVVTQRPLTLSDDELIRFLSQENDTHIDSLARFGFHLTLILRDLLHCKMKFIFSDSWSKSDVVGGSVGAVVDQTADLTATPSLATEGRLKYLSAIIETGFFRSVCIFRTPHNAGLRGDVFLQPFSPLVWYLFGGVLSLIGVLLWITFYMECKRMQKRWRLDYLPSLLSTFLISFGAACIQSSSLIPRSAGGRLIYFALFLISFIMYNYYTSVVVSSLLSSPVKSKIKTMRQLAESSLTVGLEPLPFTKSYLNYSRLPEIHLFIKRKIESQTQNPELWLPAEQGVLRVRDNPGYVYVFETSSGYAYVERYFTAQEICDLNEVLFRPEQLFYTHLHRNSTYKELFRLRFLRILETGVYRKQRSYWVHMKLHCVAQNFVITVGMEYVAPLLLMLICADILVVVILLVELAWKRFFTRHLTFHP

>DmelIR75b

MLQLHNLILHNLIHMAKLSHVLILHCSLSHLALLAQSKNIFTQFQPLHSDIQLNDDFLNHNILKLGVFLDINCDKSGTVLDMASAKRFFSHRYHWLIYDRSMNFSVLESHFKEAQIFVDADVTYVTHDPFSKNFLLYDVYNKGRQLGGELNITADREIFCNKTNCRVERYLSELYTRSALQHRKSFTGLTMRATAVVTALPLNVSIKEIFDFMNSKYRIQLDTYARLGYQARQPLRDMLDCKFKYIFRDRWSDGNATGGMIGDLILDKADLAIAPFIYSFDRALFLQPITKFSVFREICMFRNPRSVSAGLSATEFLQPFSGGVWLTFALLLLLAGCLLWVTFILERRKQWKPSLLTSCLLSFGAGCIQGAWLTPRSMGGRMAFFALMVTSYLMYNYYTSIVVSKLLGQPIKSNIRTLQQLADSNLDVGIEPTVYTRIYVETSEEPDVRDLYRKKVLGSKRSPDKIWIPTEAGVLSVRDQEGFVYITGVATGYEFVRKHFLAHQICELNEIPLRDASHTHTVLAKRSPYAELIKLSELRMLETGVHFKHERSWMETKLHCYQHNHTVAVGLEYAAPLFIILLGAIILCMGILGLEVIWHRHCTLH

>DmelIR75c

MTSWPLYRLIVFNLLEINLSNLMVFHCWSIKEAFPLVEMLNQNGIFSQYIDVQNPDNLANVHKEYLDSDLVRLGVFLDLGCDKAELVTNQSSRARLYNQNLHWLLYDEAGNFTKLTQLFEGANLSLNADVTYVSREDEERFILHDVYNKGSHLGGKLNITVDQTLQCNRSHCQVKEYLSELHLRPRLQHRMDLSSVTFRLAALVSVLPINSSEEELLEFLNSDRDSHMDSISRIGNRLIMHTQEILGFKLHYIWCGTWSVQDAFGGAIGMLTNESAELCTTPFVPSWNRLHYLHPMTEQAQFRAVCMFRTPHNAGIKAAVFLEPFMPSVWFAFAGLLIFAGVLLWMIFHLERHWMQRCLDFIPSLLSSCLISFGAACIQGSYLMPKSAGGRLAFIAVMLTSFLMYNYYTSIVVSTLLGSPVRSNIRTIQQLADSSLDVGFDTVPFTKTYLVSSPRPDIRSLYKQKVESKRDPNSVWLSPEEGVIRVRDQPGFVYTSEASFMYHFVEKHYLPREISDLNEIILRPESAVYGMVHLNSTYRQLLTQLQVRMLETGITSKQSRFFSKTKLHTFSNSFVIQVGMEYAAPLFISLLVAYFLALLILILEICWARYAKKKFSTIIPQNQ

>DmelIR75d

MKVQVAHWLPLIFFLLVSGTPRVAGSWRSEYSRQDPDPKTRWGNQLPDMLVAYYRHHGVHSLMLVVCHTDIADFRLWKLWQHFNLNNFYVQVSTESSLRDLQHVDALDEHKDAPPPKSFHANNSTHWETSFLLPALPYKMGILLLEFSSECALNLLRWSAASEHNYFTTNRFWLLLTEDPGDIDLLEDPEIFIPPDSELRVLHYENVGNFSCSLIDLYKVAAWKPLKRTLVGHNIRNSRHVIHALQHFGSAITYRQDLEGIVFNSAIVIAFPDLFTNIEDLSLRHIDTISKVNHRLMLELANRLNMSYNTYQTVNYGWRQPNGSFDGLMGRFQRYELDLAQLAIFMRLDRIALVDFVAETYRVRAGIMFRQPPLSAVANIFAMPFENDVWVSILMLLIITTVVLVLELFFSPHNHDMSYMDTLNFVWGAMCQQGFYVEVRNRSARIIVFTTFVAALFLFTSFSANIVALLQSPSDAIQSLSDLGQSPLEIGVQDTQYNKIYFTESTDPVTKNLYHKKIASKGENIYMRPLLGMEKMRTGLFAYQVELQAGYQIVSDTFSEPEKCGLMELEPFQLPMLAIPTRKNFPYKELIRRQLRWQREVSLVNREERKWIPQKPKCEGGVGGFVSIGITECRYALGIFGCGAAVSFVLFLFEFIFRHFKQVYRIIKGYREVQR

>DmelIR76a

MENLLVESYYFSTVLSFFAQQFFADSHATCIFWHPAFDFRLETVHPMPLIIMDWHRWANRSDQDVYDYKIKEDEFEGKGIPYNDWTLRLTVAIERSHCETFIAFQEQIPEFARYFYHASIYSIWRSLRNRFMFVYTKEFEDKKDSYLSGYIFQDQPNILVITSQYLNSSTFEIKTNRFVGPRNFNKNPEPVEFYILQRFDAKGTKATWETQSAMSSKMRNLKGREVVIGIFDYKPFMLLDYEKPPLYYDRFMNTTDVTIDGTDIQLMLIFCELYNCTIQVDTSEPYDWGDIYLNASGYGLVGMILDRRNDYGVGGMYLWYEAYEYMDMTHFLGRSGVTCLVPAPNRLISWTLLLRPFQFVLWMCVMLCLLLESLALGITRRWEHSSVAAGNSWISSLRFGCISTLKLFVNQSTNYVTSSYALRTVLVASYMIDIILTTVYSGGLAAILTLPTLEEAADSRQRLFDHKLIWTGTSQAWITTIDERSADPVLLGLMEHYRVYDANLISAFSHTEQMGFVVERLQFGHLGNTELIENDALKRLKLMVDDIYFAFTVAFVPRLWPHLNAYNDFILAWHSSGFDKFWEWKIAAEYMNAHRQNRIVASEKTNLDIGPVKLGIDNFIGLILLWCFGMICSLLTFLGELWRGQG

>DmelIR76b

MATGIELLVAAALCVACPPLNDSPPTNLIQMGENGTLSPVTELPMDVDASEAGFDADAPVETLETINRKKPKLREMLDWIGGKHLRIATLEDFPLSYTEVLENGTRVGHGVSFQIIDFLKKKFNFTYEVVVPQDNIIGSPSDFDRSLIEMVNSSTVDLAAAFIPSLSDQRSFVYYSTTTLDEGEWIMVMQRPRESASGSGLLAPFEFWVWILILVSLLAVGPIIYALIILRNRLTGDGQQTPYSLGHCAWFVYGALMKQGSTLSPIADSTRLLFATWWIFITILTSFYTANLTAFLTLSKFTLPYNTVNDILTKNKHFVSMRGGGVEYAIRTTNESLSMLNRMIQNNYAVFSDETNDTYNLQNYVEKNGYVFVRDRPAINIMLYRDYLYRKTVSFSDEKVHCPFAMAKEPFLKKKRTFAYPIGSNLSQLFDPELLHLVESGIVKHLSKRNLPSAEICPQDLGGTERQLRNGDLMMTYYIMLAGFATALAVFSTELMFRYVNSRQEANKWARHGIGRTPNGQSVAPSRWLRGWRRLNSGHGQLLGASTHGQNVTPPPPYQSIFNGGSHGDPLNRWRRPLANGNALGNGVLLGGDSEGGVRRLINGRDYMVFRNPNGQSQLVPVRSPSAALFQYSYTE

>DmelIR84a

MIKLQVKVISWPLIILTAFLRVLQIESINTNFLELAAFEDFLRSEHLSHVLVVRGDDADGDWKIECHQKLLANYRVQFYRPEMSANFEDLMFYGSPRTAVLVLNSEHVLVRRQVFGVASEAGYFNNSLAWFILGSGRESLPVEQLIDQLLSGYRMGIDADITVALRGPDNASMLFYDVYRISRQANTPLIIEKKGLWTHSGGYQKFGNFKNTWVIRRRNFLNVTLIGSTVLTEKPPGFGDMEYLADDKQLQQLDPMQRKTYQLFQLVERMFNLSLAISLTDKWGELLDNGSWSGVMGQVTSREADFAVCPIRFVLDRQPYVQYSAVLHTQNIHFLFRHPRRSHIKNIFFEPLSNQVWWCVLALVTGSTILLLFHVRLERMLSNMENRFSFVWFTMLETYLQQGPANEIFRLFSTRLLISLSCIFSFMLMQFYGAFIVGSLLSESARSIVNLQALYDSNLAIGMENISYNFPIFTNTSNQLVRDVYVKKICKSGEHNIMSLQQGAERIIQGRFAFHTAIDRMYRLLLELQMDEAEFCDLQEVMFNLPYDSGSVMPKGSPWREHLAHALLHFRATGLLQYNDKKWMVRRPDCSLFKTSQAEVDLEHFAPALFALALAMVASALVFLLELFLHWLPDFRRRLGTMST

>DmelIR85a

MSIQWLKHILLLAILVNLAGTRENHIPLDLKKSSIVMVKMSQILCKARIKVLFVYFENQTSHEHTGQILKEVTKCDISNQNTPLEAVKDDGILMYMVMITTNISQPLELSLIRKKSAAKHRSHVFLLVRDADTVSDAWMRASFRQFWKIWLLNIVILYWRDGRLNAYRYNPFMDNYLIPVDNKPNEVPTLEQLFPKTIPNMQRKPLRMCIYKDDVRAIFWRQGTILGTDGLLAAYVAERLNATMMITRPHSYNNHNLSSDICFLEVAKEYVDVAMNIRFLVPDTFRKQAESTVSHTRDDLCVIVPKAKTAPTFWNIFRSFGSLVWALILVSVLVANVFCYILKSEVGRVPMQLFAGALTMPMTQIPPNHSIRLFLIFWLYFGLLICSAFKGNLTSMMVFQPYLPDINQLGALARSHYHIIIRPRHVKHIQHFLTLGHKHESRIREQMLEVSDTQMYEMMRNNDIRFAYLEKYHIARFQVNSRVHMHLGRPLFHLMNSCLVPFHAVYIVPYGSPYLGFLDSLIRSSHEFGFERYWDRIMNSAFIKSGVKVVNRRRGSGNDEPVVLKLQHFHAVFALWLVGIGMACIVLAWEHLTHNYNLAVTKRRD

>DmelIR87a

MSTPEQRFWLAALLFLLSQHSEVRGFGINLMKVQTEDKGQEACILALLRKYFDSGDGLSGSVLCINRNYQLPNIEEQLLRGVNNYENYPWSLLITNSREGPSPAKFLMNEKPQCYFLIVDNLEDEDLDEVFEHWKGMVNWNPLAQFVVYLASLEETDEEMNDLMVELLLTFINKKIFNVNVIGQSEENQFYYGKTVFPYHPDNNCGNRVISVELLDACDYPSEETDSEDENDEDEGDGAQEEDDGPQEEGDGEQEEEDGPQEQEDGDQAKGDEGQENDDGGLENKVENEFRIGASDDDELENDLSSNSSEPEAIIEEFFRAKFEDKFPRDLSGCPLTASFRPWEPYIFRNSEEQPVDDYYYGLQGDEDDYNDTSPNYGESDDESYADPGEDGDGAIPDTETQSGGKLKLSGIEYEMVQTIAERLHVSIEMQGENSNLYHLFQQLIDGEIEMIVGGIDEDPSISQFVSSSIPYHQDELTWCVARAKRRHGFFNFVATFNADAGFLIGIFVVTCSLVVWLAQRVSGFQLRNLNGYFPTCLRVLGILLNQAIPAQDFPITLRQLFALSFLMGFFFSNTYQSFLISTLTTPRSSYQIHTLQEIYSNKMTVMGTSEHVRHLNKDGEIFKYIREKFQMCYNLVDCLNDAAQNEHIAVAVSRQHSFYNPRIQRDRLYCFDRRESLYVYLVTMLLPKKYHLLHQINPVIQHIIESGHMQKWARDLDMRRMIHEEITRVREDPFKALTFDQFRGAIAFSGGLLLVASCVFAFELCYVKYVYRTEKRERKTKKITKKVHNIKIQHD

>DmelIR92a

MLLQPLVMHLSQLLRIIVGQYFAEFPSILIVYNNSASTTPLQLEYLSALELVLRELSKPIRLQWINVAFLKDLNDLEDQVMGALNSSVTEGFITILSQTHHFIHARYYATRNANVRLKDKRYLFLCEDESPAELLCMDILQFYPHHLMVRPGTETAPTGPTGPHPDPRRGGGASVSTKNKDDGEGGAGNKTTSPYRDINFELWTQKFVGAVGNLDALLLDAFLPNETFANRVELYPNKLLNLQRRSLLVGSITYVPYTITNYVPAGQGDVDPIHPQWPNRSLTFDGAEANVMKTFCQVHNCHLRVEAYGADNWGGIYDNESSDGMLGDIYEQRVEMAIGCIYNWYDGITETSHTIARSSVTILGPAPAPLPSWRTNIMPFNNRAWLVLISTLVICGTFLYFMKYVSYRLRYSGTQVKFHHSRKLEKSMLDIFALFIQQPSAPLSFDRFAPRFFLATILCATITLENIYSGQLKSMLTFPFYSAPVDTIEKWAQSGWKWSAPSIIWVHTVQSSDLETEQILARNFEVHDYSYLSNVSFMPNYGFGIERLSSGSLSVGDYVSTEALENRIVLHDDLYFDYTRAVSIRGWILMPELNKHIRTCQETGLYFHWELEFIDKYMDKKKQEVLMDLANGHKVKGAPQALDVRNIAGALFVLAFGVAFAGCALVAELLIHRMDLSK

>DmelIR93a

MNPGEMRPSACLLLLAGLQLSILVPTEANDFSSFLSANASLAVVVDHEYMTVHGENILAHFEKILSDVIRENLRNGGINVKYFSWNAVRLKKDFLAAITVTDCENTWNFYKNTQETSILLIAITDSDCPRLPLNRALMVPIVENGDEFPQLILDAKVQQILNWKTAVVFVDQTILEENALLVKSIVHESITNHITPISLILYEINDSLRGQQKRVALRQALSQFAPKKHEEMRQQFLVISAFHEDIIEIAETLNMFHVGNQWMIFVLDMVARDFDAGTVTINLDEGANIAFALNETDPNCQDSLNCTISEISLALVNAISKITVEEESIYGEISDEEWEAIRFTKQEKQAEILEYMKEFLKTNAKCSSCARWRVETAITWGKSQENRKFRSTPQRDAKNRNFEFINIGYWTPVLGFVCQELAFPHIEHHFRNITMDILTVHNPPWQILTKNSNGVIVEHKGIVMEIVKELSRALNFSYYLHEASAWKEEDSLSTSAGGNESDELVGSMTFRIPYRVVEMVQGNQFFIAAVAATVEDPDQKPFNYTQPISVQKYSFITRKPDEVSRIYLFTAPFTVETWFCLMGIILLTAPTLYAINRLAPLKEMRIVGLSTVKSCFWYIFGALLQQGGMYLPTADSGRLVVGFWWIVVIVLVTTYCGNLVAFLTFPKFQPGVDYLNQLEDHKDIVQYGLRNGTFFERYVQSTTREDFKHYLERAKIYGSAQEEDIEAVKRGERINIDWRINLQLIVQRHFEREKECHFALGRESFVDEQIAMIVPAQSAYLHLVNRHIKSMFRMGFIERWHQMNLPSAGKCNGKSAQRQVTNHKVNMDDMQGCFLVLLLGFTLALLIVCGEFWYRRFRASRKRRQFTN

>DmelIR94a

MALPKQLKFINIFLVLLIIYGSSDGTENQHEIFLNRLLQAVHNERSVETLFLLHHSNLANCSLQDWNPPRIPTIRSNELTVFNVEKTFNHNALALVCLMKNSYREILNTLAKSFDCMRQERIILMIHRKSDSKFIEDITHEVKNLQFLHLIVLIVQEKYNGQVFASTLRLQSFPEPHFKRIRNVFAIQRIFYRPINFHGKVLNAIPNDIPILFVALNEMFTEYARRYNSTLRIQNRTIKEDIEITEDNYDIDMKIQLHNSQNFLHHMNIAMDIGSNSLIILVPCATELRGLDIFKELGVRTLTWLALLFYIIFVLVEMLFVFISNRFNGRNFTMRYTNPLINLRAVRAILGQTSPISNRYSLSIQHFFVFMSLFGTLFGGFFDCKLRSFLTKRPYYSQIENFSELRKSGVTVVVDHTTRQFIEQEINANFFRDEVPNVRTTTIQELINHVYSYDRKFAFVANSIPWRTFREEMKSINQKILCDSKNLTILENVPLTFSIRRNAIFSHHLRNFIINAADSGMITCWFKMAGKVIRKHIKTTLRESEQQPSHLPLSFDHFKWLWAVLCIAYVMSFMVFVMEILWSKYQRRTRSVSIV

>DmelIR94b

MSLIFNLLFILILSQAVSQETEFLQLKYLNNIVRSMIKLHKMETLVIVKHHLDNNCSLQNWNAHGMGIIRTNDQGKLIMKDTFNSRTLAIICIGQNSHITLLRNVFETFGKVQQKKIILWTQMELKEKFFQEISKKSRDLKLLNLLVLKAVTKDKLLIYRLNPFPSPHFKRIENIWTPNDTLFMDTKFNFHGMTAVVKHDYNWTIQMGNIRKFPISRIEDKEVIEFALKYNLTLQFFNDVERFDIELRKRIILKSNSTQPIDSGIPMVFSSLLIVVPCGNYLSIQDVIKVSGIEKWIFYIILVYVIFVLIEITFLGVTILISRQSRHQMIPNTLVNLCAFRAILGLPFPETRRTSLSLRQLFLAIALFGMIFSIFINCKLSSMLTNPCPRPQVNNFEELKTSGLTVVMDHDAENFIEKEIGVDFFNQYMPRKVTLTFTERAKLLFSLKGNHAFTLFSESFAIIESYQRSKGLRAHCTSEDLIVAERVPRIYILENNSILDRPLRRFIRQMQESGITNHWLKNIPSSLEKNLMQITIPYDRERVHPLSIEHLTWLWCILILGYSISMIVFFVEMSLKRRKKNLENRAPNICIC

>DmelIR94c

MSKVFKLLVLPLIYLSLTKGSKNPQLKFLRELINVIEEGREIRTIMVIKHSRDEYCHLDQWNPRGSPILRTNEMGSIRISGYFNDQAVILACMGENSDYGLLKSLANAMDNMRQERIILWSEREPTKMLMDYISQQADRYNFAQIIIVTMNEDVDAVPSLHQLNPYPTPRFRQITNISNIRRTSFFGCGLSFQGKTAILKESVVSNIRFKVWSPSGPIPLSELKDYEIVQFAVKYNLSLKLYDQNESKSDHFDIQLGPLFITKDFPTQMAFVSPNTACSLIVIVPCSPKWRFMDVLHKLGVLKLIGCLLIAYAVFVLIETLILWLTHRISGREVRLTSLNQLLNPRAFRGILGLPFPEFRRSSISLRQLFLVISVFGLVYSNFVSCTLSALLTKPAQNPQVRNFKELRDSGLITIMDKYTHSFIEKHIDPEFFDHVLPHYLILQKKEALRMIWNFNDSYSYVMYTTTWKSLNTVQKSFDERVFCESESLTIAWNLPRMYVLGNNSVLKWMLSRYITYMPQTGIPDSWTEQLPKVLKLLYNVTSPRRIKEGAVPLSIQHLSWIWHLLFIGESIATLVFIVEILLQKSNQHTSNMRERSSEDDDFV

>DmelIR94d

MGQLHLLLVALVLLSPGGDSFYHSLIHHLNRELKIEYVLLLGNFDTTWLDILWQLPVSVLQIKEHSRETYSLLENPSHNVLTIAFVNDSPEDILEILYRNLRMLNTQPVLLVIRKSTIRVNSLLEWCWHHQLLKVVAIAQDFMESLIVYSYNPFPVLQFIERRLDNSTVIFEKRLENLHGYEVPIALGGSSPRLIVYRDLEGKLIFSGPVGNFMKSFEQRYNCRLVQPYPFDESAISPARDLIASVQNGSVQIALGAIYPQVPYTGYSYPIELMSWCLMMPVPEEVPHSQLYSMVFSPMAFGITIVAMVLISLTLSMALRLHGYRVSFSEYFLHDSCLRGVLSQSFYEVLRAPALIKAMYLVICLLGLLITSWYNSYFSTFVTSAPRFPQLTSYESIRHSNIKIVIWKPEYEMLLFFSENMEKYSSIFQLQEDYKEFLHLRDSFDTRYGYMMPMEKWSLMKEQQRVFSSPLFSLQDDLCVFHTVPIVFPMVKNSIFKEPFDRLILDVTATGLLSRWRDMSFTEMIKAGQLGLEDRGHPKEFRAMKVGDLIQIWRFVGWMLGLATIVFLLELICFWRHKMWQNMKYMFCRNKNI

>DmelIR94e

MDCPKWILSGLCLISLVSGATVIELLGTLKLELDFEYVLLMKNRNFSLSDQVWNGTSLTKDVMDEVQVPVLQFNENVSYFLHNSISRRLVTLGFMSDANLDEHRGLLTALVANLRHMTTSRVIFLVQSKASTDFLYELFRNCWRKKLLNVIVIFQDFETTSTFYSYSNFPILQIEERIYETSLQTLPIFPDRLRNLHGYEMPVILGGTAPRMIAYRNKKGNVVYDGTVGHFMTAFQQKYNVKFVQPLQAKNPLDFAPSMQTVGAVRNETVEISISLTFPTIPPFGFSYPYEQMNWCVMLPVEADVPPFEYYTRVFELAAFLLTLGTLVLISCLLASALSLHGYATNISEFLLHDSCLRGVLGQSFVEVFRAPTLVRGIYLEICVLGILITAWYNSYFSSYVTSAPKQPPFRTYDDILASKLKVVAWKPEYAELVGRLLEFRKYETMFLVEPDFNRYLALRDTLDTRYGYMITTNRWVLINEQQKVFSRPLFQKRDDFCFFNNIPFGFPLHENSVFMEPVQKLIMELAETGLYYHWITTGFSELIDAGEMHFVDLSPHREFRAMQIQDLQYVWYGYAFMVVLSSLVWLLENLAYTVKSKTIFPTHFMQRNKK

>DmelIR94f

MWQQVLLAETSNWFRSDVLQRFWTHLRVEIRFRTMLNYRLESCDCWFDNVLGSDNSTALLWNDQTYPHYLRRRQDTDILVVSCLRFHQYQEVLLALSLMLDQMRSMPVVLQLCGDEDSMQELNSARLLLKHSQDLKMPNVVLLSSTFFTSATLYSYEMFPEFNVQKLVYQAYLTLFPYKLGNLKGHPIRTVPDNSEPLTIVRKTLNGSIAIDGLVWQFMIEFAKHINATLQLPIEPHPEKSIKLVQILDLVRNQTVDIAASLRPYSLNVQRSSTHIYGSPMMVGNWCMMLPTERVIGSHEALTRLMKSPWTWLILLLFYSVHRFLAQKTRLRSSLIHLIKLLINLSLICFLQAQLSAYFIGPQKVNHISNMQQVEESGLKIRGMRGEFMEYPIDMRSRYASSFLLHDLFFDLAQYRNSLNTSYGYTVTSVKWELYKEAQRHFRRPLFRYSEEICVQKLSLFSLIQQSNCIYCYRSRIFILRMHEAGLIRLWYRRSYYVMVTAGRFPIGDLSTVHRAQPIRWTEWQNVVLLHGVGLLFSVVVFVIELTVHYANVCLNNL

>DmelIR94g

MSTAVNSVHSKLVSLISRGQELTSIFFYAPAKEKCHLEDTISSATWGLPLVIWRTDRTVILNGFIGEGLLVLACLPGFHWRALLGSLARSLKYLRQARILIELMQDRDEFLVSEVLQFCLSQDMINVNAIFDDFPETENLSSFEAYPSFEVVNQTFTPDTQVSDLYPNKMLNLRGGVIRTMPDYSEPNTILYQDKEGNKEILGYLWDLLEAYAHKHNAQLQVVNKYADDRPLNFIELLDAAQSGIIDVGASIQPMSMGSLSRMHEMSYPVNQASWCTMLPVERQLHVSELLTRVIPYPTLALLLLLWIFYEVLRGRWRRHSRLQSIGWLVLATLVSSNYVGKLLNLFTDPPSLPPVNSLAALMESPVRIISIRSEYSAIEFTQRTKYSAAFHLALHASILIGLRNAFNTSYGYTITSEKWKIYEEQQKRSSKPVFRYSKDLCFYEMIPFGLVIPENSPHRAPLHSYTLLLRQAGLHDFWVNRGFSYMVKAGKINFTAVGERYEAKTLTITDLRNVFIIYVSVLLISLILFTCELFVSWVNYWLGF

>DmelIR94h

MLSNISFSSAPELVDLYGLVLKFLVSSETTLFYFNPTGQKCSWETLPRTILSNHPQIIWFREETYPGLYKRHSSNLFVMACLSSTSYDGQLQLLAESLTRYRSVRVLIEVQDKEGSFLASQILLLCQQHSMLNVVLYFSRWTRTLNVFSYLAFPYFKLLKQRLSGSLRPKIFINQLKDLQGYKIRVQPDLSPPNSFSYRDRHGECQVGGFLWRIVENFSKSLKGDTQVLYPTWAKAKVSAAEYMIQFTRNGSSDIGVTTTMITFKHEERYRDYSYPMYDISWCTMLPVEKPLSVEILFSHVLSPGSALLLILAFILFFLIVPQLIKCLGITFRGRLIGMASRIFALVMLCSSSAQLLSLLMSPPLHTRIKSFDDLLTSGLKIFGIRSELYFLDGGFRAKYASAFHLTENPNELYDNRNYFNTSWAYTITSVKWNVIEAQQRHFAHPVFRYSTDLCFSSETPWGLLIAPESFYREPLQHFTLKINQAGLITQWMTQSFHEMVRAGRMTIKDYSRTNLMKPLRIQDLRKCWVIFAVGLGTSTVVFTIELLLIYTNVFLNSL

>DmelIR100a

MATTLQLIMLALVGGTLGQANNTDHKQVLTSIVKQLEGGLELHLRTSEDGGNDLVQFLMQEKSSIIISAKQEEVPSRAKIMRHHFFIFDGVHQMQEIRTSLFNTDGFYILALENNTIEDDVLLMEFAADVWLQHGHSRIYYVQLSKKSVLLFNPFLQRLVVVQDSKTYSRIYKDLEGYHLRIYIFDSVYSSVIGDGENKVLSVTGADAKLAKTVARQLNFTADFVWPDDEFFGGRLANGEYSGGVGRAHRGEVDIIFAGFFIKDYLTTHIQFSAAVYMDELCLYVKKAQRIPQSILPLFAVHMDVWLCFLLVGLLGALVWLILRAVNLILGIEGVPDGSRATRISYFGAARRIFVDTWVIWVRVNVGRFPPFHSERIFVASLCLVSVIFGALLESSLATVYIRPLYYRDVNTLRELDESGQPIYIKHPAFKDDLFYGHNSEVYRRLDAKMMLVAEGEERLIEMVSKRGGFAGVTRSASLQLSDIRYVMTKKVHKIPECPKNYHIAYVLPRPSPYLEEVNRIVLRLVAGGIVGLWTGEAKERAKWSIQRFPEYLAELDVGRWKVLTLSDVQLAFYALTIGCLLSAIVCMAEILLGRQRRLHSPK

>LmigIR1

NASWRFTVDAEHEWGEIWDNNGSGNGILGAVVRDAADAGFGALYQWLHEYLFLDFSRPYIRTGITCLAPRPRPGWQVPLLRPSPPLWAAVTASVLLATVALFAARRSSDRLLGEDAVRAAAGSRYSTVEDCFFRSVGLLVLQTPDVERRHTRVVGPTRHVLSWLLIAYLLVTASYGSGLSSVLTVPKYEPPIDSVRDLYESGIEWAATHEAWVFSLREATQPVISDLYRRFRVHKEDVLHARTVARDLAYSIERLPAGYFAIGSYIDEASRWLRPMREDIYWERSNVVVRKGWPHLPRLDALIDRLLDSGLLLAWEGQV

>LmigIR2

VSSIFALRSIEIIPDILVARQLDDVDSFELVTMKFTGEETWRDELVLARWRRGHGFDPPTVDLFPDRMADMEGRQLVFATIDYPPYVILKSDSNILDGVESRILLEKLFVVGWQKNATWRVVDHAVDRWGTIWENGSGNGLLGAVATGAADAGFAAVYRWFPEFLWVDYSRPFLRAGIACLAPRPPEVPLLPFAPPLWAAVAASVLLATIALYTARITSNWVLGDVQATGRYSTVEDCFFRSVGLLVLQTPDLERRHTRVVGPTRHVLSWLLIAYLLVTASYGSGLSSVLTVPRYDRPIDTVSDLHESGLEWAERHLVYLYSIRELTDQIYVDLIDLFRVLTSDVLHSRTTTRDLAFVIERLPGGYFTIGDYIDEEAASRWLRPMREDIYWEYVVFAVPKGWAYLQRLDDLIDRLLQAGIMYAWEGQVAHKWLVPRVQLAAQIGMRSYAQSPDGPIQLQLTHVQGEFALLGLGLCLALVVLLVEVAV

>LmigIR3

FELVTMKFTGEETWRDELVLARWRRGHGFDPPTVDLFPDRMADMEGRQLVFATIDYPPYVILKSDSNILDGVESRILLEFVSKKNATWRVVEHAVDRWGTIWENGSGNGLLGAVAAGAADAGFAAVYRWFPEFLWVDYSRPFLRAGIACLAPRPPDIKGGFEPPTAYSLTLRIIAPQCTRPLPRAPCPPWSDMSVQYTISSSYSGRYSTVEDCFFRSVGLLVLQTPDLERRHTRVVGPTRHVLSWLLIAYLLVTASYGSGLSSVLTVPRYDRPIDTVSDLHESGLEWAERHLVYLYSIRELTDQIYVDLIDRFRVLTSDVLHSRTTTRDLAFVIERLPGGYFTIGDYIDEEAASRWLRPMREDIYWEYVVFAVPKGWAYLQRLDDLIDRLLQAGIMYAWEGQVARKWLVPRVQLAAQIGMRSYAQSPDGPIQLQLTHVQGEFALLGLGLCLALVALLVEVAV

>LmigIR4

EEDKWGAIWENGSGNGLLGAVAMDDADAGYAAVYHWYPEYHFVEYTRPYVRAGLTCMAPRPRPQPGWQRSVDMLDNRDFVLVCYIITRCLLHVVPMNRVIGWIESSLDACMVLDTRLLTQRRCLLSSPDSVVLWGHRLQYLHLHVGLYHIKYYSHLVTIWSSLGVTLSALFGGRLRNLLCLRRYEPPIDSVVDLRDSGLEWAETHIAFLNSLRGRTEQVYLDLVKQYRVLSVDVLKSRTTTRDLAFIIERLPGGYFTIGDYIDEEAASRYLRPMREDIYWEYVVLAVQKGWPYAQQLDELIDKLFEAGIMHAWEGQV

>LmigIR6

MRSLLIVTMLVNSMCDGRLVLQNNDVSSIDSLIRELGSRILSKEFGSYKCVAALSDVPEAVAYFTQWFPTTVLISLVSGDVSAEDLLVSALDADCQGFLVRCRDAAAGVDAILKASKFAMRRVNRRLLVLPTAQSPHINITAIFDLRNIDLVPEIVVARPSGDNSFDLVTLKLTGEDTWRDELLVAQWYRGRGLQPPSADLYAHRLADLGGRRIVISTVDYPPYVIFNSGDNVFDGIEARIIIEFIKKINVTWSVVEDTTYKWGTVWCNGSGNGMLGAVAADEVDVAFSALYRWYPEFMEYSRQYVRSSVVCLAPRPLMLAGWQVPVLPFSPAMWAAVGFSVVVATAALYAVKKLSDWVLGSDDDPSGGRYSTVEDCFFRSVGLLVLQTPDVERRHTRVVGPTRHVLSWLLITYLLVTSSYGGGLSSVLTIPRYEPPIDTVTDLHDSGLEWAQTDIAYLNSLRGLTDPVYLDLIDRYRVLTPDMLRSRVTTRDLAFAVEKLPGGYFAIGDYIDEEAVSNWLRPMREDIYWGTVVLATRKCWPHLERLNDIIDRLFEAGIIRAWEGTVAREWLVARVQLAAQVGMRSAQAPDGPIKLRLTHVQGGFALLILGLCLSLLLFFLEIFVYWKSNGSTRLEFSSTIKVTLEHYRS

>LmigIR7

MLAWVAVTSFFLWPDVVARHQPAVVTNSSLEDCLAYVFSSQPFTWAPLLLVTTTSPDDREPDVSAITNAVVHTVQLPVVLVKLPTTSVYADLVAGYIIVLPDSDLDQDTFQSILVNISYVANWSPRRPVLVIFRAGYGRHEDSVRMILDCLDKRNAIEATVFVVNVERTSWEVFGYYTVRNISAYTWFPYRQGHCRDDGVETELIGNIATTGRHNFTVFPEKLPRSLHGCSVVAATYHLPPFVSKVESEGRQTSFSGYDVILFRLLARAANFRPVVSAPHFSGPQWGERLENGSWSGVVGRMLRRQADAAFGGLHDNAXXXXXXXXXXXXXXXXXLPDALTWFAPRARRACALCEFGLIVPARDAGLLLLGALLMSVPFKLFSGQDEISALRRLDAAFLFALRMLVTTPARAPRTLRPRIVFIFWLQLTYILITGYQSTVTSKMVQPGTEEQILTLEQLLRSDLYCGTLLPFQRWMDTVMKSKAPKISYLCPNIADCMFRMAYSKDLAVMTTKVHTEFMGKVKYVDQAGRSLFLPFTENLFTYYVAVHVQQGSPLLPLFDDVILRVQAGALMTPAMREYERLFRYGTVDHSDLPEEVDSQGFIALSHLSGAFTAWAVALATAFCVFVAELAYWRSGFRLFARQLRCSRLPEKEKDKNAFGSDNFITEKKGRKLRL

>LmigIR8

LCGKATLADVTRPAQSIIQTVAPVTTEKAAAPLQEPHVCLASPQIPIRCGCTYGTAICIVEARKPPHLGNVCGSWEWDTAVKFHKVNKRLNIFEQTNLHWTVDKRRKTRNERCSLSLLSRKNSGLTSLETFANTRRISAAEVWEMTSRIFFHETINSDRIVDWPPRYADLNPCDLYLWGMLKELACREMNFSSGTVLTYDPFLVKDSIWSDSYTPKLQEVKETADIYYKKYMNLNGNNVRASMFTVNPSAIEDNTTVSKFRGCDAQMIETLAKYMNATLVMLPNDGSGFGKWNGTVNTGTDGDVMFDRADIAPNTRYVIVERLKVHGYTYPHDKEDLCILVNKSPRIPQYLNIILPFALIAWLTILLSLPFSALFWSLIRRFGARPSNSEPFARVYIGSFLKIFSAFLSVAVSALPTVGRERILFVMWTFFSLIITNTYQGSLTSYLTIPKYMPDIDTMEELSKSGLKILIHPELLPVFKLDTGNPVMDALNRNLVSDIDMEGYPEKIQSNTDTCALVNAYVGQFLIRSRHYVINGFPLLHLTNECPMPAVVAFATPKFSPLQPRFDVLIRRIVEAGLYKKWQKNMLDESIASGDLLLISNREGKADPERITLSHLQMPFYLLFLGYFLTSVLFLTEYLRLKMNTRSTNEEEAKLERREGCQTLENDSASYLLDV

>LmigIR8a

MWPLWMSVVAAHLQLASSQATSEPLLIRFLLVTEVNASWVGGELRANLSGLEARYVGLRLQLDLSAVEVDREHEVEEFQQKVCGALASGVSALLDATWTGWRRLRDEAQHRGLPYLRLDATLANFVDAVDKYLHAREASDAALIFHTEEELDQALYHLIGNSVLRVIALNGLEKDTVSRLKDMRPVASYFVIFADTAHMAELYSKAAAGGLVRKAERWALAFTDWDWRSFRTDQLNLSTALLQMKPASCCALQGEPDTCKCALRKVAPAFLRAALSAAVDTLAELHSKGMDVRATPRQCSSTGDGGADDEDGDGAGEEEESAEPPANATGNYDAFLRAIATRGQSNSTLFFRAATAQLTFNTPLQLRMVNRSEDVSLGDWSPEKGLQLERQLKPAKRFFRVGTAEGVPWSFPVRDEKTGEPLVGPDGEPIWDGYCIDLLKKLAEPTHMNFDYELVPAKDNDFGSRSPSGSWTGLVGDLAMGETDMIIAPLTMTSEREEMIDFVAPYFDQSGISIVIRKPVRETSLFKFMTVLRLEVWLSIVGALTVTGIMIWLLDKYSPYSAQNNKEMYPYPCREFTLKESFWFALTSFTPQGGGEAPKALSGRTLVAAYWLFVVLMLATFTANLAAFLTVERMKSPVQSLEQLARQSRINYTVVLNSDTHEYFRNMKNAEDVLYNVWKDITLNSSSDQSRYRVWDYPIKEQYGHILQAIDQAGPVPNASVGFQKVIDQEEGKFAFIHDAAQIRYEVSKNCNLTEVGEMFAEQPYAIAVQQGSHLQEEISRQILDLQKDRYFESLTAKFWNNSAKGTCPNSDDSEGITLESLGGVFIATLFGLALAMITLAGEIFYYKRKKLTAVNVTSSSAKVPKKQVTIGKEFRPVMDKTAPRVSYISVFPRNQLY

>LmigIR9

MQLHPSPCENWTEPHVWIHYPYRKENPMLRVPASQFVVVETLATTHRYWNLEGAHISAVMYPAYPTAMPAGQREINTKTCDGDTTGYIELHANSVRIFKYVRGSDHFLRLTLEEKLNARVVARYPKDENFVEGEELPNGQLTGALAEMASGQAQLGLNLRFVRPLPTPEILYLQPVCSFARVGIAVARAPLAPLHVAIMRSFDLKVWFSVAAFYILSAMIWSLLTSSINYKSVLQEIQKFLLIGCGSFAQSPSQKFFASACAIFSLIIVTIFQEKEKERKGEEEIRKELGKTNGSLVNVLTTPVYDRDINTANELLQSGLPIATNRTSLIHMFQLAAEGSPVMTELCKHMKPTDKHNLTELLFDRKQALIIADAGLAQLKNHHPSQEKMLHMIKDTFIVLQRAYAARTDCPVKEQVEKIIWCLTEAGIPRYWNTQASRSEFSNCPLPEDTPKPFSLKQFKIAFYTLCIGLLFSSFVFLLEIISYKRCQKHILK

>LmigIR10

LDVRLLASTHGLCRSAVADVATSILRSHHPRPAVVSTGPTYLPPATTFLTPLQNEQRLLVTDSASPVVEFFQFGRAHSRLWSATNDFIILLLKAYDHDGGDCNADCANLLRHLWSAFGAARVLVRHVEVSDGCHVHRLGPTVALFDPFDTSAGTIVSTTWTNVELIKTLLDNRTRDLRGRPFRVCMFARNYTALPVDANGVLVYRSSLETVKYAGVDGWALQTLAQYMNFTTVFRQPYDRERYGELLPNGSFTGALGELMRGRVEIIFNAIFMKSYGSHEIYFTKGVSKDDIRLVVPKGGLLSKRHLLTMLMRPEVQLSILVTYLLMTLLWYASSRLHMFFKPEDAERPDILMIAFQNFHPPTSSALEKLPRHAAERFLVVSYLLFHMLVFTIVQGKMLEVINNPRYEDDINTLQELVASHLPIKTGSATLIDIFGPTGSAPQPESLLQSLSRKTNENHQKGGRKPDGNLRFNVMIFRAYVSASFVALLLSTGHRHSSVAPMAFTFTVLGNECIKELDDVIDNMVLLEKGSAVEEVARSRDAACVVRDSDLRHGKYSAYRRADGSWLLHAVQEAALSFELGYATRRDSPFLAAIDRFLWRAVEAGLSRCWERRGYLLVTLRARADPASIDTAAAAAKTYKQFTVSDLQMPFYAWIVGVASSVICFAAELLYSWWLSGHKN

>LmigIR11

MSACEVTIGADRIGVLTFAQPVRNAGCRFYFQTQFQEVMFKPYVQPFEDRVWIALLGYVVATALLKWLFHYVLRERADPITALFAVIAALLNQASGDPRRCAVRVLYFSLCALFLILRIGYGARLTATSTLRQAAPPYRRMEDILASDWGVNVISNSFALESMQMSPPNSTAWKLWQTKIDNNPYSTVETTEDGLWNVLDKKKLAFFGFEDACRDVLHRRFSPDQSCRISELDGVFFKAPLSFALPRDSEFLLTINYWILRMFETGIIDRLSRKWLPKPPVCDETKYEAETFADVLPMVILFASGFCLAIIVLIGEICVYSFKNRNMKVVSKNRKVRKTQKNYLYPML

>LmigIR12

LQPEVSSHSTDPSGKKFDGHRFKKDTNVNRCRGGVAKRIKALCSDERGHSPLGDYMAFETAWQIKIVCRNRDSNLGPLPFAGKRTTDCAIPTRLTTPPHNFTSASTSPPSSRSASLVTSSALPKNWYEISGKCLSCVHATVSQTYQKLDKVLRHVIATARYEVSTYSGLVPGNQKEDKSWTGVIGLLQQGSAEIITDLLTVTASRVNAVDFSSPATTDKFGLFIKDEVYTDVNRWSFVSPFETTLWMAVIGTILLYMLCVTVMNLASYDKNPQCSTKEILMGIFAAFCLRGYSLLLPRWSLRLAYLSAFITAVVIHAAYCARVVSHLANSNRSLPFTDLEEAYAAGYEIQVVPGTSAAETFKYASDGIIRTINEEMIEPRYFYLPVTINEGLLHMCNWKKTCFVSERNSVRCSQKQPCGIIEVSVTMPSVYLAFALRKKSPFRRIISYQMEKLRTGGILKRLKSTSCNRAQADQGSDFKRVQLKNAAPLLAIIYFAVIISFLVVILERALFRYTQKTNSKSVPKFRKYRKRIRQTPMYLP

>LmigIR13

MDRMCDCCVRAQEELAAVREQLSVLLAAVSRLKALGGSGASHGTPQVVLASPTIPAVGTSSRVPDAVESPSPQGVWRVQRRSRRTGRKVNVEAGRAATPALPNKEFCTKSIWLVFLRGDEILEKAFEDMYIPLNCQLLVVHQYGQQHFITEIFHLKEKLHLRKVLYGIWSAEHDLQKTADGFYSRRCDLLGETVKVSTFKSYNSEYGLSQLRLLSETLNFTYKFVKVKEMFPGRLTASGYSGMLGAITRREVDMTIDLLTHTTARSHVVDFLFPTQKDTHSMFIKLSLDEGIPWTSYLSPFCGRLWGTVVLIITLDAVALAVLLRSSGHKVWTSDFLHLLLDVLGMYSLQGLKGSTGTDRPPCHPPPEVVIGYEIAKDLEEQLNGMENVLKGTHEMNINKSKTRVMECSLTKSGDAQEIRLGVSVGSRTSAAQLVSVSAYVTAIVLQAAYCANVVSVVASRRYSPPFSDLAGLLADRTYRVAVMRESITNDVFEFAGDKEMKLVYQHFIEPHNNDMPVLEEDIDVILCHRYRYCFTGESKLVDSHTVSCEIFEVRLKTPPSHLGFALWKGHPYKNIFNYRYTIRIARLADVTRPAQLIVQTVAPVTTEKAAAPLQEPHICLSSPQIPLCCGFTYGTPICILEARKPPHLGNVFDYDPALVPFLTDRLTRDENSTNGIEDDTSNGEGD

>LmigIR14

MRRGAININGDYTGKKVELAETSSKMGLDVLAFSDVRVRGEKEDEVGEYNVYLSGVKAGRAQWGVGFYIRKAIEPSIIAIRPFFTWQELLKTRQSEFMWLLLLRRDQSPSAVLQDIDIRFDTLFFVAVPTTESDVHIVEAYRVAKGYPLVMQTFCTWNTTYHPSCRTESTIARRGDLMGYKMKSGIVEHSPMVQEGLSNLMRPPRHELASRYQFKEMSGWGIRSRDGSWSGLIGRLVSGNLDVGVAYKLYIQTPSEQEFIWGHFLWPFDKWLWLCSFALVVTTWLVLRVQERFTRDDESEISARGTYNDFLQVLGIFLMQGVYGGRTAPRACVRASLCAASLLALLLYTAYCGALTASLASRRPRLPFRDMAGLLRDASYSLVTIADSSILNVLSAERQAEDEALRGGSDECGPQMEHRQPPVEREILNPVHSESSLRSHLMKDSNNPVDRKIYEKHIEGKHMPHTSEELFGWLCSSQKHAMLVSKTRYMHGTDFVQCPVTSVHETSGSQEGFVLTKKSPYREAINYHHDCHRDGEASGSDPPLSSKPMDPINPGNYATNQQLCLYPVCEGSSLHQFRQLPIGTEDVLRTQATSIIDADVSHDLQSTAPHNLDSRQQDRIHISDEAPRQTH

>LmigIR15

MFIGFEQYASCKIPIKTVAAVVSSLQDEIHFGCVYLTYSSREAELLPQSRFHVEFRRGLRNSIQLGFLPDTHETQLRHNCSSSPLLVVPHTDLLLNHSSVIKDLLRKRQSDFMWLLLLNDGNMDAALADIYVEFDSNFFVIFESTDGTVVIVEAYRVAKDFPLVTKKFCTWQINTNLTCTKEGIRSRRNDLMGYELKTGVVEVWPDMYDLGNGSCSGYLCDIWNILSRKLNFTKLAKHNGHTIIRKPLNVSNTVAIEEEEKEARQRKINVWIHSTWITKPVQKEFRTLFPHLMEPYGPAAVAELSAARQSQSARPLQSWVRMRPRAWLFVPSLLLVSSKQTRWVKDGVSQQRVGFESGELMLLFLRMQNSQCGDHLLRLLQENLFGVRYRLQRSSLFGSRLSDGSWSGAVGLLAGGEAEVALSVMMITPSRLSAVDFSLPFFSTREYLYIREPEEEDLAWDGFLRPFDGPLCVCTVAVVLVIWGVLKVHHRCAPSAGTQLDEGRLYSDFLHVIGIFCMQGCASASRDSARLSLLVASMCALVVYTAYCGALTASLATHQPRLPFTDLKGLLQDGSYQLLLLDGSGEMTILAIWPSCAFVWSPSHIGVLVNEHVNRLAKQATTTPALEIGFTESNLKLALHLKESEDPVTKEIYNNMVVKDDLPEDSNEAFDRLCSSEKQVLLCQLMDFRLNENSLKCNVTTTQEYITSQLAIAFRKRSPYREIINYQLTNYEWGAQPSVVRTSVLAVCVSASEYEAPVWDASAHTKQVDVSVKETLRIVTGCLRPPPTGRRRHQGLKEACGESALPYRTVARWVKAFNEDRQTVAATHRAGLPSAPEEDCILLPRWRTVIDARRFVNSPMKPD

>LmigIR17

MFFYLLIRDVATGGIREVYRFYVGHAVLYFNPYPLSKNSTQMDGSQLTVNEDGVINDVFPAGELWKFIAKRLNISTQLIIKKDPSCGSYDHGQWSGMVGDVHEGKAHVGLSLFTMSASRASVVSFTTPIFLGRYFIFIRSDEITHVGPNPLELLVPLKHDLWLAVIFTLFAYTTVMAGIFYIQRKTLCDAVFTIIGAFVQKSGKNMAADRIIRFGPLDGITSYHQRITSEQCLRFAAYVTGSVLLSAYSAGIISILTITEPKLPFQNFAEIEEDGSYMLGVTDNSFEYDFFKLSQQKLLQKLFNHQMDIHNLPRSKPEGLSRVCTGTKYAFLSTDLSVQSSWSQQNCNVIAVPIDMFRCTLALILRKNSPYIGGMNFHIQILRGAGLLNHMLILYNIRDTSFHQLYAERNIMELQNIFPIICVLVLGIIFSFMCLIME

>LmigIR18

FADSVNSVWLEKNLAKQRKALTFAAHNWTSGVRRCPRNKVLTIADLSYVATPGVFTKGVRRNVTWLVAANGGEWRRQLERCYVPLDALLLVAEALSAGQLVEITEVFSPRRGRPPTFAFFGRWTPATGLYTKRDDVTALYRRRTSLDGAQLAVITYHDPPSVTVTQNETHTTVGGYFGEVWNTLAKHLRFRTQWLVQEWPNPGYNRADGTWDGAAGALLKGEADVALVATTMRPYQGTPIAFSYPLYYSSRSDDIEGRPGVNSSYSASGAGRDRLFVRRMDSPRSSWGDFVAPFERRLWVAVALSVPALAAALAALYRLGRHFGTADATGPYDYSFYDSLLYVFGAFCQQAIITFAAYSAALISSLTVVSDDLPFSDIEGLLRDGTYTVGVLNRTETFHGLMSPPGNTNGGLFYLRNIFTREDALIIYYTVELHVLGILPYPIKGSNIPRSNLLLPLFRNHTFVYPLHKHPSVLVVPTRFIALEVKSSKVFKVLKTRENQVGRHAKTATWSNKEMVSLLMPKHVLIFLALLVNIEVSLYEGC

>LmigIR19

MCINKPPDGELSYKIHIYDIGKESDYKYYARDTVVRTDGNFQMDMNGNRELFDKSILQLLQDILEFSLLCVEYHEIKIYLIKLGHIQYGRVRRLKVSNHNGKKYSRDLSILRQLATDKIDIGSTTILLTYDRIGMADYTIGIDTFNSSYVFVKNPAAENSPVTGINASRKQFSLNMLEQPSWDLSEVMLVTIGALSQQGADRHPNTMAARILFLVLFLLAVLMYTAYSASVISIMSSTKPVSNSLQGILESSSKMSVALHDIHYYHTQFQIERNPLSQNLHKLELVPEFLSLEEGLEGALEGNIAFSCGRVDAHTYLQNSQHTDESLCGLGEIPILRGMGYQRSFALKHNSPLRRAFDRGLLRLMEHGLIKREWHRYFGKRPASELHCEETSSSTGFVRITLDDVWPAVKMFGVGVIIAILLLPTELLVRQWLVLLEGNNLCENVLRSKMLTPIIK

>LmigIR20

MPSLCKASVIMTVLTLCLTSTIFCESQKSYGNSINFILDFIRHTTTASSIKGVHAFVCWDAGDLQLLKALSRNGMLASVYSGWQHWEQLPTFHMDGNSLLFILDLKCEKSFAFLKKTGRNVEFFKPPHMWLILHDAYHPDTQLFTDHSAETMLPAAKNNGKNVLKQQHNVSSYNNSIILDAMELGDEDFSSADVYDLSYGTSPKRRAGYTNSNSTHVTHVPSGVGMKNSEFTSNHINDNSAVVEAAETGFYCEVFRGLDILVDSQVTVGRRDADSKYTLLEAYRRRRQGELVVSELGYWEATAGIVWRNVREVSVRRLDLKRTKLVASIVVTNPETLDHLDDIHNRHIDTVTKLNYVLLLHVADVLNASLELLVTDEWGYESNGSWSGLVGSLQRAEADVGGTALFVTADRMRLIDYIALTTPSVAAFVFRQPPLSLVSNLFTLPYTVRQS

>LmigIR21

LSLVSNLFTLPFSRAVWACAAALVVTCTVLLLAATRWERTRGDPQLADYLSQFYAVAGQLRDKWGEVAMLAVGAVCQQGSPAESRGVPGRIVTLSLLVTVMFLYTSYSASIVVLLQSTTSSIRTLADLLYSPLGLGVHDIVYNRHFFPAADDPVRRALYRQKVAPPGAEPRFMTLEEGVRRMRTEPFAFHSELSPAWQLVQETFREDEKCGLQAIPFLQLMHPYIAVQRGSAYKEMFKIAYRRLWESGLQHRQLSRLYTMRKPRCAAGRGSSFVSVGIADCYPALLVPVYGVAVAIIVVLAEILFHRRVEVLRW

>LmigIR22

MDEGHQADCVFLDFKKAFDTVPHCRLLTKIRAYGIGSQLCDWLEDVLSNRTQPAFMFREPSLAAVGNVYTRPFSRGVWLSYSLAAMLLALLVVGSQRLLASARGLLDDMTDPAVAPPWADVPLVGFSIICEEGASLVWGYELLSAFSAVHSFFNKHTVYYNPLSIFVFEIFDFEIRESFSGHPVFSTRELDQFLYQRTSKTLQEINNFFSSMILSVHSRRSPIEFPEHFRDNRALVEPTSNEPSCSPLNCFYISSKRDRKYHKNNWMYFKVRVNPYCEGYRDTFNINARPTPWRWSGIRPPPQNVSSRILLMFLLVLAVFHVTAYSACIVSLLQLPSGSINDLHSLFGSKLRVVMQNLPYNFNYGNTHLVTPLCLAVKETKDPLTRSFYQERVYPQPYGKVFKPLEDCVALMRGGRFACHADEAAYKVIGDTFLEAEKCSLKSVPMFPLRAIILGVRKHSQYKETLSVIQAWLRETGLLKREWTRWVAQKPRCLNRDSGYAEVGLTEVSPALLMLCYGVAGSLVALLLELLLHRAIAARPGRHQRQPKTPARGNALVNRAFLR

>LmigIR24

MKPPAALLVLLLVCQESSGQDSYFTDVISLTRDYFVAKRVSLVTVYTCWNSWLEKDLLRSLWDRGLRASRLPLAEAAETSCGGSHGAALASLEAAGPYRSGVLVDVACPGGQQLLTKASARRMFGIQRHWLLVDSSGNSTAGDEPEFPALPARWRDVLSSLWMMPDSEVVWMGAADDGAIQLLDVYRLTSFTPVLNISLAGWAVRRNSGVSLYLLPRPDTSKRRNSLHGAKLKAGAAILFPKYFTGMYDLRLPHLDTWTKITYPLIEYLGQNFNFTMEVFYTDSYGWQTNGTFDGVIGMMQREEIQIAASSLFMRRDRMPYVDFAAEAFYLKTAVVFRQPTLASVANIFTLPFSAAVWACCLLLCVLTLLLFGVQLRLAAKRGIEGELTHVTYAELFTFVLGSICQQGLPQTPTSLSGRVTVFVLALTSLFLFTSYSANIVALLQSPSHSIRTVSDLASSPLTLGVQDIAYNKVYLGETTDRELRQFVRRKMQPLGNRVFYNGAEGMERVRKGMFGFQVDTSTAYKIISETYTEREKCGLMEVNLFPLPPLCVATTKHAGYREMFSQRVGWQREVGILTRQRRLWLPQRPVCENMVSGFVSVGIMDFYPALLVLQYGVAGAVVVLALELLYFHRSRLWQRMICISQLSGAKPTSMYAVATDQISQKRSQLQRTGSRLK

>LmigIR25

LHNGTYGGMIELMQRNLSQVSGSSLIMKKNRWDVVDYTGPAWRFKSYLAFRHPTSQGLRSSLFAPFCSSVWVASGAVWLLIMSSLRLITWIGARQAGGIVQAESSWGAVVVFAACAIGLQGTTERSQWLSWRVVLLFTFLLSLMLNTYYGAAVVGSLLVPPPKTIRTLRHLIDSPIQVGIEDIGYNRDYLEKSTDPLVRELFVRKVFPPSAKRPHYYPIDVGVERMRTSLFAFQAEAV

>LmigIR25a

MEAGVTWVPDLQAETPEGVPAAAQGAPSAEKVHVNDDKNSIPNKALKQVVADLSKQGMIFDGVFKATANGSDVEALIDSMCLEYNTSIDENKKIHVVLDTTLQDVSSEAVKYFTRALELPTVSASCGQEGDLRRRLSWWENMYKKNLSTICLPRCVCVCICVGVGVFAYLPFSPQVIKTLLFSLYAVMDHKYKSLLQNVPTRHIINEIKFQNIANQLSTFKQREVFNYFILGRMDTVNKVLEAAADMEFYGRQFGWYAITQDEGNPSCQKCGKGASVLHVKPNDAEGTVVGAENPKLAYQFYYELFRNTFLAIGQMIKEESWPDMQYIPCEEYEENKNVPPTRKLNLLEALQQISMLNPGAYGQLMLSSNGHSHMQFNMTAFNVSLSDNSATEVGTWAADLDSPFITKVKPSVPVTQYTVVVALQQPFVIKYQDENGNTKFKGYCIDLINAIRNITNFEIEIYEVADGKFGNMDEEGRWNGMIKDLIDKKAHIALGALSVMAERENVVDFTVPYYDLVGITILMKKPKTPTSLFKFLTVLENDVWLCILAAYFFTSFLMWVFDRWSPYSYQNNREKYKDDEEKREFDLKECLWFCMTSLTPQGGGEAPKNLSGRLVAATWWLFGFIIIASYTANLAAFLTVSRLDTPVESLDDLSKQYKIQYAPIANSSAHGGGEAPKNLSGRLVAATWWLFGFIIIASYTANLAAFLTVSRLDTPVESLDDLSKQYKIQYAPIANSSAHVYFQRMAAIENRFYEIWKDMSLNDSLSEVERAKLAVWDYPVSDKYTKILQAMTEAGFPANIEEALERVRASKSSSEGFAFIGDATDIRYQVLTNCDLQMVGEEFSRKPYAIAVQQGSPLKDQFNNAILQLLNKRKLEKLKEQWWNQNPEKRNDCEKQDDQSDGISIQNIGGVFIVIFVGIGLACITLAFEYWWYKLRPQHNAVVEAAPPRTKSDSLQALNMMRSSFDKRYGRRQGVALAGVTNPW

>LmigIR26

MLGGKAGSVDAGATNLRQNVSRVPTCKRSCCPAADDDVAEALMFEIITITITQHAEYLITVTSTQTDNLHERLISENQKHLDPLARFNYALFLHLKEFYNFTFSVQATRSWGYKTKAGRFDGMMGVIQRNEADIGASSALIKKERLEIVDYAGHTWKFWPRFLFLHPSGQRLHTALLTPLSTKVWFCAILAGLMITLILNTSSYVHADNFCNLDGSWSSTLITTIGTFTLQGAGSSWSQISWRITLLTALLLASLLNIHYGAAVVGSLLIPAPHTIRTLQDLMESPLRVAFENVSYNREYVARTTDKLGRELIHRKKPEFVQLSEGVIKIRKGFFAFHTEGGSVFRLAAMTFTESQKCALSDVSLFTPAVMSMPVKKKSPLRELFARGWSTRRPNCDFLRQSLPMDGPMVEVKKIDSTNPWLKL

>LmigIR27

GTDLLCADHCRNHGFRRSLGGLILRAGTVVHDLPLVNLGHRLTNVEEKHVDTMARFGWELTTILSKKLNFTPVLYATDSFGYKQDTGNDTLDGLVGMLETGLVDVGSAGLAMFKDRLNRIDFVGPSRLWVSKIMFRHPTVAAEQGALFRPFTPGLWLGVMLVFAALALVSHAICRAHGWSAQRAWGDTFLLVASAVGQQGSSEGVQWPSWRLLLMVSLVCATLLDVHYSAAIVSSLLLPPPRTINTREDLLRSPLHFGIENISYAHDLIEMSEDPVIRSLYQKKVAPPGAVRPNYFTLEEGMTKVATEMFAFHSQEFQMYQLVEKLFTEQDKCVLVTIPLFPPQMTYVTVAKNSPLRETFTVGMRAMWEQGHLRHLRMRWHSKKPACMAERDFVNVDLATISPAFMLLLSATLISLLLLLRESAKARQTQDNTPVPLSH

>LmigIR28

LEALYRVHPSRPLTVRQAGSWTPHQGIVLHRSAVHNGWRLLDGLILGAAGVVVDTPTDRLGERLRVVQDRHLDTNIRFGWDLCDKLRFMFNFSVVLYKTESYGYLTPNGTIDGQAGMLCDGTIDLALSQLMLSHHRLDFIDFTTAPSRMWTLKTVFRHPASRAVYGTIFRPFSAALWLSSGLVFLLVLVAARLGCWAAAHNPADDSWSAAFLLVSSAISQQGTTLDTGRPSWRLVVFLSFSCALLLDTYYTAAIVTSLLLPPPRTINSKADLVHSQLAVGMENISYTHEYFEKSSDPVDHALMKYKVWPAGTARPNYHSLEAGVRKVAAEAFAFTGEDVSLYPLLDRYVTEADKCALVALDFMKSRSTYMPVRKNSPYRELLTIGLRRLLERGHLAR

>LmigIR29

LLKDLQGLALGAALPIVDTPLDNLGERLKNVNDRQLDTMARFGWGLSTTLAELLNFSIILYRVRNFGSLINDHEMDGAVALIHNGTVEFGAAGFIMTTRRMDFMDYTGPGRLWAPEIMFRHPKSASVLTTIFKPYTAELWVSSGALFVLILVVSRLFCWVEHKVTATVDEIDNSWNSTFLLVSSAIGQQGVSRSSEWLSWRMLLFVSFLCTNLLDTHYAAGIVSSLLMPPPRTINNKKDLADSTLGFGLENVSYTYQFFVKSDDPVDRALCSRKLYQAGGRANFFPAEVGVLKMAREPFAFHAEDVRVGPLIDRFFSDDDKCALVFIPLLTPVATYTAVRRNSPVKELFNFGLRMMWERGHVNYLRKAWYFTRVRCLSETEYASVDLVPMSPAFMLLGCAFLLSGFLL

>LmigIR76b

MQVSPVLRTVLTTVCSNYFLNGTKMEVPEGEPDPGCVLRIPKLMEGKTIRIGTLENPPLTMINKTDGTLIGHGVIFEIVDILKHKLGFNYEVVTPRANILGDENHGIIGLVHSRQVDLAVGYLPQFSQQARLVRHSESLAEAPWVFLMKRPLVSASGTGLLAPFDATVWYLVLASVVLMGPAIYFIILVRVRLCAGSERLTRIFPLSSCVWFVYGALMKQGSTLMPVTDSSRMLFATWWIFITLLTSFYTANLTAFLTLSRFTLQITSLKDIATKKAHWAAQKGSAMEYLVYNNDEYSFLNQSLQAGFGQFVDISDTDMLLRIKKDDLVYLREKQHVEHTMFRDYLEKTRNPKVEEKDRCTFVMTKQPFLHLPISFYYPLNSNLAHIFDPLLKALVETGIVRHLLRKDLPQIEICPLDLGSKERQLRNSDLYMTYMIVVTGFCAATVAFFGEILTRQVKRCIAEAELQTGPSTSYPDDWKTVKAQANRMPYTMYLNGNIINVKQPAYSITKDFQSSNRKSLSRQRNTNYVFQYTS

>OasiIR1

LGPPYLKEKTPTTPPRVGNDRYEGYSMDLIAQIADLLNFTFEFKLAPDGQYGSRDEKTGSWNGLVGELIAGRADLAICDLTITQERQSAVDFTMPFMTLGISILYKQPEKADPNLFSFLDPFTIDVWIYMATAYLGVSIIFFILARMAPGEWDKSHPCDPDPTELENTFNMINIFWFSTGSLMGQGCDLLPKAVSTRLIAGMWWFFTLIMIASYTANLAAFLTNSKLEAPIEGVQDLAKQTKIKYGTYGRGSTAAFFRNSNDSLYQRMWLVMKQARPDVFTADNQEGVERVKKEKGNYAFFMESTSIEYQTALNCDLRKVGGLLDSKGYGIALQRDSPFRTAVSGAVLTLQERGNLSALKTRWWKAPEGRECAEEDAAATDNNEMG

>OasiIR2

MFAAYIGVSLMLFVMGRISPYEWTNPYPCIEEPETLENQFTLSNSLWFTIGSLMQQGTEIAPIAVSTRMVAGIWWFFTLIMVSTYTANLAAFLTVESMYQPIKNVKDLADQNTIKYGAKRGG

>OasiIR3

MVGVVFAIFVTFFELLWDVGHKSLKEKIPFKTLLMEELRFVAKLHGTTKPVRKYNQDAEVEDTNNFIPLSPYTNSYGFVDSKEPLT

>OasiIR8a

MWPLWMSVVAAQLQLATSQAASDPLLIRFLLVTEVNASWVGGELRANLSGLEARYAGLRLQLDVSAVEVDREHEVQEFQQKVCGALAAGVSALLDATWTGWRRLRGEAQRRGLPYLRLDATLANFVDAVDKYLHAREASDAALIFHTEEELDQALYHLIGNSVLRVIALNGLEKDTVTRLKDMRPVASYFVIFADTAHMAELYSKAAAGGLVRKAERWALVFTDWDWRSFRTDQLNLSTALLQMRPASCCALQAEPDSCKCALRKVAPAFLRAALSAAVDALAELHSKGLDVRAAPKQCSEGSGDDEDGDGGEEEDGVEPPANATGNYDAFLRAIAPRGQSNSTLFFRAAAAQLTFNTPLQLRMVNRSEDVSLGDWSPEKGLQLDKQLKPAKRFFRVGTAEGVPWSFPVRDEKTGDPLVGPEGEPIWDGYCIDLLKKLAEPTHMNFDYELVPAKDNDFGSRSPSGSWTGLVGDLAMGETDMIIAPLTMTSEREEVIDFVAPYFDQSGISIVIRKPVRETSLFKFMTVLRLEVWLSIVGALTVTGIMIWLLDKYSPYSAQNNKDMYPYPCREFTLKESFWFALTSFTPQGGGEAPKALSGRTLVAAYWLFVVLMLATFTANLAAFLTVERMKSPVQSLEQLARQSRINYTVVMNSDTHEYFRNMKNAEDVLYDVWKDITLNSSSDQSRYRVWDYPIKEQYGHILQAIDQAGPVPNASVGFQKVIDQEEGKFAFIHDAAQIRYEVSKNCNLTEVGEMFAEQPYAIAVQQGSHLQEEISRQILDLQKDRYFESLTAKFWNNSAKGTCPNSDDSEGITLESLGGVFIATLFGLALAMITLAGEIFYYKRKKLTAVNVTSSSAKVPKKQVTIG

>OasiIR25a

MTLLLLMVTLLSNLLSALGQTRISYLHVNDDRNLIPNKALKQVVSDLSKQGMIFDGVFKATANGSDVEALIDSMCLGYNTSIEENKKIHVVLDTTLQDVSSEAVKYFTRALELPTVSASCGQEGDLRYWRNIDKKQEKYLIQVMPPIDTIPEFIRSFCSEQNLTNAGILFDDTFIMDHKYKSLLQNVPTRHIINEIKFQNIGKQLSTFKQREVFNYFILGRMDTVNKVLEAAADMEFYGRQFGWYAITQDEGNPSCQKCGKGASVLHVKPNDAEGTVVGAENPKLAYQFYYELFRNTFLAIGQMIEEGSWPDMQYIPCEDYEENKNVPPMRKLNLLDSLQQISMQNPGAYGQLMLSSNGHSHMQFNMTAFNVSLSDSSATEVGTWAADLDSPFITKVKPSVPVTQYTVVVALQQPFVIKYQDENGNTKFKGYCIDLINAIRNITNFEIEIYEVADGKFGNMDEEGRWNGMIKDLIDKKAHIALGALSVMAERENVVDFTVPYYDLVGITILMKKPKTPTSLFKFLTVLENDVWLCILAAYFFTSFLMWVFDRWSPYSYQNNREKYKDDEEKREFDLKECLWFCMTSLTPQGGGEAPKNLSGRLVAATWWLFGFIIIASYTANLAAFLTVSRLDTPVESLDDLSKQYKIQYAPTANSSAHVYFQRMAAIENRFYEIWKDMSLNDSLSEVERAKLAVWDYPVSDKYTKILQAMTEAGFPANMEEALERVRASKSSSEGFAFIGDATDIRYQVLTNCDLQMVGEEFSRKPYAIAVQQGSPLKDQFNNAILQLLNKRKLEKLKEQWWNQNPEKRNDCEKQDDQSDGISIQNIGGVFIVIFVGIGLACITLAFEYWWYKLRPQHNAVVEAAPPRTKSDSLQALNMMRSSFDKRYGRRQGVALAGVTNPW

>OasiIR76b

MKRPLVSASGTGLLAPFDATVWYLVLASVVLMGPAIYLIILVRVRLCAGSERLTRIFPLSSCVWFVYGALMKQGSTLMPVTDSSRMLFATWWIFITLLTSFYTANLTAFLTLSRFTLQITSLKDIATKKAHWAAQKGSAMEYLVYNNDEYSFLNQSLQAGFGQFVDISDADMLLRIKKDDLVYLREKQHVEHTMFRDYLEKTRNPKVEEKDRCTFVMTKQPFLHLPISFYYPLNSSLAHIFDPLLKALVETGIVRHLLRKDLPQIEICPLDLGSKERQLRNSDLYMTYMIVVTGFCAATVAFFGEILTRQVKRCIAEAELQTGPAASYPDDWKNVKAQANRMPYTMYLNGNIINVKQPAYSITKDFQSSNRKSLSRQRNTNYVFQYTS

Table S5 Amino acid sequences of 24 SNMPs of *Ceracris nigricornis* and other insect species used to construct phylogenetic tree.

>CnigSNMP1

MQLPVGLAAGGGGVFFLAVAAGWYGMPKLIHSQIANGLALKKGSDIRQMWSNFSDPIDFRVYMLNLTNPEAVHRGEKPIVQEIGPYFYEEYKQKVKLRDHKEDDTVSYNQKVTWLFNQAKSGPGLTGDEVITMPHPLLLGLLLTLERDKPGMLALVNKAIPPLFRKPESIFVTAPARNFLFDGIVINCTVTDFSAKALCTGLKKEAKELKREGDNFFFSFFGHKNGTVDNNRLRVKRGIENIDELGRVVAFNGENKLATWRGDPCNDLRGTDSTIFPPFITPNDTIHIFAHDICRSMHADFEREQQVSGVRGLRFVASGSLLRQGGPNACTCPDGRCLPTGAISVRECFRAPIAVSYPHFYQASPEYLQYAEGLSPSKDLHETFVVIEPESGTPLLGAKRLQFNMKVARVQQVPAMASVSEGLFPLLWVDEGVELEEATLSQVRALFVARASLGGVAWAVLAVGVAAMLFCAFRLAKGKMKERNTSLALDKGAAAGGKMAVPTLGAAYPESAPRRASPPHAAPAAPVDATHF

>CnigSNMP2

MLSARVCGCGCGRRGLLWGLAAGLAVLALAVALKWAAFPYILTAKIIQAVQLQDGSAAMARFEQLPQPLLFKAYLFNVTNPGQVALGAKPVLRQVGPYVYEEWRRRRNVTRLADGSLSYRLETSYHFSPQRSPGLSEDDEFTYLNVVMMGIVVQVAEDYASMLSLVEPVLAELLSGGAQLFLRSSARRLLWAGEPAVDCRGNLSAVAALACSALPSLLPQTVTQAEPGLYAFSFFGFKNGTSRQLWRVDSGLKEVARLGSVLAYDNSSRLKVWSPSSSPCNDIKGTDSTIFP

>CnigSNMP2a

LSLGAAWERPAEYMGVPGNRYTGEMPDMKGNAEHHCYCPTEDTCLEKGALDLSPCAGAPVIATLPHFYLSSETYLRTVTGLNPSKENHELFMVFESTTGSPMEARKRLQFNMFLHKINKIDLLANVPYALMPLIWVEEGLALEEKYVSTLRMLFRMQGIMSGVKWTLMAMGMGMGGAGGYLYFKRRKELVVGPAEPKKVAAGHSDAPGHPIRLESSHSRY

>AaegSNMP1

MLIKNRKNLMLKPGTQMRGMFEKIPFPLDFKLYLFHVTNPDVVMKGGKPRVREIGPYFFEEWKEKYDTVDNEEDDTLTFTLKNTWIFRPDLSKPLTGDEMITIPHPLILGALLMVQRDREAMMPLVSKGMDIIMNPLTTGFLTTRVMDLLFDGILIDCSSHEFSAKALCSGLESEGAVMPFNETHFKFSMFGLKNGTDAGRWVVYRGVKNIMDLGRVVSFNDETEMDIYDGDECNRYIGTDSTIFPPFLTTKDKLWAWSPEICRSIGAEYGGKSKYAGLPMSFFKLDFGDARNEPEHHCFCRDPPDICPPKGTIDLAPCLGAPIIGSKPHFYDSDPKLLAAVDGLTPNEKDHDVYIHFQLLSGTPVSAAKRLMFSMEIEPIRDHAVLGNLPTVILPLFWAEEGASLNKTWTNQLKYTLFLGLRFNTAVKWLTIIIGTIGTIVGGFMHYKRTTKMVNVTPVQSVNGSSAKGKGAGMTVVGHQPDSKGGSVTAPVIPSAKDLLQNSRNLPTVIEGLDKPQKVTVTEMQERY

>AaegSNMP2

MMVMNTELRQDTPQFKRWEAVPQPLDFKVYIFNVTNPYEVQMGRRPRVVEVGPYVYFQYRHKDNIRFSRDRSKVHFSQQQMYVFDAESSYPLTENDQLTVLNMHMNSILQIIDTQAKETITNFRSDVNNTLEKIPVVRVIKRIIEKTTPIQSILQLAEDETYDSLRLINAELNRIFGRPDSMFLRTTPREFLFEGVPFCVNVIGIAKAICKEIEKRNTKTIRVQPDGSMKFSFFNHKNMTNDGTYTINTGIKEPALTQMIEYWNGRNTLDRWINQSAGSSSKCNKIVGTDGSGYPPFREGVERMTIFSSDICRTVDIKYVGPSSYEGIPALRFETDSHFLNEIGPEYGNDCYCVNRIPKAIVKNNGCLYKGALDLSTCFDAPVVLTHPHMMGAAQEYTSLIDGLYPDPEKHQIFVDVEPLTGTPLNGGKRVQFNMFLRRIDSIRLTDRLQTTLFPVLWIEEGIALNEDMVKLIDDSLMKVLTLLDIVQWVMIGSGLLLAIIMPIVYFIKRRPSSGSITPTLTTTTSTVSISDGGGLGGNPQK

>AlinSNMP1a

MGAPLRLGVAGGALFLFGSVFGFWGFHKFLNSQIAQTVQLKKGNEMRDTWATFPVALEFKVYLFNLTNPEEVQNGGKPKVQEVGPYFFDEWKSKGNFEDDSAEDTVSFNMKAVWYFQKDRSEGLTGDEMITIPHPVVFSMIAQVERDKPGALPMLAKALPALFNNLTSPFIAARAMDILFDGLPINCSSKEFGPKAVCTLINANPKGLIKKSPELFLFSFFGPKNGTLDEGRFTVKRGINDPKEVGLMVKYNNKTKLDVWAGPECNTLSGTDSTIFPPFIDDSEDIVSFSPDLCRSLGAKFRYKITYKGVPGNHYTADLGDMSANEDEKCYCPTPTTCLKKGAMDITKCAGAPIILTLPHYYLADPSYLDEVEGLHPEEEKHQIFLNFEPITGTPLGARKRLQFNIKSHPVKKIPFMKSLPTTMIPLMWIEEGLELDQKFIDILNANLFRVMKIVGVSKWVMMLLGLGMGGFGAFLYYKRKGEAGQPSEKSPTPKTVQVESISGKF

>AlinSNMP1b

MPSQKSESRNSESKMSQYPRVSQVSKPTKGSRATSPVFSNLMERMREMPTKIKEAPPRQFGKFGAAMVAGGVGFGWVAFPYILSFAISKMVNLAPGGEIHDIWKDIPQSLDFNIWIWNVTNPMEVQNGGKAVLQEVGPYRYIEWKKKVDLIDNPADDEITYSSLNTWYFQKDRSYPLTGDEIVTIPHLPLMSMLLVAEQDFPPAMMTVLNAAIPRIYGKLDSVFMQIKAKDLLFDGYPIDCTSRDLIGRTVCVAVKANSKPLVKNGRNKYLFSVLGTKNATPEDVRITVKKGTVNTYDIGKVVKVNGNPMNSVWKDECNVLDGTDATIFPPYRSADNVSIVAYATDICRSIRGTYIGEGSYNGVRGHQYAVDLGDMSSNPKDVCYCIKKCYKKGTVDLTKCQGAPLVGTLPHFYLADESYLDGVIGMKPDREKHQITFIMEPITGVPLLARKRFQFNVDMHPIRFVNVTKNIRPTLFPILWVEEALDLGPELMGFLQARLLTNLTLVDIVKWTLIVVGAGIGIMGIVKHQMEKEQRKKHERGASVSPAPSNASQERLVGQSAFRSDSEFSFKSSEMLMDPARLTGASKTTPPPLIPHPHIPTPPQVFTLERSLQERLSPEVEGIPPVEVPPSRLSVVTSVTPVEESAPAAGAQPGSKPASGKSKK

>AlinSNMP2a

MMRNGWTSVDLRMGNIHINRVLYLGAFGAVIFIIGLFFATSGTDMMINSKIKKGIVLEEGSEGLKRFQKTPFPLEFKVFLFNITNTDDVMMGGKPVLTEMGPYTYDLYKEKPELKFLKDGMIEYNMTYQFHFNAQKSRGSESDMVTGLNVPLLGTATMVEQTFPMGLGFLNNAIPFLFPNITDIFVTTTVKDLLFDGILLRCNYTSGPAMPICNGLKGRAPPTIWREEETKNYRFAMFRHKNKTSEGPYKVKTGKGDVTEVGQIVEYQHRQTLKNWDKNSSCTIIKGTDTTIFGPLKNPHDDLYIFVPDVCLSFTANYVNTSIQNGIPLNKYFAAEKNMASYSKDPDNLCRCAKDDEGVRHCLKDGVIDASPCQGAPVIMSNPHFLDADAEYQNAVVGLKPIEEKHKTFVMLEPKTGAPVEGRKRMQMNLKVKKVNSITLLENVTERIIPLLWIEEGTRLEGPLLQELQKLYHVMGLLGTFSWVLLVAGLVIMGIAGVLYLKVRHLFCFAGTQIVAPVDSSIGGAQKMNTFGVTNQGSDDYQEHGYPGTAIYPQLGDGQGKNGDLVHTVAHPQAR

>AlinSNMP2b

MPSLNFVNELLELGFPELLRDNYAMRLNRTVDELLFSGITTHCPPNASLSAATVCSILRHFPGLKSLQKYPNGDMNVGIMRFKNDTLSDTYEVYRGNHDFDKIGQIVTLNGQQSVDNWYGDECNKVAGSYGETLLKPFLTEDSTMKVYGSDLCSSLPVGFKETSSYEGVDSFKFGPQKKFLGSVVDYPENYCYCPGSIDGITLGQGCMKAGAMEFSACQAVPVVLSFPHFYKASSHFQNAVGGLDPDSDKHESYIHLEPITGIPLKGVKRIQINFQMKGTPAMKITKNARDTLIPFLWVEEVAALGDDQVNLLKDMLLKMLKILSIVRWVLIAVGSLMVLVGCVMSFLSARKEHRHQY

>AmelSNMP1.X1

MRFKKLIHDITTRFFHRGVHVILHSSFVKLQSFVTTGRKLHETLRVPVGRKCVTMKPKKLGIIGGSLLAFGILICAIAFPPFLRSQVKKQIALKDGSEMRELWSNFPVPLDFKIYLFNVTNPMEITAGEKPILEEVGPFFYDEYKQKVDLVDREEDDSLEYNLKATWFFNPSRSEGLTGEEELIVPHVLILSMIKLTLEQQPAAMGILNKAVDNIFKKPESVFVRAKAREILFDGLPVDCTGKDFASSAICSVLKEKDDALIADGPGRYLFSLFGPKNGTVLPERIRVLRGIKNYKDVGKVTEVNGKTKLDIWGEGDCNEFNGTDSTIFAPLLTEQDDIVSFAPDICRSMGARFDSYTKVKGINTYHYKADLGDMSSHPEEKCFCPSPDSCLTKNLMDLTKCVGAPLIASLPHLLGAEEKYLKMVDGLHPNEEEHGIAMDFEPMTATPLSAHKRLQFNLYLHKVAKFKLMKNFPECLFPIFWVEEGILLGDEFVKKLKTVFKTISIVGFMKWFTIVSGTCVSGAAAALFFKNKDKNKLDITKVTPQKGEEKKWPNQMTISTIQSAAVPPNLDAD

>AmelSNMP1.X2

MKPKKLGIIGGSLLAFGILICAIAFPPFLRSQVKKQIALKDGSEMRELWSNFPVPLDFKIYLFNVTNPMEITAGEKPILEEVGPFFYDEYKQKVDLVDREEDDSLEYNLKATWFFNPSRSEGLTGEEELIVPHVLILSMIKLTLEQQPAAMGILNKAVDNIFKKPESVFVRAKAREILFDGLPVDCTGKDFASSAICSVLKEKDDALIADGPGRYLFSLFGPKNGTVLPERIRVLRGIKNYKDVGKVTEVNGKTKLDIWGEGDCNEFNGTDSTIFAPLLTEQDDIVSFAPDICRSMGARFDSYTKVKGINTYHYKADLGDMSSHPEEKCFCPSPDSCLTKNLMDLTKCVGAPLIASLPHLLGAEEKYLKMVDGLHPNEEEHGIAMDFEPMTATPLSAHKRLQFNLYLHKVAKFKLMKNFPECLFPIFWVEEGILLGDEFVKKLKTVFKTISIVGFMKWFTIVSGTCVSGAAAALFFKNKDKNKLDITKVTPQKGEEKKWPNQMTISTIQSAAVPPNLDAD

>AmelSNMP2

MWSYQVCAIICVIFGIYACITNLFSDGLFSIKNAILKNLPLIKGKDMYDEWILPVNLIFKCYFFNVTNPDEVMEGNNPNLVEYGPFTYREVFEKQIVDVDEELDEIIYDVKSTFTFDKYASLNISKRDTVTILNPAYIGTISMASIIGLTTLPPSYIEKFGNNIPKLFPNRSSIFLKANPKEILFDGVKLTCNERKFPELSTICKTLKALRSPVLKEGEKEGVYYLSIFQRVNGTIRGRFSVNRGVNNISELGNIGSYNGRRVQTIWRTEKCNTVRGSDTITWAPLINPMPSVLSFIPDLCRSIEADYDKEVSIYGLIGSRFVMRERTWFLNQSQCYCLERNKVPNCLPQGLIDVSDCLVMLRYVMLQKVPIIMSEPHFLHGDPQLLMYALGLNPSEDLHETFIVIEPYTGTPLSGQKKIQLNLKLERQPVDLLSNISEGYFPLLWCANVRIFSKIIKLQY

>BmorSNMP1

MQLAKPLKYAAISGIVAFVGLMFGWVIFPAILKSQLKKEMALSKKTDVRKMWEKIPFALDFKIYLFNYTNAEDVQKGAVPIVKEVGPFYFEEWKEKVEVEENEGNDTINYKKIDVFLFKPELSGPGLTGEEVIVMPNIFMMAMALTVYREKPAMLNVAAKAINGIFDSPSDVFMRVKALDILFRGIIINCDRTEFAPKAACTTIKKEAPNGIVFEPNNQLRFSLFGVRNNSVDPHVVTVKRGVQNVMDVGRVVAIDGKTKMNVWRDSCNEYQGTDGTVFPPFLTHKDRLQSFSGDLCRSFKPWFQKKTSYNGIKTNRYVANIGDFANDPELQCYCDSPDKCPPKGLMDLYKCIKAPMFVSMPHYLEGDPELLKNVKGLNPNAKEHGIEIDFEPISGTPMVAKQRIQFNIQLLKSEKMDLLKDLPGTIVPLFWIEEGLSLNKTFVKMLKSQLFIPKRVVSVVCWCMISFGSLGVIAAVIFHFKGDIMHLAVAGDNSVSKIKPENDENKEVGVMGQNQEPAKVM

>BmorSNMP2

MLAKYTKTIFSVSVAFLVVSIVLATWGFPKIIRKQIQKNVQISNTSKMYDKWVKLPMPLDFKIYVFNVTNRDAINQGEKPNLKEIGPYVYKQYREKIILGYGDNDTIKYNLKKTFVFDPVASGDLREDDELTVINFSYMAAIISVQEMMPAAVGMINRALEQFFTNLTDPFQTVKVKDLFFDGLFLNCEGDNTALGLICGKIRAEKPPTMRISKSANGFYFSMFSHMNRTVSGPYEMVRGTENLSDLGHVISYQGKRIMSAWDDQYCGQLNGTDSTIFPPLEDGNIPEKLYTFEPDICRSLFASLVGKDTLFNISTYYYEISDMTLGSKSANPDNKCFCKRNWSVKHDGCLLMGVLNLAPCQGAPAIASLPHFYLGSDELADFFGDGIKPDKEKHNTYVHLDPITGVVIKGVKRLQFNIELRNVPSVPQLKEVPSGLFPLLWIEEGAEIPEWLRKEIMDSHTMLWYVDAARWLVLAVAVVAVLVSATLVARSAALIPWPRNSNSISFILGNSVNTSKVHS

>DmelSNMP1

MQVPRVKLLMGSGAMFVFAIIYGWVIFPKILKFMISKQVTLKPGSDVRELWSNTPFPLHFYIYVFNVTNPDEVSEGAKPRLQEVGPFVFDEWKDKYDLEDDVVEDTVSFTMRNTFIFNPKESLPLTGEEEIILPHPIMLPGGISVQREKAAMMELVSKGLSIVFPDAKAFLKAKFMDLFFRGINVDCSSEEFSAKALCTVFYTGEIKQAKQVNQTHFLFSFMGQANHSDSGRFTVCRGVKNNKKLGKVVKFADEPEQDIWPDGECNTFVGTDSTVFAPGLKKEDGLWAFTPDLCRSLGAYYQHKSSYHGMPSMRYTLDLGDIRADEKLHCFCEDPEDLDTCPPKGTMNLAACVGGPLMASMPHFYLGDPKLVADVDGLNPNEKDHAVYIDFELMSGTPFQAAKRLQFNLDMEPVEGIEPMKNLPKLILPMFWVEEGVQLNKTYTNLVKYTLFLGLKINSVLRWSLITFSLVGLMFSAYLFYHKSDSLDINSILKDNNKVDDVASTKEPLPSANPKQSSTVHPVQLPNTLIPGTNPATNPATHHKMEHRERY

>DmelSNMP2

MIHWSLIVSALGVCVAVLGGYCGWILFPNMVHKKVEQSVVIQDGSEQFKRFVNLPQPLNFKVYIFNVTNSDRIQQGAIPIVEEIGPYVYKQFRQKKVKHFSRDGSKISYVQNVHFDFDAAASAPYTQDDRIVALNMHMNAFLQVFEREITDIFQGFANRLNSRLNQTPGVRVLKRLMERIRGKRKSVLQISENDPGLALLLVHLNANLKAVFNDPRSMSVSTSVREYLFDGVRFCINPQGIAKAICNQIKESGSKTIREKSDGSLAFSFFGHKNGSGHEVYEVHTGKGDPMRVLEIQKLDDSHNLQVWLNASSEGETSVCNQINGTDASAYPPFRQRGDSMYIFSADICRSVQLFYQTDIQYQGIPGYRYSIGENFINDIGPEHDNECFCVDKLANVIKRKNGCLYAGALDLTTCLDAPVILTLPHMLGASNEYRKMIRGLKPDAKKHQTFVDVQSLTGTPLQGGKRVQFNMFLKSINRIGITENLPTVLMPAIWVEEGIQLNGEMVAFFKKKLISTLKTLNIVHWATLCGGIGVAVACLIYYIYQRGRVVEPPVK

>OasiSNMP1

MQLPVGLAAGGGGVFFLAVVAGWYGMPKLIHSQIANGLALKKGSDIRQMWSNFSDPIDFRVYILNLTNPEAVHRGEKPIVQEIGPYFYEEYKQKVKLRDHKEDDTVSYNNKVTWLFNRGKSAPGLTGDEMVTMPHPLLLGLLLTLERDKPGMLALVNKAIPP

>OasiSNMP2a

MLSARVCGCGRRGLWWGLAAGAVVLAAALVLRWAAFPAILTAKITQAVQIQDGSAAMERFVQLPQPLLYKAYLFNVTNPDQVSLGAKPALQEVGPYVYEEWRRRRDVTRLEDGSLSYRLETTFRFSPERSPGLSEDDEFTYLNVVMMGIVVQVAEDYASMLTMVEPVLSELLSGGAQLFLRATARQLLWDGVPTVDCRGNLSAVAVLACSALPSLLPPTVQQAQPGVYAFSFFGFKNGTARQWWRVDPGLSDVRSLGAVLSYDNSSRLKVWTPASSPCNDIRGTDSTIFPPFRDPKEPIVAFGADLCLSLGATWERSAEYMGVPGNRYTGEMPDMTGNPEHHCYCPSETSCLEKGALDLSPCAGAPVIATLPHFYLSSESYLRTVSGLQPTKEHHELFMVFESTTGSPMEARKRLQFNMFLHKINKIDLLANVPYALMPLIWVEEGLALEEKYVSTLRMLFKMQGIMSGVKWTMMVVGMGMAGAGGYLHFKRRKELVVGPAEPKKVAAGHAEPPGHPIRLESSHSRY

>OasiSNMP2b

MLSARVCGCGRRGLWWGLAAGAVVLAAALVLRWAAFPAILTAKITQAVQIQDGSAAMERFVQLPQPLLYKAYLFNVTNPDQVSLGAKPALQEVGPYVYEEWRRRRDVTRLEDGSLSYRLETTFRFSPERSPGVSEDDEFTYLNVVMMGIVVQVAEDYASMLTMVEPVLSELLSGGAQLFLRATARQLLWDGVPTVDCRGNLSAVAVLACSALPSLLPPTVQQAQPGVYAFSFFGFKNGTARQWWRVDPGLSDVRSLGAVLSYDNSSRLKVWTPASSPCNDIRGTDSTIFPPFITPNHTIHIFAHDICRSMHADFEREQEVSGVRGLRFVASRSLLRQGGRNACTCPEGHCLPSGAISVKECFRAPIAVSYPHFYQAEPEYLQYAEGLSPSKELHETFVVIEPESGTPLLGAKRLQFNMRAARVPQVPALANLTDGLFPLLWVEEGVELEEAELSQVRALFVARASLGGVAWAVLAGGVAALLFCAYRLARGRLRERNTTLALDKGAAAGGKLSVPTLGAAYPESAPRRASPPPAAPVDATNF

>SgreSNMP1

MQLPVGLAAGGGGVFFMAVVAGWYGMPKLISSQIASGLALKKGSDIRQMWSNFSDPIDFRVYVLNLTNPEAVHRGEKPIVQEIGPYFYEEYKQKVKLRDHKEDDTVSYNNKITWLFNQGKSAPGLTGDELVTLPHPLLLGLLLTLERDKPGMLALVNKAIPPLFRKPESIFVTAPVRNFLFDGIVINCTVTDFSAKALCTGLKKEAKELKREGDNFFFSFFGHKNGTVDAGRLRVKRGIQNIDDLGRVVAFNGEPKMSAWRGDPCNDLRGTDSTIFPPFRDPKEPIVAFGPDLCLSLGANWERKAEYMGVPGNRYTAELPDMKGNPEHHCYCPTEQTCLEKGTLDLSPCAGAPVIATLPHFYLASETYLQTVSGLQPTKENHELFMVFESTTGSPMEARKRLQFNMFLHKINKIDLLANVPYALMPLIWVEEGLALEEKYVSTLRMLFRMQGIMSGVKWTLMAVGMGMAGAGGYLHFKRRKELVVGPAEPKKVVAGHDTTGHPIRLESSHSRY

>SgreSNMP2

MLSARVCGCGRRGLWWGLAAGAALLAVALVLRWAAFPAILTAKIKQAVQLHDGSPAMERFVQLPQPLLYKVYLFNVTNPDEVEQGAKPVLQQVGPYVYEEWRRRRDVTRMANGSLDYRLETTYHFSPERSPGLSEDDEFTYLNVVMVGIVVQVSEDYSSLLSMVEPVLSELVPGGAQLFQRASARQLLWSGVPTVDCRGNLSAVATLACGALPSLLPATVQQTEPGVYVFSFFGFKNGTSKQWWRVDSGVEDVRTLGSVISYDNSSRLKVWSPSNSPCNEIRGTDSTLFPPFITPNDTIYIFAHDICRSMHAEYEREQDVSGVHGLRFVASGSLLRRGGPNACTCPDGRCLATGAISVRECFRAPIAVSFPHFYQASPEYLQYAEGLSPNKELHETFVVIEPETGTPLVGAKRLQFNMKAVRVSQVPALRNVSDGLFPLLWVEEGVELEEKQLSQVRALYVARASMGGVAWAVLAVGVAALLFCAVRLAKARVAERNRSLSLEKGVTAGGKLSVPTLGAAYPESATKRPSPPAAAPAASTAPAAPVDATHF

>TcasSNMP1

MIKGKVKSMINLNKGSEIRQMFVKVPFALDFKIYMFNVTNPMDVQKGALPVLKEVGPFCFEEWKEKVDLDDNDDEDVMFYNPKDTFYKANGPGCLDGSQMITMAHPLILGMVNTVVRTKPGAISLISKAINSIYGNPDSIFMTASAMDILFDGVVIKCGVKDFAGKAVCSQLKEAPDLRHVDENDLAFSFIGPKNATPGKRFKVLRGVKESHDVGRILEYDNKKEMEVWPTKECNQYKGTDGTVFPPYLTKEEGLASYAPDLCRSLVAVYSGDTKYDGIPVRIYTATLGDMSKNADEKCYCPTPDTCLKKGMMDLFKCAGVPVYVSLPHFYESDESYVKGVVGLNPNKKDHGIQILFESTTGGPVKAAKRLQFNMPLEPNPKLPIFANLPNTVLPLFWVEEGVALNNTFTKPLKDLFKIMKIVKIAKWLIMLGCLGGLGAAGYLYFSKKGEANITPVHKVKPAENGVSTLGGEVNHAMSDNEIEKY

>TcasSNMP1-like

MVKWQRQLKPGNEVRDFYIKLPIPLDFRVYFFNISNPEEVKQGEKPILKQIGPYCYDAYKEKINVEDDKDNDTLTYNPYDTYFFNQMRTGDLSQDDYVTILHPLTVGIVNAVATQKPQYLSAVNKALPVIFKENSSIYLTAKVREILFDGVLINCNVKDFSANAVCSQFKGQPAMVEVEKNIYSFSLLGSRNGSIPTRITIHRGVKNAADIGRVVTIDNKTDLDVWPEPECNAFRGTDGWVFPSFLEKEDGIWTVASDLCRSFKAQYVEDLKFHGVVVRKYFADLGDMSSNPAEKCFCPAPEKCLPKGVMDLTKCMKVPLYCTLPHFLRADEKLLQQVEGLSPELERHIIKIYFEPLTGTPMLGQRRIQFNLQLMPIPKVAMMKTVPEALHPILWIEEGVELEGFLLKKVTSVFTLLKLMTFVRYIMLGLSIQGILYGGYKLYQESKSKKVSPVQNGTTESKNHNQGKTGGIELPSMNKRNKENTKNA

>TcasSNMP2

MDIVNDALPFLYPGIKNIFVTNTVRNILFDGVTMSCGSDEVAMICDGLKKRRPPSIRPADNNKDYLVAMFHHMNGSVDGPYEMQRGLKDSSKGQVVGFKDNNMLTLWTGDCNTIQGTDLTLNPNLNDLPPKIYFFASDFCRSFSVKFDKELVYLGLKSYKFKNSNLFHIEKNCFCDKNPENEVPGCTPAGTMDVSPCTGSSVVLSQPHFLNAEKSLLDEAQGLAPNENRHGTFIIMEPKTGLALVVKTRFQMNVYLQDFEDVDLLANVSAGFFPLLWLENVWRCYLRLF
